# Cancer diagnoses, referrals, and survival in people with a learning disability in the UK: a population-based, matched cohort study

**DOI:** 10.1016/j.lanepe.2025.101519

**Published:** 2025-11-14

**Authors:** Oliver John Kennedy, Umesh Chauhan, Louise Gorman, Paul Lorigan, Samuel Merriel, Tjeerd Van Staa, Alison Wright, Darren Mark Ashcroft

**Affiliations:** a Division of Cancer Sciences, University of Manchester, Manchester, UK; b The Christie NHS Foundation Trust, Manchester, UK; c School of Medicine, University of Lancashire, Lancashire, UK; d National Institute for Health and Care Research (NIHR) Greater Manchester Patient Safety Research Collaboration (GM PSRC), The University of Manchester, Manchester, UK; e Centre for Primary Care and Health Services Research, University of Manchester, Manchester, UK; f Centre for Pharmacoepidemiology and Drug Safety, Division of Pharmacy and Optometry, School of Health Sciences, Faculty of Biology, Medicine and Health, University of Manchester, Manchester, UK; g Manchester Academic Health Science Centre, Manchester, UK

**Keywords:** Learning disability, Intellectual disability, Cancer

## Abstract

**Background:**

People with a learning disability (LD, also known as intellectual disability) face poorer health outcomes, yet the burden of cancer in this population is poorly understood. This study investigated cancer-related outcomes in people with a LD compared to the general population.

**Methods:**

A matched cohort study was conducted using linked primary care, hospital, mortality, and cancer registry data from Clinical Practice Research Datalink (CPRD) Aurum. In total, 180,911 individuals with a LD were matched with 3,405,467 controls. Outcomes included urgent suspected cancer (USC) referrals, cancer diagnoses, treatment within six months, and overall survival (OS) post-diagnosis.

**Findings:**

Individuals with a LD had fewer USC referrals within 28 days of possible cancer symptoms (adjusted risk ratio [aRR] 0.52, 95% confidence interval [CI] 0.49–0.55). LD was associated with several cancers, including sarcoma (adjusted hazard ratio [aHR] 1.98, 1.65–2.39), central nervous system (aHR 3.42, 2.99–3.90), testicular (aHR 2.06, 1.61–2.62), and uterine cancers (aHR 1.69, 1.40–2.05) as well as cancer before age 50 years (aHR 1.74, 1.63–1.86). Absolute incidence was lower in individuals with a LD compared to without (3396 [1.9%] vs 67,506 [2.0%]) due to increased all-cause mortality (aHR 3.19, 3.12–3.27). LD was associated with fewer diagnoses via USC referrals (aRR 0.81, 0.76–0.86), fewer treatments within six months (aRR 0.83, 0.80–0.85) and shorter OS (median 4.4 years, 95% CI 3.9–5.1 vs 9.1 years, 8.8–9.5; aHR 1.73, 1.65–1.83). Melanoma, breast, and prostate cancers were less common but had up to a fourfold increased risk of death after diagnosis in individuals with a LD.

**Interpretation:**

Individuals with a LD have higher cancer risk, more diagnoses outside USC pathways, fewer treatments, and poorer prognosis. Fewer diagnoses of some cancers, alongside worse outcomes, may indicate under-investigation. As premature all-cause mortality improves, cancer burden in this population may rise disproportionately.

**Funding:**

10.13039/501100000272NIHR Greater Manchester Patient Safety Research Collaboration (NIHR204295).

**Research in context**

**Evidence before this study**

We searched PubMed from database inception until January 15, 2025, for relevant studies on cancer incidence, diagnosis, and survival outcomes among people with a learning disability (LD), using the terms (“cancer” OR “malignancy” OR “neoplasm”) AND (“learning” OR “intellectual” OR “developmental”) AND (“disability” OR “disabilities”). Previous studies produced inconsistent findings regarding cancer risks in individuals with a LD, often limited by small sample sizes, inconsistent definitions of LD, and insufficient adjustment for premature death as a competing risk. While the existing evidence suggested later cancer diagnoses and potentially worse survival outcomes overall in people with a LD, no quantitative estimates of survival times across all cancer types or by specific cancer sites were available. Nor was there information regarding early onset cancers, use of urgent suspected cancer referrals following symptoms or treatments received following diagnosis. Moreover, the influence of LD severity, including the impact of conditions such as Down syndrome, the most common genetic cause of a LD, remained unclear.

**Added value of this study**

Our large, population-based cohort study, using linked primary care, hospital, and national cancer and death records from England, compared 180,911 individuals with a LD to over 3.4 million matched controls. We found that individuals with a LD had more than a threefold higher risk of all-cause mortality, rising to more than tenfold among those with Down syndrome. Elevated risks were observed for several cancer types, including sarcoma, central nervous system, digestive tract, testicular, gynaecological, unknown primary, endocrine, and haematological cancers. Nevertheless, individuals with a LD were approximately half as likely to be referred for urgent investigations after presenting with symptoms suggestive of cancer. Although overall cancer risk was higher, the cumulative incidence in this group was lower than expected due to premature deaths, suggesting the burden of cancer may rise disproportionately as premature mortality from all causes is reduced. Among those diagnosed with cancer, people with a LD were less likely to have received an urgent cancer referral, were more often diagnosed with advanced or unstaged disease, were less likely to receive treatment (surgery, radiotherapy or systemic anticancer therapy) within six months, and had significantly shorter overall survival, particularly in those with a severe LD or Down syndrome. Melanoma, breast, and prostate cancers were less frequently diagnosed but had up to a fourfold increased risk of death after diagnosis, highlighting potential underdiagnosis and inequities in access to timely and effective cancer care.

**Implications of all the available evidence**

People with a LD face an increased risk of cancer overall, with particularly high risks for certain cancer types, including at younger ages, which may have implications for screening. Efforts to reduce early deaths from all causes will likely reveal a greater cancer burden in this population, especially given that cancer is often diagnosed at a later stage and overall survival outcomes are poorer. There may be missed opportunities for earlier diagnosis given the reduced likelihood of urgent suspected cancer referral following symptoms. There is a need to understand why cancers such as breast, prostate, and melanoma are diagnosed less frequently, particularly in those with more severe LD, and how General Practitioners can help ensure timely investigations. The significantly poorer cancer survival outcomes and reduced rates of treatment highlight potential inequities, and it is crucial to examine how consent, best-interest decision-making, and reasonable adjustments are managed within clinical pathways to ensure equitable cancer care for this population.

## Introduction

Learning disability (LD), referred to in some contexts as intellectual disability, is defined by lower intellectual ability (usually an IQ of less than 70), significant impairment of social or adaptive functioning, and onset in childhood.^1^ LD affects 1–3% of the global population, including approximately 1.5 million people in the UK.^2^^,^^3^ Individuals with a LD frequently encounter barriers to healthcare access, such as communication difficulties, diagnostic overshadowing, and a lack of reasonable adjustments in clinical environments.^3^ These contribute to poorer health outcomes. On average, adults with a LD die 19–23 years earlier, and 42% of deaths are considered preventable.^4^ The NHS Long Term Plan, NICE guidance and the Learning from Lives and Deaths (LeDeR) programme all emphasise the need to tackle health inequalities for this group.^5^^,^^6^

Existing evidence suggests that cancer care and outcomes are poorer for individuals with a LD.^7^^,^^8^ In the UK, cancer screening uptake is consistently lower compared to the general population (bowel: 50.3% vs 66.8%; breast: 47.2% vs 61.9%).^9^ Screening tests may also be less effective. Only one-third of eligible women with a LD have adequate cervical smears, compared to nearly three-quarters without.^9^ Studies of cancer incidence have reported mixed results including lower, similar or higher rates among people with a LD.^8^ Cancer is often diagnosed late; one UK study involving deceased adults with a LD found that nearly 45% of cancers were identified at stage 4.^7^ Qualitative research also highlights a tendency for clinicians to offer less intensive or more palliative treatment to patients with a LD without a clear rationale.^10^

The current evidence base is limited by small or unrepresentative samples and inconsistent definitions of LD, which may lead to under-ascertainment. Few studies have examined cancer outcomes by LD severity or explored diagnostic pathways such as the urgent suspected cancer referral system. The distinct cancer profile of people with Down syndrome is also under-researched. Additionally, the impact of enhanced care for those on LD registers and the role of premature death as a competing risk remain unclear in population-level studies.

This study explored the relationship between LD and cancer outcomes in a large UK cohort. It examined cancer incidence among individuals with a LD compared to those without, including subgroup analyses based on LD severity, inclusion on a LD register, and the presence of Down syndrome. The study also assessed urgent suspected cancer referrals after symptoms suggestive of cancer, whether cancer cases were preceded by such a referral, receipt of anti-cancer treatment within six months of diagnosis, and overall survival.

## Methods

### Data source and study population

This study used anonymised general practice electronic health records from NHS patients in the Clinical Practice Research Datalink (CPRD) Aurum database,^11^ and linked data from the National Cancer Registration and Analysis Service (NCRAS), Office of National Statistics (ONS) and Hospital Episode Statistics (HES). The NHS is a publicly funded healthcare system free at the point of use. Registration with a general practice is mandatory to access NHS care, resulting in nearly the entire UK population being registered. CPRD Aurum includes data from 1784 nationally representative general practices in England using the EMIS Web electronic patient record system. The database covers approximately 25% of the UK population (total patients = 50,595,450; currently contributing patients = 16,680,848). It contains information on diagnoses, primary care consultations, prescriptions, laboratory results, and referrals to secondary care.

The study population included patients with a recorded Read, SNOMED, or EMIS code indicating a LD diagnosis between 1 January 2000 and 31 December 2018, who were eligible for linkage to NCRAS, ONS and HES data. The study end date coincided with the latest linked data release. Official estimates indicate approximately 2.16% of adults and 2.5% of children in the UK have a LD, yet only 0.5% of the population have a formal diagnosis with their primary care provider. To capture a more representative range of individuals with a LD, codes for explicit diagnoses of “learning disability”, “intellectual disability”, or “mental retardation” were used, as well as codes for clinical conditions associated with LD (e.g., Down syndrome, acrocephalosyndactyly, Sotos syndrome). This code set was developed from previous research^12^, ^13^, ^14^, ^15^, ^16^, ^17^, ^18^, ^19^, ^20^, ^21^ and manual searches of the CPRD data dictionary. Terms related to “learning difficulty” were also included, as UK legislation and government guidance often use this term interchangeably with “learning disability” (e.g., “profound and multiple learning difficulty”).^22^ Subgroups were defined for patients stratified by severity (mild, moderate vs severe), patients with Down syndrome and on the LD register. The LD register was introduced in 2008 as a funding linked scheme to improve identification, monitoring, and healthcare provision for individuals with a LD.^23^^,^^24^ Following previously established methods, the final code set was reviewed and agreed upon by two clinicians (OJK, UC) (Supplementary Code List 1).^25^

Eligible patients required at least one LD code recorded on or after 1 January 2000. Follow-up commenced at the date of LD diagnosis (index date). Patients were excluded if they had a prior cancer diagnosis or less than six months of continuous registration before their index date. Matched controls were identified using incidence density sampling; each patient with a LD was matched with up to 20 controls based on sex, age (±2 years), and the date of sampling. Comparators with prior LD or cancer diagnoses before the index date were excluded. Comparators began follow-up at the index date of the matched patient with a LD.

### Outcomes and statistical analysis

Study outcomes included all-cause mortality, incident cancers, “red-flag” symptoms, urgent suspected cancer referrals, stage at diagnosis (for cases registered with NCRAS using TNM or equivalent staging systems, categorised as stages 1–3 vs stage 4, and presence vs absence of staging information), treatment within six months (surgery, radiotherapy or systemic anticancer therapy; from 2014 to align with NCRAS treatment data) and overall survival (median, 1-year, and 5-year survival). Red-flag symptoms, also known as ‘alarm’ symptoms, are those specified in national guidelines^26^ as suspicious for a new cancer. A previously published code list was used to identify such symptoms.^27^ The urgent suspected cancer referral pathway (often called a “two-week wait”) was introduced in the NHS in 2000 and allows primary care clinicians to refer a patient with red-flag symptoms directly to a specialist clinic and be seen within two weeks. Urgent suspected cancer referrals were evaluated within 28 days of a new red-flag symptom (if no referral had occurred in the previous 12 months) and, among those diagnosed with cancer, within the year prior to diagnosis.

The analysis covered any cancer (excluding non-melanoma skin cancer) and the most common individual cancers: prostate, breast, lung, digestive tract (any, lower gastrointestinal [LGI], oesophageal, gastric, and hepatobiliary pancreatic [HPB]), haematological (any, lymphoma, leukaemia, and other haematological), renal, urinary tract (ex. renal), gynaecological (any, ovarian, uterine, cervical), melanoma, central nervous system (CNS), head and neck (any, oropharyngeal), sarcoma, endocrine, and testicular cancers as well as cancer of unknown primary (CUP). Outcomes were identified using primary care data in combination with NCRAS, HES and ONS data (Supplementary Code Lists 2 and 3).

Crude and age-standardised cancer incidence rates (using the 2013 European standard population) were calculated for individuals with and without a LD per 100,000 person-years at risk. Age-standardised rates account for differences in age structure that emerged during follow-up. Sex-specific cancers were analysed only within the relevant sex group. Poisson regression was used to estimate incidence rate ratios (IRRs) of red-flag symptoms and urgent suspected cancer referrals. In these analyses, each control's follow-up was limited to their matched case to allow for comparisons over equivalent timeframes. Modified Poisson regression was used to estimate risk ratios (RRs) of referral within 28 days of symptom onset, referral within 1 year prior to a diagnosis of cancer, stage at diagnosis, and treatment within six months of diagnosis. All regression analyses, including those below, were adjusted for age, gender, deprivation and ethnicity. Potential mediators, such as obesity and smoking, were not adjusted for. Robust variance estimators were used for standard errors and, where applicable, clustering was used to account for within-patient correlation (e.g., for IRRs).

Cox models were used to estimate adjusted hazard ratios (aHRs) for all-cause mortality, cancer diagnoses (including before age 50 years), and OS. All-cause mortality and cancer diagnoses were assessed from the index date and OS was evaluated from diagnosis. Individuals at risk were censored at the end of follow-up and, for cancer diagnoses, at death. For analyses of individual cancer types, sensitivity analyses were performed in which other cancers were censored at diagnosis and using Fine–Gray models to calculate sub-distribution HRs for cancer incidence, accounting for death as a competing risk. Kaplan–Meier methods estimated median, 1-year, and 5-year cancer survival rates. For survival analyses, log–log (survival) vs log–time plots were used to check proportionality assumptions, and stratification on ethnicity was employed due to that assumption not being met. Missing data (<5% for all variables, Table 1) were handled using multiple imputation by chained equations, and cases with complete data were evaluated in sensitivity analyses. All aIRRs, aRRs, aHRs, and survival estimates (median, 1-year, and 5-year OS) were generated with 95% confidence intervals (CIs). Analyses were conducted using R (Version 4.4.2). This study is reported in line with the recommendations of the RECORD statement.^28^

**Table 1:** Baseline characteristics of the included population.

	Learning disability (n = 180,911)	Matched controls (n = 3,405,467)
**Sex**		
Males	112,241 (62.0%)	2,109,355 (61.9%)
Females	68,670 (38.0%)	1,296,112 (38.1%)
**Age**		
Mean (SD)	23.9 (22.0)	23.6 (22.0)
<20	97,792 (54.1%)	1,873,394 (55.0%)
20–30	21,763 (12.0%)	398,861 (11.7%)
30–40	15,659 (8.7%)	285,567 (8.4%)
40–50	16,254 (9.0%)	300,863 (8.8%)
50–60	13,657 (7.5%)	254,891 (7.5%)
60–70	8304 (4.6%)	155,207 (4.6%)
>70	7482 (4.1%)	136,684 (4.0%)
**Ethnicity**		
White	145,425 (80.4%)	2,650,203 (77.8%)
Asian	15,062 (8.3%)	299,190 (8.8%)
Black	10,803 (6.0%)	182,729 (5.4%)
Mixed/Multiple	4584 (2.5%)	85,532 (2.5%)
Other	463 (0.3%)	17,802 (0.5%)
Unknown	4574 (2.5%)	170,011 (5.0%)
**Deprivation**		
1–Least deprived	23,904 (13.2%)	573,766 (16.8%)
2	28,742 (15.9%)	601,885 (17.7%)
3	33,642 (18.6%)	635,284 (18.7%)
4	42,231 (23.3%)	743,631 (21.8%)
5–Most deprived	52,144 (28.8%)	846,167 (24.8%)
Unknown	248 (0.1%)	4734 (0.1%)

Abbreviations: n, number; SD, standard deviation.

### Ethics approval

This study was approved by the Clinical Practice Research Datalink's (CPRD) independent scientific advisory committee (23_003009). CPRD also has ethical approval from the Health Research Authority to support research using anonymised patient data (research ethics committee reference 21/EM/0265). Individual patient consent was not required as all data were deidentified.

### Role of the funding source

The funder had no role in the study design, data collection, analysis, or interpretation; manuscript preparation; or the decision to submit for publication.

## Results

### Included population

The study included 180,911 individuals with a LD, of whom 112,241 (62.0%) were male (Table 1), reflecting previous studies and known gender differences in learning disabilities.^29^, ^30^, ^31^ The mean age at study entry was 23.9 years (standard deviation 22.0), with a median of 17 years (interquartile range [IQR] 5.0–40.0), indicating a predominantly young population but with substantial age variation. The matched comparison group consisted of 3,405,467 individuals without a recorded LD, closely reflecting the LD group by age, sex, ethnicity and deprivation. The most common LD codes were “on learning disability register” (n = 68,098, 37.6%), “learning difficulties” (n = 59,862, 33.1%), “learning disability” (n = 30,009, 16.6%), and “moderate learning disability” (n = 16,313, 9.0%). Nearly half the cohort (n = 85,123, 47.1%) had one LD-related code recorded during the study period, 38,381 (21.2%) had two codes, and 57,407 (31.7%) had three or more codes.

### All-cause mortality

During a median follow-up of 5.0 years (IQR 2.0–9.8), 9622 (5.3%) individuals with a LD died compared to 71,362 (2.1%) matched controls. Among those who died, the median age at death was significantly lower in the LD group (64.1 years, IQR 50.3–76.9 vs 75.9 years, IQR 63.7–85.0). Overall, LD was associated with over a threefold increase in all-cause mortality risk (aHR 3.19, 3.12–3.27). Individuals on the LD register also had higher risk (aHR 3.13, 3.03–3.23). The risk of mortality increased by LD severity: mild (aHR 1.53, 1.40–1.68), moderate (aHR 1.76, 1.63–1.89), and severe (aHR 3.16, 2.95–3.39). Those with Down syndrome had the highest risk of all-cause mortality (aHR 12.16, 11.37–13.00).

### Cancer diagnoses

**Fig. 1:**
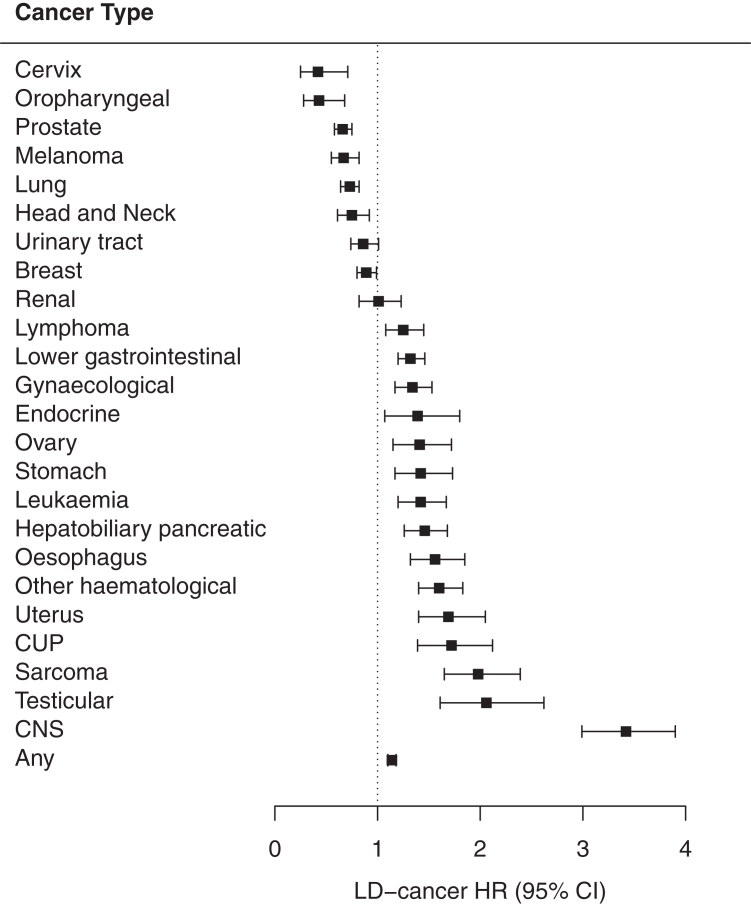
A forest plot of hazard ratios and 95% confidence intervals for the associations between learning disabilty and incident cancer.

Cancer was diagnosed in 3396 (1.9%) individuals with a LD and 67,506 (2.0%) matched controls (aHR 1.14, 1.10–1.18). LD was associated with increased risks of LGI (aHR 1.32, 1.20–1.46), HPB (aHR 1.46, 1.26–1.68), oesophageal (aHR 1.56, 1.32–1.85), gastric (aHR 1.42, 1.17–1.73), ovarian (aHR 1.41, 1.15–1.72), uterine (aHR 1.69, 1.40–2.05), endocrine (aHR 1.39, 1.07–1.80), testicular (aHR 2.06, 1.61–2.62), CUP (aHR 1.72, 1.39–2.12) and CNS (aHR 3.42, 2.99–3.90) cancers, as well as sarcoma (aHR 1.98, 1.65–2.39), leukaemia (aHR 1.42, 1.20–1.67), and lymphoma (aHR 1.25, 1.08–1.45) (Fig. 1). In contrast, LD was associated with reduced risks of breast (aHR 0.89, 0.80–0.99), prostate (aHR 0.66, 0.58–0.75), lung (aHR 0.73, 0.64–0.82), melanoma (aHR 0.67, 0.55–0.82), cervical (aHR 0.42, 0.25–0.71) and head and neck cancers (aHR 0.75, 0.61–0.92), including oropharyngeal cancer (aHR 0.43, 0.28–0.68).

As with the broader LD cohort, individuals on the LD register had higher risks of digestive tract, gynaecological, and haematological cancers, but lower risks of prostate, breast, lung, and urinary tract (ex. renal) cancers (Supplementary Table S1). Across the mild, moderate, and severe LD subgroups, the risk of most cancers declined progressively with increasing LD severity (Supplementary Tables S2–S4). This inverse relationship was particularly pronounced for prostate cancer (mild: aHR 0.63, 0.44–0.90; moderate: aHR 0.56, 0.40–0.77; severe: aHR 0.19, 0.10–0.37) and breast cancer (mild: aHR 0.79, 0.58–1.06; moderate: aHR 0.68, 0.52–0.88; severe: aHR 0.54, 0.38–0.78). Down syndrome was not associated with overall cancer risk (aHR 1.08, 0.94–1.25) (Supplementary Table S5). The risk of breast cancer was markedly reduced in individuals with Down syndrome (aHR 0.14, 0.06–0.32), and fewer than five prostate cancer cases were recorded. By contrast, Down syndrome was strongly associated with increased risk of haematological cancers (aHR 3.76, 2.98–4.74).

In sensitivity analyses (Supplementary Table S6), complete-case analyses and censoring of other cancers produced similar estimates to the main analysis. Fine–Gray models also yielded HRs broadly consistent with cause-specific models, though often smaller, suggesting that competing mortality may have had a greater influence in the LD group. Consistent with this, the LD group showed larger gaps between crude and age-standardised incidence rates, likely reflecting fewer individuals surviving to the older ages when most cancers arise.

The associations of LD with cancer before the age of 50 were similar in direction to the associations with cancer at any age but generally greater in magnitude (aHR of any cancer 1.74, 1.63–1.86). The associations were strongest for CNS (aHR 5.87, 4.96–6.96), uterine (aHR 2.24, 1.30–3.88), ovarian (aHR 1.73, 1.19–2.52) and digestive tract cancers (aHR 2.54, 2.12–3.03), especially oesophageal (aHR 5.69, 3.70–8.76) and HPB cancers (aHR 2.57, 1.71–3.88). LD was inversely associated with the risks of breast cancer and melanoma before the age of 50 years (Table 2).

**Table 2:** Cancer diagnoses among patients with a learning disability compared to matched controls.

Cancer type	n	Person years	n cancer (%)	aHR (95% CI) before 50 years	aHR (95% CI)	Median age at diagnosis (years with IQR)
**Any cancer**						
LD	180,911	1,072,023	3396 (1.9%)	1.74 (1.63–1.86)	1.14 (1.10–1.18)	61.2 (48.3–71.9)
Matched controls	3,405,467	20,813,230	67,506 (2.0%)			66.6 (56.3–75.9)
**Digestive tract**						
LD	180,911	1,079,725	855 (0.5%)	2.54 (2.12–3.03)	1.43 (1.33–1.53)	64.6 (54.9–73.4)
Matched controls	3,405,467	20,994,632	14,199 (0.4%)			69.9 (60.9–78.7)
**LGI**						
LD	180,911	1,080,205	432 (0.2%)	2.12 (1.66–2.71)	1.32 (1.20–1.46)	65.2 (55.6–74.8)
Matched controls	3,405,467	21,002,675	7753 (0.2%)			69.8 (60.7–78.6)
**Oesophageal**						
LD	180,911	1,080,861	138 (0.1%)	5.69 (3.70–8.76)	1.56 (1.32–1.85)	61.7 (52.1–69.6)
Matched controls	3,405,467	21,022,871	2037 (0.1%)			68.9 (60.5–77.3)
**Stomach**						
LD	180,911	1,080,840	103 (0.1%)	2.11 (1.17–3.81)	1.42 (1.17–1.73)	65.6 (59.1–76.4)
Matched controls	3,405,467	21,023,291	1671 (0.0%)			70.9 (61.0–79.1)
**HPB**						
LD	180,911	1,080,816	199 (0.1%)	2.57 (1.71–3.88)	1.46 (1.26–1.68)	65.7 (55.9–73.5)
Matched controls	3,405,467	21,022,698	3356 (0.1%)			70.4 (61.9–79.1)
**Breast**						
LD	68,670	400,354	373 (0.5%)	0.74 (0.58–0.94)	0.89 (0.80–0.99)	59.6 (52.0–69.8)
Matched controls	1,296,112	7,946,844	9533 (0.7%)			60.0 (51.3–69.7)
**Prostate**						
LD	112,241	678,415	248 (0.2%)	0.40 (0.10–1.65)	0.66 (0.58–0.75)	70.4 (63.5–77.6)
Matched controls	2,109,355	13,001,127	9403 (0.4%)			70.6 (64.0–77.2)
**Lung**						
LD	180,911	1,080,773	269 (0.1%)	0.98 (0.61–1.58)	0.73 (0.64–0.82)	69.9 (61.4–76.7)
Matched controls	3,405,467	21,016,662	8281 (0.2%)			70.7 (63.3–78.3)
**Haematological cancer**						
LD	180,911	1,079,249	525 (0.3%)	2.13 (1.86–2.45)	1.41 (1.29–1.54)	55.1 (33.4–68.9)
Matched controls	3,405,467	20,997,307	8430 (0.2%)			65.6 (51.3–76.1)
**Leukaemia**						
LD	180,911	1,080,439	158 (0.1%)	2.06 (1.61–2.62)	1.42 (1.20–1.67)	54.1 (10.6–67.7)
Matched controls	3,405,467	21,017,577	2523 (0.1%)			64.9 (47.7–75.8)
**Lymphoma**						
LD	180,911	1,080,458	188 (0.1%)	1.65 (1.31–2.08)	1.25 (1.08–1.45)	56.0 (33.9–67.8)
Matched controls	3,405,467	21,014,776	3254 (0.1%)			64.1 (49.2–74.9)
**Other haematological**						
LD	180,911	1,080,180	230 (0.1%)	2.99 (2.38–3.75)	1.60 (1.40–1.83)	57.3 (45.6–71.6)
Matched controls	3,405,467	21,014,548	3458 (0.1%)			68.1 (56.0–77.3)
**Gynae**						
LD	68,670	401,305	233 (0.3%)	1.15 (0.87–1.51)	1.34 (1.17–1.53)	58.6 (51.5–67.8)
Matched controls	1,296,112	7,976,122	3901 (0.3%)			61.9 (51.4–72.1)
**Ovary**						
LD	68,670	401,675	101 (0.1%)	1.73 (1.19–2.52)	1.41 (1.15–1.72)	57.7 (46.7–67.8)
Matched controls	1,296,112	7,985,304	1572 (0.1%)			62.7 (52.0–73.0)
**Uterine**						
LD	68,670	401,498	113 (0.2%)	2.24 (1.30–3.88)	1.69 (1.40–2.05)	60.1 (53.1–69.7)
Matched controls	1,296,112	7,983,723	1585 (0.1%)			64.6 (57.6–72.7)
**Cervical**						
LD	68,670	401,801	14 (0.0%)	0.14 (0.05–0.45)	0.42 (0.25–0.71)	57.8 (50.7–65.8)
Matched controls	1,296,112	7,987,132	731 (0.1%)			45.9 (32.9–60.5)
**Renal**						
LD	180,911	1,080,725	99 (0.1%)	1.30 (0.83–2.04)	1.01 (0.82–1.23)	62.4 (53.7–70.2)
Matched controls	3,405,467	21,019,890	2186 (0.1%)			67.7 (57.9–76.4)
**Urinary tract**						
LD	180,911	1,080,607	158 (0.1%)	1.66 (1.06–2.59)	0.86 (0.74–1.01)	65.8 (57.0–76.5)
Matched controls	3,405,467	21,010,979	4352 (0.1%)			71.5 (62.6–79.0)
**CNS**						
LD	180,911	1,080,060	255 (0.1%)	5.87 (4.96–6.96)	3.42 (2.99–3.90)	21.2 (8.8–52.7)
Matched controls	3,405,467	21,022,402	1661 (0.0%)			57.5 (32.1–69.2)
**Melanoma**						
LD	180,911	1,080,664	105 (0.1%)	0.53 (0.36–0.77)	0.67 (0.55–0.82)	60.3 (49.7–71.4)
Matched controls	3,405,467	21,011,161	3485 (0.1%)			60.9 (48.3–72.0)
**Sarcoma**						
LD	180,911	1,080,502	128 (0.1%)	2.63 (2.05–3.39)	1.98 (1.65–2.39)	46.5 (23.7–61.6)
Matched controls	3,405,467	21,021,095	1415 (0.0%)			58.2 (38.8–70.4)
**Head and Neck**						
LD	180,911	1,080,734	91 (0.1%)	1.08 (0.72–1.62)	0.75 (0.61–0.92)	61.1 (47.4–71.9)
Matched controls	3,405,467	21,017,142	2620 (0.1%)			62.1 (54.2–70.3)
**Oropharyngeal**						
LD	180,911	1,080,956	19 (0.0%)	0.66 (0.24–1.81)	0.43 (0.28–0.68)	62.9 (56.8–74.5)
Matched controls	3,405,467	21,023,288	895 (0.0%)			60.4 (54.5–66.9)
**Endocrine**						
LD	180,911	1,080,774	60 (0.0%)	1.29 (0.90–1.85)	1.39 (1.07–1.80)	49.3 (29.8–61.0)
Matched controls	3,405,467	21,022,134	979 (0.0%)			51.7 (38.5–65.2)
**Testicular**						
LD	112,241	678,818	73 (0.1%)	2.19 (1.70–2.84)	2.06 (1.61–2.62)	31.7 (26.7–42.9)
Matched controls	2,109,355	13,032,870	651 (0.0%)			33.8 (25.6–46.2)
**Cancer of unknown primary**						
LD	180,911	1,079,931	93 (0.1%)	2.88 (1.57–5.30)	1.72 (1.39–2.12)	67.6 (54.8–77.7)
Matched controls	3,405,467	20,995,109	1303 (0.0%)			70.8 (62.2–80.0)

Abbreviations: n, number of individuals; CI, confidence interval; IQR, interquartile range; LD, learning disability; LGI, lower gastrointestinal; OG, oesophago-gastric; HPB, hepatopancreaticobiliary; CNS, central nervous system; AS, age-standardised; aHR, age and sex adjusted hazard ratio.

### Symptoms, urgent suspected cancer referrals, and stage at diagnosis

Red-flag symptoms were more commonly recorded in individuals with a LD compared to those without (n = 21,948 [12.1%] vs 221,404 [6.5%] with ≥1 symptom; aIRR 1.81, 1.77–1.86). The burden of symptoms was highest in individuals with a severe LD (n = 3431 [23.0%] with ≥1 symptom; aIRR 2.41, 2.25–2.58). A greater proportion of individuals with a LD had at least one urgent suspected cancer referral compared to those without (n = 6851 [3.8%] vs 99,807 [2.9%]; IRR 1.04, 1.01-1.07). However, the incidence of referrals was lower for individuals with a moderate or severe LD or Down syndrome compared to no LD (Table 3). Individuals with a LD were markedly less likely to be referred within 28 days of a new red-flag symptom (aRR 0.52, 0.49–0.55), with the lowest rates in those with a severe LD (aRR 0.23, 0.20–0.28) and Down syndrome (aRR 0.27, 0.22–0.34).

**Table 3:** Red-flag symptoms and urgent suspected cancer referrals in individuals with a learning disability compared to matched controls.

	Matched controls	Learning disability	Estimate
**Red-flag symptoms**	**n with symptoms/total**	**n with symptoms/total**	**aIRR (95% CI)**
Any LD	221,404/3,405,467 (6.5%)	21,948/180,911 (12.1%)	1.81 (1.77–1.86)
LD register	116,478/1,272,987 (9.1%)	12,517/68,098 (18.4%)	2.05 (1.98–2.13)
Mild LD	27,472/295,506 (9.3%)	2935/15,818 (18.6%)	1.94 (1.79–2.12)
Moderate LD	36,648/366,124 (10.0%)	3853/19,580 (19.7%)	1.98 (1.84–2.13)
Severe LD	27,427/279,935 (9.8%)	3431/14,946 (23.0%)	2.41 (2.25–2.58)
Down syndrome	16,263/199,543 (8.2%)	1762/10,634 (16.6%)	2.03 (1.83–2.25)
**USC referrals**	**n referred/total**	**n referred/total**	**aIRR (95% CI)**
Any LD	99,807/3,405,467 (2.9%)	6851/180,911 (3.8%)	1.04 (1.01-1.07)
LD register	57,554/1,272,987 (4.5%)	3507/68,098 (5.1%)	0.89 (0.86–0.93)
Mild LD	14,894/295,506 (5.0%)	981/15,818 (6.2%)	0.99 (0.92–1.07)
Moderate LD	19,171/366,124 (5.2%)	1173/19,580 (6.0%)	0.89 (0.83–0.95)
Severe LD	13,555/279,935 (4.8%)	718/14,946 (4.8%)	0.76 (0.70–0.83)
Down syndrome	6679/199,543 (3.3%)	353/10,634 (3.3%)	0.78 (0.70–0.88)
**USC referral within 28 days of symptomatic episode**	**n referred within 28 days/total symptomatic episodes**	**n referred within 28 days/total symptomatic episodes**	**aRR (95% CI)**
Any LD	41,921/452,913 (9.3%)	1841/38,487 (4.8%)	0.52 (0.49–0.55)
LD register	23,498/221,578 (10.6%)	924/23,931 (3.9%)	0.36 (0.34–0.39)
Mild LD	5274/45,466 (11.6%)	281/5080 (5.5%)	0.49 (0.43–0.55)
Moderate LD	6733/61,213 (11.0%)	300/7279 (4.1%)	0.38 (0.33–0.42)
Severe LD	4763/47,409 (10.0%)	161/6766 (2.4%)	0.23 (0.20–0.28)
Down syndrome	3902/38,543 (10.1%)	89/3265 (2.7%)	0.27 (0.22–0.34)

Abbreviations: LD, learning disability; USC, urgent suspected cancer; aIRR, adjusted incidence rate ratio; aRR, adjusted risk ratio; CI, confidence interval; n, number.

Among individuals with cancer, those with a LD were significantly less likely to have received an urgent suspected cancer referral in the year prior to diagnosis compared to those without a LD (n = 701 [20.6%] vs 16,014 [27.1%]; aRR 0.81, 0.76–0.86) (Supplementary Table S7). This disparity was consistent across multiple cancer types, including digestive tract, haematological, urinary tract (ex. renal) and renal cancers, and lymphoma. Lower referral rates were also seen among patients on the LD register, and those with a moderate or severe LD. Patients with Down syndrome were least likely to have been referred (aRR 0.53, 0.35–0.79) (Supplementary Table S7).

Compared to controls, individuals with a LD were more likely to be diagnosed with stage 4 cancer relative to stages 1–3 (aRR 1.12, 1.02–1.22) or with unstaged cancer (aRR 1.30, 1.24–1.37) (Supplementary Table S8). Associations with stage 4 and/or unstaged cancer were observed for digestive tract, breast, and gynaecological cancers, among others. Individuals on the LD register and those with severe LD or Down syndrome were also more likely to have missing staging information (Supplementary Table S9).

### Treatment rates and overall survival

Fewer individuals with a LD received treatment within six months of cancer diagnosis compared to those without a LD (n = 1243 [66.5%] vs 27,609 [79.7%]; aRR 0.83, 0.80–0.85; Table 4). The proportions treated decreased with the severity of LD, with those with Down syndrome being the least likely to receive treatment within six months (aRR 0.71, 0.61–0.84). Individuals with a LD were less likely to be treated for most cancer types, including digestive tract cancers, uterine and ovarian cancers. The largest difference in treatment rates was seen for those with cancer of unknown primary, with just 5 (6.6%) of those with a LD receiving treatment within six months (aRR 0.14, 0.06–0.34).

**Table 4:** Treatment within six months of diagnosis of cancer in individuals with and without a learning disability.

	No learning disability	Learning disability	aRR (95% CI)
**Learning disability severity**	**Treated within six months/total diagnoses**	**Treated within six months/total diagnoses**	
Any LD	27,609/34,637 (79.7%)	1243/1869 (66.5%)	0.83 (0.80–0.85)
LD register	16,112/19,770 (81.5%)	575/892 (64.5%)	0.77 (0.74–0.81)
Mild LD	3444/4213 (81.7%)	153/205 (74.6%)	0.88 (0.81–0.95)
Moderate LD	4757/5795 (82.1%)	170/247 (68.8%)	0.82 (0.76–0.89)
Severe LD	3468/4135 (83.9%)	88/136 (64.7%)	0.75 (0.67–0.84)
Downs syndrome	2822/3325 (84.9%)	50/84 (59.5%)	0.71 (0.61–0.84)
**Cancer type**			
Digestive tract	5682/7190 (79.0%)	326/512 (63.7%)	0.78 (0.73–0.83)
LGI	3426/3942 (86.9%)	199/278 (71.6%)	0.80 (0.74–0.87)
Oesophageal	750/852 (88.0%)	49/77 (63.6%)	0.69 (0.58–0.82)
Stomach	442/585 (75.6%)	15/32 (46.9%)	0.61 (0.42–0.89)
HPB	961/1663 (57.8%)	52/108 (48.1%)	0.80 (0.65–0.97)
Breast	4805/4963 (96.8%)	198/214 (92.5%)	0.95 (0.92–0.99)
Prostate	3862/4999 (77.3%)	105/139 (75.5%)	0.97 (0.88–1.07)
Lung	2564/4136 (62.0%)	62/150 (41.3%)	0.63 (0.52–0.76)
Haematological cancer	2423/3561 (68.0%)	147/211 (69.7%)	0.94 (0.87–1.03)
Leukaemia	586/1015 (57.7%)	36/62 (58.1%)	0.77 (0.65–0.92)
Lymphoma	1269/1616 (78.5%)	83/96 (86.5%)	1.04 (0.96–1.13)
Other haematological	568/930 (61.1%)	28/53 (52.8%)	0.88 (0.68–1.15)
Gynae	1761/1941 (90.7%)	106/139 (76.3%)	0.84 (0.77–0.92)
Ovary	531/610 (87.0%)	35/47 (74.5%)	0.84 (0.72–0.99)
Uterine	736/801 (91.9%)	57/70 (81.4%)	0.88 (0.79–0.98)
Cervical	333/352 (94.6%)	6/8 (75.0%)	0.89 (0.61–1.29)
Renal	811/1113 (72.9%)	44/72 (61.1%)	0.76 (0.63–0.91)
Urinary tract	899/1013 (88.7%)	29/50 (58.0%)	0.64 (0.50–0.81)
CNS	481/627 (76.7%)	43/74 (58.1%)	0.66 (0.54–0.80)
Melanoma	1499/1526 (98.2%)	52/55 (94.5%)	0.97 (0.91–1.03)
Sarcoma	264/299 (88.3%)	16/24 (66.7%)	0.76 (0.57–1.01)
Head and Neck	1137/1302 (87.3%)	25/36 (69.4%)	0.79 (0.64–0.97)
Endocrine	430/463 (92.9%)	19/24 (79.2%)	0.85 (0.69–1.03)
Testicular	363/366 (99.2%)	48/49 (98.0%)	0.98 (0.94–1.02)
Cancer of unknown primary	188/489 (38.4%)	5/76 (6.6%)	0.14 (0.06–0.34)

Abbreviations: aRR, adjusted relative risk; LGI, lower gastrointestinal; HPB, hepatopancreaticobiliary; CNS, central nervous system.

**Fig. 2:**
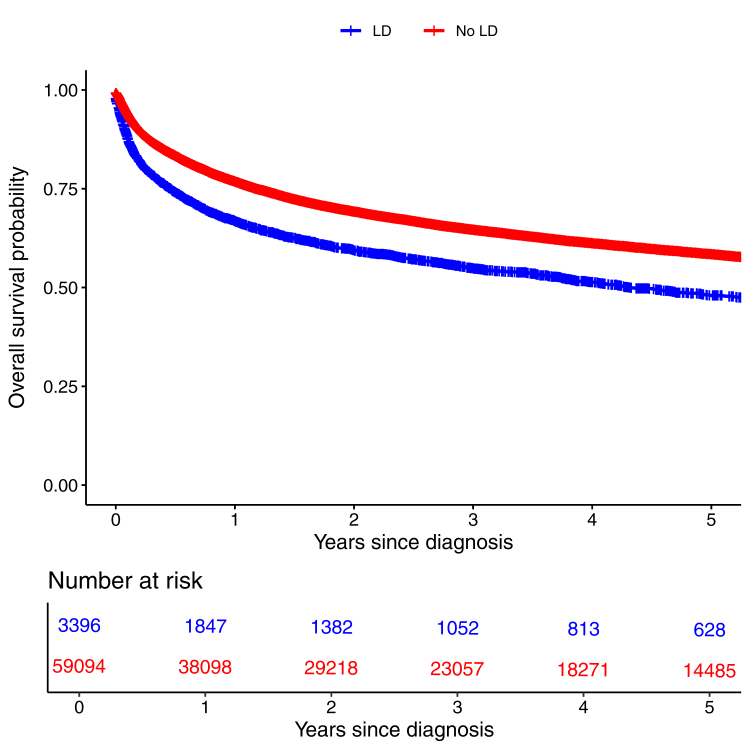
Overall survival following a cancer diagnosis among individuals with and matched comparitors.

Individuals with a LD also had worse OS following a cancer diagnosis. For all cancers, median OS was 4.4 years (3.9–5.1) among patients with a LD vs 9.1 years (8.8–9.5) for those without (Fig. 2), with 1-year survival of 66.8% (65.2–68.5) vs 76.9% (76.5–77.2) and 5-year survival of 48.1% (46.1–50.2) vs 58.5% (58.1–59.0). The aHR for OS in individuals with a LD was 1.73, 1.65–1.83. This survival disadvantage was observed across most cancer types (Supplementary Figures S1–S26), including melanoma (aHR 2.26, 1.54–3.31), lymphoma (aHR 2.25, 1.80–2.82), breast (aHR 1.82, 1.48–2.23) ovarian (aHR 2.27, 1.74–2.98) and uterine cancers (aHR 1.74, 1.22–2.49). Adjustment frequently increased the strength of these associations, reflecting the younger age of diagnosis in the LD population yet worse survival. The only cancer where patients with a LD experienced longer survival was CNS cancer, but this advantage was not significant after adjustment (Table 5).

**Table 5:** Overall survival following cancer diagnoses among patients with and without a learning disability.

Cancer type	Median OS (years with 95% CI)	1-year OS (% with 95% CI)	5-year OS (% with 95% CI)	HR OS (95% CI)	aHR OS (95% CI)
**Any cancer**					
LD	4.35 (3.86–5.14)	66.8% (65.2–68.5)	48.1% (46.1–50.2)	1.42 (1.35–1.50)	1.73 (1.65–1.83)
Matched controls	9.14 (8.78–9.50)	76.9% (76.5–77.2)	58.5% (58.1–59.0)		
**Digestive tract**					
LD	0.74 (0.61–0.97)	46.0% (42.6–49.7)	23.0% (19.7–26.8)	1.53 (1.41–1.67)	1.70 (1.56–1.86)
Matched controls	1.80 (1.69–1.94)	60.0% (59.1–60.9)	35.9% (34.9–36.9)		
**LGI**					
LD	1.70 (1.26–2.45)	58.8% (54.1–63.9)	33.8% (28.5–40.0)	1.89 (1.65–2.16)	2.09 (1.83–2.38)
Matched controls	6.12 (5.52–6.82)	76.7% (75.6–77.7)	53.0% (51.6–54.4)		
**Oesophageal**					
LD	0.58 (0.47–0.77)	37.1% (29.5–46.7)	7.4% (3.5–15.9)	1.48 (1.22–1.79)	1.60 (1.31–1.95)
Matched controls	0.95 (0.87–1.04)	48.5% (46.1–51.0)	16.7% (14.6–19.0)		
**Stomach**					
LD	0.62 (0.32–1.13)	41.1% (32.2–52.3)	14.3% (8.5–24.2)	1.21 (0.96–1.51)	1.30 (1.04–1.63)
Matched controls	0.87 (0.80–1.00)	47.3% (44.8–50.1)	18.6% (16.4–21.2)		
**HPB**					
LD	0.22 (0.18–0.33)	24.7% (18.9–32.3)	11.8% (7.3–19.1)	1.18 (1.00–1.39)	1.33 (1.13–1.57)
Matched controls	0.43 (0.38–0.47)	31.2% (29.5–33.0)	10.1% (8.8–11.6)		
**Breast**					
LD	11.07 (9.92–a)	90.6% (87.6–93.7)	72.9% (67.6–78.6)	1.95 (1.59–2.40)	1.82 (1.48–2.23)
Matched controls	a(a–a)	95.6% (95.2–96.1)	84.4% (83.4–85.3)		
**Prostate**					
LD	8.51 (6.12–a)	86.5% (82.2–91.1)	60.2% (52.2–69.5)	1.62 (1.28–2.06)	1.57 (1.24–2.00)
Matched controls	12.84 (11.81–15.18)	93.6% (93.0–94.1)	75.6% (74.5–76.8)		
**Lung**					
LD	0.34 (0.26–0.46)	30.9% (25.4–37.6)	10.2% (5.7–18.3)	1.20 (1.04–1.38)	1.21 (1.05–1.40)
Matched controls	0.58 (0.56–0.61)	38.5% (37.4–39.7)	13.5% (12.5–14.6)		
**Haematological cancer**					
LD	7.61 (5.44–9.54)	78.7% (75.2–82.4)	57.0% (52.1–62.4)	1.22 (1.05–1.40)	1.75 (1.52–2.03)
Matched controls	9.82 (9.11–10.72)	82.7% (81.8–83.6)	64.0% (62.7–65.3)		
**Leukaemia**					
LD	8.34 (5.55–a)	77.1% (70.7–84.1)	60.3% (52.3–69.6)	1.01 (0.78–1.31)	1.60 (1.23–2.08)
Matched controls	7.97 (7.05–9.70)	77.7% (75.9–79.5)	59.0% (56.6–61.5)		
**Lymphoma**					
LD	3.83 (2.65–a)	69.4% (62.9–76.6)	45.6% (37.5–55.4)	1.63 (1.31–2.04)	2.25 (1.80–2.82)
Matched controls	11.22 (10.21–a)	80.9% (79.4–82.4)	65.5% (63.5–67.6)		
**Other haematological**					
LD	8.07 (5.78–a)	86.4% (81.9–91.0)	62.2% (54.9–70.6)	0.97 (0.77–1.22)	1.44 (1.14–1.82)
Matched controls	7.93 (7.27–9.14)	84.6% (83.3–85.9)	61.8% (59.7–64.0)		
**Gynae**					
LD	4.68 (2.03–6.86)	68.5% (62.6–74.9)	47.9% (40.6–56.5)	1.87 (1.53–2.29)	1.97 (1.61–2.41)
Matched controls	14.26 (13.67–a)	83.2% (81.9–84.5)	63.1% (61.2–65.0)		
**Ovary**					
LD	1.24 (0.58–2.96)	56.2% (47.1–67.0)	30.8% (20.9–45.2)	2.03 (1.55–2.64)	2.27 (1.74–2.98)
Matched controls	4.65 (4.00–5.66)	74.7% (72.4–77.1)	48.9% (45.9–52.1)		
**Uterine**					
LD	11.31 (6.04–a)	80.1% (72.8–88.1)	65.2% (55.0–77.3)	1.44 (1.01–2.05)	1.74 (1.22–2.49)
Matched controls	a(14.24–a)	88.5% (86.8–90.2)	71.6% (68.9–74.4)		
**Cervical**					
LD	1.38 (1.10–a)	71.4% (51.3–99.5)	38.1% (18.6–78.1)	2.73 (1.34–5.55)	1.61 (0.78–3.33)
Matched controls	a(a–a)	86.9% (84.2–89.6)	71.6% (67.7–75.6)		
**Renal**					
LD	3.84 (2.14–a)	69.1% (60.2–79.2)	43.3% (32.7–57.3)	1.27 (0.95–1.71)	1.52 (1.13–2.04)
Matched controls	6.23 (5.72–7.91)	74.7% (72.7–76.8)	54.6% (52.0–57.5)		
**Urinary tract**					
LD	3.61 (1.97–5.14)	66.8% (59.5–75.1)	41.7% (33.1–52.5)	1.96 (1.56–2.48)	2.16 (1.71–2.74)
Matched controls	10.25 (9.37–11.35)	83.7% (82.5–84.9)	64.8% (63.0–66.6)		
**CNS**					
LD	a(a–a)	74.3% (68.8–80.2)	69.6% (63.6–76.1)	0.42 (0.33–0.53)	0.82 (0.64–1.06)
Matched controls	1.54 (1.39–1.75)	59.7% (57.1–62.5)	34.2% (31.3–37.4)		
**Melanoma**					
LD	10.91 (8.29–a)	87.2% (80.6–94.3)	68.4% (58.0–80.6)	2.31 (1.58–3.39)	2.26 (1.54–3.31)
Matched controls	a(a–a)	95.1% (94.3–95.9)	83.8% (82.3–85.4)		
**Sarcoma**					
LD	a(8.28–a)	81.0% (74.2–88.3)	67.9% (59.4–77.7)	0.86 (0.62–1.18)	0.94 (0.68–1.31)
Matched controls	12.86 (9.56–a)	81.4% (79.1–83.7)	59.5% (56.3–62.9)		
**Head and Neck**					
LD	a(2.00–a)	72.4% (63.4–82.8)	53.8% (43.3–66.8)	1.31 (0.94–1.83)	1.47 (1.05–2.06)
Matched controls	9.61 (8.16–11.61)	81.5% (79.9–83.2)	60.6% (58.2–63.0)		
**Oropharyngeal**					
LD	1.33 (0.33–a)	52.3% (32.7–83.7)	44.8% (25.6–78.4)	1.83 (0.94–3.56)	1.36 (0.69–2.70)
Matched controls	6.73 (6.09–10.25)	80.3% (77.4–83.3)	57.0% (52.7–61.6)		
**Endocrine**					
LD	a(a–a)	89.4% (81.7–97.8)	72.1% (59.4–87.6)	1.02 (0.57–1.84)	1.16 (0.64–2.09)
Matched controls	a(a–a)	86.8% (84.5–89.2)	79.0% (75.9–82.2)		
**Testicular**					
LD	a(a–a)	93.8% (88.0–99.9)	90.0% (82.6–98.0)	1.40 (0.63–3.12)	2.50 (1.08–5.81)
**Matched controls**	a(a–a)	95.9% (94.2–97.6)	92.4% (89.9–94.9)		
CUP					
LD	0.07 (0.04–0.13)	14.2% (8.1–25.0)	7.1% (1.6–31.8)	1.46 (1.15–1.84)	1.61 (1.27–2.04)
Matched controls	0.15 (0.13–0.18)	22.2% (19.7–25.0)	11.0% (8.8–13.8)		

Abbreviations: LD, learning disability; OS, overall survival; HR, hazard ratio; aHR, adjusted hazard ratio; CI, confidence interval; LGI, lower gastrointestinal; OG, oesophago-gastric; HPB, hepatopancreaticobiliary; CNS, central nervous system.

a Not reached.

Similar patterns of shorter OS were observed among individuals on the LD register (Supplementary Table S10). For those with mild LD, OS was closer to that of individuals without a LD (aHR 1.12, 0.92–1.37), with disparities increasing in moderate (aHR 1.21, 1.02–1.4) and severe LD (aHR 1.94, 1.60–2.36) (Supplementary Tables S11–S13). This gradient appeared most pronounced in prostate cancer (mild aHR 1.70, 0.82–3.50; moderate aHR 2.13, 1.18–3.83; severe aHR 3.95, 1.40–11.15) and breast cancer (mild aHR 0.86, 0.31–2.34; moderate aHR 2.00, 1.10–3.63; severe aHR 2.34, 1.14–4.83). Individuals with Down syndrome experienced the shortest OS (median 3.8 years, 2.4–8.0; aHR 3.29, 2.66–4.08, with particularly poor outcomes for digestive tract (aHR 3.84, 2.63–5.60), urinary tract ex. renal (aHR 18.49, 7.63–44.82), haematological (aHR 3.36, 2.18–5.18) and gynaecological cancers (aHR 2.68, 1.08–6.67) (Supplementary Table S14).

## Discussion

In this large, population-based cohort study involving 180,911 individuals with a LD and 3,405,467 matched controls, all-cause mortality, cancer incidence, diagnostic pathways, and survival were examined using nationally representative primary care data linked to cancer registrations, death records, and hospital data in England. Over a median follow-up of 5.0 years (IQR 2.0–9.8), 9622 deaths and 3396 incident cancer cases were observed among individuals with a LD. A greater than three-fold higher risk of all-cause mortality was identified in this population compared to those without a LD, with risk increasing in line with LD severity. Individuals with Down syndrome had over a ten-fold increased risk, highlighting the severe mortality gap associated with some forms of learning disability.

Although overall cancer risk was elevated among individuals with a LD, there was substantial variation by cancer type. Increased risks were observed for LGI, HPB, oesophageal, gastric, ovarian, uterine, endocrine, testicular, and CNS cancers, as well as sarcoma, leukaemia, and lymphoma. These patterns may partly reflect genetic predisposition. For example, individuals with Down syndrome have increased risk of haematological malignancies due to GATA1 mutations on the short arm of X chromosome.^32^ CNS cancers are more common in neurofibromatosis, a known cause of LD, due to NF gene mutations.^33^ Environmental exposures may also contribute. Higher obesity rates in LD may explain elevated risk of digestive and uterine cancers, while lower tobacco and alcohol use may contribute to inverse associations with lung and head and neck cancers.^34^^,^^35^ LD was inversely associated with cervical and oropharyngeal cancers, both strongly linked to HPV. Literature on HPV exposure in LD is limited, and requires further investigation.

Where lower rates of cancers were observed, such as for breast and prostate cancer, it was not possible to determine if this was due to a genuinely lower risk or under-diagnosis. Prostate and breast cancer are frequently detected through screening, and individuals with a LD are known to participate less in screening programmes (prompting policy efforts to improve inclusion).^36^ Prostate cancer screening is initiated by patient request rather than automatic invitation. It is frequently subclinical and would not be diagnosed without screening. The possibility of underdiagnosis in the LD population in the present study was supported by a stronger inverse association with increasing LD severity and the implausibly low number of cancer cases recorded in individuals with Down syndrome (fewer than five cases).

Although absolute cancer incidence was slightly lower in individuals with LD (n = 3396 [1.9%] vs 67,506 [2.0%]), elevated HRs observed in cause-specific Cox models of incident cancer suggested that individuals with a LD have a higher instantaneous risk of developing cancer overall and across several cancer types. This apparent paradox is consistent with the attenuated subdistribution hazard ratios from Fine and Gray models, which account for the high competing risk of death in the learning disability group. Similarly, the overall crude incidence rate was slightly lower in the LD group but the age-standardised rate was higher. If premature and preventable deaths were reduced, the burden of cancer in this population could increase disproportionately. These findings highlight the need for current efforts to reduce premature mortality in people with a LD to be accompanied by effective strategies to improve cancer detection and care in this population.

Another notable finding of the present study was the strong association between LD and cancer before the age of 50 years. This association was strongest for CNS, uterine, ovarian and digestive tract cancers, particularly oesophageal cancer, for which the risk was more than five-fold higher in those with a LD. The reasons for the association with oesophageal cancer are unclear but may be related to earlier onset obesity and poor diet as major risk factors for most digestive cancers.^37^ In addition, gastroesophageal reflux disease, a major risk factor for oesophageal cancer, has also been shown to be highly prevalent in individuals with a LD.^38^

The present study highlights possible missed opportunities for earlier diagnosis of cancer in individuals with a LD, who were half as likely to be referred for urgent suspected cancer investigation following “red-flag” symptoms suggestive of cancer. This was likely a contributing factor to more cancers being diagnosed outside the urgent suspected cancer referral pathway, and more frequently at stage 4 or without staging information. Because stage at diagnosis is critical for determining standard treatment pathways and cure is rarely possible for stage 4 disease, this likely contributed to the observed association between LD and shorter OS.

Down syndrome, in particular, was associated with low rates of urgent suspected cancer referrals following symptom presentation and the lowest proportion of cancers diagnosed via this pathway. This may reflect attribution of symptoms to non-cancer causes or clinician reluctance to make repeated referrals due to the high incidence of symptoms in this group. Diagnosis outside the urgent referral pathway may have contributed to individuals with Down syndrome having the shortest median overall survival post diagnosis. These findings suggest that targeted interventions to improve cancer symptom assessment in this population could enable earlier detection and better outcomes.

Individuals with a LD were less likely to receive anti-cancer treatment within six months of diagnosis, a pattern observed across many cancer types. It should be noted, however, that this analysis was limited in scope, as it did not examine treatment by modality, intent (e.g., curative vs palliative), or stage. It also did not account for co-morbidities and other factors that may mediate the inverse association between LD and treatment. Nonetheless, the findings suggest that individuals with a LD may face barriers to accessing treatment after a cancer diagnosis, and this should be investigated in future work.

Previous studies from the UK and internationally have shown inconsistent findings on cancer incidence among individuals with a LD. Some have been limited by smaller sample sizes, less detailed identification of learning disabilities, particularly in terms of severity, and a lack of data on competing risks, such as premature mortality. A recent study in Scotland,^30^ where LD was identified using census data, suggested approximately 24% lower cancer incidence in adults with a LD compared to the general population, but higher incidence for specific cancers including uterus, ovarian, kidney, and testicular cancers. This contrasts with a recent Swedish cohort study,^39^ which reported a 1.5-fold increase in overall cancer risk, with elevated risks for most individual cancers. However, that study included only 27,956 individuals with a LD, among whom there were just 188 cancer cases. Another UK study by Heslop et al.^40^ based on 1096 deceased adults with a LD, found that cancers of the digestive organs were the most common cause of cancer-related deaths, compared to the general population in which lung cancer is most common. Analyses from the LeDeR programme has shown that 15% of adults with a LD who died from bowel cancer were younger than 50 years at the time of death.^41^ A large nationwide study in the Netherlands also reported a younger average age at diagnosis among people with intellectual disabilities.^42^

Heslop et al.^40^ previously reported that among deceased adults with cancer, patients with a LD were substantially more likely to be diagnosed via emergency presentation and that nearly half of cancer diagnoses occurred at stage IV. These findings align with international evidence: in Canada, individuals with intellectual and developmental disabilities had significantly higher odds of metastatic disease at diagnosis.^43^ Regarding mortality, a study in Ontario^44^ reported a 1.5–2.5-fold increase in cancer-related deaths across several tumour types in individuals with intellectual disabilities. While UK-based studies remain limited, they have similarly documented high short-term mortality following cancer diagnosis in people with a LD, particularly within the first 30 days.^40^ The reasons for poorer outcomes in individuals with a LD remain unclear, but lower participation in national screening programmes may contribute.^29^ Few studies have examined survival outcomes stratified by LD severity. Our findings add new evidence that individuals with severe or profound disabilities experience the poorest cancer survival. Further research is needed to identify and address the mechanisms driving this disparity.

The present study has several strengths. It represents the most comprehensive population-based investigation of cancer in people with a LD to date, drawing on national datasets. The use of a broad diagnostic code list and stratified analyses by LD severity, Down syndrome status, and inclusion on a LD register provides granular insight into variation within the LD population. However, several limitations warrant consideration. Generalisability may be limited. The study included individuals in England, so findings may not apply to other countries. Fewer than 5% were aged 70 or older at baseline, which may have led to under-representation of age-related cancers. Misclassification of LD may have occurred and could have varied by age, for example if recording improved after the introduction of the LD register in 2008. If misclassification were non-differential, it would likely attenuate associations; however, if it was more common in groups with higher or lower cancer risk, it could bias estimates in either direction. To reduce this risk, we used a broad set of diagnostic codes rather than relying solely on the term “learning disability.” We also conducted a subgroup analysis of patients on the LD register, and the results were similar to those of the main analysis. A lack of information regarding cancer screening, diagnostic activity, or cause-specific mortality prevented exploration of mechanisms underlying observed disparities. It was not possible to determine whether lower diagnosis rates for some cancers reflected underdiagnosis or true lower risk. Our analysis used ICD-10 code C80.0 to identify cases of CUP, in line with NHS coding standards.^45^ This was to reduce the risk of misclassifying malignancies of unknown origin as CUP (compared to a broader set of codes e.g., C77–C80), but may also have led to under-ascertainment of CUP cases. In addition, ascertainment bias may have influenced CUP case identification if individuals with a learning disability were less likely to undergo a full diagnostic work-up, with CUP potentially recorded more frequently where cancers were labelled “unknown” without full investigation. Finally, treatment data may also have been incomplete where not captured by NCRAS.

This study highlights critical gaps and persistent uncertainties in cancer care for individuals with a LD that merit further investigation. While people with a LD face higher risk of certain cancers, the underlying drivers (e.g., lifestyle, genetic factors, or their interaction) remain unclear. There are unknown implications of an increasing cancer burden as improvements in care reduce other health inequalities and premature mortality. Underdiagnosis is a concern, especially for cancers such as prostate and breast, particularly in those with a more severe LD. Research is needed to assess how often General Practitioners initiate relevant investigations following common precursor events, such as infections or hospitalisations. Poorer cancer survival may reflect delayed diagnosis, incomplete staging or unequal treatment access. Care pathways may be disrupted by challenges related to consent and best interest decision-making, potentially contributing to higher rates of cancers with unknown primary. Barriers such as lack of staff training, communication challenges and inflexible appointment systems may also contribute to disparities.^46^ Finally, further work is needed to understand what constitutes appropriate reasonable adjustment at each stage of cancer care, from screening and diagnosis through to treatment and follow-up.^47^

In conclusion, this study offers the most comprehensive evidence to date on cancer incidence, diagnosis, and survival among people with a LD. While overall cancer risk is elevated in this population, premature mortality likely masks the true burden, which may rise as life expectancy improves. Risk varies by cancer type, with higher rates for digestive tract, haematological, CNS, gynaecological, and testicular cancers. Low diagnosis rates for some cancers, along with fewer urgent referrals, more advanced or missing stage at diagnosis, and poorer survival, highlight gaps across the cancer care continuum that warrant urgent attention.

## Contributors

Oliver John Kennedy: Conceptualization, Literature Search, Formal analysis, Investigation, Methodology, Writing—original draft, Writing—review & editing. Umesh Chauhan: Conceptualization, Investigation, Methodology, Writing—review & editing. Louise Gorman: Conceptualization, Investigation, Writing—review & editing. Paul Lorigan: Conceptualization, Investigation, Methodology, Writing—review & editing. Supervision. Samuel Merriel: Conceptualization, Investigation, Methodology, Writing—review & editing. Supervision, Project administration. Tjeerd Van Staa: Conceptualization, Investigation, Methodology, Writing—review & editing. Alison Wright: Conceptualization, Data collection & curation, Investigation, Methodology, Writing—review & editing. Darren Mark Ashcroft: Conceptualization, Investigation, Methodology, Writing—review & editing. Supervision, Project administration. Oliver John Kennedy and Alison Wright had full access to and verified the underlying data. Darren Mark Ashcroft had final responsibility for the decision to submit for publication.

## Data sharing statement

Electronic health records are, by definition, considered sensitive data in the UK by the Data Protection Act and cannot be shared via public deposition because of information governance restriction in place to protect patient confidentiality. Access to Clinical Practice Research Datalink (CPRD) data is subject to protocol approval via CPRD's research data governance process. For more information see https://cprd.com/data-access. Linked secondary care data from Hospital Episodes Statistics, mortality data from the Office for National Statistics, and index of multiple deprivation data can also be requested from CPRD.

## Declaration of interests

DA and TVS report receiving support for the present work from National Institute for Health and Care Research (NIHR). PL reports grants or contracts from Pierre Fabre, consulting fees from Regeneron, payment or honoraria for lectures, presentations, speakers bureaus, manuscript writing or educational events from BMS, Pierre Fabre and Merck, and Support for attending meetings and/or travel from Merck.
