# Supplementary material for: Cancer diagnoses, referrals, and survival in people with a learning disability in the UK: a population-based, matched cohort study
Source: Lancet Reg Health Eur. 2025 Nov 14;60:101519. doi: 10.1016/j.lanepe.2025.101519 (PMC12767837; doi:10.1016/j.lanepe.2025.101519)
Supplement: Supplementary Information [file mmc1.pdf]

Cancer diagnoses, referrals, and survival in people with a learning disability in the UK: a population-based, matched cohort study– supplementary tables, figures and code lists

Oliver John Kennedy[1,2], Umesh Chauhan[3], Louise Gorman[4], Paul Lorigan[1,2], Samuel Merriel[5], Tjeerd Van Staa[6], Alison Wright[6,7], Darren Mark Ashcroft[4,6]

1. Division of Cancer Sciences, University of Manchester, Manchester, UK
2. The Christie NHS Foundation Trust, Manchester, UK
3. School of Medicine, University of Lancashire, Lancashire, UK
4. NIHR Greater Manchester Patient Safety Research Collaboration, University of Manchester, Manchester, UK
5. Centre for Primary Care and Health Services Research, University of Manchester, Manchester, UK.
6. Centre for Pharmacoepidemiology and Drug Safety, Division of Pharmacy and Optometry, School of Health Sciences, Faculty of Biology, Medicine and Health, University of Manchester, Manchester, UK.
7. Manchester Academic Health Science Centre, Manchester, UK.

Keywords: learning disability, intellectual disability, cancer

Supplementary table 1. Cancer diagnoses among patients on the learning disability register compared to matched controls.

Cancer type	n	Person years	n cancer (%)	Crude incidence rate per 100,000 person years (95% CI)	AS incidence rate per 100,000 person years (95% CI)	aHR (95% CI)	aSHR (95% CI)	Median age at diagnosis (years with IQR)
**Any cancer**								
LD	68,098	458,673	1,564 (2·3%)	341 (324-358)	559 (523-596)	0·90 (0·86-0·95)	0·84 (0·79-0·88)	58.9 (48.9–68.4)
Matched controls	1,272,987	8,043,202	34,912 (2·7%)	434 (430-439)	684 (676-693)			64.4 (54.9–72.8)
**Prostate**								
LD	40,829	276,760	94 (0·2%)	34 (27-42)	86 (63-114)	0·43 (0·35-0·52)	0·38 (0·31-0·47)	67.3 (58.5–72.4)
Matched controls	758,206	4,804,559	4,886 (0·6%)	102 (99-105)	205 (198-213)			68.7 (62.6–74.7)
**Breast**								
LD	27,269	185,010	192 (0·7%)	104 (90-120)	141 (119-167)	0·76 (0·66-0·88)	0·72 (0·62-0·84)	58.1 (52.1–66.7)
Matched controls	514,781	3,308,968	5,242 (1·0%)	158 (154-163)	176 (170-181)			58.2 (50.6–67.4)
**Digestive tract**								
LD	68,098	462,145	424 (0·6%)	92 (83-101)	164 (144-185)	1·26 (1·14-1·38)	1·15 (1·04-1·27)	61.4 (53.1–70.1)
Matched controls	1,272,987	8,139,444	7,056 (0·6%)	87 (85-89)	144 (140-148)			67.0 (58.7–75.0)
**Haematological cancer**								
LD	68,098	461,942	246 (0·4%)	53 (47-60)	70 (60-81)	1·22 (1·07-1·38)	1·12 (0·99-1·28)	52.4 (39.5–63.9)
Matched controls	1,272,987	8,141,336	4,104 (0·3%)	50 (49-52)	77 (74-80)			63.8 (51.8–73.3)
**Lung**								
LD	68,098	462,719	94 (0·1%)	20 (16-25)	41 (32-53)	0·43 (0·35-0·53)	0·40 (0·32-0·49)	66.7 (59.0–74.7)
Matched controls	1,272,987	8,150,576	4,227 (0·3%)	52 (50-53)	89 (86-92)			68.6 (61.9–75.6)
**Gynae**								
LD	27,269	185,475	138 (0·5%)	74 (63-88)	102 (82-126)	1·33 (1·12-1·57)	1·24 (1·04-1·49)	57.3 (51.2–65.3)
Matched controls	514,781	3,325,336	2,065 (0·4%)	62 (59-65)	72 (69-76)			59.9 (50.3–69.3)
**Urinary tract**								
LD	68,098	462,666	67 (0·1%)	14 (11-18)	27 (19-37)	0·63 (0·50-0·81)	0·57 (0·44-0·73)	62.0 (54.8–70.4)
Matched controls	1,272,987	8,147,639	2,209 (0·2%)	27 (26-28)	47 (45-49)			68.7 (60.6–75.9)

Abbreviations: n (number of individuals); LD (learning disability); AS (age-standardised); CI (confidence interval); aHR (adjusted hazard ratio); aSHR (adjusted subdistribution hazard ratio)

Supplementary table 2. Cancer diagnoses among individuals with a mild learning disability compared to matched controls.

Cancer type	n	Person years	n cancer (%)	Crude incidence rate per 100,000 person years (95% CI)	AS incidence rate per 100,000 person years (95% CI)	aHR (95% CI)	aSHR (95% CI)	Median age at diagnosis (years)
**Any cancer**								
LD	15,818	106,029	339 (2·1%)	320 (287-356)	535 (455-625)	0·87 (0·78-0·97)	0·85 (0·76-0·95)	58.3 (47.5–67.2)
Matched controls	295,506	1,677,579	6,535 (2·2%)	390 (380-399)	667 (646-689)			62.0 (53.0–70.2)
**Prostate**								
LD	9,384	63,950	30 (0·3%)	47 (32-67)	137 (61-263)	0·63 (0·44-0·90)	0·61 (0·42-0·88)	64.4 (58.4–70.3)
Matched controls	173,977	997,574	880 (0·5%)	88 (82-94)	197 (179-216)			67.0 (61.0–72.9)
**Breast**								
LD	6,434	42,891	44 (0·7%)	103 (75-138)	146 (91-222)	0·79 (0·58-1·06)	0·78 (0·57-1·06)	53.3 (47.5–64.6)
Matched controls	121,529	692,903	988 (0·8%)	143 (134-152)	161 (149-174)			56.2 (49.5–64.5)
**Digestive tract**								
LD	15,818	106,950	79 (0·5%)	74 (58-92)	135 (96-185)	1·03 (0·82-1·30)	1·01 (0·80-1·28)	59.7 (51.6–67.0)
Matched controls	295,506	1,694,720	1,298 (0·4%)	77 (72-81)	146 (135-156)			64.7 (56.9–72.4)
**Haematological cancer**								
LD	15,818	106,919	55 (0·3%)	51 (39-67)	67 (41-102)	1·27 (0·97-1·67)	1·23 (0·93-1·64)	51.7 (44.5–62.4)
Matched controls	295,506	1,695,228	748 (0·3%)	44 (41-47)	74 (67-81)			60.0 (48.6–69.7)
**Lung**								
LD	15,818	107,098	21 (0·1%)	20 (12-30)	51 (28-86)	0·46 (0·30-0·71)	0·44 (0·29-0·69)	70.2 (58.9–75.3)
Matched controls	295,506	1,696,709	750 (0·3%)	44 (41-47)	87 (80-96)			66.8 (59.8–74.0)
**Gynae**								
LD	6,434	42,983	31 (0·5%)	72 (49-102)	103 (65-156)	1·24 (0·87-1·78)	1·22 (0·83-1·79)	61.4 (53.5–67.7)
Matched controls	121,529	695,748	390 (0·3%)	56 (51-62)	66 (59-74)			58.5 (49.1–66.6)
**Urinary tract**								
LD	15,818	107,064	16 (0·1%)	15 (9-24)	25 (12-46)	0·67 (0·41-1·10)	0·66 (0·40-1·09)	64.2 (56.9–69.7)
Matched controls	295,506	1,696,152	406 (0·1%)	24 (22-26)	47 (41-53)			67.2 (58.8–73.6)

Abbreviations: n (number of individuals); LD (learning disability); AS (age-standardised); CI (confidence interval); aHR (adjusted hazard ratio); aSHR (adjusted subdistribution hazard ratio)

Supplementary table 3. Cancer diagnoses among individuals with a moderate learning disability compared to matched controls.

Cancer type	n	Person years	n cancer (%)	Crude incidence rate per 100,000 person years (95% CI)	AS incidence rate per 100,000 person years (95% CI)	aHR (95% CI)	aSHR (95% CI)	Median age at diagnosis (years with IQR)
**Any cancer**								
LD	19,580	143,821	441 (2·3%)	307 (279-337)	514 (447-589)	0·82 (0·74-0·90)	0·80 (0·72-0·88)	58.5 (47.7–67.9)
Matched controls	366,124	2,270,237	9,253 (2·5%)	408 (399-416)	676 (657-694)			63.0 (53.5–71.1)
**Prostate**								
LD	11,722	86,948	36 (0·3%)	41 (29-57)	108 (63-171)	0·56 (0·40-0·77)	0·54 (0·39-0·76)	64.4 (57.2–71.3)
Matched controls	217,498	1,357,151	1,238 (0·6%)	91 (86-96)	195 (181-210)			67.8 (62.0–73.6)
**Breast**								
LD	7,858	57,976	56 (0·7%)	97 (73-125)	118 (80-168)	0·68 (0·52-0·88)	0·67 (0·51-0·88)	55.8 (52.2–63.2)
Matched controls	148,626	931,691	1,442 (1·0%)	155 (147-163)	170 (160-181)			56.9 (50.3–66.0)
**Digestive tract**								
LD	19,580	145,074	107 (0·5%)	74 (60-89)	137 (102-180)	1·00 (0·82-1·21)	0·97 (0·80-1·19)	60.3 (55.1–69.1)
Matched controls	366,124	2,295,450	1,851 (0·5%)	81 (77-84)	141 (133-150)			64.9 (57.0–73.0)
**Haematological cancer**								
LD	19,580	144,984	82 (0·4%)	57 (45-70)	91 (65-123)	1·28 (1·03-1·59)	1·23 (0·98-1·55)	54.4 (38.7–67.4)
Matched controls	366,124	2,295,822	1,134 (0·3%)	49 (47-52)	80 (74-86)			62.8 (51.1–71.6)
**Lung**								
LD	19,580	145,249	22 (0·1%)	15 (9-23)	29 (14-53)	0·32 (0·21-0·48)	0·32 (0·21-0·48)	64.3 (57.7–69.7)
Matched controls	366,124	2,298,402	1,069 (0·3%)	47 (44-49)	87 (80-94)			67.2 (60.8–73.8)
**Gynae**								
LD	7,858	58,117	39 (0·5%)	67 (48-92)	101 (62-154)	1·21 (0·88-1·67)	1·20 (0·85-1·68)	58.8 (47.7–64.6)
Matched controls	148,626	936,228	542 (0·4%)	58 (53-63)	71 (63-78)			58.4 (49.6–68.8)
**Urinary tract**								
LD	19,580	145,245	21 (0·1%)	14 (9-22)	29 (14-54)	0·64 (0·42-0·98)	0·63 (0·40-0·97)	61.6 (53.8–70.9)
Matched controls	366,124	2,297,740	562 (0·2%)	24 (22-27)	47 (42-52)			66.5 (59.4–73.8)

Abbreviations: n (number of individuals); LD (learning disability); AS (age-standardised); CI (confidence interval); aHR (adjusted hazard ratio); aSHR (adjusted subdistribution hazard ratio)

Supplementary table 4. Cancer diagnoses among individuals with a severe learning disability compared to matched controls.

Cancer type	n	Person years	n cancer (%)	Crude incidence rate per 100,000 person years (95% CI)	AS incidence rate per 100,000 person years (95% CI)	aHR (95% CI)	aSHR (95% CI)	Median age at diagnosis (years with IQR)
**Any cancer**								
LD	14,946	110,570	254 (1·7%)	230 (202-260)	358 (301-423)	0·65 (0·57-0·74)	0·62 (0·55-0·71)	55.3 (44.7–64.3)
Matched controls	279,935	1,764,935	6,765 (2·4%)	383 (374-393)	656 (637-676)			62.9 (53.4–71.3)
**Prostate**								
LD	8,860	65,051	10 (0·1%)	15 (7-28)	31 (12-66)	0·19 (0·10-0·37)	0·18 (0·09-0·35)	62.4 (59.3–69.7)
Matched controls	165,026	1,032,938	901 (0·5%)	87 (82-93)	199 (182-217)			67.3 (61.4–73.6)
**Breast**								
LD	6,086	46,141	31 (0·5%)	67 (46-95)	101 (61-157)	0·54 (0·38-0·78)	0·52 (0·36-0·75)	56.6 (48.6–65.4)
Matched controls	114,909	745,884	1,078 (0·9%)	145 (136-153)	176 (164-189)			57.7 (50.3–66.8)
**Digestive tract**								
LD	14,946	111,228	71 (0·5%)	64 (50-81)	124 (87-173)	0·92 (0·72-1·16)	0·89 (0·70-1·14)	57.9 (51.1–67.3)
Matched controls	279,935	1,783,544	1,380 (0·5%)	77 (73-82)	143 (134-152)			65.7 (57.1–73.7)
**Haematological cancer**								
LD	14,946	111,147	44 (0·3%)	40 (29-53)	46 (31-65)	1·01 (0·74-1·37)	0·97 (0·71-1·33)	46.3 (28.0–57.2)
Matched controls	279,935	1,783,840	781 (0·3%)	44 (41-47)	72 (66-79)			61.5 (49.2–71.3)
**Lung**								
LD	14,946	111,332	9 (0·1%)	8 (4-15)	17 (6-36)	0·17 (0·09-0·34)	0·16 (0·08-0·33)	66.4 (55.8–71.4)
Matched controls	279,935	1,785,831	774 (0·3%)	43 (40-47)	83 (76-90)			67.6 (60.3–74.2)
**Gynae**								
LD	6,086	46,222	23 (0·4%)	50 (32-75)	52 (32-80)	1·10 (0·73-1·67)	1·01 (0·66-1·57)	52.1 (43.6–57.7)
Matched controls	114,909	749,240	398 (0·3%)	53 (48-59)	68 (61-76)			59.3 (49.9–68.1)
**Urinary tract**								
LD	14,946	111,325	12 (0·1%)	11 (6-19)	17 (7-33)	0·56 (0·32-0·98)	0·51 (0·29-0·92)	57.0 (49.5–65.7)
Matched controls	279,935	1,785,206	401 (0·1%)	22 (20-25)	41 (36-46)			66.3 (58.1–73.6)

Abbreviations: n (number of individuals); LD (learning disability); AS (age-standardised); CI (confidence interval); aHR (adjusted hazard ratio); aSHR (adjusted subdistribution hazard ratio)

Supplementary table 5. Cancer diagnoses among individuals with Down syndrome compared to matched controls.

**Cancer type**	**n**	**Person years**	**n cancer (%)**	**Crude incidence rate per 100,000 person years (95% CI)**	**AS incidence rate per 100,000 person years (95% CI)**	**HR (95% CI)**	**aSHR (95% CI)**	**Median age at diagnosis (years with IQR)**
**Any cancer**								
LD	10,634	65,593	186 (1·7%)	284 (244-327)	579 (267-1092)	1·08 (0·94-1·25)	0·82 (0·71-0·95)	55.3 (44.7–64.3)
Matched controls	199,543	1,384,506	5,387 (2·7%)	389 (379-400)	699 (643-759)			62.9 (53.4–71.3)
**Breast**								
LD	5,057	30,460	6 (0·1%)	20 (7-43)	883 (114-3114)	0·17 (0·08-0·38)	0·14 (0·06-0·32)	62.4 (59.3–69.7)
Matched controls	95,609	660,585	990 (1·0%)	150 (141-160)	197 (161-239)			67.3 (61.4–73.6)
**Digestive tract**								
LD	10,634	66,098	35 (0·3%)	53 (37-74)	74 (38-129)	1·21 (0·87-1·70)	0·88 (0·62-1·24)	56.6 (48.6–65.4)
Matched controls	199,543	1,401,288	1,009 (0·5%)	72 (68-77)	150 (123-182)			57.7 (50.3–66.8)
**Haematological cancer**								
LD	10,634	65,830	79 (0·7%)	120 (95-150)	111 (86-140)	3·76 (2·98-4·74)	2·94 (2·29-3·77)	57.9 (51.1–67.3)
Matched controls	199,543	1,401,511	597 (0·3%)	43 (39-46)	70 (55-87)			65.7 (57.1–73.7)
**Lung**								
LD	10,634	66,134	<5 (<0.01%)	3 (0-11)	21 (1-98)	0·14 (0·03-0·57)	0·09 (0·02-0·37)	46.3 (28.0–57.2)
Matched controls	199,543	1,403,110	581 (0·3%)	41 (38-45)	86 (70-104)			61.5 (49.2–71.3)
**Gynae**								
LD	5,057	30,453	13 (0·3%)	43 (23-73)	40 (20-70)	1·11 (0·65-1·91)	0·88 (0·50-1·54)	66.4 (55.8–71.4)
Matched controls	95,609	663,882	359 (0·4%)	54 (49-60)	74 (51-103)			67.6 (60.3–74.2)
**Urinary tract**								
LD	10,634	66,128	9 (0·1%)	14 (6-26)	15 (6-29)	1·24 (0·64-2·43)	0·82 (0·42-1·60)	52.1 (43.6–57.7)
Matched controls	199,543	1,402,753	287 (0·1%)	20 (18-23)	43 (27-66)			59.3 (49.9–68.1)

Abbreviations: n (number of individuals); LD (learning disability); AS (age-standardised); CI (confidence interval); aHR (adjusted hazard ratio); aSHR (adjusted subdistribution hazard ratio)

Supplementary table 6. Incidence rates and hazard ratios for the association between learning disability and incident cancers as estimated in sensitivity analyses.

Cancer type	Crude incidence rate per 100,000 person years (95% CI)	AS incidence rate per 100,000 person years (95% CI)	aSHR (95% CI)	unadjusted HR (95% CI)	aHR (95% CI) for complete case analysis[1]	aHR (95% CI) with other cancers censored[1]
**Any cancer**						
LD	317 (306-328)	708 (681-736)	1·04 (1·00-1·08)	1·17 (1·13-1·21)	1·13 (1·09-1·17)	1·14 (1·10-1·18)
Matched controls	324 (322-327)	677 (671-682)				
**Digestive tract**						
LD	79 (74-85)	191 (177-205)	1·28 (1·19-1·37)	1·45 (1·36-1·55)	1·41 (1·32-1·51)	1·40 (1·30-1·51)
Matched controls	68 (67-69)	147 (144-149)				
**LGI**						
LD	40 (36-44)	98 (88-109)	1·20 (1·09-1·32)	1·34 (1·22-1·47)	1·30 (1·18-1·44)	1·26 (1·14-1·40)
Matched controls	37 (36-38)	79 (78-81)				
**Oesophagus**						
LD	13 (11-15)	28 (23-34)	1·43 (1·20-1·71)	1·62 (1·36-1·92)	1·55 (1·31-1·84)	1·62 (1·34-1·95)
Matched controls	10 (9-10)	20 (19-21)				
**Gastric**						
LD	10 (8-12)	24 (19-30)	1·32 (1·07-1·62)	1·49 (1·23-1·82)	1·41 (1·15-1·72)	1·74 (1·36-2·22)
Matched controls	8 (8-8)	17 (17-18)				
**HPB**						
LD	18 (16-21)	45 (38-52)	1·32 (1·14-1·53)	1·47 (1·28-1·70)	1·44 (1·25-1·67)	1·45 (1·23-1·72)
Matched controls	16 (15-17)	35 (34-36)				
**Breast**						
LD	93 (84-103)	158 (141-176)	0·85 (0·76-0·94)	0·90 (0·81-1·00)	0·88 (0·79-0·98)	0·87 (0·78-0·97)
Matched controls	120 (118-122)	171 (167-174)				
**Prostate**						
LD	37 (32-41)	138 (119-159)	0·59 (0·52-0·67)	0·65 (0·57-0·73)	0·65 (0·58-0·74)	0·62 (0·54-0·71)
Matched controls	72 (71-74)	204 (199-208)				
**Lung**						
LD	25 (22-28)	68 (59-77)	0·66 (0·58-0·75)	0·78 (0·69-0·88)	0·72 (0·64-0·82)	0·76 (0·66-0·87)
Matched controls	39 (39-40)	86 (85-88)				
**Haematological cancer**						
LD	49 (45-53)	93 (84-103)	1·29 (1·18-1·41)	1·43 (1·31-1·56)	1·40 (1·28-1·53)	1·42 (1·30-1·56)
Matched controls	40 (39-41)	77 (75-79)				
**Leukaemia**						
LD	15 (12-17)	25 (20-30)	1·30 (1·10-1·54)	1·46 (1·24-1·71)	1·41 (1·19-1·66)	1·42 (1·18-1·70)
Matched controls	12 (12-12)	22 (21-23)				
**Lymphoma**						
LD	17 (15-20)	33 (28-39)	1·18 (1·02-1·38)	1·29 (1·11-1·49)	1·22 (1·05-1·41)	1·24 (1·06-1·46)
Matched controls	15 (15-16)	29 (27-30)				
**Other haematological**						
LD	21 (19-24)	44 (38-51)	1·46 (1·27-1·68)	1·59 (1·39-1·81)	1·60 (1·40-1·82)	1·60 (1·39-1·85)
Matched controls	16 (16-17)	34 (33-35)				

**Gynae**						
LD	58 (51-66)	95 (82-109)	1·24 (1·08-1·43)	1·40 (1·23-1·59)	1·32 (1·15-1·50)	1·36 (1·19-1·57)
Matched controls	49 (47-50)	71 (69-74)				
**Ovarian**						
LD	25 (20-31)	40 (32-50)	1·37 (1·11-1·69)	1·47 (1·20-1·79)	1·35 (1·10-1·66)	1·47 (1·16-1·87)
Matched controls	20 (19-21)	29 (27-30)				
**Uterus**						
LD	28 (23-34)	47 (39-57)	1·56 (1·28-1·91)	1·74 (1·45-2·10)	1·68 (1·39-2·04)	1·63 (1·32-2·01)
Matched controls	20 (19-21)	30 (28-31)				
**Cervical**						
LD	3 (2-6)	6 (3-10)	0·52 (0·30-0·88)	0·43 (0·25-0·72)	0·43 (0·25-0·72)	0·35 (0·19-0·66)
Matched controls	9 (9-10)	12 (11-13)				
**Renal**						
LD	9 (7-11)	20 (16-24)	0·93 (0·75-1·14)	1·07 (0·87-1·30)	0·99 (0·80-1·21)	1·03 (0·81-1·30)
Matched controls	10 (10-11)	21 (20-22)				
**Urinary tract**						
LD	15 (12-17)	37 (31-44)	0·79 (0·67-0·92)	0·89 (0·76-1·04)	0·85 (0·72-1·00)	0·81 (0·68-0·98)
Matched controls	21 (20-21)	46 (44-47)				
**CNS**						
LD	24 (21-27)	28 (24-33)	3·28 (2·85-3·78)	3·44 (3·02-3·91)	3·36 (2·94-3·84)	3·63 (3·15-4·17)
Matched controls	8 (8-8)	13 (12-13)				
**Melanoma**						
LD	10 (8-12)	21 (17-26)	0·63 (0·52-0·77)	0·68 (0·56-0·82)	0·67 (0·55-0·81)	0·66 (0·54-0·81)
Matched controls	17 (16-17)	31 (30-32)				
**Sarcoma**						
LD	12 (10-14)	19 (15-23)	1·91 (1·57-2·32)	1·99 (1·66-2·37)	1·98 (1·64-2·38)	2·38 (1·92-2·95)
Matched controls	7 (6-7)	11 (10-12)				
**Head and Neck**						
LD	8 (7-10)	18 (14-23)	0·73 (0·59-0·91)	0·80 (0·65-0·98)	0·73 (0·59-0·90)	0·67 (0·53-0·85)
Matched controls	12 (12-13)	23 (22-23)				
**Oropharyngeal**						
LD	2 (1-3)	4 (2-7)	0·53 (0·33-0·84)	0·49 (0·31-0·76)	0·41 (0·26-0·65)	0·33 (0·17-0·65)
Matched controls	4 (4-5)	7 (7-8)				
**Endocrine**						
LD	6 (4-7)	9 (7-12)	1·41 (1·07-1·85)	1·32 (1·02-1·70)	1·36 (1·05-1·76)	1·33 (0·99-1·78)
Matched controls	5 (4-5)	7 (7-8)				
**Testicular**						
LD	11 (8-14)	15 (11-19)	2·12 (1·63-2·75)	2·13 (1·69-2·69)	1·94 (1·51-2·49)	2·01 (1·56-2·60)
Matched controls	5 (5-5)	7 (7-8)				
**CUP**						
LD	9 (7-11)	23 (18-28)	1.60 (1.28-1.99)	1.80 (1.46-2.21)	1.71 (1.39-2.11)	1.70 (1.22-2.36)
Matched controls	6 (6-7)	14 (13-15)				

Abbreviations: aHR (adjusted hazard ratio); CI (confidence interval); LD (learning disability); LGI (lower gastrointestinal); HPB (hepatopancreaticobiliary); CNS (central nervous system); CUP (cancer of unknown primary)

1. Adjusted for age, gender, ethnicity and deprivation

Supplementary table 7. Urgent referrals for suspected cancer within one year preceding cancer diagnosis by learning disability category and by cancer type.

	LD referred / not referred (n, %)	Non-LD referred / not referred (n, %)	aRR LD-referral (95% CI)
**Any cancer**			
LD register	334 (21·4%) / 1,230 (78·6%)	9,141 (27·9%) / 23,619 (72·1%)	0·80 (0·73-0·89)
Mild LD	78 (23·0%) / 261 (77·0%)	1,832 (28·5%) / 4,593 (71·5%)	0·84 (0·69-1·03)
Moderate LD	93 (21·1%) / 348 (78·9%)	2,545 (28·3%) / 6,445 (71·7%)	0·79 (0·65-0·94)
Severe LD	50 (19·7%) / 204 (80·3%)	1,793 (27·1%) / 4,813 (72·9%)	0·78 (0·61-1·01)
Down syndrome	21 (11·3%) / 165 (88·7%)	1,464 (27·6%) / 3,838 (72·4%)	0·53 (0·35-0·79)
**Cancer type (any LD)**			
Any cancer	701 (20·6%) / 2,695 (79·4%)	16,014 (27·1%) / 43,080 (72·9%)	0·81 (0·76-0·86)
Digestive tract	188 (22·0%) / 667 (78·0%)	3,344 (26·8%) / 9,129 (73·2%)	0·87 (0·76-0·99)
LGI	100 (23·1%) / 332 (76·9%)	1,763 (25·9%) / 5,035 (74·1%)	0·94 (0·79-1·11)
Oesophagus	35 (25·4%) / 103 (74·6%)	647 (36·1%) / 1,143 (63·9%)	0·75 (0·56-1·01)
Gastric	18 (17·5%) / 85 (82·5%)	427 (28·6%) / 1,067 (71·4%)	0·64 (0·42-0·98)
HPB	38 (19·1%) / 161 (80·9%)	707 (24·0%) / 2,238 (76·0%)	0·84 (0·62-1·12)
Breast	96 (25·7%) / 277 (74·3%)	2,269 (26·5%) / 6,284 (73·5%)	0·97 (0·82-1·16)
Prostate	102 (41·1%) / 146 (58·9%)	3,046 (37·4%) / 5,102 (62·6%)	1·11 (0·95-1·29)
Lung	53 (19·7%) / 216 (80·3%)	1,796 (24·6%) / 5,510 (75·4%)	0·80 (0·63-1·03)
Haematological cancer	49 (9·3%) / 476 (90·7%)	1,249 (17·1%) / 6,039 (82·9%)	0·61 (0·47-0·80)
Leukaemia	13 (8·2%) / 145 (91·8%)	256 (11·9%) / 1,893 (88·1%)	0·92 (0·54-1·56)
Lymphoma	30 (16·0%) / 158 (84·0%)	694 (24·5%) / 2,137 (75·5%)	0·69 (0·50-0·97)
Other haematological	15 (6·5%) / 215 (93·5%)	418 (13·8%) / 2,605 (86·2%)	0·56 (0·34-0·93)
Gynae	74 (31·8%) / 159 (68·2%)	1,033 (29·7%) / 2,440 (70·3%)	1·10 (0·90-1·33)
Ovarian	17 (16·8%) / 84 (83·2%)	389 (27·9%) / 1,006 (72·1%)	0·61 (0·39-0·94)
Uterus	41 (36·3%) / 72 (63·7%)	536 (37·9%) / 880 (62·1%)	1·02 (0·79-1·31)
Cervical	5 (35·7%) / 9 (64·3%)	96 (14·8%) / 551 (85·2%)	2·06 (0·95-4·49)
Renal	18 (18·2%) / 81 (81·8%)	569 (29·8%) / 1,340 (70·2%)	0·64 (0·42-0·98)
Urinary tract	38 (24·1%) / 120 (75·9%)	1,412 (37·0%) / 2,401 (63·0%)	0·66 (0·50-0·87)
CNS	7 (2·7%) / 248 (97·3%)	65 (4·5%) / 1,364 (95·5%)	0·99 (0·46-2·13)
Melanoma	37 (35·2%) / 68 (64·8%)	1,192 (38·4%) / 1,915 (61·6%)	0·92 (0·70-1·19)
Sarcoma	17 (13·3%) / 111 (86·7%)	228 (19·3%) / 956 (80·7%)	0·77 (0·48-1·21)
Head and Neck	21 (23·1%) / 70 (76·9%)	688 (29·9%) / 1,615 (70·1%)	0·79 (0·54-1·15)
Oropharyngeal	5 (26·3%) / 14 (73·7%)	304 (40·0%) / 456 (60·0%)	0·63 (0·29-1·34)
Endocrine	14 (23·3%) / 46 (76·7%)	177 (20·9%) / 670 (79·1%)	1·20 (0·74-1·94)
Testicular	19 (26·0%) / 54 (74·0%)	175 (31·3%) / 384 (68·7%)	0·82 (0·55-1·22)
CUP	21 (22.6%) / 72 (77.4%)	329 (30.0%) / 767 (70.0%)	0.76 (0.51-1.12)

Abbreviations: LD (learning disability); Non-LD (without learning disability); aRR (adjusted risk ratio); CI (confidence interval); LGI (lower gastrointestinal); HPB (hepatopancreaticobiliary); CNS (central nervous system).

Supplementary table 8. Cancer stage at diagnosis among patients with and without a learning disability.

	Stage 1–3 (n, %)	Stage 4 (n, %)	Unknown (n, %)	aRR (95% CI) stage 4 vs 1–3	aRR (95% CI) unknown stage vs staged
**Any cancer**					
LD	877 (39·2%)	360 (16·1%)	1001 (44·7%)	1·12 (1·02-1·22)	1·30 (1·24-1·37)
Matched controls	21212 (47·4%)	7966 (17·8%)	15541 (34·8%)		
**Digestive tract (any)**					
LD	237 (30·8%)	178 (23·1%)	355 (46·1%)	1·14 (1·01-1·27)	1·18 (1·09-1·28)
Matched controls	4267 (37·1%)	2642 (23·0%)	4581 (39·9%)		
**LGI**					
LD	166 (41·9%)	91 (23·0%)	139 (35·1%)	1·27 (1·07-1·51)	1·08 (0·94-1·24)
Matched controls	3019 (48·0%)	1189 (18·9%)	2080 (33·1%)		
**Oesophageal**					
LD	26 (22·0%)	30 (25·4%)	62 (52·5%)	1·23 (0·94-1·61)	1·42 (1·18-1·72)
Matched controls	524 (36·8%)	342 (24·1%)	556 (39·1%)		
**Stomach**					
LD	13 (17·1%)	12 (15·8%)	51 (67·1%)	0·87 (0·57-1·32)	1·41 (1·19-1·68)
Matched controls	251 (23·7%)	305 (28·8%)	504 (47·5%)		
**HPB**					
LD	27 (16·6%)	45 (27·6%)	91 (55·8%)	0·98 (0·81-1·18)	1·07 (0·93-1·23)
Matched controls	445 (17·4%)	767 (29·9%)	1351 (52·7%)		
**Breast**					
LD	201 (60·4%)	13 (3·9%)	119 (35·7%)	1·16 (0·67-1·99)	1·33 (1·15-1·55)
Matched controls	5335 (69·1%)	296 (3·8%)	2090 (27·1%)		
**Prostate**					
LD	104 (50·7%)	33 (16·1%)	68 (33·2%)	1·13 (0·85-1·50)	1·00 (0·82-1·22)
Matched controls	4025 (53·0%)	1050 (13·8%)	2517 (33·2%)		
**Lung**					
LD	72 (30·6%)	85 (36·2%)	78 (33·2%)	0·98 (0·85-1·13)	1·21 (1·00-1·46)
Matched controls	2177 (32·8%)	2620 (39·5%)	1834 (27·7%)		
**Gynae** (any)					
LD	106 (50·7%)	12 (5·7%)	91 (43·5%)	0·84 (0·49-1·43)	1·42 (1·20-1·67)
Matched controls	1875 (59·9%)	300 (9·6%)	956 (30·5%)		
**Ovary**					
LD	34 (44·7%)	†	40 (52·6%)	0·33 (0·09-1·21)	1·75 (1·39-2·20)
Matched controls	560 (52·9%)	172 (16·3%)	326 (30·8%)		
**Uterine**					
LD	66 (65·3%)	5 (5·0%)	30 (29·7%)	1·04 (0·44-2·44)	1·61 (1·17-2·21)
Matched controls	956 (74·3%)	77 (6·0%)	253 (19·7%)		
**Cervical**					
LD	†	†	6 (60·0%)	1·53 (0·31-7·61)	1·25 (0·75-2·09)
Matched controls	265 (49·6%)	28 (5·2%)	241 (45·1%)		
**Renal**					
LD	33 (41·8%)	12 (15·2%)	34 (43·0%)	1·05 (0·64-1·71)	1·08 (0·83-1·40)
Matched controls	706 (44·7%)	275 (17·4%)	599 (37·9%)		
**Urinary tract**					

LD	21 (28·4%)	5 (6·8%)	48 (64·9%)	0·90 (0·41-1·99)	1·54 (1·29-1·84)
Matched controls	772 (46·0%)	190 (11·3%)	717 (42·7%)		
**Melanoma**					
LD	55 (64·0%)	†	30 (34·9%)	0·78 (0·11-5·80)	1·10 (0·82-1·47)
Matched controls	1537 (67·2%)	33 (1·4%)	716 (31·3%)		
**Head and Neck**					
LD	16 (25·4%)	16 (25·4%)	31 (49·2%)	0·92 (0·65-1·30)	1·29 (1·00-1·67)
Matched controls	532 (27·7%)	656 (34·1%)	735 (38·2%)		
**Oropharyngeal**					
LD	†	†	8 (57·1%)	0·91 (0·54-1·54)	2·00 (1·20-3·32)
Matched controls	92 (17·1%)	300 (55·7%)	147 (27·3%)		
**Endocrine**					
LD	10 (26·3%)	†	24 (63·2%)	1·51 (0·74-3·11)	1·02 (0·80-1·31)
Matched controls	192 (31·4%)	57 (9·3%)	362 (59·2%)		
**Testicular**					
LD	41 (61·2%)	†	24 (35·8%)	1·17 (0·25-5·40)	0·85 (0·60-1·19)
Matched controls	289 (56·0%)	12 (2·3%)	215 (41·7%)		

Abbreviations: LD (learning disability); aRR (age and sex relative risk); LGI (lower gastrointestinal); OG (oesophago-gastric); HPB (hepatopancreaticobiliary); CNS (central nervous system); * not reached; † counts of <5 not shown.

Supplementary table 9. Cancer stage at diagnosis among patients with and without a learning disability.

	Stage 1–3 (n, %)	Stage 4 (n, %)	Unknown (n, %)	aRR (95% CI) stage 4 vs 1–3	aRR (95% CI) unknown stage vs staged
**LD register**	433 (39·5%)	168 (15·3%)	494 (45·1%)	1·12 (0·98-1·28)	1·44 (1·34-1·54)
**Matched control**	12479 (50·1%)	4620 (18·6%)	7805 (31·3%)		
**Mild LD**	132 (55·7%)	35 (14·8%)	70 (29·5%)	0·89 (0·66-1·19)	1·03 (0·84-1·26)
**Matched control**	2622 (53·5%)	894 (18·2%)	1383 (28·2%)		
**Moderate LD**	151 (52·1%)	40 (13·8%)	99 (34·1%)	0·87 (0·65-1·15)	1·17 (0·99-1·38)
**Matched control**	3561 (52·6%)	1261 (18·6%)	1945 (28·7%)		
**Severe LD**	76 (46·3%)	20 (12·2%)	68 (41·5%)	0·92 (0·62-1·36)	1·38 (1·14-1·67)
**Matched control**	2633 (52·6%)	881 (17·6%)	1488 (29·7%)		
**Down syndrome**	26 (32·1%)	9 (11·1%)	46 (56·8%)	1·22 (0·67-2·23)	1·63 (1·32-2·01)
**Matched control**	2102 (52·0%)	695 (17·2%)	1249 (30·9%)		

Abbreviations: LD (learning disability); aRR (adjusted relative risk); CI (confidence interval)

Supplementary table 10. Overall survival following cancer diagnosis in individuals on the learning disability register compared to individuals without a learning disability.

Cancer type	Median OS (years with 95% CI)	1-year OS (% with 95% CI)	5-year OS (% with 95% CI)	HR OS (95% CI)	aHR OS (95% CI)
**Any cancer**					
LD	4·27 (3·73-5·21)	66·7% (64·3-69·1)	47·5% (44·6-50·5)	1·60 (1·48-1·72)	1·94 (1·80-2·09)
Matched controls	10·53 (10·16-11·43)	78·7% (78·2-79·1)	60·9% (60·3-61·5)		
**Prostate**					
LD	7·55 (4·91-*)	85·5% (78·4-93·1)	58·2% (46·5-73·0)	2·04 (1·43-2·92)	2·24 (1·56-3·20)
Matched controls	* (12·84-*)	94·8% (94·1-95·4)	77·7% (76·1-79·3)		
**Breast**					
LD	10·24 (7·76-*)	91·3% (87·3-95·5)	72·1% (64·7-80·3)	2·56 (1·95-3·36)	2·43 (1·84-3·21)
Matched controls	* (*-*)	97·0% (96·5-97·5)	86·3% (85·1-87·4)		
**Digestive tract**					
LD	0·71 (0·56-1·11)	46·4% (41·6-51·6)	23·4% (18·9-28·9)	1·58 (1·40-1·79)	1·77 (1·57-2·00)
Matched controls	2·05 (1·86-2·21)	61·7% (60·5-62·9)	37·2% (35·8-38·6)		
**Haematological cancer**					
LD	7·54 (5·24-*)	79·7% (74·7-85·0)	59·5% (52·5-67·3)	1·26 (1·02-1·56)	1·92 (1·55-2·37)
Matched controls	10·30 (9·65-11·98)	83·9% (82·7-85·1)	66·4% (64·7-68·3)		
**Lung**					
LD	0·42 (0·27-0·99)	36·3% (27·0-48·9)	13·8% (5·9-32·5)	1·03 (0·80-1·32)	1·05 (0·82-1·36)
Matched controls	0·63 (0·59-0·67)	39·8% (38·2-41·5)	13·9% (12·5-15·4)		
**Gynae**					
LD	3·73 (1·33-*)	64·8% (57·2-73·5)	45·5% (36·4-56·9)	2·33 (1·81-3·00)	2·43 (1·88-3·14)
Matched controls	* (13·67-*)	85·2% (83·6-86·9)	65·2% (62·7-67·8)		
**Urinary tract**					
LD	3·33 (1·41-*)	66·4% (55·5-79·3)	42·5% (29·8-60·7)	2·25 (1·56-3·25)	2·85 (1·96-4·14)
Matched controls	13·59 (10·11-*)	85·8% (84·2-87·3)	68·5% (66·2-71·0)		

Abbreviations: OS (overall survival); CI (confidence interval); LD (learning disability); HR (hazard ratio); aHR (adjusted hazard ratio); * not reached

Supplementary table 11. Overall survival following cancer diagnosis in individuals with mild learning disability compared to individuals without a learning disability.

Cancer type	Median OS (years with 95% CI)	1-year OS (% with 95% CI)	5-year OS (% with 95% CI)	HR OS (95% CI)	aHR OS (95% CI)
**Any cancer**					
LD	12·22 (8·35-*)	78·6% (74·2-83·2)	64·9% (59·1-71·2)	1·00 (0·82-1·21)	1·12 (0·92-1·37)
Matched controls	12·84 (10·61-*)	80·3% (79·3-81·4)	63·5% (62·1-64·9)		
**Prostate**					
LD	9·51 (7·55-*)	88·9% (77·6-100·0)	72·0% (54·6-94·9)	1·88 (0·92-3·83)	1·70 (0·82-3·50)
Matched controls	* (*-*)	95·5% (94·1-97·0)	81·4% (78·1-84·9)		
**Breast**					
LD	12·22 (*-*)	100·0% (100·0-100·0)	90·0% (77·7-100·0)	0·90 (0·33-2·46)	0·86 (0·31-2·34)
Matched controls	14·51 (14·35-*)	97·2% (96·1-98·3)	89·2% (86·7-91·6)		
**Digestive tract**					
LD	2·32 (0·65-*)	55·8% (45·5-68·4)	40·1% (29·3-54·8)	1·14 (0·83-1·55)	1·26 (0·92-1·72)
Matched controls	2·52 (2·03-3·01)	63·8% (61·1-66·6)	39·8% (36·6-43·2)		
**Haematological cancer**					
LD	* (7·90-*)	81·2% (71·3-92·4)	70·2% (56·9-86·6)	0·96 (0·57-1·62)	1·21 (0·70-2·08)
Matched controls	10·61 (8·73-*)	86·0% (83·4-88·6)	69·2% (65·2-73·4)		
**Lung**					
LD	0·49 (0·23-*)	42·3% (24·3-73·5)	0·0% (**-**)	1·01 (0·60-1·72)	0·92 (0·54-1·57)
Matched controls	0·67 (0·57-0·77)	41·5% (37·9-45·4)	16·7% (13·5-20·7)		
**Gynae**					
LD	* (5·26-*)	83·1% (70·6-97·8)	71·3% (56·2-90·5)	1·05 (0·53-2·07)	0·98 (0·49-1·97)
Matched controls	* (*-*)	86·2% (82·7-89·8)	62·3% (56·5-68·6)		
**Urinary tract**					
LD	* (1·46-*)	80·2% (62·4-100·0)	61·9% (39·7-96·5)	1·38 (0·56-3·41)	1·25 (0·50-3·14)
Matched controls	11.60 (9.80-*)	87.4% (84.1-90.8)	71.2% (66.0-76.8)		

Abbreviations: OS (overall survival); CI (confidence interval); LD (learning disability); HR (hazard ratio); aHR (adjusted hazard ratio); *not reached; **not estimable

Supplementary table 12. Overall survival following cancer diagnosis in individuals with moderate learning disability compared to individuals without a learning disability.

Cancer type	Median OS (years with 95% CI)	1-year OS (% with 95% CI)	5-year OS (% with 95% CI)	HR OS (95% CI)	aHR OS (95% CI)
**Any cancer**					
LD	11·07 (5·68-*)	79·9% (76·1-83·9)	59·0% (53·7-64·9)	1·08 (0·91-1·27)	1·21 (1·02-1·42)
Matched controls	13·52 (12·08-*)	80·6% (79·7-81·4)	63·0% (61·8-64·2)		
**Prostate**					
LD	7·55 (4·66-*)	82·3% (70·3-96·3)	57·4% (39·8-82·7)	2·08 (1·16-3·74)	2·13 (1·18-3·83)
Matched controls	* (*-*)	95·8% (94·6-97·0)	80·0% (77·1-83·1)		
**Breast**					
LD	11·07 (9·29-*)	100·0% (100·0-100·0)	78·9% (66·7-93·4)	1·99 (1·10-3·58)	2·00 (1·10-3·63)
Matched controls	* (14·51-*)	98·1% (97·4-98·9)	88·9% (86·9-91·0)		
**Digestive tract**					
LD	2·36 (1·13-4·13)	62·8% (53·9-73·1)	33·1% (23·6-46·4)	1·14 (0·87-1·48)	1·19 (0·91-1·55)
Matched controls	2·29 (2·02-2·73)	63·1% (60·8-65·5)	38·7% (36·1-41·6)		
**Haematological cancer**					
LD	* (5·27-*)	84·8% (77·2-93·1)	65·3% (53·5-79·6)	1·00 (0·66-1·52)	1·29 (0·85-1·97)
Matched controls	* (10·04-*)	86·3% (84·3-88·5)	68·1% (64·8-71·6)		
**Lung**					
LD	3·17 (0·85-*)	63·9% (45·5-89·8)	33·9% (15·7-73·2)	0·52 (0·28-0·94)	0·55 (0·30-1·00)
Matched controls	0·70 (0·59-0·80)	42·7% (39·6-46·0)	16·3% (13·5-19·7)		
**Gynae**					
LD	* (4·51-*)	77·7% (65·1-92·8)	54·9% (36·9-81·6)	1·35 (0·75-2·43)	1·25 (0·69-2·26)
Matched controls	* (*-*)	86·7% (83·8-89·8)	65·8% (60·9-71·1)		
**Urinary tract**					
LD	* (0·49-*)	68·2% (49·9-93·1)	53·0% (33·2-84·7)	1·86 (0·91-3·79)	2·24 (1·06-4·72)
Matched controls	* (10·00-*)	86·5% (83·5-89·5)	69·5% (64·8-74·5)		

Abbreviations: OS (overall survival); CI (confidence interval); LD (learning disability); HR (hazard ratio); aHR (adjusted hazard ratio); * not reached

Supplementary table 13. Overall survival following cancer diagnosis in individuals with severe learning disability compared to individuals without a learning disability.

Cancer type	Median OS (years with 95% CI)	1-year OS (% with 95% CI)	5-year OS (% with 95% CI)	HR OS (95% CI)	aHR OS (95% CI)
**Any cancer**					
LD	5·21 (3·17-*)	72·5% (67·1-78·4)	50·7% (43·9-58·5)	1·46 (1·21-1·77)	1·94 (1·60-2·36)
Matched controls	12·13 (10·59-*)	80·8% (79·8-81·8)	62·5% (61·1-63·9)		
**Prostate**					
LD	4·91 (2·23-*)	90·0% (73·2-100·0)	32·1% (7·3-100·0)	3·16 (1·16-8·55)	3·95 (1·40-11·15)
Matched controls	* (12·84-*)	95·7% (94·3-97·1)	77·5% (73·8-81·5)		
**Breast**					
LD	13·48 (5·21-*)	93·2% (84·6-100·0)	72·6% (56·8-92·6)	2·38 (1·21-4·69)	2·34 (1·14-4·83)
Matched controls	* (*-*)	97·1% (96·0-98·1)	86·8% (84·4-89·4)		
**Digestive tract**					
LD	1·27 (0·61-2·54)	52·5% (41·8-66·1)	25·1% (15·6-40·6)	1·56 (1·16-2·08)	1·66 (1·24-2·24)
Matched controls	2·12 (1·77-2·75)	64·1% (61·4-66·8)	37·9% (34·8-41·2)		
**Haematological cancer**					
LD	* (13·92-*)	90·6% (82·3-99·8)	73·7% (59·7-90·9)	0·67 (0·36-1·27)	1·22 (0·63-2·34)
Matched controls	10·72 (10·25-*)	86·6% (84·1-89·1)	67·9% (64·0-72·0)		
**Lung**					
LD	0·82 (0·01-*)	44·4% (21·4-92·3)	16·7% (3·2-88·2)	0·90 (0·42-1·89)	0·87 (0·39-1·95)
Matched controls	0·68 (0·60-0·78)	41·2% (37·6-45·1)	11·0% (8·2-14·7)		
**Gynae**					
LD	* (1·25-*)	72·9% (56·5-94·1)	52·6% (32·5-85·3)	1·67 (0·85-3·30)	2·43 (1·18-5·00)
Matched controls	14·24 (10·18-*)	86·4% (83·0-89·9)	66·6% (61·3-72·4)		
**Urinary tract**					
LD	* (0·28-*)	66·7% (44·7-99·5)	66·7% (44·7-99·5)	1·99 (0·73-5·43)	2·91 (1·05-8·07)
Matched controls	* (10·25-*)	87·9% (84·5-91·4)	71·9% (66·6-77·7)		

Abbreviations: OS (overall survival); CI (confidence interval); LD (learning disability); HR (hazard ratio); aHR (adjusted hazard ratio); * not reached

Supplementary table 14. Overall survival following cancer diagnosis in individuals with Down syndrome compared to individuals without a learning disability.

Cancer type	Median OS (years with 95% CI)	1-year OS (% with 95% CI)	5-year OS (% with 95% CI)	HR OS (95% CI)	aHR OS (95% CI)
**Any cancer**					
LD	3·77 (2·37-7·98)	66·2% (59·6-73·5)	44·8% (37·2-53·9)	2·05 (1·67-2·52)	3·29 (2·66-4·08)
Matched controls	14·42 (12·90-*)	82·6% (81·5-83·6)	67·0% (65·5-68·5)		
**Breast**					
LD	* (*-*)	100·0% (100·0-100·0)	83·3% (58·3-100·0)	2·11 (0·29-15·20)	1·55 (0·21-11·44)
Matched controls	* (*-*)	98·1% (97·3-99·0)	89·4% (87·1-91·7)		
**Digestive tract**					
LD	0·18 (0·10-1·33)	31·8% (19·2-52·6)	9·4% (2·9-31·1)	3·19 (2·21-4·62)	3·84 (2·63-5·60)
Matched controls	2·54 (2·08-3·17)	65·3% (62·3-68·4)	41·2% (37·7-45·0)		
**Haematological cancer**					
LD	7·26 (4·51-*)	79·3% (70·7-88·9)	55·7% (44·4-70·0)	1·62 (1·11-2·35)	3·36 (2·18-5·18)
Matched controls	11·98 (10·72-*)	88·1% (85·5-90·9)	71·6% (67·4-76·1)		
**Lung**					
LD	0·00 (0·00-*)	50·0% (12·5-100·0)	50·0% (12·5-100·0)	1·53 (0·21-10·87)	1·77 (0·24-12·92)
Matched controls	0·77 (0·62-1·02)	45·9% (41·8-50·4)	17·4% (13·8-22·0)		
**Gynae**					
LD	* (0·10-*)	69·2% (48·2-99·5)	55·4% (31·4-97·8)	2·36 (0·96-5·81)	2·68 (1·08-6·67)
Matched controls	* (*-*)	88·0% (84·5-91·5)	71·0% (65·6-76·7)		
**Urinary tract**					
LD	0·49 (0·36-*)	19·0% (3·7-98·7)	0·0% (*-*)	11·36 (4·96-26·00)	18·49 (7·63-44·82)
Matched controls	14·42 (10·49-*)	90·5% (87·1-94·2)	80·1% (74·6-85·9)		

Abbreviations: OS (overall survival); CI (confidence interval); LD (learning disability); HR (hazard ratio); aHR (adjusted hazard ratio); * not reached; ** not estimable

Supplementary figures

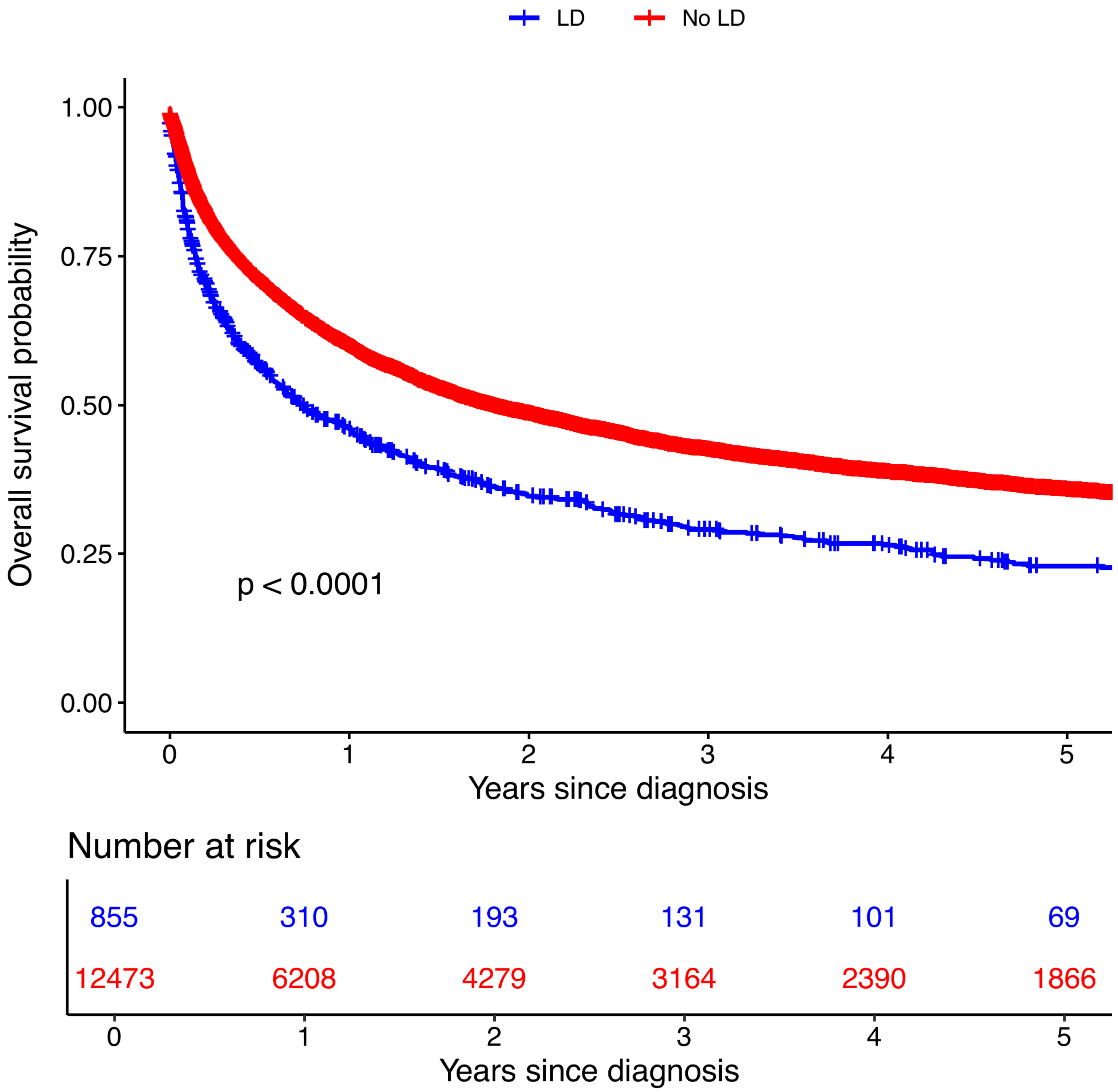

Supplementary figure 1. Overall survival among patients diagnosed with digestive tract cancer (all)

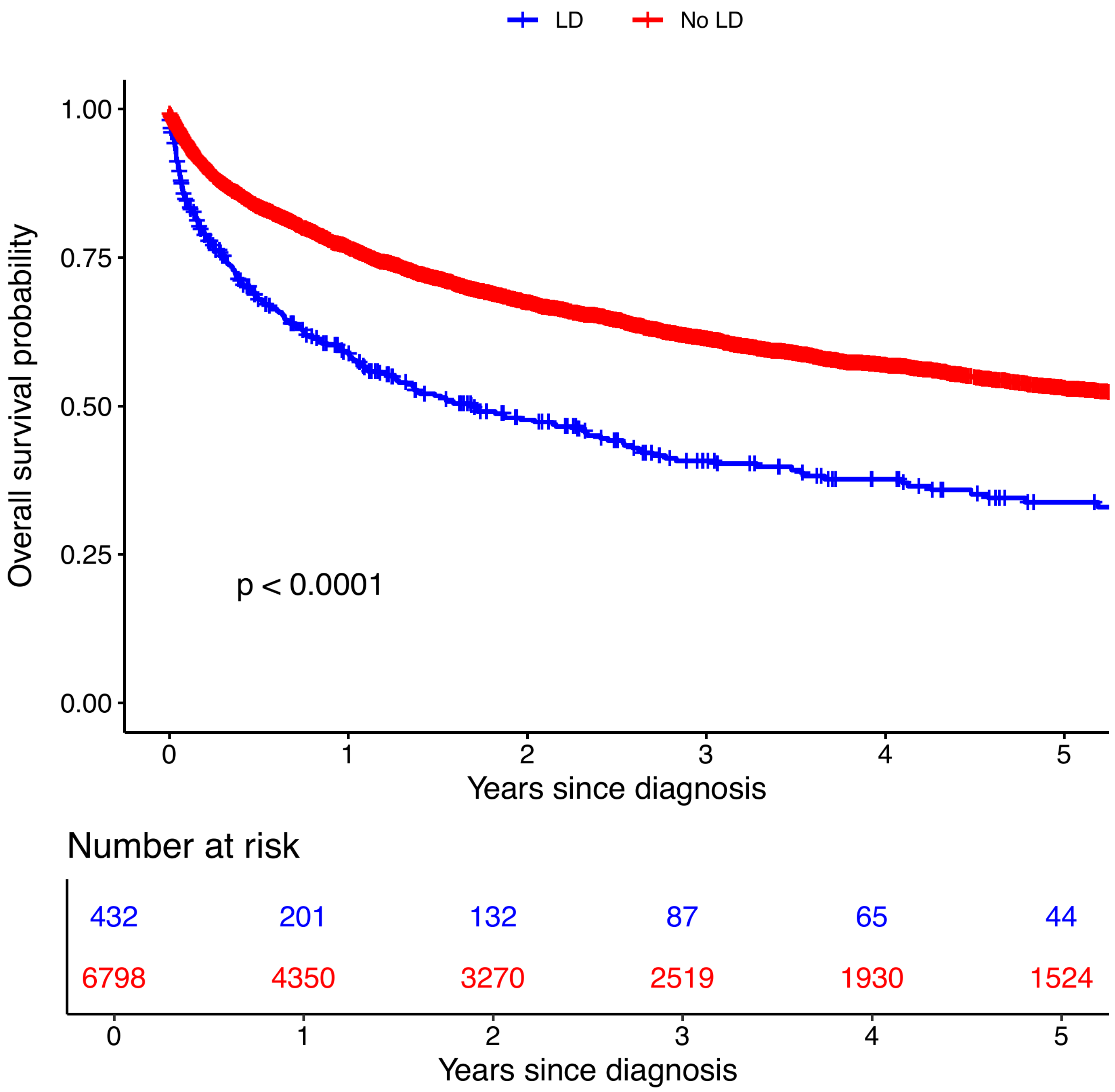

Supplementary figure 2. Overall survival among patients diagnosed with lower gastrointestinal cancer

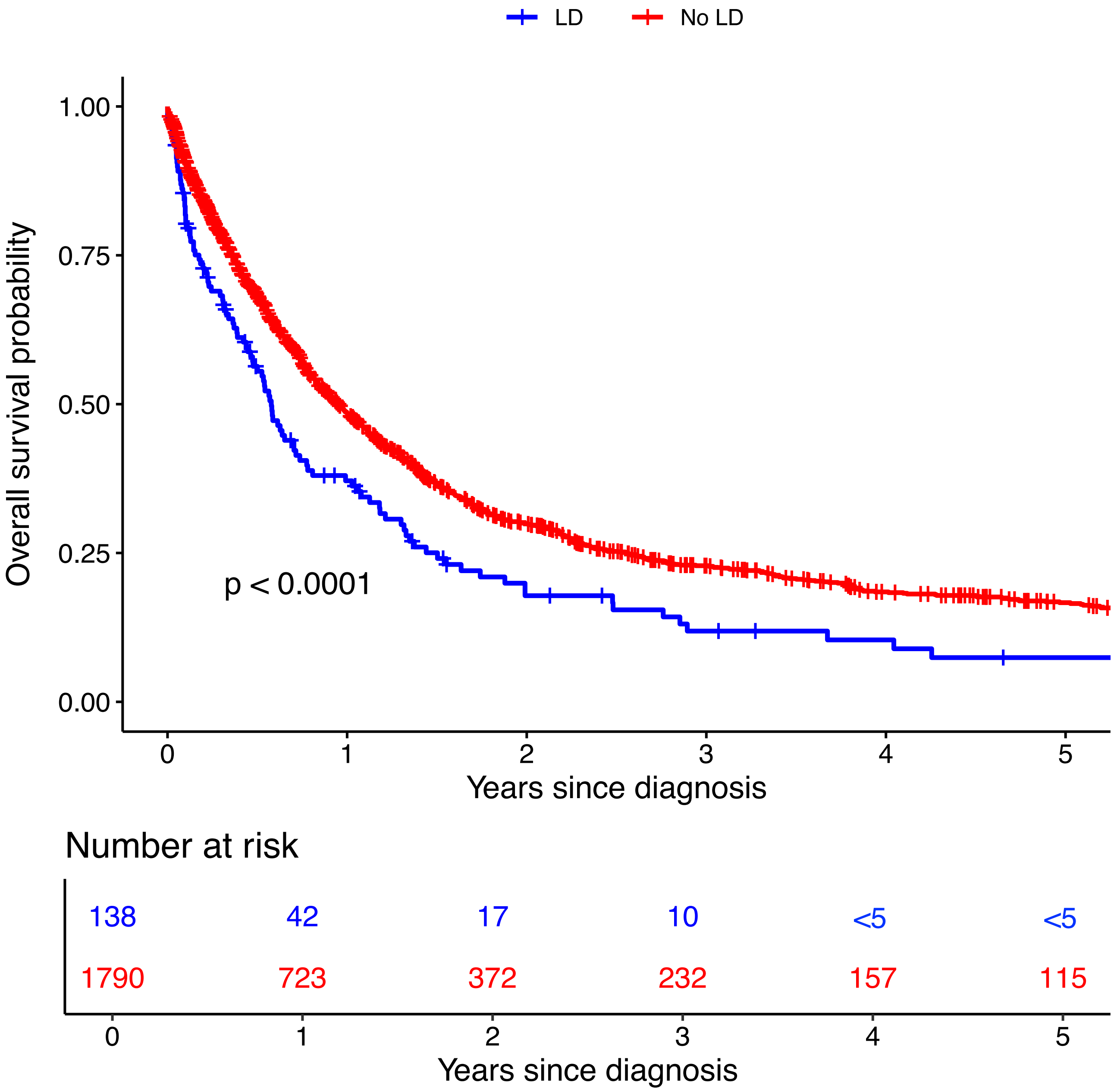

Supplementary figure 3. Overall survival among patients diagnosed with oesophageal cancer

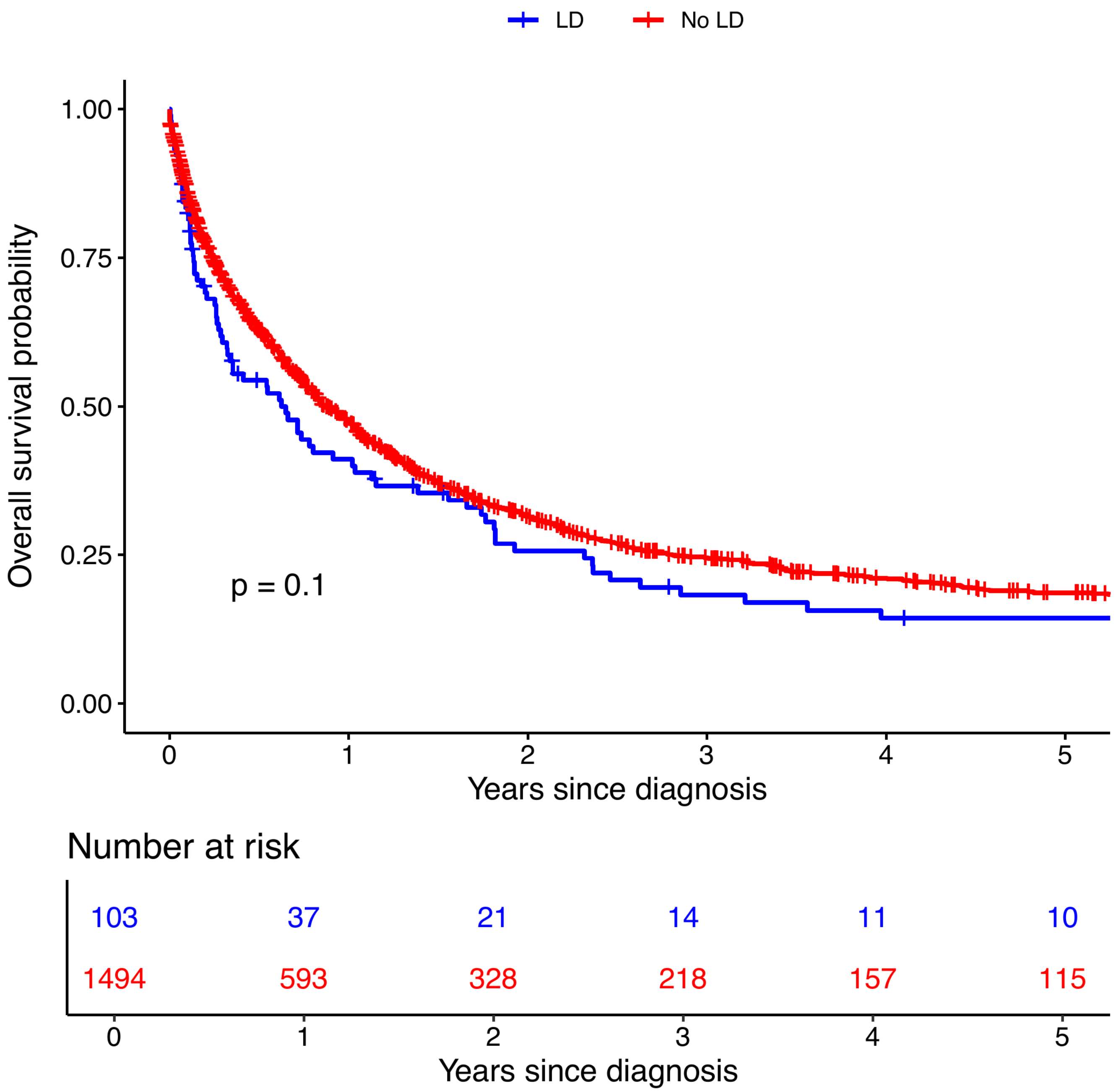

Supplementary figure 4. Overall survival among patients diagnosed with stomach cancer

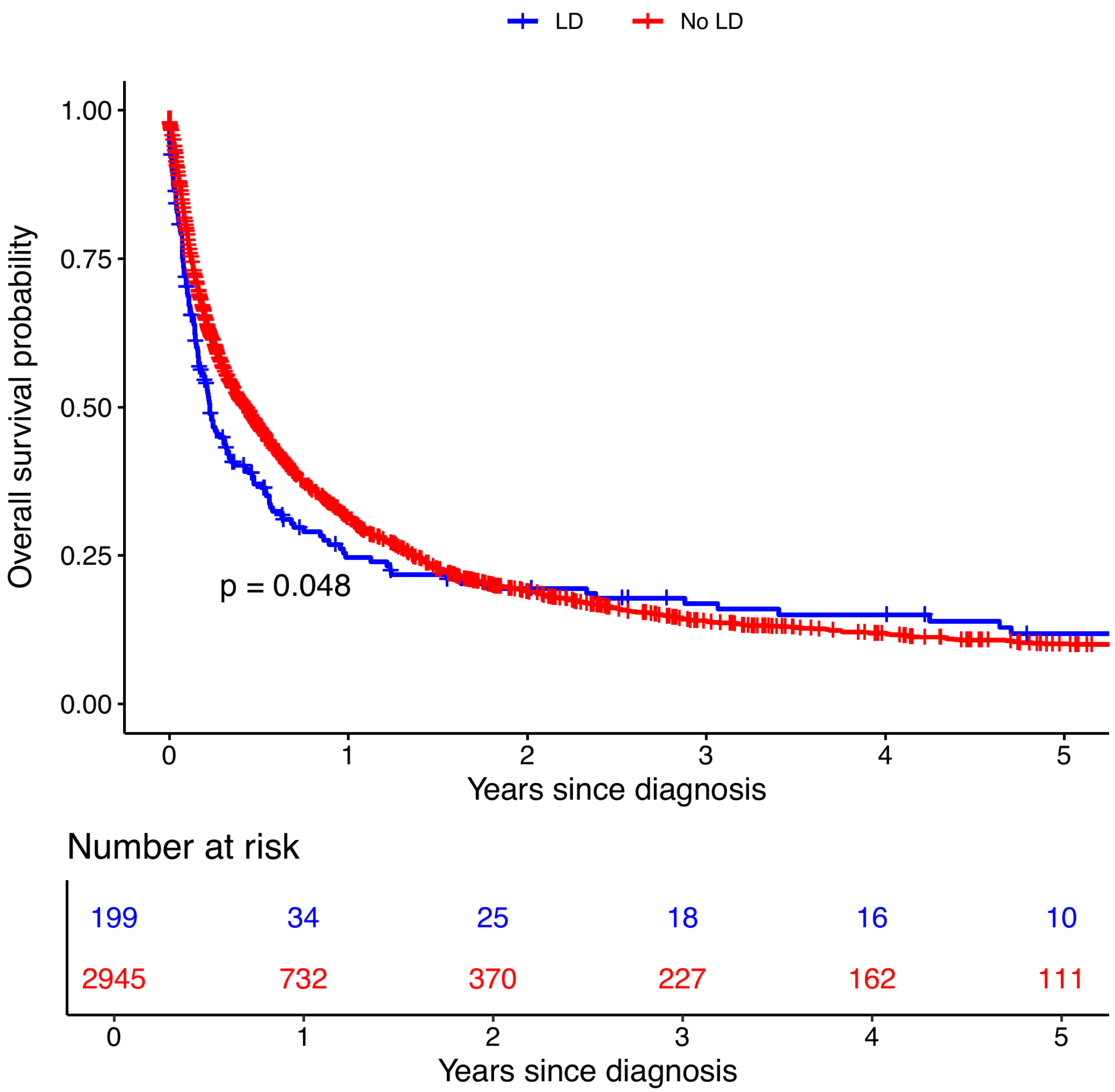

Supplementary figure 5. Overall survival among patients diagnosed with hepatopancreaticobiliary cancer

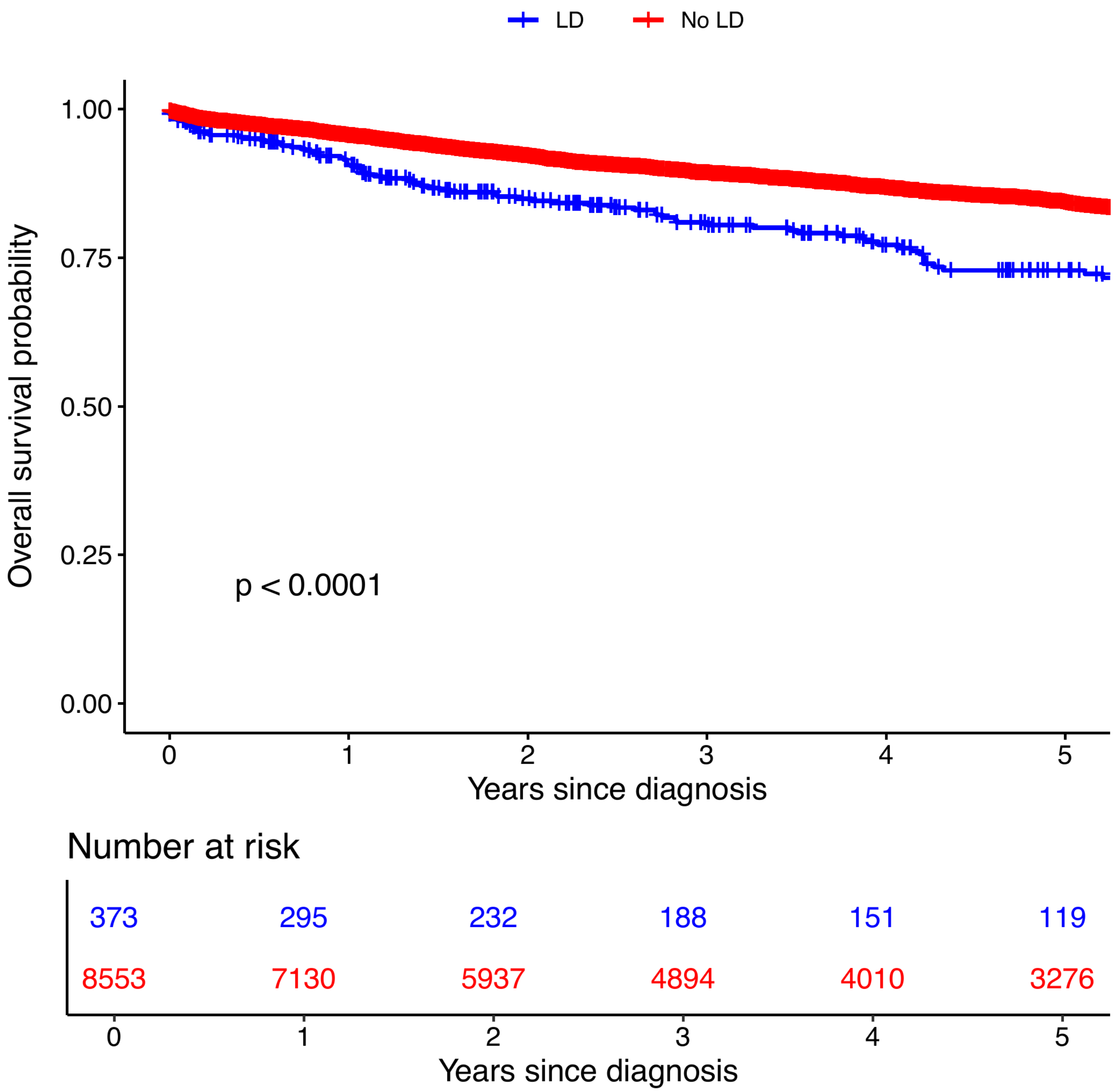

Supplementary figure 6. Overall survival among patients diagnosed with breast cancer

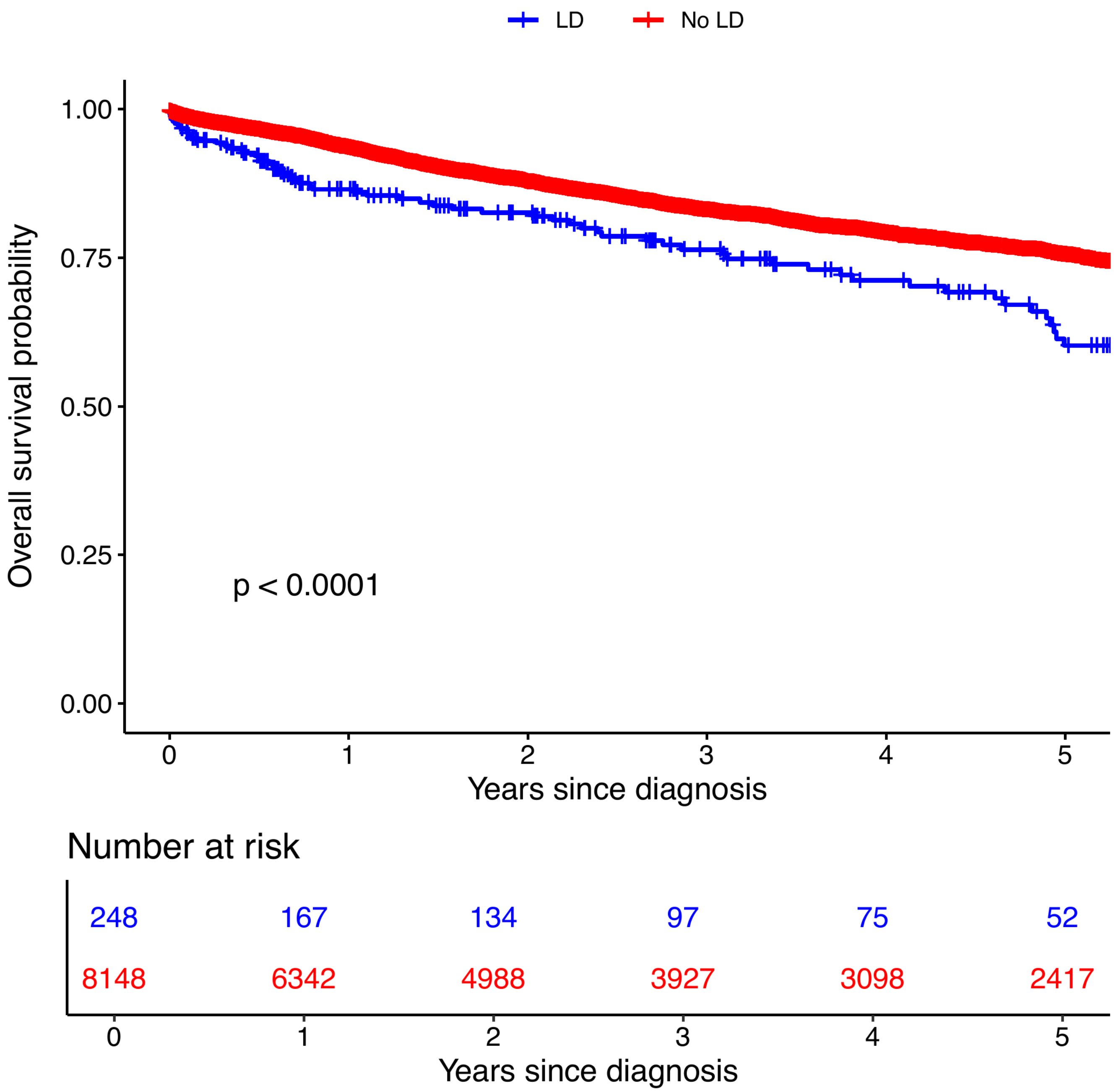

Supplementary figure 7. Overall survival among patients diagnosed with prostate cancer

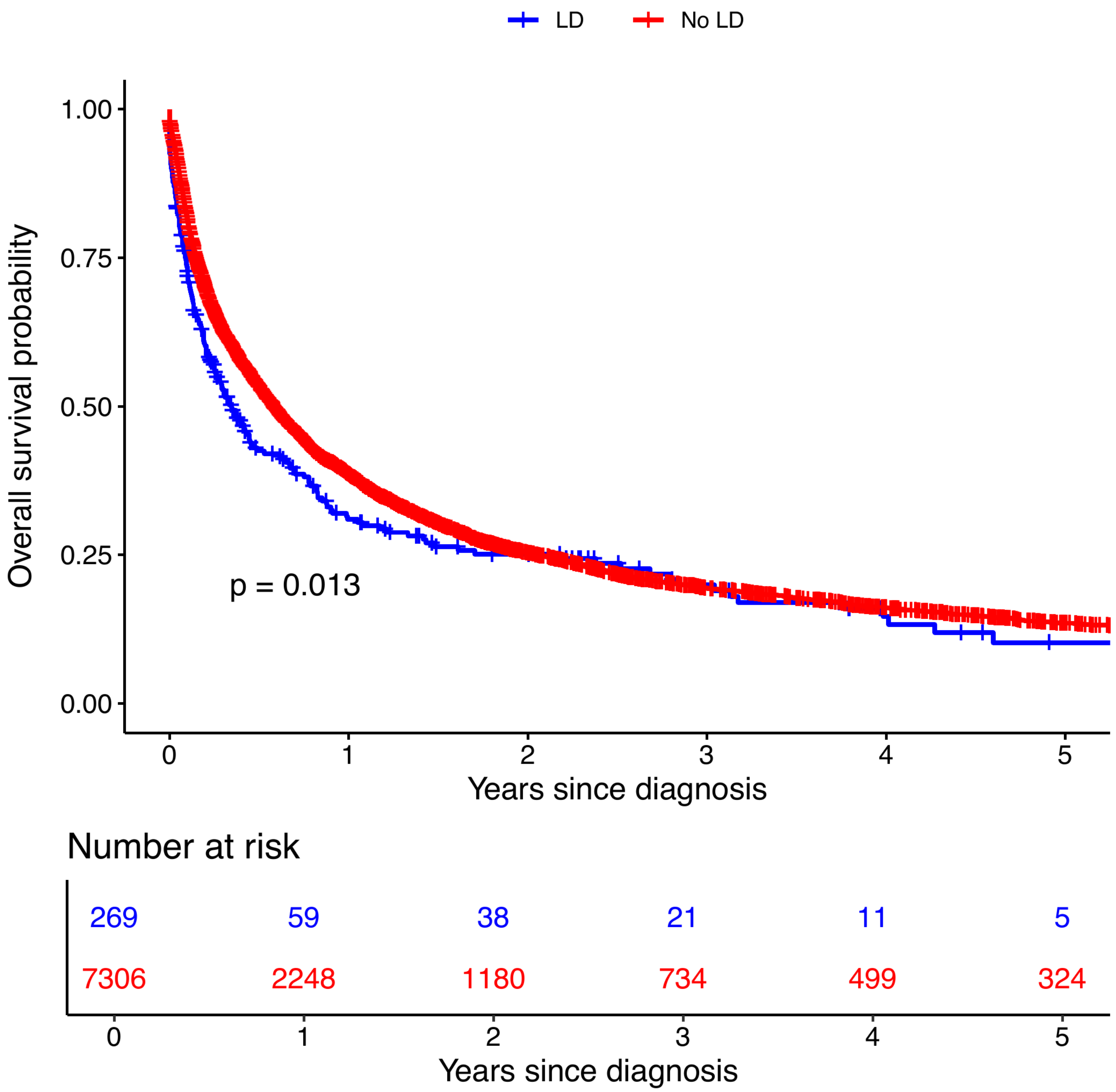

Supplementary figure 8. Overall survival among patients diagnosed with lung cancer

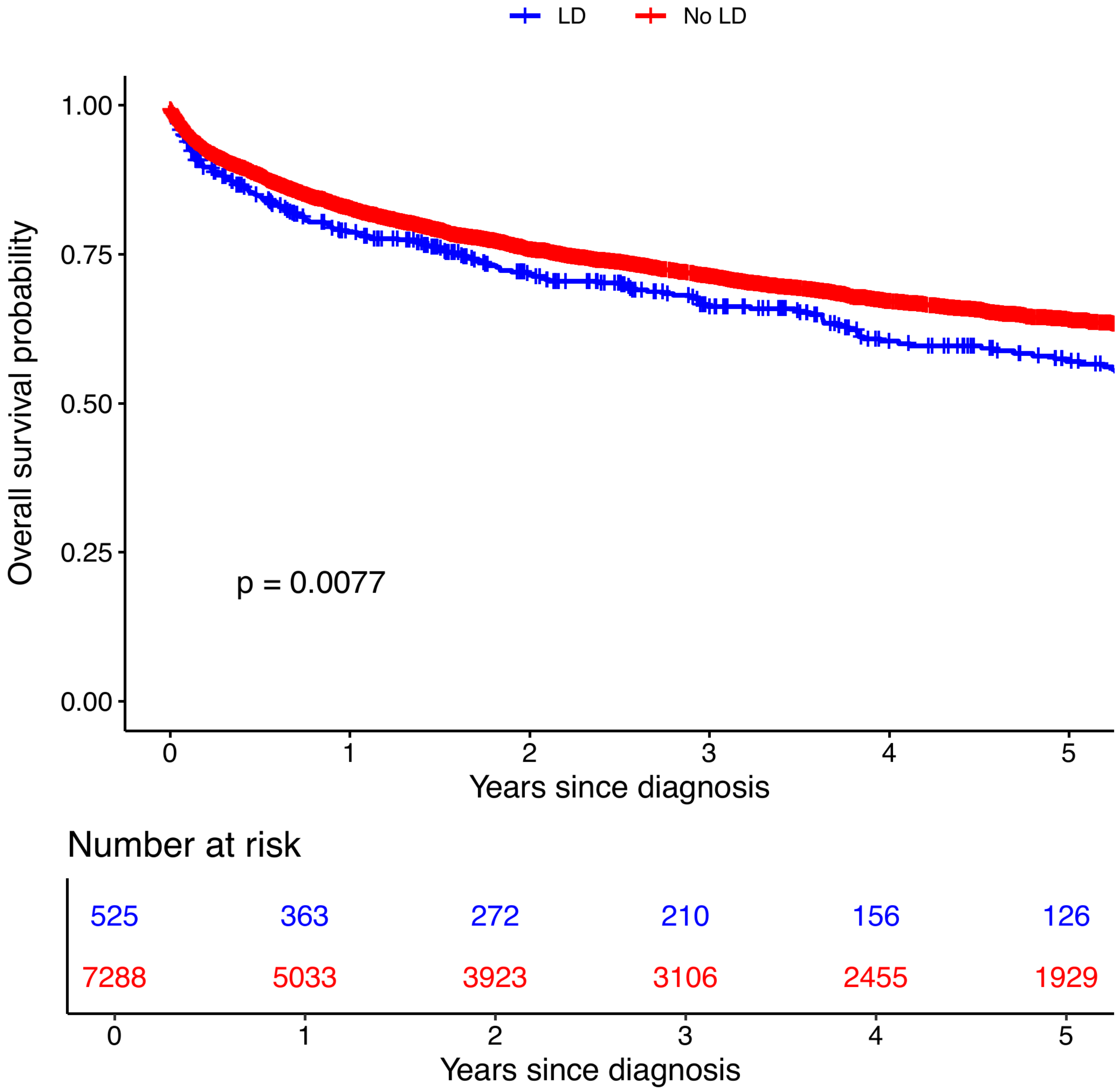

Supplementary figure 9. Overall survival among patients diagnosed with haematological cancer (all)

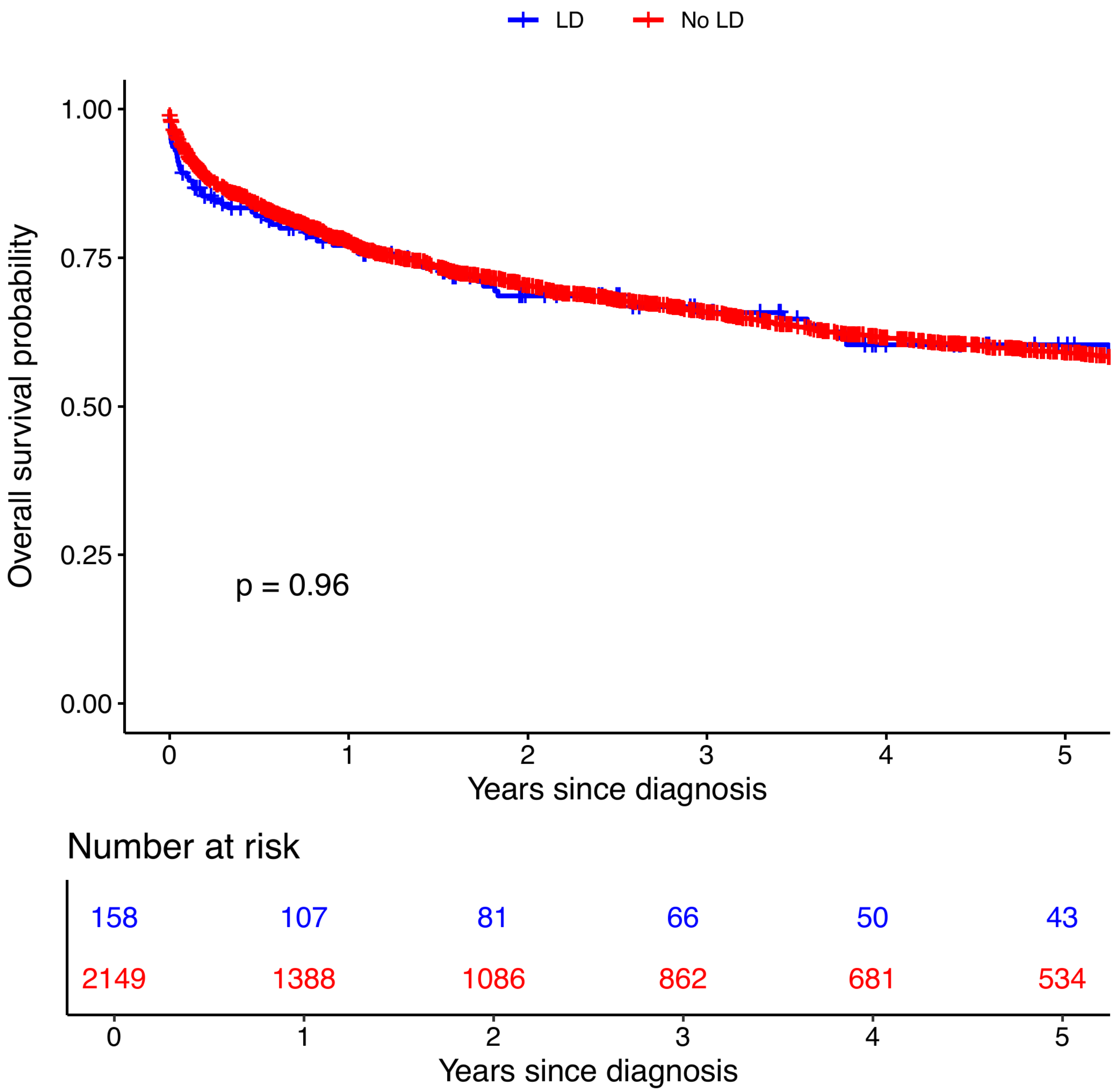

Supplementary figure 10. Overall survival among patients diagnosed with leukaemia

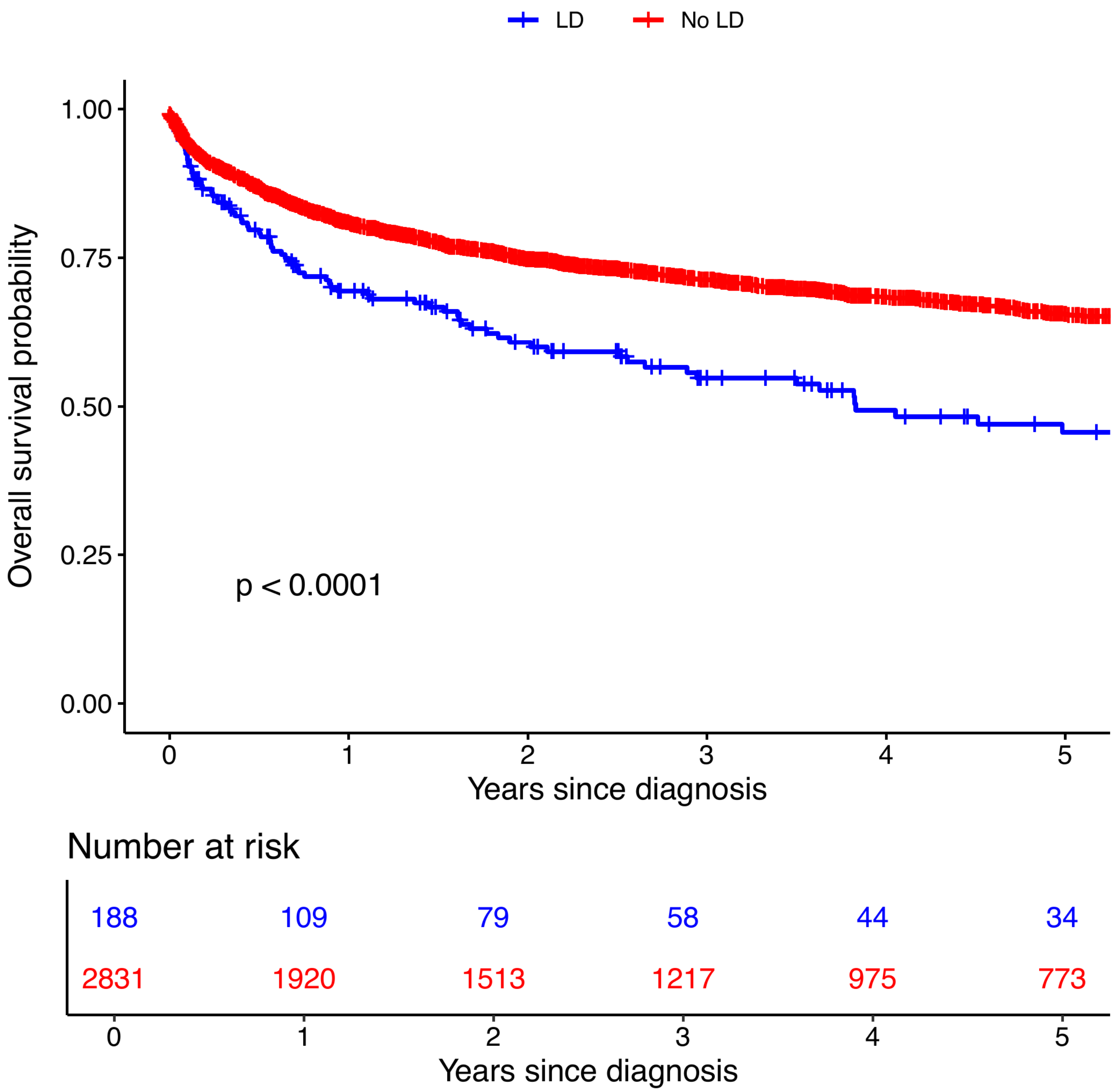

Supplementary figure 11. Overall survival among patients diagnosed with lymphoma

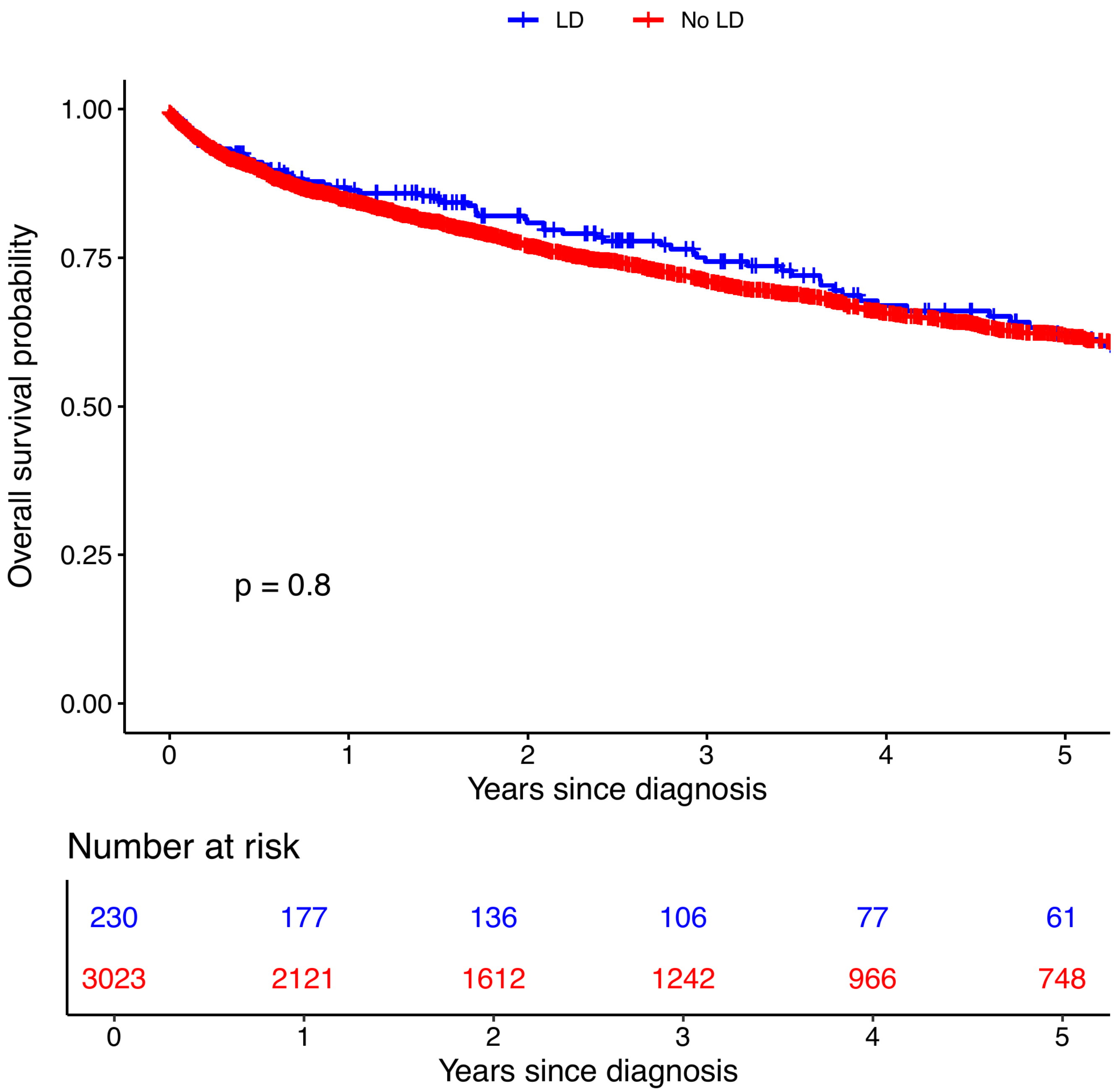

Supplementary figure 12. Overall survival among patients diagnosed with other haematological cancer

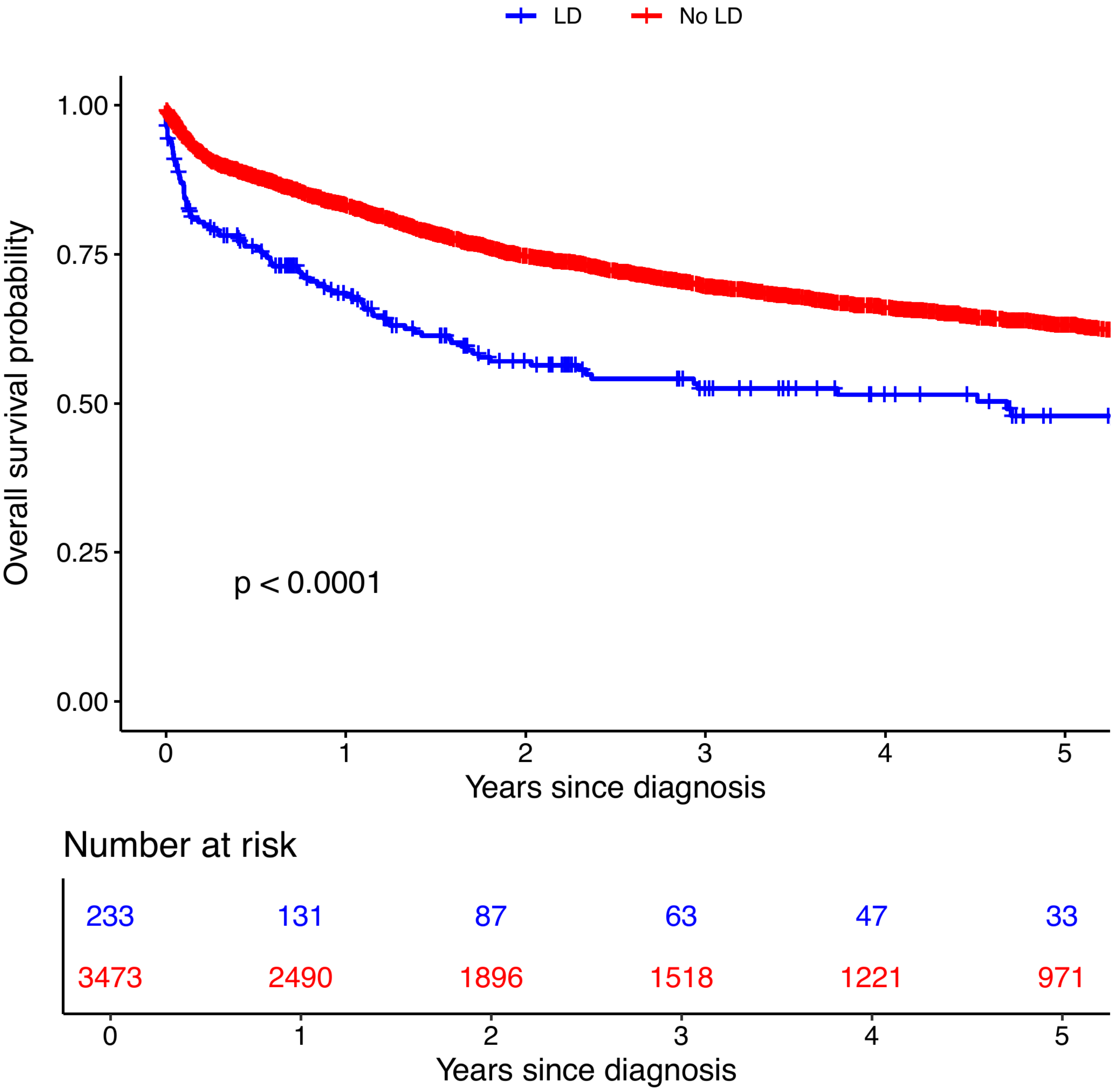

Supplementary figure 13. Overall survival among patients diagnosed with gynaecological cancer (all).

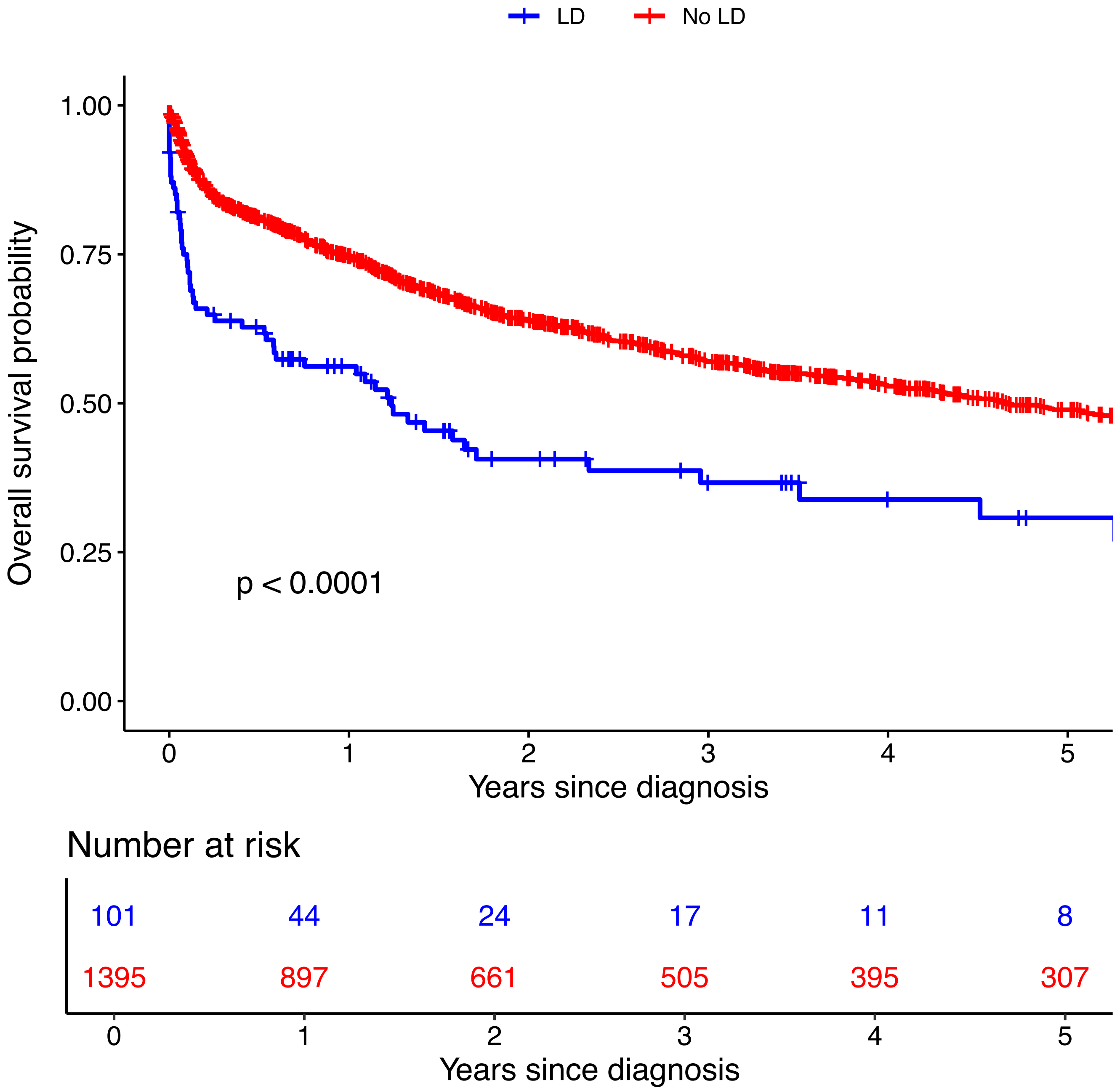

Supplementary figure 14. Overall survival among patients diagnosed with ovarian cancer.

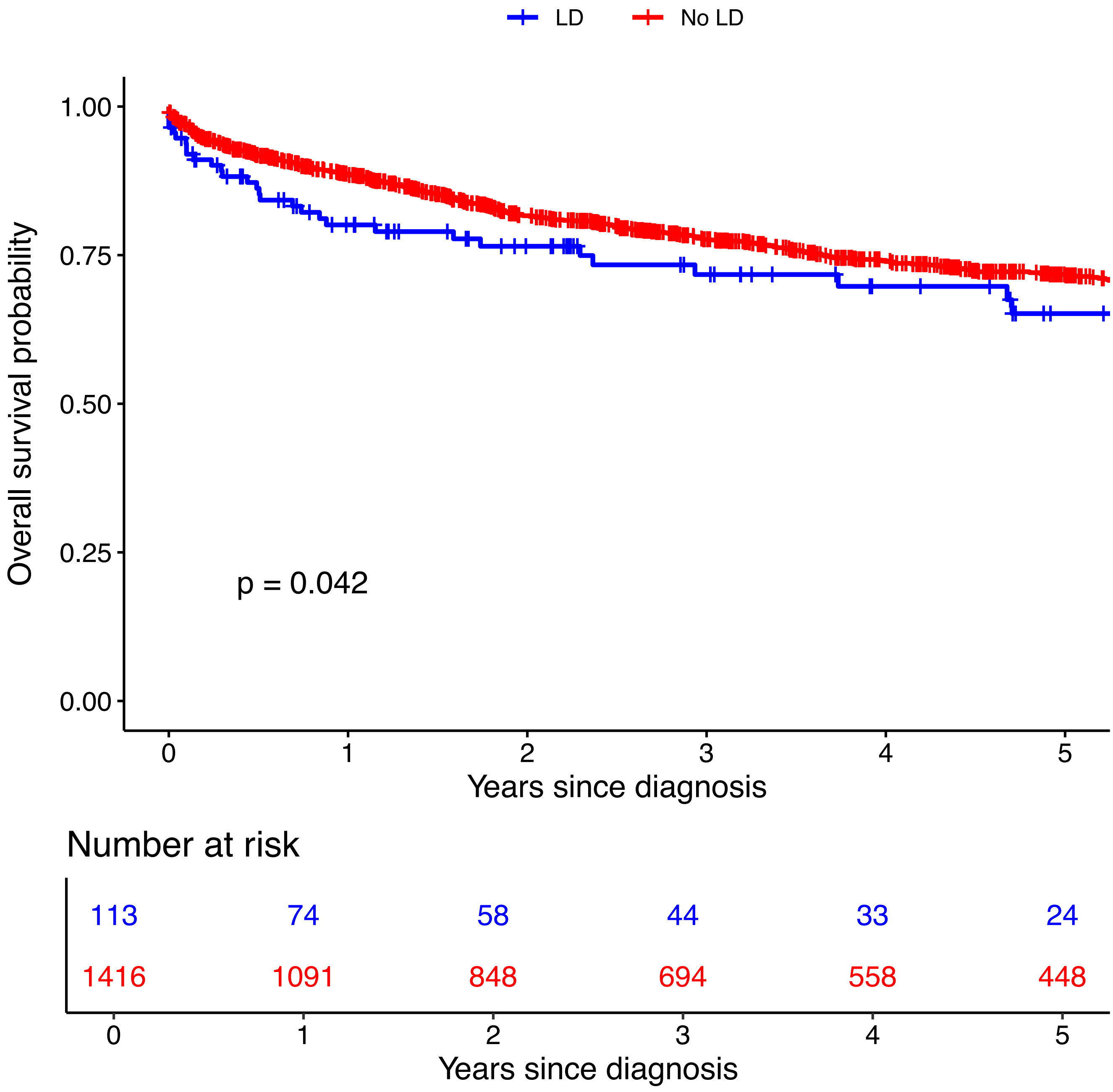

Supplementary figure 15. Overall survival among patients diagnosed with uterine cancer.

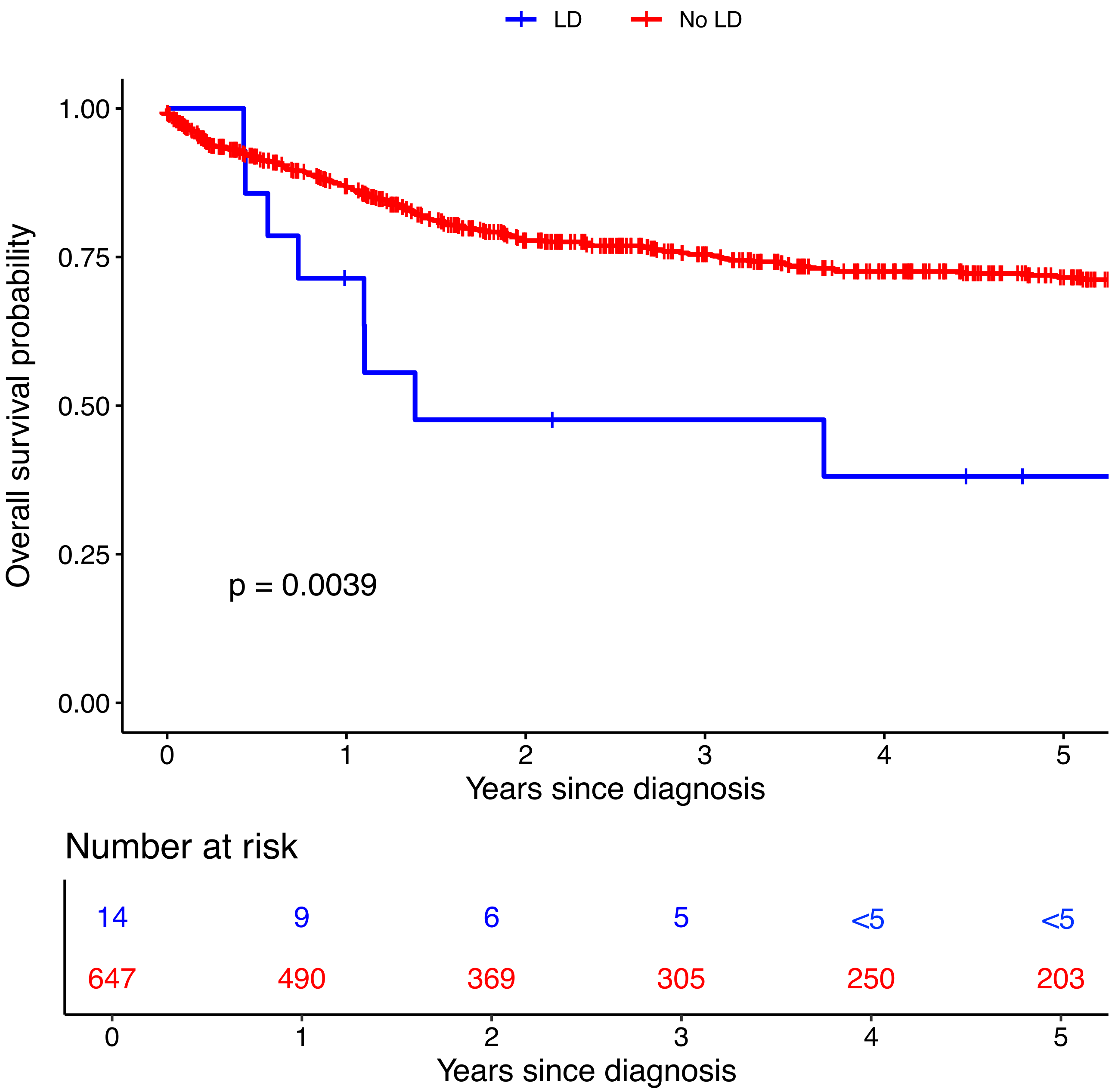

Supplementary figure 16. Overall survival among patients diagnosed with cervical cancer.

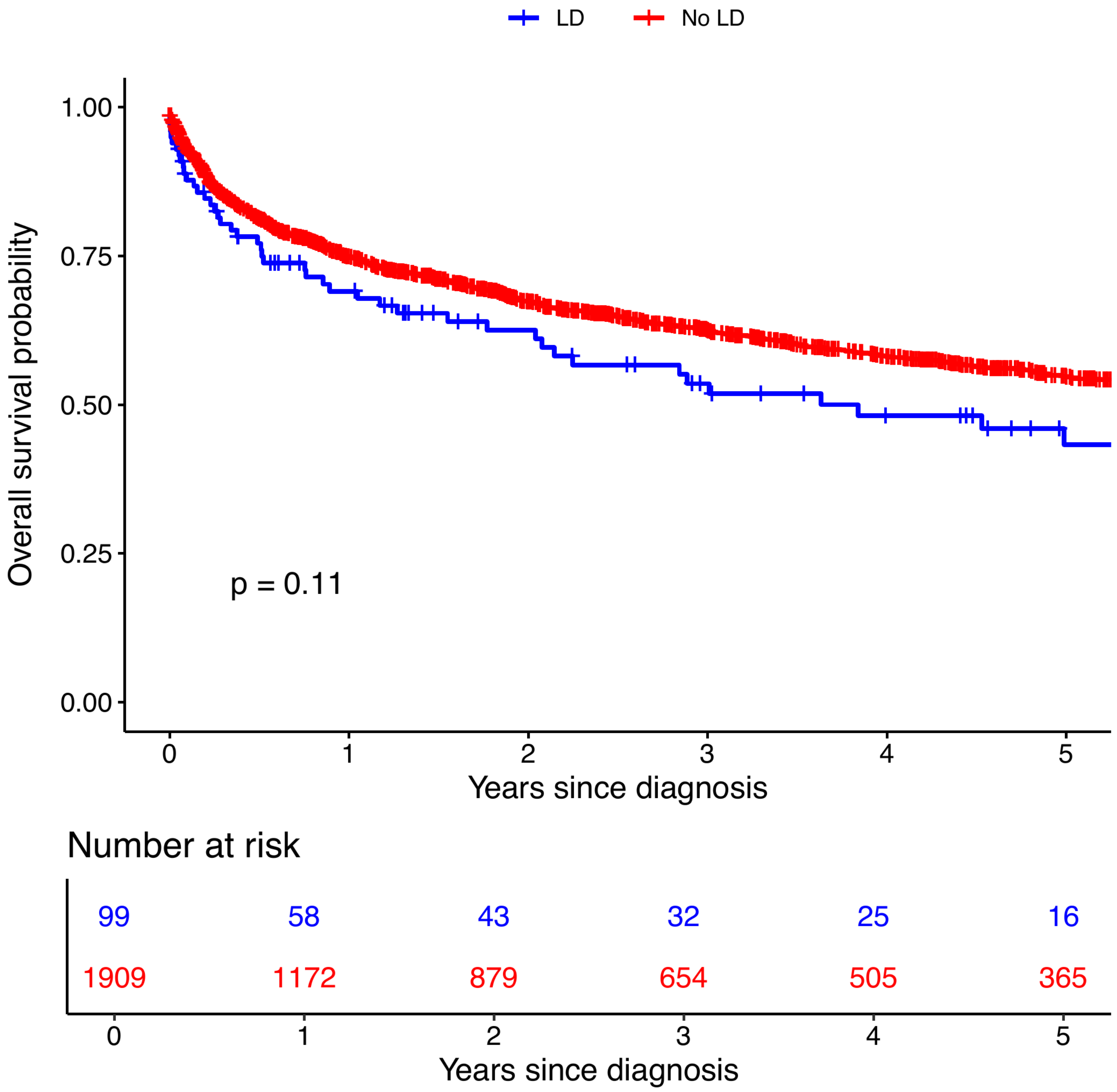

Supplementary figure 17. Overall survival among patients diagnosed with kidney cancer

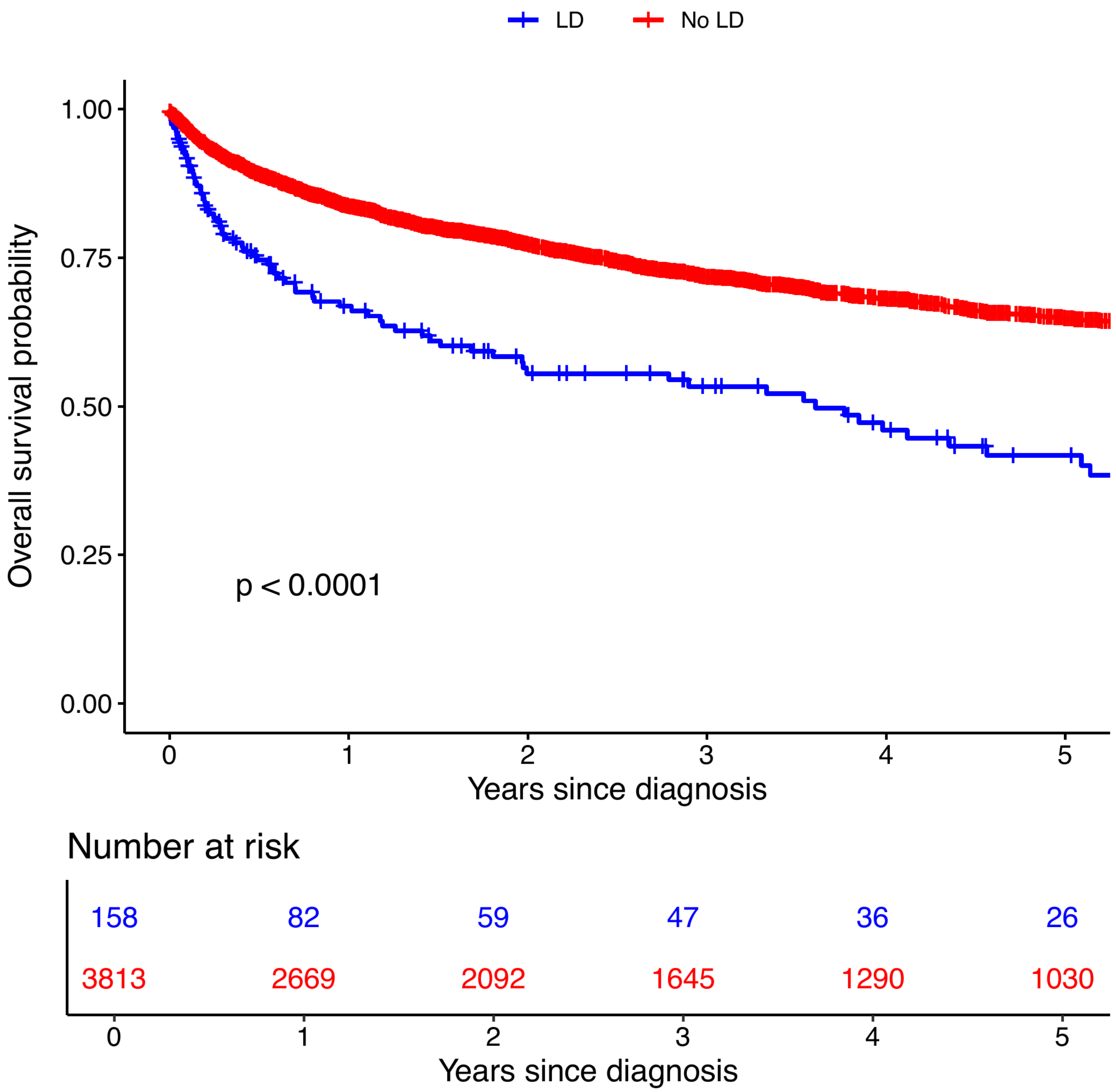

Supplementary figure 18. Overall survival among patients diagnosed with urinary tract cancer (excluding kidney)

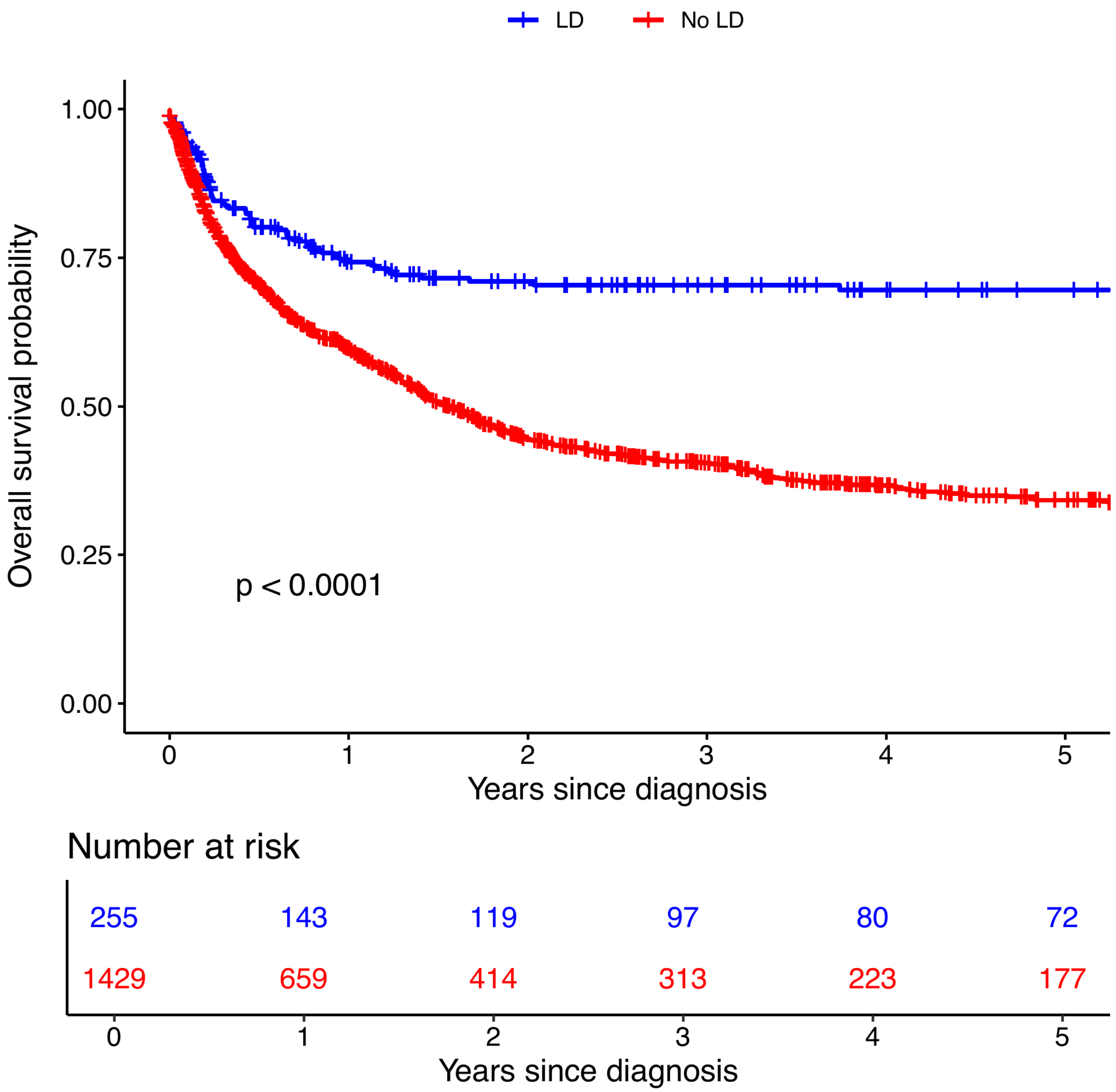

Supplementary figure 19. Overall survival among patients diagnosed with central nervous system cancer

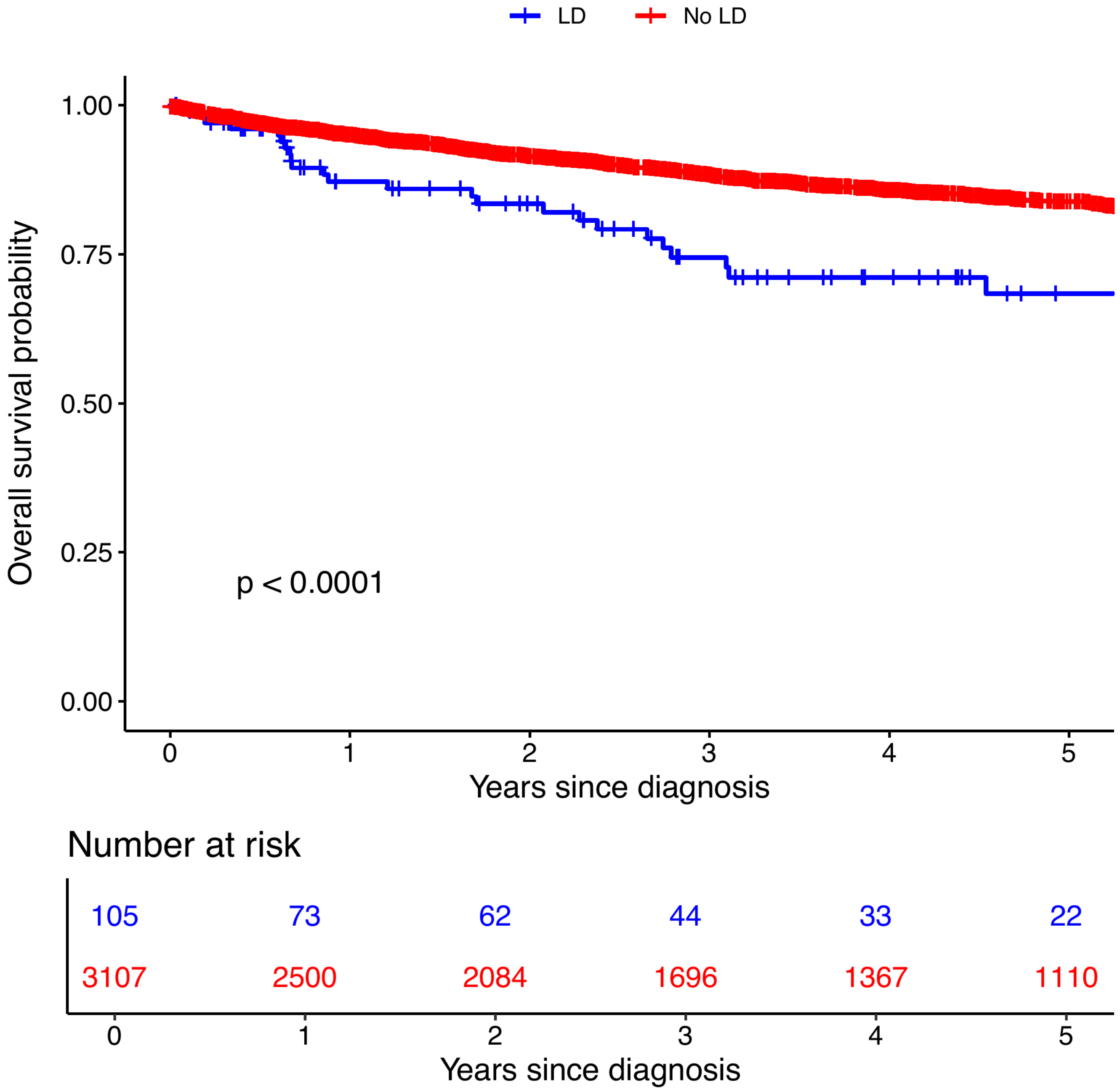

Supplementary figure 20. Overall survival among patients diagnosed with melanoma

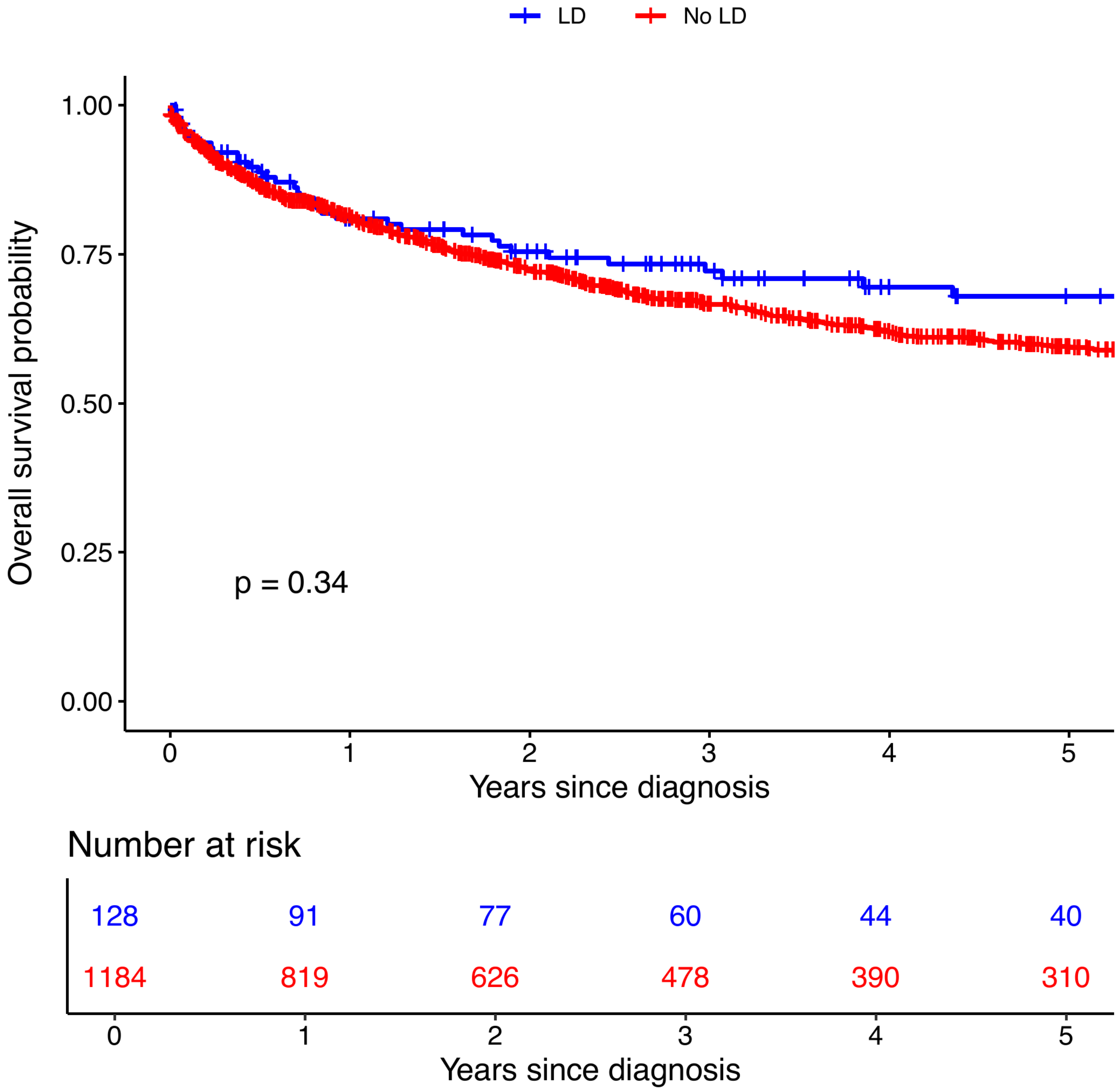

Supplementary figure 21. Overall survival among patients diagnosed with sarcoma

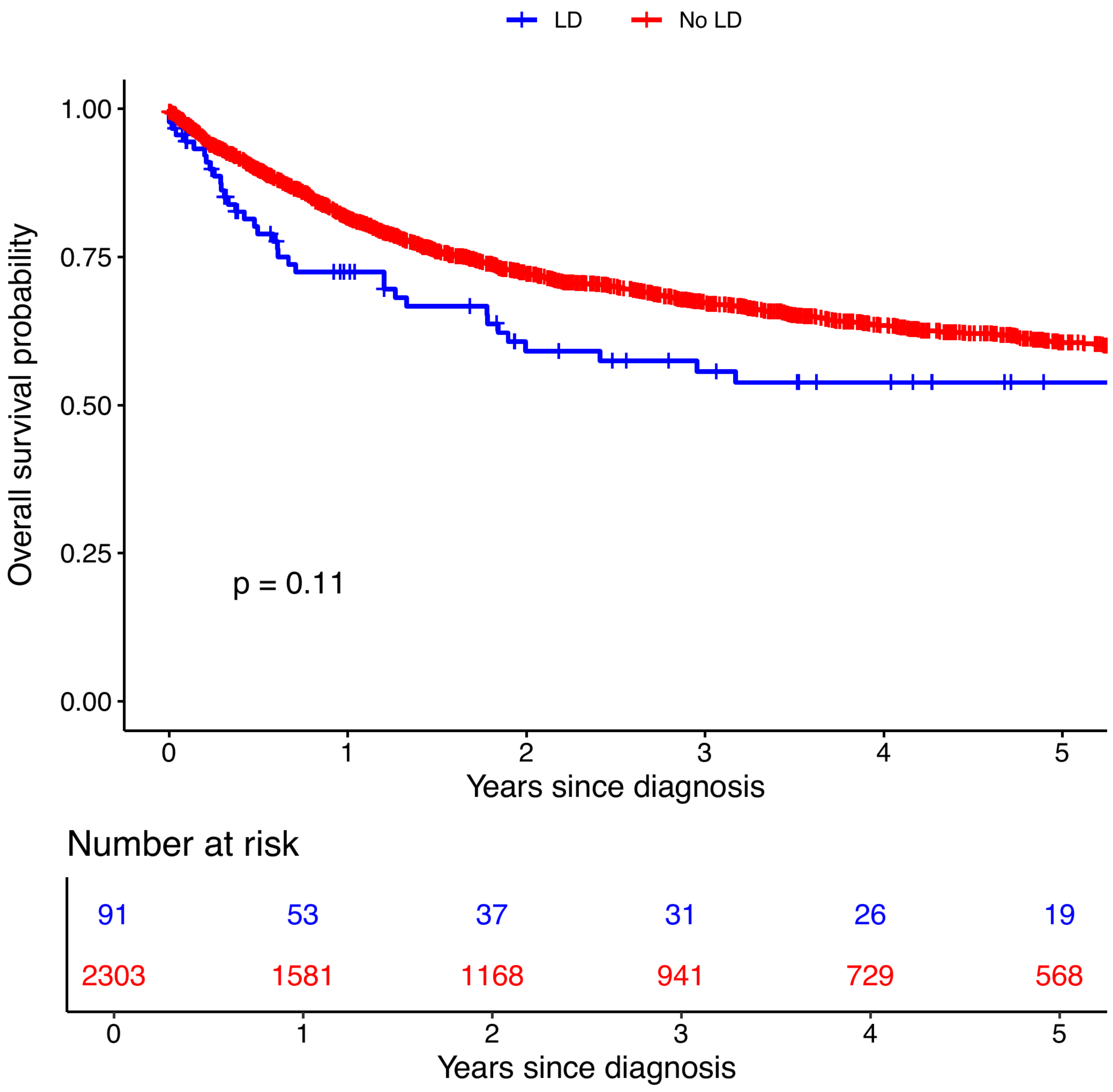

Supplementary figure 22. Overall survival among patients diagnosed with head and neck cancer

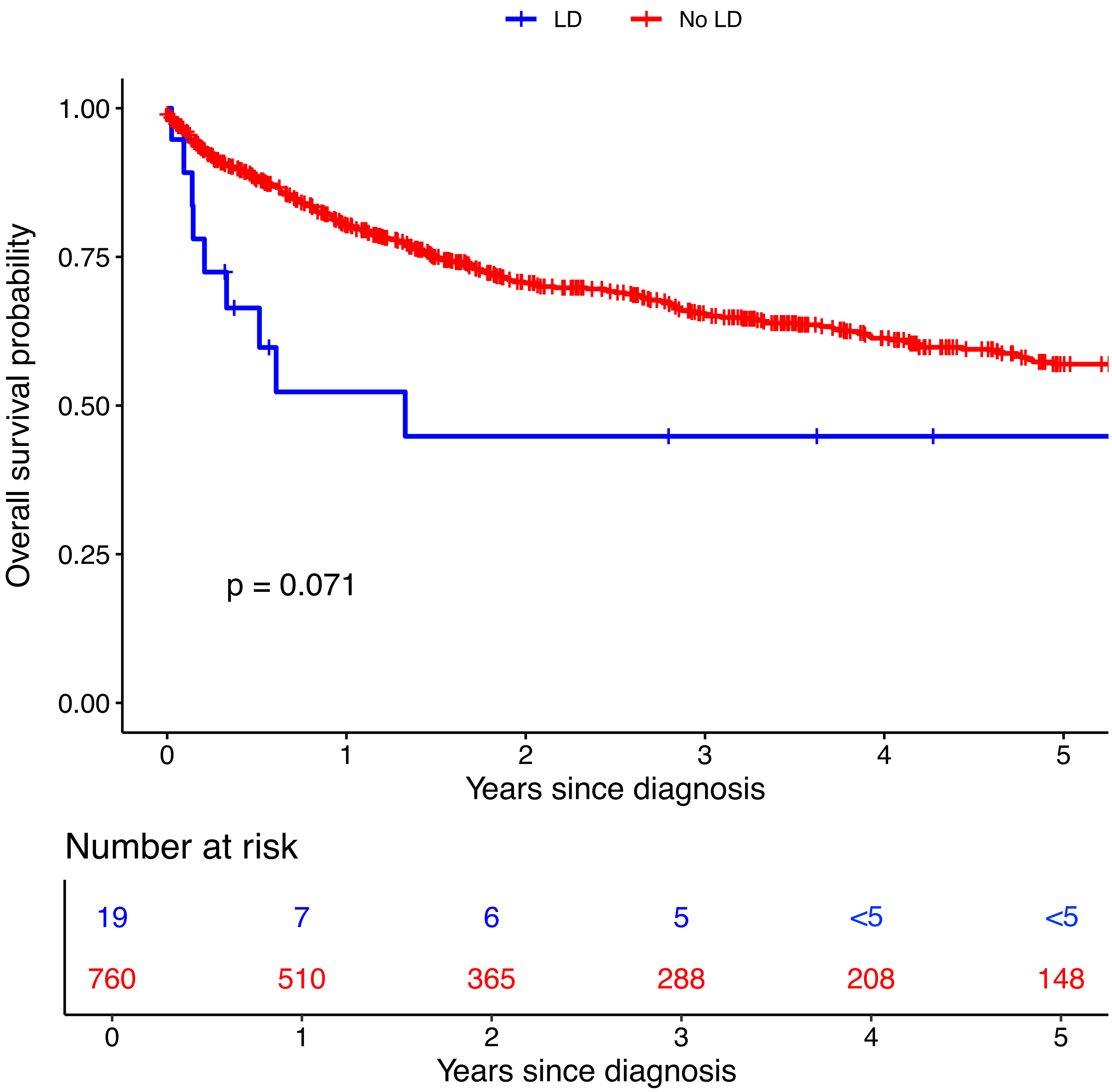

Supplementary figure 23. Overall survival among patients diagnosed with oropharyngeal cancer.

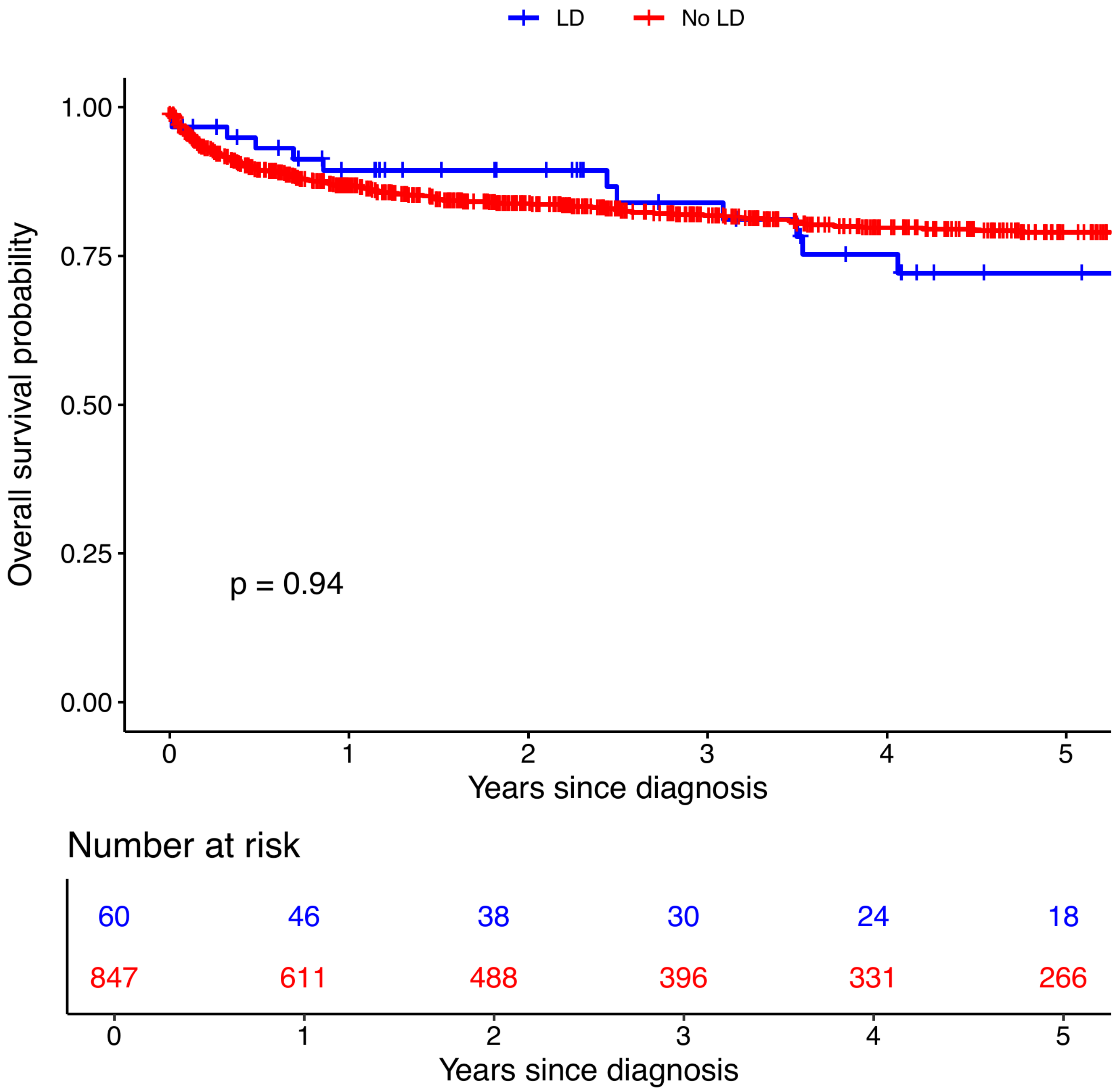

Supplementary figure 24. Overall survival among patients diagnosed with endocrine cancer

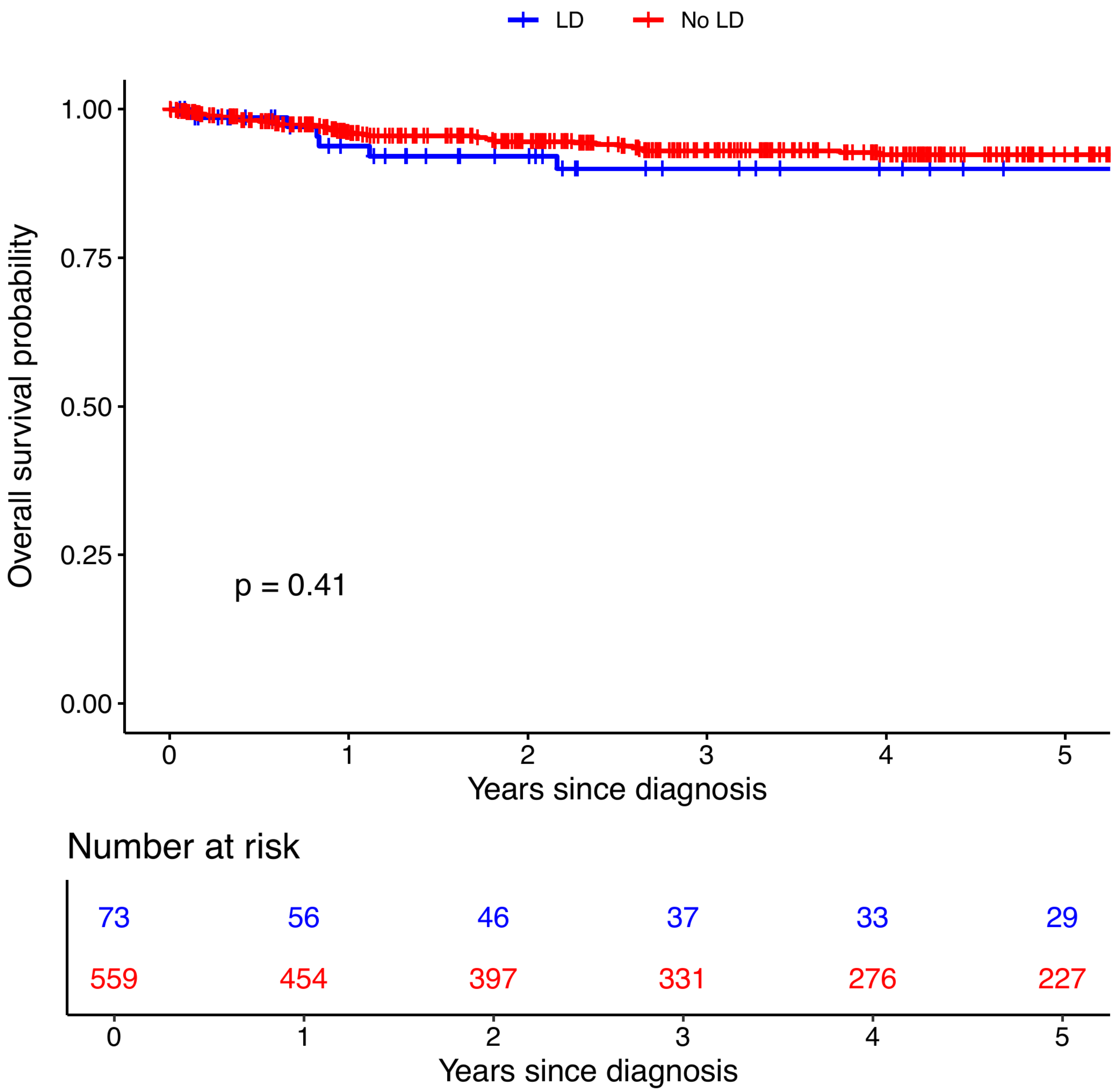

Supplementary figure 25. Overall survival among patients diagnosed with testicular cancer

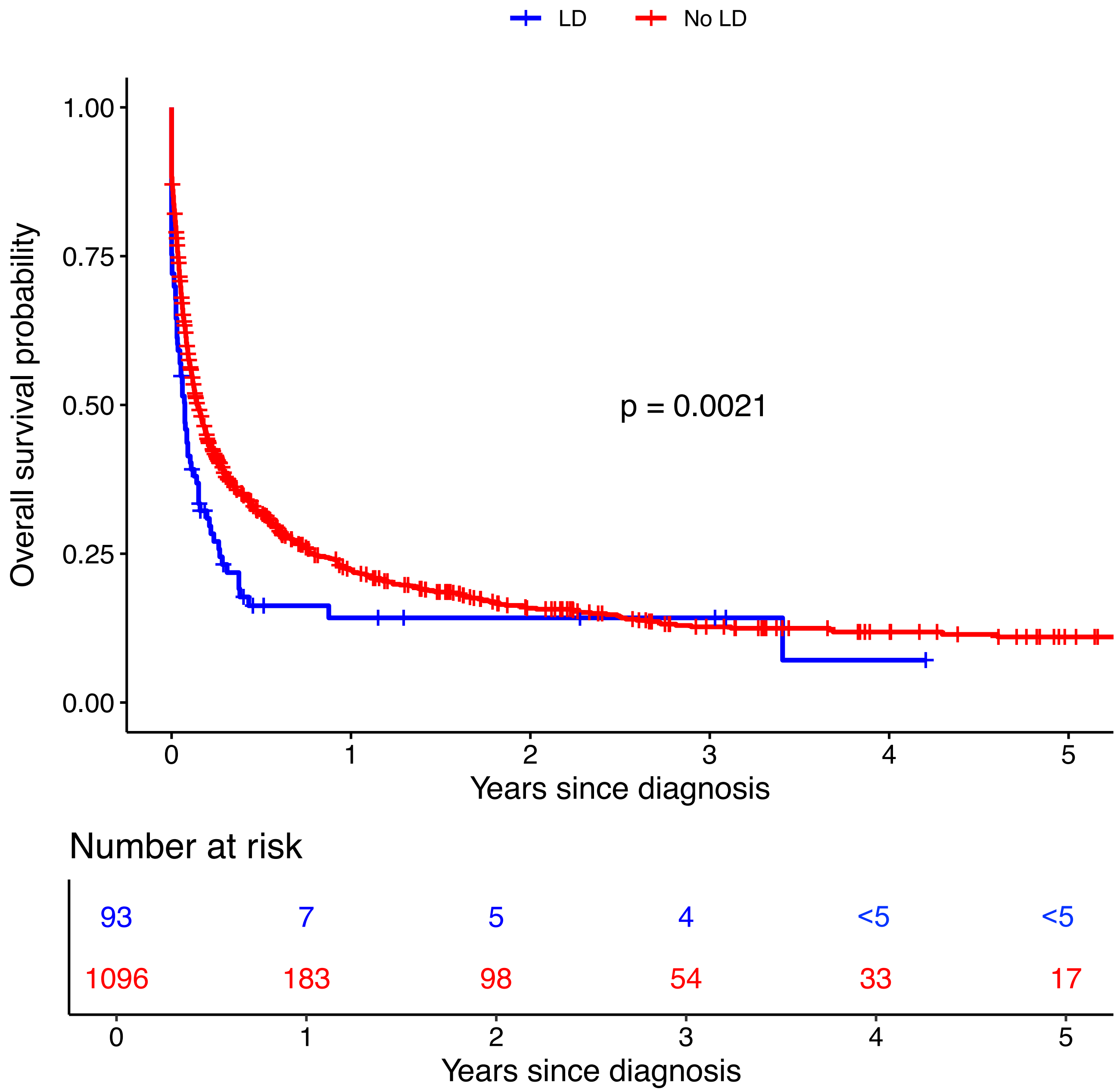

Supplementary figure 26. Overall survival among patients diagnosed with cancer of unknown primary.

Supplementary Code List 1. Codes for the ascertainment of diagnoses of learning disabilities.

MedCodeId	OriginalReadCode	CleansedReadCode	Term	SnomedCTConceptId	SnomedCTDescriptionId
**REGISTER**					
2548475019	918e	918e.00	On learning disability register	416075005	2548475019
**MILD LEARNING DISABILITY**					
296557014	Eu70y	Eu70y00	[X]Mild mental retardation, other impairments of behaviour	86765009	143892017
507246016	E30	E30..00	Mild mental retardation, IQ in range 50-70	86765009	507246016
398381000006119	Eu70	Eu70.00	[X]Mild mental retardation	86765009	507246016
398391000006116	Eu70z	Eu70z00	Mild intellectual disability	86765009	3643513017
398411000006116	Eu70-2	Eu70.12	[X]Mild mental subnormality	86765009	507246016
882771000006119	E30-99	E30..99	Mild mental retardation	86765009	882771000006119
1550041000000110	Eu816	Eu81600	Mild learning disability	984661000000105	2504041000000113
2730391000000116	^ESCT1171812		Mild intellectual development disorder without significant impairment of behaviour	1089831000000105	2730391000000116
2730411000000116	^ESCT1171814		Mild intellectual development disorder with significant impairment of behaviour	1089841000000101	2730411000000116
2730431000000112	^ESCT1171816		Mild intellectual development disorder with minimal impairment of behaviour	1089851000000103	2730431000000112
2740421000000114	^ESCT1171813		Mild mental retardation without significant impairment of behaviour	1089831000000105	2740421000000114
2740451000000116	^ESCT1172258		Mild mental retardation with impairment of behaviour	1093991000000101	2740451000000116
2740461000000118	^ESCT1172257		Mild intellectual development disorder with impairment of behaviour	1093991000000101	2740461000000118
3910901000006110	^ESCTMI391090		Mild mental handicap	86765009	507245017
3910931000006119	^ESCTMI391093		Mild learning disability, intelligence quotient in range 50-70	86765009	536871000000113
3910951000006114	^ESCTMI391095		Mild learning disability	86765009	1666311000000112
12202451000006111	^ESCT1220245		Mild intellectual development disorder	86765009	3654174011
12703781000006112	^ESCT1270378		Mild mental retardation (I.Q. 50-70)	86765009	143892017
**MODERATE LEARNING DISABILITY**					
398811000006118	Eu71	Eu71.00	Moderate intellectual disability	61152003	3643518014
398821000006114	Eu71-1	Eu71.11	[X]Moderate mental subnormality	61152003	3643518014
700071000006118	E310	E310.00	Moderate mental retardation, IQ in range 35-49	61152003	3643518014
882781000006116	E310-99	E310.99	Moderate mental retardation	61152003	882781000006116
1129811000000119	Eu814	Eu81400	Moderate learning disability	984671000000103	2504061000000114
2730291000000112	^ESCT1171804		Moderate intellectual development disorder without significant impairment of behaviour	1089781000000100	2730291000000112
2730311000000113	^ESCT1171806		Moderate intellectual development disorder with significant impairment of behaviour	1089791000000103	2730311000000113
2730351000000112	^ESCT1171808		Moderate intellectual development disorder with minimal impairment of behaviour	1089811000000102	2730351000000112
2730371000000115	^ESCT1171810		Moderate intellectual development disorder with impairment of behaviour	1089821000000108	2730371000000115
3493641000006112	^ESCTMO349364		Moderate mental handicap	61152003	1232179016
3493681000006118	^ESCTMO349368		Moderate learning disability	61152003	1666321000000118
12703791000006110	^ESCT1270379		Moderate mental retardation (I.Q. 35-49)	61152003	101619019
**SEVERE LEARNING DISABILITY**					
296574014	Eu72y	Eu72y00	[X]Severe mental retardation, other impairments of behaviour	40700009	67882016
146051000006113	E311	E311.00	Severe mental retardation, IQ in range 20-34	40700009	3643515012
201751000006110	E312	E312.00	Profound mental retardation with IQ less than 20	31216003	3643527010
423481000006119	Eu731	Eu73100	[X]Profound ment retard sig impairmnt behav req attent/treat	31216003	52225019
423491000006116	Eu730	Eu73000	[X]Profound ment retrd wth statement no or min impairm behav	31216003	52225019
423501000006112	Eu73	Eu73.00	Profound intellectual disability	31216003	3643527010

423511000006110	Eu73y	Eu73y00	[X]Profound mental retardation, other impairments of behavr	31216003	52225019
423521000006119	Eu73-1	Eu73.11	[X]Profound mental subnormality	31216003	3643527010
426591000006111	Eu72	Eu72.00	Severe intellectual disability	40700009	3643515012
426611000006117	Eu72-1	Eu72.11	[X]Severe mental subnormality	40700009	3643515012
882791000006118	E311-99	E311.99	Severe mental retardation	40700009	882791000006118
1129781000000117	Eu815	Eu81500	Severe learning disability	508171000000105	1129801000000116
1550051000000113	Eu817	Eu81700	Profound learning disability	984681000000101	2504081000000117
2730131000000115	^ESCT1171788		Profound intellectual development disorder without impairment of behaviour	1089701000000105	2730131000000115
2730191000000119	^ESCT1171794		Profound intellectual development disorder with impairment of behaviour	1089731000000104	2730191000000119
2730211000000115	^ESCT1171796		Severe intellectual development disorder without significant impairment of behaviour	1089741000000108	2730211000000115
2730231000000111	^ESCT1171798		Severe intellectual development disorder with significant impairment of behaviour	1089751000000106	2730231000000111
2730251000000116	^ESCT1171800		Severe intellectual development disorder with minimal impairment of behaviour	1089761000000109	2730251000000116
2730271000000113	^ESCT1171802		Severe intellectual development disorder with impairment of behaviour	1089771000000102	2730271000000113
2740341000000113	^ESCT1171797		Severe mental retardation without significant impairment of behaviour	1089741000000108	2740341000000113
3003301000006113	^ESCTPR300330		Profound mental handicap	31216003	1227448012
3003361000006114	^ESCTPR300336		Profound learning disability	31216003	1666271000000112
3155151000006112	^ESCTSE315515		Severe learning disability	40700009	2310251000000113
3155171000006119	^ESCTSE315517		Severe mental handicap	40700009	1229669012
12009301000006116	^ESCT1200930		Hypotonia, speech impairment, severe cognitive delay syndrome	763722004	3643495013
12177541000006119	^ESCT1217754		Profound intellectual development disorder	31216003	3654215011
12703921000006110	^ESCT1270392		Profound mental retardation (I.Q. below 20)	31216003	52225019
12703941000006115	^ESCT1270394		Severe mental retardation (I.Q. 20-34)	40700009	67882016
13622001000006116	^ESCT1362200		Severe intellectual disability, progressive spastic diplegia syndrome	782723007	3755494018
15004801000006113	^ESCT1500480		Congenital insensitivity to pain with severe intellectual disability	1237623009	5100092010
**DOWNS SYNDROME**					
378493013	PJ0z	PJ0z.00	Down's syndrome NOS	41040004	598021000000114
378494019	PJ02-1	PJ02.11	Partial trisomy 21 in Down's syndrome	254264002	378494019
1224878018	PJ0z-1	PJ0z.11	Trisomy 21 NOS	41040004	222121000000113
3528203016	^ESCT1167827		Trisomy 21	737542000	3528203016
88351000006114	PJ00	PJ00.00	Trisomy 21, meiotic nondisjunction	205615000	315346017
88361000006111	PJ01-1	PJ01.11	Trisomy 21, mitotic nondisjunction	205616004	315347014
88371000006116	PJ01	PJ01.00	Trisomy 21- mitotic nondisjunction mosaicism	205616004	315347014
88381000006118	PJ02	PJ02.00	Trisomy 21, translocation	254264002	378495018
222121000000113	PJ0-2	PJ0..12	Trisomy 21	41040004	68470016
628281000006114	PJ0	PJ0..00	Down's syndrome	41040004	598021000000114
893481000006117	PJ0-98	PJ0..98	Down's syndrome	41040004	893481000006117
1009531000006118	EMISNQCA42		Cause of learning disabilities: Down's syndrome	1009531000006102	1009531000006118
3161031000006112	^ESCTCO316103		Complete trisomy 21 syndrome	41040004	68470016
3161041000006119	^ESCTDO316104		Down syndrome	41040004	68471017
3161051000006117	^ESCTT2316105		T21 - Trisomy 21	41040004	1229711010
3161061000006115	^ESCTDO316106		Downs syndrome	41040004	2921053011
4830941000006112	^ESCTTR483094		Trisomy 21- meiotic nondisjunction	205615000	315346017
6348371000006113	^ESCTTR634837		Translocation Down syndrome	371045000	1209755010
7955161000006118	^ESCTDE795516		Dementia with Down syndrome	733194007	3498962017
8039101000006116	^ESCTFE803910		Fetal trisomy 21, Down syndrome	125501000119105	3004785019
13944201000006118	^ESCT1394420		Down syndrome co-occurrent with leukaemoid reaction associated transient neonatal pustulosis	840505007	3902179014

OTHER LEARNING DISABILITY

2090010	PJyy4	PJyy400	Fragile X syndrome	613003	2090010
4159010	P21	P21..00	Microcephalus	1829003	4159010
4161018	P211	P211.00	Micrencephaly	1829003	4161018
5179014	P02	P02..00	Iniencephaly	2438005	5179014
9538013	P2280-1	P228011	Agenesis of corpus callosum	5102002	9538013
10374011	PKy1	PKy1.00	Laurence-Moon-Biedl syndrome	5619004	10374011
12877016	PK5	PK5..00	Tuberous sclerosis	7199000	12877016
17479015	ESCTCO12		Coffin-Siris syndrome	10007009	17479015
18114013	C3723	C372300	Lesch-Nyhan syndrome	10406007	18114013
19694018	C375	C375.00	Mucopolysaccharidosis	11380006	19694018
25344018	PKy65	PKy6500	Aarskog syndrome	14921002	25344018
25776014	PKy5F	PKy5F00	Coffin-Lowry syndrome	15182000	25776014
33473011	PK61	PK61.00	Sturge-Weber syndrome	19886006	33473011
35440018	PKy61	PKy6100	Cockayne syndrome	21086008	35440018
36300015	PKy69	PKy6900	Borjeson-Forssman-Lehmann syndrome	21634003	36300015
40055016	F1016	F101600	Sandhoff disease	23849003	40055016
49116010	PG442-1	PG44211	Thanatophoric dysplasia	29352008	49116010
51766016	P225	P225.00	Holoprosencephaly	30915001	51766016
51767013	P224	P224.00	Arhinencephaly	30915001	51767013
53817017	P01	P01..00	Craniorachischisis	32219008	53817017
55063010	PJ335	PJ33500	Greig cephalopolysyndactyly syndrome	32985001	55063010
58597010	PJy2	PJy2.00	XXX syndrome	35111009	1228249015
63896011	PKy60-1	PKy6011	Cornelia de Lange syndrome	40354009	63896011
63898012	PKy60	PKy6000	Amsterdam dwarf	40354009	63898012
68472012	PJ0-1	PJ0..11	Mongolism	41040004	598021000000114
68519010	PJ339	PJ33900	Langer-Giedion syndrome	41069008	68519010
76004014	PKy73-1	PKy7311	Rubinstein-Taybi syndrome	45582004	76004014
81676016	PJ513	PJ51300	Trisomy 4p syndrome	49024004	81676016
94131019	PKy5K	PKy5K00	Cohen syndrome	56604005	94131019
96285014	PKy64	PKy6400	Seckel syndrome	57917004	96285014
97154016	C3272	C327200	Niemann-Pick disease	58459009	97154016
104922018	PKy03	PKy0300	Weaver syndrome	63119004	104922018
108539019	C375-4	C375.14	Lipochondrodystrophy	378007	486814011
108540017	C3751-1	C375111	Gargoylism	75610003	503067012
108541018	C3751-2	C375112	Hurler's syndrome	65327002	108541018
110901011	PG5F	PG5F.00	Acrodysostosis	66758006	110901011
116814019	PJ515	PJ51500	15q partial trisomy syndrome	70324008	116814019
119577010	E141-1	E141.11	Heller's syndrome	71961003	119577010
119579013	E141	E141.00	Disintegrative psychosis	71961003	119579013
121311016	PJ512	PJ51200	10q partial trisomy syndrome	73035005	121311016
121446013	C3751-3	C375113	Scheie's syndrome	73123008	1233590012
121704017	PKy04	PKy0400	Marshall-Smith syndrome	73284007	121704017
126200014	PKyM	PKyM.00	Johanson-Blizzard syndrome	75979009	126200014
127638013	PKyz5-1	PKyz511	Angelman syndrome	76880004	1234038018
127639017	PKyz5	PKyz500	Happy puppet syndrome	76880004	127639017
128696013	PJ514	PJ51400	Trisomy 9p syndrome	77527000	128696013
131704013	C3025-1	C302511	Oculocerebrorenal syndrome	79385002	131704013
137830014	PJ3y0	PJ3y000	Shprintzen syndrome	767263007	3670123013
148214012	PKy0-2	PKy0.12	Prader-Willi syndrome	89392001	148214012
148320010	PJ535	PJ53500	Shwachman-Diamond syndrome	89454001	148320010
151009017	E3	E3...00	Mental retardation	110359009	3643707012
187765019	F101-1	F101.11	Amaurotic familial idiocy	61663001	102454019
215930017	9F8	9F8..00	Statement of special educational needs	134188003	215930017
251223010	13Z4E	13Z4E00	Learning difficulties	161129001	251223010

264621015	6664	6664.00	Mental handicap problem	170695009	264621015
293559017	C372z	C372z00	Other disorder of purine or pyrimidine metabolism NOS	238006008	356736012
293603019	C375z	C375z00	Mucopolysaccharidosis NOS	11380006	19694018
294938012	E1410	E141000	Active disintegrative psychoses	191692007	294938012
294939016	E1411	E141100	Residual disintegrative psychoses	191693002	294939016
294941015	E141z	E141z00	Disintegrative psychosis NOS	71961003	119576018
295622013	E2E1	E2E1.00	Hyperkinesis with developmental delay	192131001	295622013
295633011	E2F2	E2F2.00	Specific learning difficulty	161129001	251223010
295642016	E2F3z	E2F3z00	Speech or language developmental disorder NOS	268672004	401825015
295651012	E2Fy	E2Fy.00	Developmental disorder	5294002	9881017
295652017	E2Fz	E2Fz.00	Developmental disorder NOS	5294002	9881017
295661017	E3y	E3y..00	Other specified mental retardation	110359009	175156010
295662012	E31z	E31z.00	Other specified mental retardation NOS	110359009	175156010
295664013	E3z	E3z..00	Intellectual disability	110359009	175156010
296565012	Eu71y	Eu71y00	[X]Mod retard oth behav impair	61152003	101619019
296586012	Eu7y	Eu7y.00	[X]Other mental retardation	110359009	175156010
296592018	Eu7yy	Eu7yy00	[X]Other mental retardation, other impairments of behaviour	110359009	175156010
296643017	Eu81	Eu81.00	[X]Specific developmental disorders of scholastic skills	1855002	4203013
296657015	Eu81z	Eu81z00	Developmental disorder of scholastic skill	1855002	478661012
296663012	Eu83	Eu83.00	Mixed developmental disorder	442059001	2819191012
296683013	Eu8z	Eu8z.00	Disorder of psychological development	192562009	296607011
296991018	F1021	F102100	Cerebral degeneration in Niemann-Pick disease	192792002	296991018
296995010	F1031	F103100	Cerebral degeneration in mucopolysaccharidoses	192796004	296995010
312368018	E2F	E2F..00	Specific delays in development	10720004	312368018
312877018	P21z	P21z.00	Microcephalus NOS	1829003	4159010
312878011	P22	P22..00	Reduction deformities of brain	204032005	312878011
312902012	P22y	P22y.00	Other specified reduction deformities of brain	204032005	312878011
312905014	P22yz	P22yz00	Other reduction deformity of brain NOS	204032005	312878011
314772015	PF550	PF55000	Acrocephalosyndactyly (Apert)	205258009	314772015
314773013	PF551	PF55100	Acrocephalosyndactyly type V	70410008	2816530018
315143015	PG5D	PG5D.00	Craniodiaphyseal dysplasia	205506004	315143015
315350012	PJ10	PJ10.00	Trisomy 13, meiotic nondisjunction	205619006	315350012
315359013	PJ3	PJ3..00	Monosomies and deletions from the autosomes	205627002	315359013
315360015	PJ30	PJ30.00	Antimongolism syndrome	254274004	378508010
315361016	PJ33	PJ33.00	Other deletions of part of a chromosome	254274004	378508010
315362011	PJ330	PJ33000	Deletion of long arm of chromosome 13	205630009	315362011
315369019	PJ33z	PJ33z00	Other deletion of part of a chromosome NOS	254274004	378508010
315371019	PJ35	PJ35.00	Deletions with other complex rearrangements	274908005	410807013
315377015	PJ370	PJ37000	Monosomy 21, mosaicism	205638002	315377015
315378013	PJ37z	PJ37z00	Whole chromosome monosomy, mosaicism NOS	270520003	405158019
315383017	PJ3y	PJ3y.00	Other deletions from the autosomes	254274004	378508010
315385012	PJ3z	PJ3z.00	Monosomies and deletions from the autosomes NOS	254274004	378508010
315387016	PJ5	PJ5..00	Other condition due to autosomal anomaly	74345006	123461015
315389018	PJ500	PJ50000	Trisomy 6	205647005	315389018
315390010	PJ501	PJ50100	Trisomy 7	205648000	315390010
315391014	PJ502	PJ50200	Trisomy 8	205649008	315391014
315392019	PJ503	PJ50300	Trisomy 9	205650008	315392019
315393012	PJ504	PJ50400	Trisomy 10	205651007	315393012
315394018	PJ505	PJ50500	Trisomy 11	205652000	315394018
315395017	PJ506	PJ50600	Trisomy 12	205653005	315395017
315396016	PJ507	PJ50700	Other trisomy C syndromes	270521004	405160017
315397013	PJ508	PJ50800	Trisomy 22	205655003	315397013
315398015	PJ50w	PJ50w00	Whole chromosome trisomy meiotic nondisjunction	254269007	3661470014
315399011	PJ50x	PJ50x00	Whole chromosome trisomy, mosaicism	205657006	315399011
315401017	PJ50y	PJ50y00	Other specified whole chromosome trisomy syndrome	270521004	405160017
315402012	PJ50z	PJ50z00	Whole chromosome trisomy syndrome NOS	270521004	405160017

315403019	PJ51	PJ51.00	Partial trisomy syndromes	205660004	315403019
315404013	PJ510	PJ51000	Major partial trisomy	205661000	315404013
315405014	PJ511	PJ51100	Minor partial trisomy	205662007	315405014
315406010	PJ51z	PJ51z00	Partial trisomy syndrome NOS	205660004	315403019
315407018	PJ52	PJ52.00	Trisomies of autosomes NEC	270521004	405160017
315410013	PJ522	PJ52200	Extra marker chromosomes	444655009	2870837017
315413010	PJ52z	PJ52z00	Trisomy and partial trisomy of autosome	270521004	405160017
315418018	PJ531	PJ53100	Balanced autosomal rearrangement in abnormal individual	205673000	315418018
315419014	PJ532	PJ53200	Balanced sex/autosomal rearrangement in abnormal individual	205674006	315419014
315420015	PJ533	PJ53300	Individual with marker heterochromatin	205675007	315420015
315421016	PJ534	PJ53400	Individual with autosomal fragile site	205676008	315421016
315474014	PJy13	PJy1300	Mosaic including XXXXY	205710004	315474014
315486012	PJyy2	PJyy200	Fragile X chromosome	205720009	315486012
315493011	PJz2	PJz2.00	Deletion of part of autosome	254274004	378508010
315571015	Q0071-1	Q007111	Fetal alcohol syndrome	205788004	315571015
315607013	PKy70	PKy7000	Carpenter syndrome	403767009	3494870015
315625018	PKy80	PKy8000	Noonan's syndrome	205824006	315625018
315655013	Pyu02	Pyu0200	[X]Other reduction deformities of brain	204032005	312878011
315656014	Pyu03	Pyu0300	[X]Other specified congenital malformations of brain	88425004	146609011
315799014	PyuA1	PyuA100	[X]Other deletions of part of a chromosome	254274004	378508010
315800013	PyuA2	PyuA200	[X]Other deletions from the autosomes	205627002	315358017
329968011	C03z-2	C03z.12	Cretinism	217710005	329968011
342159015	13ZK	13ZK.00	Child with special educational needs	228141003	342159015
342177013	13VCA		Intellectual functioning disability	228156007	342177013
345138018	F1306	F130600	Aicardi Goutieres syndrome	230312006	345138018
345281011	F2505	F250500	Lennox-Gastaut syndrome	230418006	345281011
347021018	E2F3-1	E2F3.11	Developmental language disorder	280032002	417527019
377050018	P2402	P240200	Schizencephaly	253159001	377050018
377071011	P22y2	P22y200	Gillespie syndrome	253176002	377071011
378221016	C375-A	C375.1A	Dysostosis multiplex	254069004	378221016
378490011	PJ5z-1	PJ5z.11	Aneuploidy NEC	74345006	200444011
378491010	PJ5y-1	PJ5y.11	Pseudotrisomy 18	254261005	378491010
378496017	PJ2z	PJ2z.00	Edward's syndrome NOS	51500006	85775018
378497014	PJ22-1	PJ22.11	Partial trisomy 18 in Edward's syndrome	254266000	378497014
378499012	PJ1z	PJ1z.00	Patau's syndrome NOS	21111006	35482019
378500015	PJ12-1	PJ12.11	Partial trisomy 13 in Patau's syndrome	254268004	378500015
378507017	PJ37-2	PJ37.12	Autosomal deletion - mosaicism	254273005	378507017
398888012	C372	C372.00	Disorder of purine and pyrimidine metabolism	238006008	356736012
400767012	P22z	P22z.00	Reduction deformities of brain NOS	204032005	312878011
400910012	PF55	PF55.00	Acrocephalosyndactyly	268262006	400910012
400911011	PF55-1	PF55.11	Apert's syndrome	268262006	400911011
400957013	PJ38	PJ38.00	Chromosome replaced with ring or dicentric	268294001	400957013
400959011	PJ5y	PJ5y.00	Other specified conditions due to autosomal anomalies	74345006	123461015
400960018	PJ5z	PJ5z.00	Unspecified conditions due to autosomal anomalies	74345006	123461015
400966012	PKy0	PKy0.00	Multiple system congenital anomalies NEC	66091009	109766011
401827011	E2F30	E2F3000	Developmental aphasia	268673009	401827011
401902015	Eu7z	Eu7z.00	[X]Unspecified mental retardation	110359009	175156010
401906017	Eu80y	Eu80y00	[X]Other developmental disorders of speech and language	268672004	401825015
401909012	Eu81y	Eu81y00	[X]Other developmental disorders of scholastic skills	1855002	4203013
401910019	Eu843	Eu84300	[X]Other childhood disintegrative disorder	35919005	59939011
401911015	Eu8y	Eu8y.00	[X]Other disorders of psychological development	5294002	9881017
405159010	PJ37	PJ37.00	Whole chromosome monosomy, mosaicism	270520003	405159010
405346013	PJ331	PJ33100	Deletion of long arm of chromosome 18	270889005	405346013
405347016	PJ332-1	PJ33211	18q- syndrome	270889005	405347016
405348014	PJ331-1	PJ33111	18p- syndrome	270890001	405348014
405349018	PJ332	PJ33200	Deletion of short arm of chromosome 18	270890001	405349018

413177014	E30-1	E30..11	Educationally subnormal	276854003	413177014
415431010	C031	C031.00	Goitrous cretin	278503003	415431010
455888019	PKy68	PKy6800	Floating-Harbor syndrome	312214005	455888019
456373012	8O07	8O07.00	Provision of special educational needs nursery	312621002	456373012
460157017	8O5	8O5..00	Special needs support	315638005	460157017
460622019	ZV400	ZV40000	[V]Problems with learning	161129001	251223010
497080018	C3758	C375800	Multiple sulphatase deficiency	54898003	497080018
500490013	PJ523	PJ52300	Triploidy	66651005	500490013
502310015	PJ524	PJ52400	Polyploidy	72991005	502310015
502891013	C0A	C0A..00	Congenital iodine deficiency syndrome	75065003	502891013
1221474011	PJ32	PJ32.00	Deletion of short arm of chromosome 4	17122004	1221474011
1222495016	R034y-1	R034y11	[D]Global retardation	224958001	338115019
1224879014	PJ2z-1	PJ2z.11	Trisomy 18 NOS	51500006	85775018
1224880012	PJ1z-1	PJ1z.11	Trisomy 13 NOS	21111006	35482019
1224941015	PKy66	PKy6600	Dubowitz syndrome	2593002	1224941015
1226349010	PG442	PG44200	Thanatophoric dwarfism	29352008	1226349010
1228248011	PJy2-1	PJy2.11	Triple X female	35111009	1228248011
1228249015	PJy2-2	PJy2.12	Karyotype 47, XXX	35111009	1228249015
1229637015	PKy60-3	PKy6013	Degenerative amsterodamensis typus	40354009	1229637015
1229639017	PKy60-2	PKy6012	Bruck-de Lange syndrome	40354009	1229639017
1231577014	PKy1-1	PKy1.11	Biedl-Bardet syndrome	5619004	1231577014
1232445010	PKy4	PKy4.00	William syndrome	63247009	1232445010
1232670018	C3082	C308200	X-linked adrenoleucodystrophy	65389002	1232670018
1233229018	PJ31-1	PJ31.11	Deletion of short arm of chromosome 5	70173007	1233229018
1233939016	C1zy2	C1zy200	Cerebral gigantism	75968004	1233939016
1233940019	C1zy2-1	C1zy211	Sotos syndrome	75968004	1233940019
1234038018	PKyz7	PKyz700	Angelman's syndrome	76880004	1234038018
1234349019	C3025-2	C302512	Oculocerebrorenal dystrophy	79385002	1234349019
1234352010	C3025	C302500	Lowe disease	79385002	1234352010
1234503010	P2283	P228300	Aicardi syndrome	80651009	1234503010
1234786015	PJ3y0-1	PJ3y011	Velocardiofacial syndrome	767263007	3670117015
1235347013	PKy94	PKy9400	Zellweger's syndrome	88469006	1235347013
1235773018	B927-2	B927.12	Neurofibromatosis type 1	92824003	1235773018
1488521019	9bA0	9bA0.00	Mental handicap (specialty)	394815005	1488521019
1491711019	Eu800-2	Eu80012	[X]Developmental speech articulation disorder	386701004	1491709011
1494665012	PyuAB	PyuAB00	Pallister-Killian syndrome	9527009	3010792013
1780502018	PJ333	PJ33300	Smith-Magenis syndrome	401315004	1780502018
1786608015	1P01	1P01.00	Psychomotor retardation	398991009	1786608015
2159902018	1JB0	1JB0.00	Suspected Downs syndrome	408338009	2159902018
2478440016	PKyG-1	PKyG.11	Ohdo blepharophimosis syndrome	412787009	2478440016
2900005013	F1y0	F1y0.00	Fragile X associated tremor ataxia syndrome	448045004	2900005013
2995031014	PKy06	PKy0600	Feingold syndrome	702431004	2995031014
3505252012	^ESCT1164321		Alpha-thalassaemia intellectual disability syndrome linked to chromosome 16	734349003	3505252012
3636386011	^ESCT1168995		Developmental language disorder and impairment of receptive and expressive language	762317007	3636386011
3636387019	^ESCT1168996		Developmental language disorder co-occurrent with impairment of receptive and expressive language	762317007	3636387019
3636390013	^ESCT1168997		Developmental language disorder and impairment of expressive language	762318002	3636390013
3636779018	^ESCT1169234		Developmental language disorder co-occurrent with language impairment	762502009	3636779018
3636780015	^ESCT1169233		Developmental language disorder and language impairment	762502009	3636780015
9881000006115	E31	E31..00	Other specified mental retardation	110359009	175156010
53891000006113	PJ32-1	PJ32.11	Wolff - Hirschorn syndrome	17122004	1221473017
60491000006112	PJ36	PJ36.00	Whole chromosome monosomy, meiotic nondisjunction	205636003	315372014

60541000006119	PJ50	PJ50.00	Whole chromosome trisomy syndromes	205646001	315388014
63811000006114	B927-1	B927.11	Von Recklinghausen's disease	92824003	1235773018
79051000006114	PKyz0	PKyz000	Ullrich - Feichtiger syndrome, chimaera	21111006	35482019
88251000006115	PJ11-1	PJ11.11	Trisomy 13, mitotic nondisjunction	205620000	315351011
88261000006118	PJ11	PJ11.00	Trisomy 13 - mitotic nondisjunction mosaicism	205620000	315351011
88271000006113	PJ12	PJ12.00	Trisomy 13, translocation	254268004	378501016
88291000006114	PJ20	PJ20.00	Trisomy 18, meiotic nondisjunction	205623003	315354015
88301000006110	PJ21-1	PJ21.11	Trisomy 18, mitotic nondisjunction	205624009	315355019
88311000006113	PJ21	PJ21.00	Trisomy 18 - mitotic nondisjunction mosaicism	205624009	315355019
88321000006117	PJ22	PJ22.00	Trisomy 18, translocation	254266000	378498016
88391000006115	PJ0-3	PJ0..13	Trisomy 22	205655003	315397013
107661000006110	F1013	F101300	Tay-Sach's disease	192787004	296986019
133861000006111	E2F3-2	E2F3.12	Developmental speech disorder	1145003	2999013
133891000006115	E2F3	E2F3.00	Disorder of speech and language development	268672004	401825015
137341000006114	PKy63	PKy6300	Smith - Lemli - Opitz syndrome	43929004	73253014
154901000006117	C375-9	C375.19	Scheie's syndrome	73123008	1233590012
158401000006118	PKy73	PKy7300	Rubenstein - Tayi syndrome	45582004	76004014
212911000006116	PKy93	PKy9300	Prader-Willi syndrome	89392001	148214012
212921000006112	PKy0-1	PKy0.11	Prader-Willi Syndrome	89392001	148214012
215821000000119	E30-3	E30..13	Moron	86765009	507246016
222131000000110	PJ71-1	PJ71.11	Klinefelter's syndrome, XXXY	275263003	411223014
222141000000118	PJ71-2	PJ71.12	Klinefelter's syndrome, XXXXY	275264009	411224015
223441000000119	C3720-1	C372011	Lesch - Nyhan syndrome	10406007	18114013
239991000006111	PJ1	PJ1..00	Patau syndrome	21111006	35483012
253231000006116	F2511-1	F251111	Otohara syndrome	192990004	297280011
297751000006119	PKy0-3	PKy0.13	Noonan's syndrome	205824006	315625018
301521000000111	Eu804	Eu80400	[X]Cocktail party syndrome	268672004	401825015
302051000000118	PKyJ	PKyJ.00	Lujan-Fryns syndrome	422437002	2649528010
362161000006110	Eu803	Eu80300	Acquired epileptic aphasia	230438007	345312010
371571000006114	Eu802-1	Eu80211	[X]Congenital auditory imperception	229748008	344387017
376631000006115	Eu843-1	Eu84311	[X]Dementia infantalis	71961003	119576018
376831000006116	Eu8y-1	Eu8y.11	[X]Developmental agnosia	5294002	9881017
376841000006114	Eu801-2	Eu80112	[X]Developmental aphasia, expressive type	268734000	401904019
376851000006111	Eu802-5	Eu80215	Receptive language delay	229736005	344370019
376891000006117	Eu80z	Eu80z00	[X]Developmental disorder of speech and language unspecified	268672004	401825015
376911000006115	Eu801-1	Eu80111	Developmental expressive language disorder	268734000	401904019
376921000006111	Eu802-2	Eu80212	[X]Developmental dysphasia, receptive type	268673009	401827011
376951000006119	Eu812-3	Eu81213	[X]Developmental Gerstmann's syndrome	229676007	344282013
376961000006117	Eu800-1	Eu80011	Developmental speech articulation disorder	386701004	1491709011
376981000006110	Eu802-3	Eu80213	[X]Developmental Wernicke's aphasia	367515004	492553013
377461000006113	Eu843-2	Eu84312	[X]Disintegrative psychosis	35919005	59939011
377921000006114	Eu8	Eu8..00	[X]Disorders of psychological development	192562009	296607011
386751000006111	Eu70-1	Eu70.11	[X]Feeble-mindedness	86765009	507246016
388761000006114	Eu843-3	Eu84313	[X]Heller's syndrome	71961003	119577010
394581000006118	Eu840-4	Eu84014	[X]Kanner's syndrome	408856003	2477201014
394991000006113	Eu80z-1	Eu80z11	[X]Language development disorder NOS	268672004	401825015
395051000006119	Eu81z-3	Eu81z13	[X]Learn acquisition disab NOS	110359009	3643707012
395061000006117	Eu81z-1	Eu81z11	Learning disability	1855002	478664016
395071000006112	Eu81z-2	Eu81z12	[X]Learning disorder NOS	1855002	478661012
398201000006115	Eu7z-1	Eu7z.11	[X]Mental deficiency NOS	110359009	175156010
398231000006111	Eu7	Eu7..00	[X]Mental retardation	110359009	3643707012
398241000006118	Eu841-2	Eu84112	[X]Mental retardation with autistic features	231536004	347022013
398251000006116	Eu7z-2	Eu7z.12	[X]Mental subnormality NOS	110359009	175156010
398591000006113	Eu813	Eu81300	Mixed disorder of scholastic skills	192575009	296652014
398651000006118	Eu701	Eu70100	[X]Mld mental retard sig impairment behav req attent/treatmt	86765009	143892017
398661000006116	Eu700	Eu70000	[X]Mld mental retard with statement no or min impairm behav	86765009	143892017

398761000006113	Eu711	Eu71100	[X]Mod mental retard sig impairment behav req attent/treatmt	61152003	101619019
398771000006118	Eu710	Eu71000	[X]Mod mental retard with statement no or min impairm behav	61152003	101619019
398781000006115	Eu71z	Eu71z00	[X]Mod mental retardation without mention impairment behav	61152003	3643518014
404851000006117	Eu7y1	Eu7y100	[X]Oth mental retard sig impairment behav req attent/treatmt	110359009	175156010
404861000006115	Eu7y0	Eu7y000	[X]Oth mental retard with statement no or min impairm behav	110359009	175156010
405381000006115	PyuA0	PyuA000	[X]Oth specif trisomies & partial trisomies of autosomes	270521004	405160017
411791000006118	Eu7yz	Eu7yz00	[X]Other mental retardation without mention impairment behav	110359009	175156010
417681000006116	Eu844	Eu84400	[X]Overactive disorder assoc mental retard/stereotype movts	35919005	59939011
423361000006118	Eu73z	Eu73z00	[X]Prfnd mental retardation without mention impairment behav	31216003	52225019
424181000006111	Eu8z-1	Eu8z.11	[X]Psychological developmental disorder NOS	192562009	296607011
424971000006111	Eu842	Eu84200	Rett syndrome	68618008	113977013
426521000006114	Eu721	Eu72100	[X]Sev mental retard sig impairment behav req attent/treatmt	40700009	67882016
426531000006112	Eu720	Eu72000	[X]Sev mental retard with statement no or min impairm behav	40700009	67882016
426541000006119	Eu72z	Eu72z00	[X]Sev mental retardation without mention impairment behav	40700009	3643515012
427261000006119	Eu80	Eu80.00	[X]Specific developmental disorders of speech and language	268672004	401825015
430061000006113	Eu7z0	Eu7z000	[X]Unsp mental retard with statement no or min impairm behav	110359009	175156010
430071000006118	Eu7zz	Eu7zz00	[X]Unsp mental retardation without mention impairment behav	110359009	175156010
430081000006115	Eu7z1	Eu7z100	[X]Unsp mentl retard sig impairment behav req attent/treatmt	110359009	175156010
431231000006115	Eu7zy	Eu7zy00	[X]Unspecified mental retardatn, other impairments of behav	110359009	175156010
539561000006115	PG421-1	PG42111	Catel-Schwartz-Jampel syndrome	29145002	48771010
553151000006114	PJ38-1	PJ38.11	Chromosome replaced with dicentric	268294001	400957013
553161000006111	PJ38-2	PJ38.12	Chromosome replaced with ring	268294001	400957013
557191000000119	PJ9	PJ9..00	Mowat-Wilson syndrome	703535000	3009119013
571541000006116	PKyz-1	PKyz.11	Cockayne's syndrome	21086008	35440018
583291000006115	C0A1	C0A1.00	Congenital iodine-deficiency syndrome, myxoedematous type	75065003	502897012
583301000006119	C0A0	C0A0.00	Congenital iodine-deficiency syndrome, neurological type	237566004	356024011
596611000000111	R034A	R034A00	[D]Communication skills development delay	274625009	410457011
600251000000118	PJ334	PJ33400	Jacobsen syndrome	715438008	3302630014
600791000006112	C03-1	C03..11	Cretinism	217710005	329968011
600811000006111	PJ31	PJ31.00	Cri du chat syndrome	70173007	116547017
610871000006117	C3770	C377000	Defects in post-translational modif'n of lysosomal enzymes	190948002	293609015
612441000006114	PJ30-1	PJ30.11	Deletion of long arm of chromosome 21	254274004	378508010
612481000006115	PJ34	PJ34.00	Deletions seen only at prometaphase	205634000	315370018
616061000006115	C3911	C391100	DiGeorge syndrome	767263007	3670122015
623961000006117	C375X	C375X00	Disorder of glucosaminoglycan metabolism, unspecified	238043005	356812017
623971000006112	C31yX	C31yX00	Disorder of glycoprotein metabolism, unspecified	238045003	356821016
624501000006112	C377	C377.00	Disorder of glycoprotein metabolism	238045003	356821016
632481000006113	PJ520	PJ52000	Duplications seen only at prometaphase	205665009	315408011
632491000006111	PJ521	PJ52100	Duplications with other complex rearrangements	205666005	315409015
636711000006113	PJ2	PJ2..00	Edward's syndrome - trisomy 18	51500006	85776017
667621000006119	E30-2	E30..12	Feeble-minded	86765009	507246016
669211000006118	PK80	PK80.00	Fetal alcohol syndrome	205788004	315571015
676511000006115	B927	B927.00	Neurofibromatosis - Von Recklinghausen's disease	92824003	1235773018
682861000006114	PG421	PG42100	Schwartz-Jampel syndrome	29145002	48771010
696681000006115	C3751	C375100	Mucopolysaccharidosis, type 1	75610003	503067012
696691000006117	C3752	C375200	Mucopolysaccharidosis type II	70737009	501652016
696701000006117	C3753	C375300	Mucopolysaccharidosis type III	88393000	1235342019
696711000006119	C3754	C375400	Mucopolysaccharidosis type IV	378007	486815012
696721000006110	C3756	C375600	Mucopolysaccharidosis, type VI	69463008	501281010
696731000006113	C3757	C375700	Mucopolysaccharidosis type VII	43916004	493711018
698811000006110	PKy92	PKy9200	Menkes syndrome	59178007	198877015
701121000006111	E2F5	E2F5.00	Mixed disorder of psychological development	192147004	295650013
733951000006111	13Z3	13Z3.00	Intelligence quotient low	102942005	256780013
747231000006115	C372-1	C372.11	Lesch - Nyhan syndrome	10406007	18114013
754291000006111	PKy92-1	PKy9211	Kinky hair syndrome	59178007	498370014
785941000006115	E310-1	E310.11	Imbecile	61152003	3643518014

787151000006113	E312-1	E312.11	Idiocy	31216003	3643527010
787441000006118	C3720	C372000	Hypoxanthine-guanine-phosphoribosyltransferase deficiency	124275001	203921018
798681000006113	C375-1	C375.11	Gargoylism	75610003	503067012
803251000006114	E2F5-1	E2F5.11	Global developmental delay	224958001	338115019
829441000006116	C375-3	C375.13	Hurler's syndrome	65327002	108541018
831231000006112	EMISQPS1		Psychomotor retardation	831231000006108	831231000006112
855571000006112	EGTONCH5		Child language development delayed	855571000006108	855571000006112
855591000006113	EGTONCH6		Child language development abnormal	855591000006109	855591000006113
881231000006117	B927-99	B927.99	Neurofibromatosis	92824003	881231000006117
882741000006110	E2Fz-99	E2Fz.99	Development delay - NOS	686491000000105	882741000006110
882761000006114	E3-99	E3...99	Mental subnormality	91138005	882761000006114
882801000006117	E31z-99	E31z.99	Mental subnormality NOS	686511000000102	882801000006117
893501000006110	PJyy2-99	PJyy299	Fragile X syndrome	205720009	893501000006110
905641000006118	HNG0038		[RFC] Development delay	905641000006102	905641000006118
906941000006119	HNG0150		[RFC] Learning disabilities	906941000006103	906941000006119
907741000006115	HNG0250		[RFC] Specific learning problems	907741000006104	907741000006115
908591000006117	HNG0517		[RFC] General development delay	908591000006101	908591000006117
908941000006118	HNG0625		[RFC] Learning disability	908941000006102	908941000006118
909931000006115	HNGNQRF38		[RFC] Special needs	909931000006104	909931000006115
909941000006113	HNGNQRF39		[RFC] Communication special needs	909941000006109	909941000006113
909951000006110	HNGNQRF40		[RFC] Learning special needs	909951000006106	909951000006110
923881000006119	PCNQGE2		General development delay	923881000006103	923881000006119
940371000006118	EMISNQFR2		Fragile X syndrome	940371000006102	940371000006118
968201000006114	PKyG	PKyG.00	Mental retardation, congenital heart disease, blepharophimosis, blepharoptosis and hypoplastic teeth	412787009	2474343011
988941000006119	E3-98	E3...98	Mental subnormality NOS	91138005	988941000006119
1009521000006116	EMISNQCA41		Cause of learning disabilities	1009521000006100	1009521000006116
1009541000006111	EMISNQCA43		Cause of learning disabilities: Tuberous sclerosis	1009541000006107	1009541000006111
1009551000006113	EMISNQCA44		Cause of learning disabilities: Birth trauma	1009551000006109	1009551000006113
1009561000006110	EMISNQCA45		Cause of learning disabilities: Meningitis/encephalitis	1009561000006106	1009561000006110
1009571000006115	EMISNQCA46		Cause of learning disabilities: Fragile X syndrome	1009571000006104	1009571000006115
1009581000006117	EMISNQCA47		Cause of learning disabilities: Late effect of head injury	1009581000006101	1009581000006117
1009591000006119	EMISNQCA48		Cause of learning disabilities: Brain tumour	1009591000006103	1009591000006119
1009601000006110	EMISNQCA49		Cause of learning disabilities: Congenital hydrocephalus	1009601000006106	1009601000006110
1009611000006113	EMISNQCA50		Cause of learning disabilities: Microcephaly	1009611000006109	1009611000006113
1009621000006117	EMISNQCA51		Cause of learning disabilities: Phenylketonuria	1009621000006101	1009621000006117
1009631000006119	EMISNQCA52		Cause of learning disabilities: Prader-Willi syndrome	1009631000006103	1009631000006119
1009641000006112	EMISNQCA53		Cause of learning disabilities: Smith-Magenis syndrome	1009641000006108	1009641000006112
1009651000006114	EMISNQCA54		Cause of learning disabilities: Rett syndrome	1009651000006105	1009651000006114
1009661000006111	EMISNQCA55		Cause of learning disabilities: Congenital rubella	1009661000006107	1009661000006111
1009671000006116	EMISNQCA56		Cause of learning disablts: Unknown/awaiting investigation	1009671000006100	1009671000006116
1009681000006118	EMISNQCA57		Cause of learning disabilities: Unknown/despite investigation	1009681000006102	1009681000006118
1009691000006115	EMISNQCA58		Cause of learning disabilities: Other	1009691000006104	1009691000006115
1563991000006112	EMISNQLE3		Learning disability - specialty	1563991000006108	1563991000006112
1620441000006116	EMISNQLE5		Learning disability	1620441000006100	1620441000006116
1667711000000114	Eu86	Eu86.00	[X]Neurodevelopmental delay	751391000000106	1667711000000114
1667721000000115	Eu85	Eu85.00	[X]Global developmental delay	224958001	1667721000000115
1694831000006112	EMISNQPI5		Pitt-Hopkins syndrome	702344008	2995603016
1705901000006118	PKyz7-1	PKyz711	Angelman syndrome	76880004	1234038018
1786301000006110	PJ331-2	PJ33112	18q- syndrome	270889005	405347016
1786311000006113	PJ332-2	PJ33212	18p- syndrome	270890001	405348014
1786391000006115	PJ512-1	PJ51211	Duplication 10q syndrome	73035005	121311016
1786411000006115	PJ513-1	PJ51311	4p partial trisomy syndrome	49024004	81675017
1786421000006111	PJ514-1	PJ51411	9p partial trisomy syndrome	77527000	128694011
1786441000006116	PJ515-1	PJ51511	Duplication 15q syndrome	70324008	116814019

1786521000006110	PKyB-2	PKyB.12	Coloboma, heart malformation, choanal atresia, retardation of growth and development, genital abnormalities, and ear malformations (CHARGE) association	47535005	2620800011
1786571000006111	R034E	R034E00	Developmental delay	248290002	370667011
1798121000000114	P3423	P342300	Peters plus syndrome	449817000	2912534012
1798581000000119	PJ336	PJ33600	Chromosome 22q11 deletion syndrome	449818005	2912736012
1798621000000119	PJ338	PJ33800	Chromosome 4q deletion syndrome	37506004	2912992019
1800151000000118	PKyN	PKyN.00	Marden Walker syndrome	449824004	2912587012
1821361000006110	EMISNQIN133		Intellectual functioning disability	228156007	1821361000006110
1823961000006113	EMISNQLE13		Learning disability confirmed	1823961000006109	1823961000006113
1825141000006111	EMISNQAD62		Adrenoleucodystrophy	65389002	2154288010
1840501000006110	EMISNQSP29		Special educational needs - moderate learning difficulties	1840501000006106	1840501000006110
1840531000006119	EMISNQSP30		Special educ need-complex learning difficulties and disabilities	1840531000006103	1840531000006119
1872711000006116	EMISNQSP32		Special educational need type	1872711000006100	1872711000006116
1872721000006112	EMISNQSP33		Special education needs - specific learning disability	1872721000006108	1872721000006112
1872731000006110	EMISNQSP34		Special education needs - learning difficulty	1872731000006106	1872731000006110
1887331000006119	Eu818	Eu81800	Specific learning disability	889211000000104	2290291000000110
1939571000006115	14g00	14g0000	Early childhood developmental disability of unknown aetiology	954711000000106	2435131000000117
1947971000006116	Eu808	Eu80800	Developmental receptive language disorder	187921002	288890011
1947981000006118	Eu809	Eu80900	Acquired language comprehension impairment	716306006	3305472013
1947991000006115	Eu80A	Eu80A00	Developmental language comprehension impairment	716578009	3307207017
2107961000000111	PJ337	PJ33700	3p deletion syndrome	449819002	2168931000000118
2108161000000114	PJ331-3	PJ33113	18q deletion syndrome	270889005	2108161000000114
2108171000000119	PJ503-1	PJ50311	Trisomy 9 mosaic syndrome	74350000	123478012
2114791000000110	PJ33A	PJ33A00	Kleefstra syndrome	724207001	3432347016
2278791000000112	PJ54	PJ54.00	Ulnar mammary syndrome	700211007	2989585014
2278801000000111	PJ54-1	PJ54.11	Schinzel syndrome	700211007	2989657018
2288511000000116	13ZK0	13ZK000	Has statement of special educational needs	888421000000104	2288511000000116
2296351000000113	PJz31	PJz3100	MECP2 duplication syndrome	702816000	3006205015
2302801000000112	13Z4P	13Z4P00	Receiving learning support	894761000000108	2302801000000112
2388891000000118	14g0	14g0.00	Early childhood developmental disability	716710007	3307284014
2388901000000117	14g0-1	14g0.11	Early developmental impairment	716710007	3307285010
2417111000000110	1Bc0-1	1Bc0.11	Developmental dysarthria	230785002	2417111000000110
2435151000000112	13VC9	13VC900	Intellectual development disorder of unknown aetiology	954731000000103	2435151000000112
2508311000006110	^ESCTFR250831		FRAXA - Fragile X syndrome	613003	1232202017
2516621000006112	^ESCTDS251662		DSD - Developmental speech disorder	1145003	1220021016
2527721000006110	^ESCTMI252772		Microcephaly	1829003	4160017
2528311000006116	^ESCTDE252831		Developmental academic disorder	1855002	4203013
2528331000006110	^ESCTLE252833		Learning disorder	1855002	4208016
2528351000006115	^ESCTGE252835		General learning disability	1855002	478662017
2567771000006116	^ESCT11256777		11q partial monosomy syndrome	4325000	8287015
2589171000006110	^ESCTBA258917		Bardet-Biedl syndrome	5619004	10373017
2665621000006114	^ESCTLE266562		Lesch-Nyhan disease	10406007	272185011
2665631000006112	^ESCTHY266563		Hypoxanthine-guanine phosphoribosyltransferase deficiency	10406007	272186012
2670451000006119	^ESCTSP267045		Specific developmental disorder	10720004	18623010
2740491000000112	^ESCT1172259		Intellectual development disorder without significant impairment of behaviour	1094001000000106	2740491000000112
2740521000000110	^ESCT1172261		Intellectual development disorder with significant impairment of behaviour	1094011000000108	2740521000000110
2740551000000117	^ESCT1172263		Intellectual development disorder with minimal impairment of behaviour	1094021000000102	2740551000000117
2740581000000111	^ESCT1172265		Intellectual development disorder with impairment of behaviour	1094031000000100	2740581000000111
2771701000006114	^ESCT4P277170		4p partial monosomy syndrome	17122004	28958010
2782761000006112	^ESCTCR278276		Cross syndrome	17827007	30111010
2835541000006116	^ESCTCO283554		Complete trisomy 13 syndrome	21111006	35482019
2840101000006116	^ESCTDE284010		Developmental abnormality	21390004	1208681014

2923561000006111	^ESCTCA292356	Cat eye syndrome	26445008	44292015
3065731000006116	^ESCTTR306573	Trisomy X syndrome	35111009	58596018
3149211000006118	^ESCTDE314921	De Lange syndrome	40354009	63895010
3266991000006117	^ESCTME326699	Mental handicap	47437004	79090014
3267011000006111	^ESCTME326701	Mental subnormality	47437004	1230464013
3268621000006113	^ESCTCO326862	Coloboma, heart malformation, choanal atresia, retardation of growth and development, genital abnormalities, and ear malformations association	47535005	2914551016
3334491000006116	^ESCTCO333449	Complete trisomy 18 syndrome	51500006	85775018
3334501000006112	^ESCTED333450	Edwards syndrome	51500006	85776017
3462421000006114	^ESCTCU346242	Cutis laxa-corneal clouding-oligophrenia syndrome	59252009	98431015
3501751000006112	^ESCTAM350175	Amaurotic idiocy juvenile type	61663001	499087012
3514701000006113	^ESCTDE351470	Delayed articulatory and language development	62415009	103743015
3514711000006111	^ESCTDE351471	Developmental language delay	62415009	499275010
3562061000006117	^ESCTMU356206	Mucopolysaccharidosis, MPS-I-H	65327002	108537017
3562141000006117	^ESCTMU356214	Mucopolysaccharidosis type I-H	65327002	500080011
3563051000006112	^ESCTAD356305	Adrenoleukodystrophy	65389002	108634018
3563061000006114	^ESCTAD356306	Adrenomyeloneuropathy	65389002	108635017
3563111000006110	^ESCTAL356311	ALD - adrenoleukodystrophy	65389002	2957107010
3616531000006110	^ESCTRE361653	Rett's disorder	68618008	113976016
3616591000006114	^ESCTRE361659	Retts syndrome	68618008	2951769014
3629711000006110	^ESCTMU362971	Mucopolysaccharidosis type VI	69463008	501281010
3640931000006110	^ESCT5P364093	5p partial monosomy syndrome	70173007	116546014
3670581000006118	^ESCTCH367058	Childhood disintegrative disorder	71961003	119576018
3708621000006115	^ESCTCO370862	Complete trisomy 9 syndrome	74350000	123477019
3729521000006119	^ESCTMU372952	Mucopolysaccharidosis type I	75610003	503067012
3750051000006112	^ESCTAN375005	Angelman syndrome	76880004	127638013
3756821000006110	^ESCTBO375682	Borderline learning disability	77287004	503502012
3756851000006118	^ESCTBO375685	Borderline intellectual functioning	77287004	2951872010
3790911000006117	^ESCTLO379091	Lowe syndrome	79385002	131703019
3790971000006114	^ESCTCE379097	Cerebro-oculorenal dystrophy	79385002	1234350019
3811871000006110	^ESCTAI381187	Aicardi's syndrome	80651009	133795010
3828101000006111	^ESCTNE382810	Neurofibromatosis	81669005	135465015
3831011000006118	^ESCTAL383101	Alexander's disease	81854007	135788015
3831031000006112	^ESCTAL383103	Alexander disease	81854007	1234649011
4009971000006118	^ESCTNE400997	Neurofibromatosis 1	92824003	3009083017
4538731000006118	^ESCTLD453873	LD - Learning difficulties	161129001	251222017
4831001000006119	^ESCTTR483100	Trisomy 18 - meiotic nondisjunction	205623003	315354015
4831021000006112	^ESCTMO483102	Monosomy and deletion from autosome	205627002	315358017
4831061000006118	^ESCTWH483106	Whole chromosome monosomy - meiotic nondisjunction	205636003	315372014
4831091000006114	^ESCTWH483109	Whole chromosome trisomy syndrome	205646001	315388014
4831751000006117	^ESCTFO483175	Foetal alcohol syndrome	205788004	3010279011
4832001000006110	^ESCTNO483200	Noonan syndrome	205824006	2475676010
4926511000006114	^ESCTED492651	Educated at special needs school	224320009	337327011
4933261000006119	^ESCTSP493326	Special needs school	224863001	337985012
4935081000006116	^ESCTSL493508	Slow learner	224998004	338166012
4935091000006118	^ESCTLE493509	Learning delay	224998004	1222770016
4976271000006116	^ESCTSP497627	Special educational needs	228142005	342160013
4976281000006118	^ESCTSE497628	SEN - Special educational needs	228142005	342161012
4988761000006111	^ESCTSP498876	Special needs register	229056002	343386018
4998521000006113	^ESCTSP499852	Speech delay	229721007	344354016
4998611000006116	^ESCTDE499861	Developmental language impairment	229729009	344362012
4998671000006113	^ESCTEX499867	Expressive language delay	229734008	344368011
4998771000006115	^ESCTRE499877	Restricted language development	229741002	344376013
4998791000006119	^ESCTRE499879	Restricted receptive language development	229743004	344378014
5031301000006110	^ESCTLA503130	Laurence-Moon syndrome	232059000	347710015

5095461000006114	^ESCTPR509546	Prune belly syndrome with pulmonic stenosis, mental retardation and deafness	236529001	354548014
5116441000006113	^ESCTBE511644	Beta-D-mannosidosis	238047006	356823018
5129031000006113	^ESCTEC512903	Ectodermal dysplasia with hair-tooth-nail-sweating defect	239006001	358182016
5129141000006116	^ESCTEC512914	Ectodermal dysplasia with hair-tooth-nail defects	239015008	358194013
5129471000006118	^ESCTEC512947	Ectodermal dysplasia with nail defect	239046007	358233010
5245841000006110	^ESCTBE524584	Below average intellect	247575000	369677019
5340181000006114	^ESCTFR534018	FRAXA	254287005	378521015
5340191000006112	^ESCTFR534019	FRAXE	254288000	378522010
5517471000006110	^ESCTWH551747	Whole chromosome monosomy - mitotic nondisjunction mosaicism	270520003	405158019
5564871000006118	^ESCTDE556487	Delayed developmental milestone	274625009	2912590018
5591451000006112	^ESCTES559145	ESN - Educationally subnormal	276854003	413176017
5707211000006118	^ESCTSE570721	Severely educationally subnormal	285840007	425006015
5903381000006117	^ESCTFI590338	Finding relating to special educational needs	302141000	443698018
6459771000006113	^ESCTDE645977	Developmental articulation disorder	386701004	1480839016
6682941000006119	^ESCTOC668294	Oculo-cerebro-cutaneous syndrome (aplasia cutis, skin tags, eye & brain defects)	403554008	1782579015
6762781000006117	^ESCTLE676278	Learning disability - speciality	408468001	2154223015
6762801000006118	^ESCTLE676280	Learning disability	408468001	2163986010
7049741000006112	^ESCTCO704974	Cognitive developmental delay	425805004	2673542015
7265361000006111	^ESCTPR726536	Progressive encephalopathy with oedema, hypsarrhythmia and optic atrophy syndrome	442511009	2820530010
7281001000006112	^ESCTDE728100	Developmentally disabled	443656000	2838797017
7282231000006119	^ESCTNO728223	Nonverbal learning disorder	443735008	2842062017
7282241000006112	^ESCTNO728224	Nonverbal learning disability	443735008	2839181011
7295921000006119	^ESCTEX729592	Extra unidentified structurally abnormal chromosome	444655009	2870837017
7349161000006111	^ESCTFX734916	FXTAS - Fragile X associated tremor ataxia syndrome	448045004	1780771000000110
7375381000006117	^ESCTCH737538	Chromosome 3p deletion syndrome	449819002	2912647011
7495051000006114	^ESCTDE749505	Developmental regression	609225004	2958423017
7519841000006110	^ESCTOH751984	Ohdo syndrome, Maat-Kievit-Brunner type	699297004	2983700013
7519861000006114	^ESCTXL751986	X-linked Ohdo syndrome	699297004	2983727012
7519871000006119	^ESCTOH751987	Ohdo syndrome, Say-Barber-Biesecker-Young-Simpson variant	699298009	2983655015
7519891000006118	^ESCTSA751989	Say-Barber-Biesecker-Young-Simpson syndrome	699298009	2983642016
7525391000006116	^ESCTRE752539	Renpenning syndrome	699669001	2985573013
7534181000006118	^ESCTNE753418	Neurodevelopmental disorder	700364009	2989930017
7558081000006114	^ESCTAL755808	Allan-Herndon-Dudley syndrome	702327009	2995021019
7558801000006110	^ESCTCH755880	Chromosome 2q37 deletion syndrome	702357000	2995103015
7560021000006117	^ESCTPA756002	Partington syndrome	702412005	2995056015
7560111000006112	^ESCTSN756011	Snyder-Robinson syndrome	702416008	2995295014
7562541000006118	^ESCTDE756254	Developmental delay in receptive-expressive language	702528003	2995989017
7566981000006116	^ESCTME756698	Methyl-cytosine phosphate guanine binding protein-2 duplication syndrome	702816000	3006219015
7566991000006118	^ESCTME756699	Methyl-CpG (cytosine phosphate guanine) binding protein-2 duplication syndrome	702816000	3006199010
7574901000006110	^ESCTCA757490	CASK related intellectual disability	703389002	3008534018
7577041000006116	^ESCTNE757704	Neuronal ceroid lipofuscinosis 8	703526007	3009778015
7589061000006116	^ESCTIM758906	Impairment of child development	704304000	3012768013
7589071000006111	^ESCTIM758907	Impaired child development	704304000	3012776010
7589991000006111	^ESCTIM758999	Impaired infant development	704370008	3012989011
7730691000006117	^ESCTAL773069	Alpha thalassaemia X-linked intellectual disability syndrome	715342005	3302291017
7742511000006112	^ESCTEA774251	Early onset parkinsonism and intellectual disability syndrome	716107009	3304758012
7742581000006117	^ESCTKA774258	Kawashima Tsuji syndrome	716112005	3304777014
7743651000006117	^ESCTPE774365	Perniola Krajewska Carnevale syndrome	716191002	3305034010
7750931000006111	^ESCTFR775093	FRAXE intellectual disability syndrome	716709002	3307280017
7755141000006110	^ESCTL1775514	L1 syndrome	716996008	3308123013

7758091000006113	^ESCTXL775809	X-linked epilepsy with learning disability and behaviour disorder syndrome	717223008	3308721019
7769521000006118	^ESCTWO776952	Wolf Hirschhorn syndrome	718226002	3311589019
7774181000006117	^ESCTAC777418	Achalasia microcephaly syndrome	718573009	3312807014
7775921000006119	^ESCTOR777592	Oro-facial digital syndrome type 9	718680001	3313178014
7778251000006113	^ESCTFR777825	Fried syndrome	718848000	3314124019
7780351000006110	^ESCTXL778035	X-linked intellectual disability Schimke type	719010001	3314659018
7781071000006113	^ESCTSH778107	Shprintzen Goldberg craniosynostosis syndrome	719069008	3314858012
7781091000006114	^ESCTSH778109	Shprintzen-Goldberg syndrome	719069008	3314860014
7781471000006115	^ESCTBS778147	BSG syndrome	719097002	3314975017
7782161000006110	^ESCTXL778216	X-linked intellectual disability with cerebellar hypoplasia syndrome	719136005	3315132015
7782211000006118	^ESCTPE778221	Pettigrew syndrome	719139003	3315144017
7782501000006119	^ESCTXL778250	X-linked intellectual disability and hypotonia with facial dysmorphism and aggressive behaviour syndrome	719157002	3315196019
7782551000006115	^ESCTSY778255	Syndromic X-linked intellectual disability type 7	719160009	3315207010
7785901000006115	^ESCTMI778590	Microcephalus with brachydactyly and kyphoscoliosis syndrome	719378009	3316143016
7786681000006111	^ESCT15778668	15q11q13 microduplication syndrome	719427001	3316310010
7786891000006113	^ESCTDI778689	Disorder of sex development with intellectual disability syndrome	719450007	3316400017
7788481000006114	^ESCT16778848	16p13.11 microduplication syndrome	719578005	3316982015
7788521000006114	^ESCT17778852	17p13.3 microduplication syndrome	719582007	3316818012
7788761000006111	^ESCT19778876	19q13.11 microdeletion syndrome	719599008	3317093011
7789681000006118	^ESCT5Q778968	5q35 microduplication syndrome	719665003	3317303010
7791131000006110	^ESCTDO779113	DOORS syndrome	719800009	3321967011
7791151000006115	^ESCTDO779115	DOOR syndrome	719800009	3322824019
7791221000006119	^ESCTCH779122	Chromosome Xp11.3 microdeletion syndrome	719808002	3317983018
7791271000006118	^ESCTXL779127	X-linked intellectual disability Cabezas type	719811001	3317994019
7791631000006119	^ESCTWI779163	Wilson Turner syndrome	719834005	3318098016
7792761000006113	^ESCTTR779276	Trisomy Xq28 syndrome	719909009	3318425019
7792771000006118	^ESCTCH779277	Chromosome Xq28 trisomy syndrome	719909009	3318428017
7803961000006117	^ESCTAN780396	Aniridia and intellectual disability syndrome	720468000	3320943011
7804931000006111	^ESCTAU780493	Autosomal recessive limb girdle muscular dystrophy type 2K	720523006	3321129012
7806401000006115	^ESCTCE780640	Cerebro-facio-thoracic dysplasia	720635002	3321612013
7806471000006114	^ESCTCO780647	Coloboma, congenital heart disease, ichthyosiform dermatosis, intellectual disability ear anomaly syndrome	720639008	3321629011
7809711000006116	^ESCTFI780971	Filippi syndrome	720954000	3322630016
7809731000006110	^ESCTFI780973	Fine Lubinsky syndrome	720955004	3322635014
7809781000006111	^ESCTFO780978	Fountain syndrome	720957007	3322643016
7810331000006116	^ESCTAL781033	Alport syndrome, intellectual disability, midface hypoplasia, elliptocytosis syndrome	720982007	3322765013
7811761000006116	^ESCTDE781176	Deafness and intellectual disability Martin Probst type syndrome	721087008	3323440018
7811801000006113	^ESCTDE781180	Developmental delay, epilepsy, neonatal diabetes syndrome	721088003	3323445011
7813691000006118	^ESCTSE781369	Seizure, sensorineural deafness, ataxia, intellectual disability, electrolyte imbalance syndrome	721207002	3324614013
7826071000006112	^ESCTSC782607	Scholte syndrome	722002002	3330092011
7828621000006116	^ESCTSP782862	Spastic paraplegia, intellectual disability, palmoplantar hyperkeratosis syndrome	722209002	3331164011
7829441000006119	^ESCTAG782944	Agenesis of corpus callosum, intellectual disability, coloboma, micrognathia syndrome	722282008	3331425019
7831931000006118	^ESCTIN783193	Intellectual disability, developmental delay, contracture syndrome	722456001	3332213016
7832001000006117	^ESCTMA783200	Male hypergonadotropic hypogonadism, intellectual disability, skeletal anomaly syndrome	722459008	3332226017
7832211000006114	^ESCTSK783221	Skeletal dysplasia with intellectual disability syndrome	722478008	3332330016
7842691000006118	^ESCTIS784269	Isodicentric chromosome 15 syndrome	723332005	3424113016
7842701000006118	^ESCTDU784270	Duplication/inversion 15q11	723332005	3424114010

7842741000006116	^ESCTFA784274	Faciocardiorenal syndrome	723333000	3424121010
7850381000006114	^ESCTRE785038	Retinitis pigmentosa, intellectual disability, deafness, hypogenitalism syndrome	724001005	3481804013
7865911000006117	^ESCTTE786591	Temple Baraitser syndrome	725140007	3439393019
7870991000006110	^ESCTBU787099	Bullous dystrophy macular type	725589005	3444137014
7874821000006113	^ESCTIN787482	Intellectual disability Buenos Aires type	725906006	3446302016
7874881000006112	^ESCTNE787488	Neurofaciodigitorenal syndrome	725908007	3446330016
7877791000006114	^ESCTDU787779	Duplication of chromosome 3	726341009	3498271015
7881411000006110	^ESCTWE788141	Weaver Williams syndrome	726670008	3464489015
7881921000006114	^ESCTPR788192	Primrose syndrome	726709001	3451973010
7882141000006114	^ESCTXL788214	X-linked intellectual disability Nascimento type	726732002	3452277017
7952741000006112	^ESCTEP795274	Epilepsy, microcephaly, skeletal dysplasia syndrome	733031004	3498608019
7952761000006111	^ESCTEP795276	Epilepsy telangiectasia syndrome	733032006	3498611018
7953441000006114	^ESCTPS795344	Pseudoprogeria syndrome	733086003	3498717014
7957681000006110	^ESCTFA795768	Facial dysmorphism, macrocephaly, myopia, Dandy-Walker malformation syndrome	733417008	3499397016
7958391000006116	^ESCTMI795839	Microcephalus, glomerulonephritis, marfanoid habitus syndrome	733472005	3499508016
7958401000006119	^ESCT16795840	16p13.3 microduplication syndrome	733473000	3499511015
7958951000006114	^ESCT16795895	16p11.2p12.2 microduplication syndrome	733518000	3499592014
7959541000006113	^ESCTML795954	MLCRD (microcephaly with or without chorioretinopathy, lymphedema or intellectual disability) syndrome	733604003	3500041011
7963181000006117	^ESCTPO796318	Potocki Lupski syndrome	734016004	3481856017
8019831000006113	^ESCTMO801983	Moderate expressive language delay	62211000119103	2922262017
8019841000006115	^ESCTSE801984	Severe expressive language delay	62221000119105	2922281013
8019851000006118	^ESCTMI801985	Mild expressive language delay	62231000119108	2922254013
8026911000006110	^ESCTSE802691	Severe receptive language delay	89391000119105	2922263010
8026951000006111	^ESCTMI802695	Mild receptive language delay	89501000119108	2922240017
8056861000006118	^ESCTMI805686	Mixed receptive-expressive language delay	196901000000106	295681000000118
8195041000006117	^ESCTCA819504	Cause of learning disability	518831000000103	1158501000000112
8231171000006117	^ESCTNE823117	Neurodevelopmental delay	751391000000106	1653161000000110
8317111000006119	^ESCTSP831711	Speech and language developmental delay	898051000000104	2310281000000119
8337561000006119	^ESCTSI833756	Significant learning disability	931001000000105	2385981000000116
8337581000006112	^ESCTSI833758	Significant developmental disability	931001000000105	2714231000000111
8443531000006111	^ESCTDI844353	Difficulty learning basic skills	1070261000000101	2697211000000116
8443541000006118	^ESCTDI844354	Difficulty learning complex skills	1070271000000108	2697221000000110
11998101000006118	^ESCT1199810	Intellectual developmental disorder	110359009	3642975016
12000381000006119	^ESCT1200038	Charcot-Marie-Tooth disease, deafness, intellectual disability syndrome	763136000	3637979015
12000811000006118	^ESCT1200081	Grubben, De Cock, Borghgraef syndrome	763186006	3638112019
12002931000006115	^ESCT1200293	Cerebellar ataxia, intellectual disability, oculomotor apraxia, cerebellar cysts syndrome	763344007	3638624010
12007891000006110	^ESCT1200789	Wiedemann Steiner syndrome	763618001	3643042018
12009521000006116	^ESCT1200952	Intellectual disability, alacrima, achalasia syndrome	763741001	3643593016
12009551000006113	^ESCT1200955	Intellectual disability, spasticity, ectrodactyly syndrome	763743003	3643600019
12009571000006115	^ESCT1200957	Intellectual disability, brachydactyly, Pierre Robin syndrome	763744009	3643604011
12009911000006119	^ESCT1200991	Macrocephaly and developmental delay syndrome	763773007	3643793019
12010191000006115	^ESCT1201019	Malan overgrowth syndrome	763795006	3644015014
12010231000006113	^ESCT1201023	Agenesis of corpus callosum and abnormal genitalia syndrome	763797003	3644025016
12011241000006112	^ESCT1201124	Pachygyria, intellectual disability, epilepsy syndrome	763861000	3644781017
12016271000006113	^ESCT1201627	17q12 microduplication syndrome	764435003	3649828016
12016901000006114	^ESCT1201690	Mosaic trisomy 12	764463001	3649959018
12017691000006113	^ESCT1201769	Distal 22q11.2 microduplication syndrome	764524005	3650205012
12022541000006113	^ESCT1202254	Intellectual disability Birk-Barel type	764861005	3655432018
12023851000006118	^ESCT1202385	Cryptorchidism, arachnodactyly, intellectual disability syndrome	764950001	3655725014
12027071000006111	^ESCT1202707	Proximal 16p11.2 microduplication syndrome	765142003	3657168016
12027451000006111	^ESCT1202745	SCN8A-related epilepsy with encephalopathy	765170001	3657306014

12027461000006113	^ESCT1202746	SCN8A encephalopathy	765170001	3657302011
12047711000006118	^ESCT1204771	Nijmegen breakage syndrome-like disorder	766753005	3662497016
12049011000006112	^ESCT1204901	Diencephalic mesencephalic junction dysplasia	766871009	3662966013
12053971000006116	^ESCT1205397	22q11.2 deletion syndrome	767263007	3670124019
12077271000006118	^ESCT1207727	PPP2R5D-related intellectual disability	768677000	3686455015
12077811000006114	^ESCT1207781	15q13.3 microduplication syndrome	768713003	3686604015
12178711000006112	^ESCT1217871	Hyperphosphatasaemia with intellectual disability	33982008	3643091012
12197931000006114	^ESCT1219793	Borderline intellectual disability	77287004	3643525019
12204941000006113	^ESCT1220494	NF1 - Neurofibromatosis type 1	92824003	3671376015
12321891000006112	^ESCT1232189	Intellectual disability, congenital heart disease, blepharophimosis, blepharoptosis and hypoplastic teeth	412787009	3643126011
12337641000006113	^ESCT1233764	X-linked intellectual disability with marfanoid habitus	422437002	3643151012
12702211000006116	^ESCT1270221	Specific learning difficulty	889211000000104	2290321000000117
13491161000006116	^ESCT1349116	10q22.3q23.3 microdeletion syndrome	770401007	3700688012
13491351000006115	^ESCT1349135	Distal monosomy 19p13.3	770411000	3700744013
13491541000006114	^ESCT1349154	Early-onset epileptic encephalopathy and intellectual disability due to GRIN2A mutation	770431001	3700827018
13492091000006117	^ESCT1349209	Microcephalic primordial dwarfism Alazami type	770564004	3701291013
13492121000006118	^ESCT1349212	Monosomy 13q14 syndrome	770566002	3701297012
13492131000006115	^ESCT1349213	Deletion 13q14	770566002	3701299010
13492711000006113	^ESCT1349271	Ring chromosome 12 syndrome	770595006	3701491010
13493581000006116	^ESCT1349358	Tetrasomy 11q24.1	770663003	3701913015
13493791000006114	^ESCT1349379	Progressive encephalopathy with oedema, hypsarrhythmia, and optic atrophy-like syndrome	770678005	3702074016
13494421000006116	^ESCT1349442	3q27.3 microdeletion syndrome	770719004	3702236016
13494971000006112	^ESCT1349497	Intellectual disability, seizures, hypotonia, ophthalmologic, skeletal anomalies syndrome	770755007	3702470017
13497191000006119	^ESCT1349719	Autosomal recessive intellectual disability, motor dysfunction, multiple joint contracture syndrome	770901001	3703343018
13497201000006116	^ESCT1349720	Recessive intellectual disability, motor dysfunction, multiple joint contractures syndrome	770901001	3703344012
13497321000006119	^ESCT1349732	Kagami Ogata syndrome	770907002	3703367014
13497341000006114	^ESCT1349734	49,XXXYY syndrome	770908007	3703371012
13497811000006119	^ESCT1349781	Rhizomelic syndrome Urbach type	770948004	3703567013
13498421000006112	^ESCT1349842	Monosomy 9p	771072001	3704188015
13499591000006116	^ESCT1349959	Hepatic fibrosis, renal cyst, intellectual disability syndrome	771149000	3704579019
13501631000006113	^ESCT1350163	Polymicrogyria with optic nerve hypoplasia	771336003	3705808010
13501641000006115	^ESCT1350164	1q21.1 microduplication syndrome	771337007	3705813014
13503121000006119	^ESCT1350312	Developmental and speech delay due to SOX5 deficiency	771472009	3706361019
13503701000006111	^ESCT1350370	Autism spectrum disorder due to AUTS2 deficiency	771512003	3706567016
13510661000006110	^ESCT1351066	White Sutton syndrome	772127009	3717154011
13520601000006115	^ESCT1352060	Cyclin-dependent kinase-like 5 deficiency	773230003	3722626014
13520621000006113	^ESCT1352062	CDKL5 deficiency disorder	773230003	3722625013
13521771000006113	^ESCT1352177	Distal 7q11.23 microduplication syndrome	773325004	3723181013
13521831000006119	^ESCT1352183	CK syndrome	773329005	3723193016
13521841000006112	^ESCT1352184	X-linked intellectual disability, microcephaly, cortical malformation, thin habitus syndrome	773329005	3723194010
13522491000006113	^ESCT1352249	Autosomal recessive frontotemporal pachygyria	773394007	3723363018
13522641000006119	^ESCT1352264	Severe feeding difficulties, failure to thrive, microcephaly due to ASXL3 deficiency syndrome	773400009	3723393012
13522651000006117	^ESCT1352265	Bainbridge Roppers syndrome	773400009	3723394018
13522691000006111	^ESCT1352269	Intellectual disability with strabismus syndrome	773405004	3723417011
13523971000006111	^ESCT1352397	Autosomal recessive cerebellar ataxia, epilepsy, intellectual disability syndrome due to TUD deficiency	773498006	3723738016
13524851000006112	^ESCT1352485	Intellectual disability, craniofacial dysmorphism, cryptorchidism syndrome	773581009	3724281013

13525931000006114	^ESCT1352593	Distal Xq28 microduplication syndrome	773670004	3725602017
13529071000006110	^ESCT1352907	Peripheral dysostosis	773985008	3727471017
13530051000006113	^ESCT1353005	AHDC1-related intellectual disability, obstructive sleep apnoea, mild dysmorphism syndrome	774068004	3727864017
13530061000006110	^ESCT1353006	Xia Gibbs syndrome	774068004	3727860014
13621171000006117	^ESCT1362117	Distal trisomy 18q	782676009	3755116014
13622051000006117	^ESCT1362205	Intellectual disability, facial dysmorphism syndrome due to SETD5 haploinsufficiency	782736007	3755540013
13622301000006119	^ESCT1362230	Intellectual disability, coarse face, macrocephaly, cerebellar hypotrophy syndrome	782753000	3755618014
13622321000006112	^ESCT1362232	Autosomal recessive spinocerebellar ataxia type 20	782753000	3755620012
13622561000006113	^ESCT1362256	Congenital muscular dystrophy with intellectual disability and severe epilepsy	782772000	3755710013
13622571000006118	^ESCT1362257	Congenital disorder of glycosylation type 1u	782772000	3755714016
13622581000006115	^ESCT1362258	Carbohydrate deficient glycoprotein syndrome type 1u	782772000	3755716019
13624181000006116	^ESCT1362418	Infantile spasms, psychomotor retardation, progressive brain atrophy, basal ganglia disease syndrome	782886007	3756554010
13625421000006113	^ESCT1362542	Severe microbrachycephaly, intellectual disability, athetoid cerebral palsy syndrome	783005002	3757107011
13627911000006119	^ESCT1362791	Congenital muscular dystrophy with intellectual disability	783174004	3757954011
13630921000006112	^ESCT1363092	Has special educational needs	783572008	3759384019
13631361000006113	^ESCT1363136	DYRK1A-related intellectual disability syndrome due to 21q22.13q22.2 microdeletion	783619003	3759527011
13632231000006117	^ESCT1363223	White matter hypoplasia, corpus callosum agenesis, intellectual disability syndrome	783703004	3759781012
13662101000006111	^ESCT1366210	Developmental delay, facial dysmorphism syndrome due to MED13L deficiency	787093004	3773933016
13697811000006116	^ESCT1369781	Significant learning disability	1239331000000100	2800231000000118
13784441000006119	^ESCT1378444	Learning disability	110359009	2800271000000116
13904831000006116	^ESCT1390483	Alopecia, epilepsy, intellectual disability syndrome Moynahan type	788417006	3780616016
13939301000006114	^ESCT1393930	MASA syndrome	838441009	3896942012
13963361000006115	^ESCT1396336	Pervasive developmental disorder with disorder of intellectual development without loss of previously acquired skills	870262000	3968748015
13963421000006115	^ESCT1396342	Pervasive developmental disorder with disorder of intellectual development with loss of previously acquired skills	870265003	3968757014
13963431000006117	^ESCT1396343	Autism spectrum disorder with disorder of intellectual development and with mild or no impairment of functional language with loss of previously acquired skills	870265003	3968756017
13963441000006110	^ESCT1396344	Pervasive developmental disorder with disorder of intellectual development and marked impairment of functional language with loss of previously acquired skills	870266002	3968759012
13963451000006112	^ESCT1396345	Autism spectrum disorder with disorder of intellectual development and with impaired functional language with loss of previously acquired skills	870266002	3968761015
13963461000006114	^ESCT1396346	Pervasive developmental disorder with disorder of intellectual development and marked impairment of functional language without loss of previously acquired skills	870267006	3968763017
13963471000006119	^ESCT1396347	Autism spectrum disorder with disorder of intellectual development and impaired functional language without loss of previously acquired skills	870267006	3968762010
13963481000006116	^ESCT1396348	Pervasive developmental disorder with disorder of intellectual development and complete impairment of functional language without loss of previously acquired skills	870268001	3968767016

13963491000006118	^ESCT1396349	Autism spectrum disorder with disorder of intellectual development and complete impairment of functional language without loss of previously acquired skills	870268001	3968765012
13963521000006116	^ESCT1396352	Pervasive developmental disorder with disorder of intellectual development and complete impairment of functional language with loss of previously acquired skills	870270005	3968771018
13963531000006118	^ESCT1396353	Autism spectrum disorder with disorder of intellectual development and complete impairment of functional language with loss of previously acquired skills	870270005	3968773015
13964061000006119	^ESCT1396406	Pervasive developmental disorder with cognitive developmental delay and marked impairment of functional language	870305003	3968864019
13964071000006114	^ESCT1396407	Autism spectrum disorder with disorder of intellectual development and impaired functional language	870305003	3968863013
13964131000006112	^ESCT1396413	Autism spectrum disorder with disorder of intellectual development and complete impairment of functional language	870308001	3968871012
14072761000006111	^ESCT1407276	Hennekam syndrome	234146006	3789477016
14132561000006113	^ESCT1413256	Bilateral megalencephaly	879919001	3993786019
14134231000006111	^ESCT1413423	12q15 deletion syndrome	880081006	3994444018
14135491000006110	^ESCT1413549	3p25.3 deletion syndrome	890123006	4011205015
14135571000006113	^ESCT1413557	9q34 deletion syndrome	890130000	4009227018
14137521000006116	^ESCT1413752	Bilateral frontal polymicrogyria	890285006	4011911011
14137531000006118	^ESCT1413753	Bilateral frontoparietal polymicrogyria	890286007	4011914015
14152591000006118	^ESCT1415259	Molybdenum cofactor deficiency complementation group B	1003368009	4168093015
14152831000006119	^ESCT1415283	Mosaic 1q duplication	1003389000	4168139015
14153041000006114	^ESCT1415304	Maternal 15q11q13 deletion	1003409002	4168186016
14163361000006116	^ESCT1416336	X-linked complicated corpus callosum dysgenesis	1010630006	4213373017
14432361000006117	^ESCT1443236	Psychomotor retardation	1144814003	4543404012
14441711000006111	^ESCT1444171	Microcephaly	1148757008	4551753012
14441721000006115	^ESCT1444172	Microcephalus	1148757008	4551752019
14452321000006112	^ESCT1445232	Partial deletion of short arm of chromosome 5	1153583005	4565146017
14507171000006113	^ESCT1450717	Oculocerebrocutaneous syndrome	403554008	4579831016
14512561000006111	^ESCT1451256	Trisomy Xq28	719909009	4570723012
14549611000006112	^ESCT1454961	Brain malformations, musculoskeletal abnormalities, facial dysmorphism, intellectual disability syndrome	1169355000	4607740010
14552291000006113	^ESCT1455229	Early-onset epilepsy, intellectual disability, brain anomalies syndrome	1172627007	4634848019
14552331000006118	^ESCT1455233	TBCK-related intellectual disability syndrome	1172628002	4634855017
14552351000006113	^ESCT1455235	Severe growth deficiency, strabismus, extensive dermal melanocytosis, intellectual disability syndrome	1172629005	4634867015
14553091000006112	^ESCT1455309	Global developmental delay, visual anomalies, progressive cerebellar atrophy, truncal hypotonia syndrome	1172696009	4635425014
14564831000006111	^ESCT1456483	DYRK1A-related intellectual disability syndrome	1179301003	4650644016
14566451000006113	^ESCT1456645	CHD3-related developmental delay, speech delay, intellectual disability, abnormalities of vision, facial dysmorphism syndrome	1179408008	4651034011
14576241000006115	^ESCT1457624	STAG1-related intellectual disability, facial dysmorphism, gastrooesophageal reflux syndrome	1187041000	4669042011
14577761000006112	^ESCT1457776	Intellectual disability, epilepsy, extrapyramidal syndrome	1187210007	4672728014
14578491000006112	^ESCT1457849	Macrocephaly, intellectual disability, neurodevelopmental disorder, small thorax syndrome	1187304005	4673265016
14608951000006111	^ESCT1460895	Tetrasomy 12p syndrome	9527009	4589824013
14753781000006110	^ESCT1475378	PHIP-related behavioural problems, intellectual disability, obesity, dysmorphic features syndrome	1208987006	5013861019
14770551000006118	^ESCT1477055	Neurodevelopmental disorder, craniofacial dysmorphism, cardiac defect, skeletal anomalies syndrome	1222710008	5048626018
14790031000006114	^ESCT1479003	CNTNAP2-related developmental and epileptic encephalopathy	1230376005	5069594018

15021671000006111	^ESCT1502167	Autosomal dominant intellectual disability, craniofacial anomalies, cardiac defects syndrome	1255319004	5146300011
15047751000006115	^ESCT1504775	GRIN2B-related developmental delay, intellectual disability, autism spectrum disorder	1260195002	5159667013
15080241000006115	^ESCT1508024	Prune belly syndrome with pulmonic stenosis, intellectual disability and deafness	236529001	5155337016
15242911000006112	^ESCT1524291	Developmental and epileptic encephalopathy	1275631007	5224368014

Supplementary Code List 2. Codes for the ascertainment of cancer cases in primary care records.

MedCodeId	OriginalReadCode	CleansedReadCode	Term	Classification	SnomedCTConceptId	SnomedCTDescriptionId
1228259019	BB911	BB91100	[M]Infiltrating duct and lobular carcinoma	Breast	35232005	1228260012
1231363012	BB9K0	BB9K000	[M]Paget's disease and intraductal carcinoma of breast	Breast	54666007	1231364018
5351511000006119	^ESCTBR535151		Breast cancer	Breast	254837009	379662011
880331000006115	B34z-99	B34z.99	Ca breast - NOS	Breast	549521000000102	880331000006115
880301000006111	B344-99	B344.99	Ca breast-upper,outer quadrant	Breast	188154003	880301000006111
531851000006119	B34-1	B34..11	Ca female breast	Breast	372064008	1210642019
880261000006119	B34-99	B34..99	Carcinoma breast	Breast	372064008	880261000006119
5351541000006115	^ESCTCA535154		Carcinoma of breast	Breast	254838004	379666014
7345821000006113	^ESCTCA734582		Carcinoma of female breast	Breast	447782002	2899466013
310311000006110	BB91	BB91.00	Infiltrating duct carcinoma	Breast	82711006	137203014
6765091000006112	^ESCTIN676509		Infiltrating duct carcinoma of breast	Breast	408643008	2160190012
7706551000006117	^ESCTIN770655		Invasive carcinoma of breast	Breast	713609000	3290542017
459378019	B36	B36..00	Local recurrence of malignant tumour of breast	Breast	314955001	459378019
155089014	B3401	B340100	Malignant neoplasm of areola of female breast	Breast	93680004	155089014
155090017	B3501	B350100	Malignant neoplasm of areola of male breast	Breast	93681000	155090017
289146012	B346	B346.00	Malignant neoplasm of axillary tail of female breast	Breast	188156001	289146012
289141019	B341	B341.00	Malignant neoplasm of central part of female breast	Breast	188151006	289141019
289149017	B34y0	B34y000	Malignant neoplasm of ectopic site of female breast	Breast	188159008	289149017
289158012	B35z0	B35z000	Malignant neoplasm of ectopic site of male breast	Breast	188168005	289158012
1210642019	B34	B34..00	Malignant neoplasm of female breast	Breast	372064008	1210642019
289151018	B34z	B34z.00	Malignant neoplasm of female breast NOS	Breast	372064008	1210642019
289143016	B343	B343.00	Malignant neoplasm of lower-inner quadrant of female breast	Breast	188153009	289143016
289145011	B345	B345.00	Malignant neoplasm of lower-outer quadrant of female breast	Breast	188155002	289145011
155364013	B35	B35..00	Malignant neoplasm of male breast	Breast	372095001	1210673015
289159016	B35zz	B35zz00	Malignant neoplasm of male breast NOS	Breast	372095001	1210673015
289137018	B340	B340.00	Malignant neoplasm of nipple and areola of female breast	Breast	188147009	289137018
289153015	B350	B350.00	Malignant neoplasm of nipple and areola of male breast	Breast	188163001	289153015
155417012	B3400	B340000	Malignant neoplasm of nipple of female breast	Breast	93924008	155417012
155418019	B3500	B350000	Malignant neoplasm of nipple of male breast	Breast	93925009	155418019
289140018	B340z	B340z00	Malignant neoplasm of nipple or areola of female breast NOS	Breast	188147009	289137018
289148013	B34y	B34y.00	Malignant neoplasm of other site of female breast	Breast	372064008	1210642019
289150017	B34yz	B34yz00	Malignant neoplasm of other site of female breast NOS	Breast	372064008	1210642019
289157019	B35z	B35z.00	Malignant neoplasm of other site of male breast	Breast	372095001	1210673015
289142014	B342	B342.00	Malignant neoplasm of upper-inner quadrant of female breast	Breast	188152004	289142014
289144010	B344	B344.00	Malignant neoplasm of upper-outer quadrant of female breast	Breast	188154003	289144010
289147015	B347	B347.00	Malignant neoplasm, overlapping lesion of breast	Breast	188157005	289147015
292137017	Byu6	Byu6.00	Malignant tumour of breast	Breast	254837009	379664012

314711000006117	BB9J	BB9J.00	Mammary Paget's disease	Breast	2985005	1226519014
6052731000006114	^ESCTME605273		Metastasis from malignant tumour of breast	Breast	315004001	459458011
314691000006115	BB9J-1	BB9J.11	Paget's disease of breast	Breast	2985005	6068012
7610911000006117	^ESCTTR761091		Triple-negative breast cancer	Breast	706970001	3028054017
1228493018	B41	B41..00	Malignant neoplasm of cervix uteri	Cervix	363354003	1228493018
289182019	B41z	B41z.00	Malignant neoplasm of cervix uteri NOS	Cervix	363354003	1228493018
289171010	B4100	B410000	Malignant neoplasm of endocervical canal	Cervix	188176007	289171010
289172015	B4101	B410100	Malignant neoplasm of endocervical gland	Cervix	188177003	289172015
155218017	B410	B410.00	Malignant neoplasm of endocervix	Cervix	372097009	1210675010
289173013	B410z	B410z00	Malignant neoplasm of endocervix NOS	Cervix	372097009	1210675010
155231010	B411	B411.00	Malignant neoplasm of exocervix	Cervix	372099007	1210677019
289178016	B41y	B41y.00	Malignant neoplasm of other site of cervix	Cervix	363354003	482507015
289181014	B41yz	B41yz00	Malignant neoplasm of other site of cervix NOS	Cervix	363354003	482507015
289180010	B41y1	B41y100	Malignant neoplasm of squamocolumnar junction of cervix	Cervix	188184006	289180010
289175018	B412	B412.00	Malignant neoplasm, overlapping lesion of cervix uteri	Cervix	188180002	289175018
543481000006111	B41-1	B41..11	Malignant tumour of cervix	Cervix	363354003	482507015
5352301000006115	^ESCTSQ535230		Squamous cell carcinoma of cervix	Cervix	254886006	379746012
1229453012	BBbB	BBbB.00	[M]Astrocytoma NOS	CNS	38713004	1229453012
309261000006112	BBb0-1	BBb0.11	[M]Glioma NOS	CNS	74532006	2552865010
2552865010	BBbz	BBbz.00	[M]Glioma NOS	CNS	74532006	2552865010
2550105015	BBb	BBb..00	[M]Gliomas	CNS	115240006	2550105015
305701000006117	BBbC	BBbC.00	Anaplastic astrocytoma	CNS	55353007	1231460011
314281000006115	BBbR	BBbR.00	Anaplastic oligodendroglioma	CNS	3102004	1227417016
305681000006115	BBbB-1	BBbB.11	Astrocytoma	CNS	38713004	64413017
5353191000006116	^ESCTAS535319		Astrocytoma of brain	CNS	254938000	379848018
308091000006116	BBb8	BBb8.00	Ependymoma, anaplastic	CNS	21589007	36220016
403757015	BBbL	BBbL.00	Glioblastoma	CNS	63634009	105759017
309231000006115	BBbL-1	BBbL.11	Glioblastoma multiforme	CNS	63634009	105761014
6545171000006115	^ESCTGL654517		Glioblastoma multiforme	CNS	393563007	1487417014
5591151000006115	^ESCTGL559115		Glioblastoma multiforme of brain	CNS	276828006	413144010
175996013	BBb-99	BBb..99	Glioma	CNS	115240006	175996013
309281000006119	BBb0	BBb0.00	Malignant glioma	CNS	74532006	1788143011
5591131000006110	^ESCTMA559113		Malignant glioma of brain	CNS	276826005	413142014
727841000006110	B5202	B520200	Malignant neoplasm of acoustic nerve	CNS	254980001	379934019
289337012	B5100	B510000	Malignant neoplasm of basal ganglia	CNS	188281006	289337012
2692069010	B51	B51..00	Malignant neoplasm of brain	CNS	428061005	2692069010
289365014	B51z	B51z.00	Malignant neoplasm of brain NOS	CNS	428061005	2692069010
727521000006118	B517z	B517z00	Malignant neoplasm of brain stem NOS	CNS	363473003	482736017
727511000006114	B517	B517.00	Malignant neoplasm of brainstem	CNS	363473003	482736017
396251000006115	ByuA	ByuA.00	Malignant neoplasm of central nervous system	CNS	372062007	1210640010
396491000006113	ByuA1	ByuA100	Malignant neoplasm of central nervous system	CNS	372062007	1210640010
155175018	B516	B516.00	Malignant neoplasm of cerebellum	CNS	449420002	2899889012
289338019	B5101	B510100	Malignant neoplasm of cerebral cortex	CNS	188282004	289338019
482737014	B521	B521.00	Malignant neoplasm of cerebral meninges	CNS	363474009	482737014
289379018	B521z	B521z00	Malignant neoplasm of cerebral meninges NOS	CNS	363474009	482737014
289355015	B5170	B517000	Malignant neoplasm of cerebral peduncle	CNS	188295009	289355015
482734019	B515	B515.00	Malignant neoplasm of cerebral ventricles	CNS	363471001	482734019
727771000006111	B510	B510.00	Malignant neoplasm of cerebrum (excluding lobes and ventricles)	CNS	188280007	289336015
289346018	B510z	B510z00	Malignant neoplasm of cerebrum NOS	CNS	188280007	289336015
482730011	B511	B511.00	Malignant neoplasm of frontal lobe	CNS	363467004	482730011
289347010	B5120	B512000	Malignant neoplasm of hippocampus	CNS	188289008	289347010

289343014	B5104	B510400	Malignant neoplasm of hypothalamus	CNS	188286001	289343014
289356019	B5171	B517100	Malignant neoplasm of medulla oblongata	CNS	188296005	289356019
724681000006111	B52X	B52X.00	Malignant neoplasm of meninges, unspecified	CNS	363497007	482781018
289357011	B5172	B517200	Malignant neoplasm of midbrain	CNS	188297001	289357011
482733013	B514	B514.00	Malignant neoplasm of occipital lobe	CNS	363470000	482733013
289369015	B5200	B520000	Malignant neoplasm of olfactory bulb	CNS	188308004	289369015
155425014	B5201	B520100	Malignant neoplasm of optic nerve	CNS	363498002	482783015
289364013	B51yz	B51yz00	Malignant neoplasm of other part of brain NOS	CNS	428061005	2692069010
289360016	B51y	B51y.00	Malignant neoplasm of other parts of brain	CNS	428061005	2692069010
482732015	B513	B513.00	Malignant neoplasm of parietal lobe	CNS	363469001	482732015
722961000006116	B543	B543.00	Malignant neoplasm of pineal gland	CNS	363483004	482752019
289358018	B5173	B517300	Malignant neoplasm of pons	CNS	188298006	289358018
721751000006111	B522	B522.00	Malignant neoplasm of spinal cord	CNS	363475005	482738016
482740014	B523	B523.00	Malignant neoplasm of spinal meninges	CNS	363476006	482740014
289383018	B523z	B523z00	Malignant neoplasm of spinal meninges NOS	CNS	363497007	482781018
482731010	B512	B512.00	Malignant neoplasm of temporal lobe	CNS	363468009	482731010
289349013	B512z	B512z00	Malignant neoplasm of temporal lobe NOS	CNS	363468009	482731010
289345019	B5105	B510500	Malignant neoplasm of thalamus	CNS	188287005	289345019
291836013	BBbT	BBbT.00	Medulloblastoma	CNS	83217000	138016017
291835012	BBbQ	BBbQ.00	Oligodendroglioma - category	CNS	443565000	2840882012
315291000006116	BBbG	BBbG.00	Pilocytic astrocytoma	CNS	128854008	207484016
314231000006116	BBcC-1	BBcC.11	Olfactory neuroblastoma	CNS	76060004	126335013
726021000006110	B120	B120.00	Malignant tumour of duodenum	Digestive tract	363403002	482606016
396651000006117	Byu13	Byu1300	Malignant neoplasm of gastrointestinal tract	Digestive tract	428905002	2693067015
155294016	B3060	B306000	Malignant neoplasm of ilium	Digestive tract	93833009	155294016
288659012	B123	B123.00	Malignant neoplasm of Meckel's diverticulum	Digestive tract	187752007	288659012
723221000006112	B12y	B12y.00	Malignant neoplasm of other specified site small intestine	Digestive tract	363509000	482804016
288663017	B12z	B12z.00	Malignant neoplasm of small intestine NOS	Digestive tract	363509000	482804016
292099015	Byu1	Byu1.00	Malignant tumour of digestive organ	Digestive tract	255077007	380111011
725271000006112	B122	B122.00	Malignant tumour of ileum	Digestive tract	363405009	482610018
288759015	B1z0	B1z0.00	Malignant tumour of intestine	Digestive tract	363508008	482802017
721601000006117	B12	B12..00	Malignant tumour of small intestine	Digestive tract	363509000	482805015
730011000006119	B201	B201.00	Malignant neoplasm of auditory tube, middle ear and mastoid air cells	Ear	187833006	288777013
730021000006110	B201z	B201z00	Malignant neoplasm of auditory tube, middle ear and mastoid air cells	Ear	187833006	288777013
724621000006112	B2013	B201300	Malignant neoplasm of mastoid air cells	Ear	363424007	482646010
1235052014	BBD9	BBD9.00	[M]Phaeochromocytoma NOS	Endocrine	85583005	1235052014
305011000006117	BB5h1	BB5h100	Adrenal cortical carcinoma	Endocrine	2227007	4846017
880531000006114	B53-99	B53..99	Ca thyroid	Endocrine	363478007	880531000006114
7351581000006113	^ESCTCA735158		Carcinoma of thyroid	Endocrine	448216007	2900738013
5354731000006117	^ESCTFO535473		Follicular thyroid carcinoma	Endocrine	255028004	380006011
729471000006114	B54z	B54z.00	Malig neop of endocrine gland or related structure NOS	Endocrine	371982006	1210569011
729511000006116	B54	B54..00	Malig neop of other endocrine glands and related structures	Endocrine	371982006	1210569011
1210553016	B5400	B540000	Malignant neoplasm of adrenal cortex	Endocrine	371964008	1210553016
155072018	B540	B540.00	Malignant neoplasm of adrenal gland	Endocrine	363355002	482509017
1210554010	B5401	B540100	Malignant neoplasm of adrenal medulla	Endocrine	371965009	1210554010
155085015	B5451	B545100	Malignant neoplasm of aortic body	Endocrine	93677000	155085015
289419010	B545z	B545z00	Malignant neoplasm of aortic body or paraganglia NOS	Endocrine	93941005	510894017
289409016	B5421	B542100	Malignant neoplasm of craniopharyngeal duct	Endocrine	188340000	289409016
292154013	ByuB	ByuB.00	Malignant neoplasm of endocrine gland	Endocrine	371982006	1210569011
292156010	ByuB1	ByuB100	Malignant neoplasm of endocrine gland	Endocrine	371982006	1210569011

289421017	B54y	B54y.00	Malignant neoplasm of other specified endocrine gland	Endocrine	371982006	1210569011
723441000006116	B541	B541.00	Malignant neoplasm of parathyroid gland	Endocrine	363481002	482748019
722981000006114	B5420	B542000	Malignant neoplasm of pituitary gland	Endocrine	363482009	482750010
720481000006117	B542	B542.00	Malignant neoplasm of pituitary gland and craniopharyngeal duct	Endocrine	188339002	289407019
289406011	B540z	B540z00	Malignant tumour of adrenal gland	Endocrine	363355002	482510010
721291000006110	B53	B53..00	Malignant tumour of thyroid gland	Endocrine	363478007	482743011
6246931000006118	^ESCTMA624693		Malignant tumour of thyroid gland	Endocrine	363478007	6246931000006118
5354751000006112	^ESCTPA535475		Papillary thyroid carcinoma	Endocrine	255029007	380009016
444773019	B540-1	B540.11	Phaeochromocytoma	Endocrine	302835009	444773019
289413011	B545	B545.00	Primary malignant neoplasm of paraganglion	Endocrine	93941005	510894017
730041000006115	B542z	B542z00	Malig neop pituitary gland or craniopharyngeal duct NOS	Endocrine	188339002	289407019
292142013	Byu73	Byu7300	[X]Malignant neoplasm of female genital organ, unspecified	Gynae	363514001	482815014
292138010	Byu7	Byu7.00	[X]Malignant neoplasm of female genital organs	Gynae	363514001	482815014
292139019	Byu70	Byu7000	[X]Malignant neoplasm of uterine adnexa, unspecified	Gynae	428322007	2694321016
729491000006110	B45	B45..00	Malig neop of other and unspecified female genital organs	Gynae	363514001	482815014
726271000006117	B441	B441.00	Malignant neoplasm of fallopian tube	Gynae	363444001	482686019
482690017	B451	B451.00	Malignant neoplasm of labia majora	Gynae	363446004	482690017
289210012	B451z	B451z00	Malignant neoplasm of labia majora NOS	Gynae	363446004	482690017
482691018	B452	B452.00	Malignant neoplasm of labia minora	Gynae	363447008	482691018
289203018	B44y	B44y.00	Malignant neoplasm of other site of uterine adnexa	Gynae	428322007	2694321016
289217010	B45y	B45y.00	Malignant neoplasm of other specified female genital organ	Gynae	363514001	482815014
155441010	B443	B443.00	Malignant neoplasm of parametrium	Gynae	448674007	2901968015
155468010	B42	B42..00	Primary malignant neoplasm of placenta	Other/unknown	721567004	3325477011
289199018	B44	B44..00	Malignant neoplasm of uterine adnexa	Gynae	428322007	2694321016
289204012	B44z	B44z.00	Malignant neoplasm of uterine adnexa NOS	Gynae	428322007	2694321016
289208010	B450z	B450z00	Malignant neoplasm of vagina NOS	Gynae	363445000	482688018
289207017	B4501	B450100	Malignant neoplasm of vaginal vault	Gynae	188209005	289207017
396531000006113	Byu71	Byu7100	Malignant tumor of female genital organ	Gynae	363514001	482815014
6246161000006113	^ESCTMA624616		Malignant tumour of fallopian tube	Gynae	363444001	482687011
289219013	B45z	B45z.00	Malignant tumour of female genital organ	Gynae	363514001	482815014
288388017	B004	B004.00	Malignant tumour of labial mucosa	Gynae	187622006	288402019
724401000006116	B0032	B003200	Malignant tumour of lower labial mucosa	Gynae	271568003	406400016
721051000006113	B450	B450.00	Malignant tumour of vagina	Gynae	363445000	482688018
403701012	B454	B454.00	Malignant tumour of vulva	Gynae	363367000	482534017
313211000006113	BBL5	BBL5.00	Mullerian mixed tumour	Gynae	84427001	201141019
723341000006112	B45y0	B45y000	Overlapping malignant neoplasm of vulva	Gynae	109885001	174573010
411399019	B454-1	B454.11	Primary vulval cancer	Gynae	275419009	411399019
5352431000006116	^ESCTSQ535243		Squamous cell carcinoma of vulva	Gynae	254895003	379761013
155155010	B442	B442.00	Malignant neoplasm of broad ligament	Gynae	93728003	155155010
1210566016	B453	B453.00	Malignant neoplasm of clitoris	Gynae	371979001	1210566016
396301000006110	Byu0	Byu0.00	[X]Malignant neoplasm of lip, oral cavity and pharynx	Head and Neck	271323007	406052010
380095017	B0-1	B0...11	Carcinoma of lip, oral cavity and pharynx	Head and Neck	255069008	380095017
808171000006118	142B	142B.00	History of malignant neoplasm of ear, nose AND/OR throat	Head and Neck	415078001	2534965019
729591000006114	B0z	B0z..00	Malig neop other/ill-defined sites lip, oral cavity, pharynx	Head and Neck	271323007	406052010

727561000006112	B050-1	B050.11	Malignant neoplasm of buccal mucosa	Head and Neck	363386008	482571015
727151000006117	B050	B050.00	Malignant neoplasm of cheek mucosa	Head and Neck	363386008	482571015
726351000006116	B005	B005.00	Malignant neoplasm of commissure of lip	Head and Neck	363374005	482544015
155211011	B011	B011.00	Malignant neoplasm of dorsal surface of tongue	Head and Neck	187633009	288413016
288418013	B011z	B011z00	Malignant neoplasm of dorsum of tongue NOS	Head and Neck	187633009	288413016
1228553019	B203	B203.00	Malignant neoplasm of ethmoid sinus	Head and Neck	363426009	1228553019
288412014	B010z	B010z00	Malignant neoplasm of fixed part of tongue NOS	Head and Neck	363376007	482548017
725611000006117	B04	B04..00	Malignant neoplasm of floor of mouth	Head and Neck	363385007	2969212012
288427014	B0131	B013100	Malignant neoplasm of frenulum linguae	Head and Neck	187641009	288427014
719021000006111	B204	B204.00	Malignant neoplasm of frontal sinus	Head and Neck	363427000	482652011
288794010	B210	B210.00	Malignant neoplasm of glottis	Head and Neck	187841006	288794010
725101000006111	B052	B052.00	Malignant neoplasm of hard palate	Head and Neck	363387004	482573017
725241000006116	B08	B08..00	Malignant neoplasm of hypopharynx	Head and Neck	363399006	482598016
288470014	B0550	B055000	Malignant neoplasm of junction of hard and soft palate	Head and Neck	187666008	288470014
724781000006117	B0z2	B0z2.00	Malignant neoplasm of laryngopharynx	Head and Neck	363399006	1228533018
288807015	B21z	B21z.00	Malignant neoplasm of larynx NOS	Head and Neck	363429002	482656014
288806012	B21y	B21y.00	Malignant neoplasm of larynx, other specified site	Head and Neck	363429002	482656014
288444013	B041	B041.00	Malignant neoplasm of lateral portion of floor of mouth	Head and Neck	187653008	288444013
724841000006116	B072	B072.00	Malignant neoplasm of lateral wall of nasopharynx	Head and Neck	363398003	482595018
724891000006113	B00	B00..00	Malignant neoplasm of lip	Head and Neck	363348004	482495016
724901000006112	B0040	B004000	Malignant neoplasm of lip unspecified, buccal aspect	Head and Neck	187622006	288392012
288391017	B0042	B004200	Malignant neoplasm of lip unspecified, mucosa	Head and Neck	187622006	288392012
288393019	B0043	B004300	Malignant neoplasm of lip, oral aspect	Head and Neck	187622006	288393019
406052010	B0	B0...00	Malignant neoplasm of lip, oral cavity and pharynx	Head and Neck	271323007	406052010
288582015	B0zz	B0zz.00	Malignant neoplasm of lip, oral cavity and pharynx NOS	Head and Neck	271323007	406052010
288408015	B00z0	B00z000	Malignant neoplasm of lip, unspecified, external	Head and Neck	363348004	482495016
288410018	B00zz	B00zz00	Malignant neoplasm of lip, vermilion border NOS	Head and Neck	421249001	2618242015
288459019	B0511	B051100	Malignant neoplasm of lower buccal sulcus	Head and Neck	187660002	288459019
724311000006111	B031	B031.00	Malignant neoplasm of lower gum	Head and Neck	363384006	482567018
288380012	B0030	B003000	Malignant neoplasm of lower lip, buccal aspect	Head and Neck	187613005	288380012
288354011	B0010	B001000	Malignant neoplasm of lower lip, external	Head and Neck	187604008	288354011
288382016	B0031	B003100	Malignant neoplasm of lower lip, frenulum	Head and Neck	187614004	288382016
406399011	B003	B003.00	Malignant neoplasm of lower lip, inner aspect	Head and Neck	271568003	406399011
288387010	B003z	B003z00	Malignant neoplasm of lower lip, inner aspect NOS	Head and Neck	271568003	406398015
1228506017	B0011	B001100	Malignant neoplasm of lower lip, lipstick area	Head and Neck	363373004	1228506017
724421000006114	B001	B001.00	Malignant neoplasm of lower lip, vermilion border	Head and Neck	363373004	1228505018
724641000006117	B202	B202.00	Malignant neoplasm of maxillary sinus	Head and Neck	363425008	482648011
288474017	B05z	B05z.00	Malignant neoplasm of mouth NOS	Head and Neck	363505006	482796019
1228552012	B200	B200.00	Malignant neoplasm of nasal cavities	Head and Neck	363422006	1228552012
288776016	B200z	B200z00	Malignant neoplasm of nasal cavities NOS	Head and Neck	363422006	482642012
729321000006119	B20	B20..00	Malignant neoplasm of nasal cavities, middle ear and accessory sinuses	Head and Neck	187828007	288770010
288772019	B2001	B200100	Malignant neoplasm of nasal conchae	Head and Neck	187830009	288772019

288522018	B07	B07..00	Malignant neoplasm of nasopharynx	Head and Neck	187692001	288522018
288450015	B05	B05..00	Malignant neoplasm of other and unspecified parts of mouth	Head and Neck	363505006	482796019
723801000006115	B0zy	B0zy.00	Malignant neoplasm of other sites lip, oral cavity, pharynx	Head and Neck	271323007	406052010
288448011	B04y	B04y.00	Malignant neoplasm of other sites of floor of mouth	Head and Neck	363385007	482569015
288439018	B03y	B03y.00	Malignant neoplasm of other sites of gum	Head and Neck	363382005	482560016
288434011	B01y	B01y.00	Malignant neoplasm of other sites of tongue	Head and Neck	363375006	482546018
288570012	B08y	B08y.00	Malignant neoplasm of other specified hypopharyngeal site	Head and Neck	363399006	482597014
288473011	B05y	B05y.00	Malignant neoplasm of other specified mouth parts	Head and Neck	363505006	482796019
288563012	B07y	B07y.00	Malignant neoplasm of other specified site of nasopharynx	Head and Neck	187692001	288519015
288404018	B006	B006.00	Malignant neoplasm of overlapping lesion of lip	Head and Neck	187624007	288404018
288471013	B055z	B055z00	Malignant neoplasm of palate NOS	Head and Neck	363390005	482579018
288532013	B0711	B071100	Malignant neoplasm of pharyngeal tonsil	Head and Neck	187694000	288532013
288573014	B0z0	B0z0.00	Malignant neoplasm of pharynx unspecified	Head and Neck	363507003	482800013
288529010	B071	B071.00	Malignant neoplasm of posterior wall of nasopharynx	Head and Neck	187693006	288529010
722421000006115	B081	B081.00	Malignant neoplasm of pyriform sinus	Head and Neck	363401000	482602019
1228523014	B0551	B055100	Malignant neoplasm of roof of mouth	Head and Neck	363390005	1228523014
1228532011	B070	B070.00	Malignant neoplasm of roof of nasopharynx	Head and Neck	363397008	1228532011
721721000006119	B205	B205.00	Malignant neoplasm of sphenoidal sinus	Head and Neck	363428005	482654012
721871000006110	B212	B212.00	Malignant neoplasm of subglottis	Head and Neck	363430007	482658010
288797015	B211	B211.00	Malignant neoplasm of supraglottis	Head and Neck	187842004	288797015
288435012	B01z	B01z.00	Malignant neoplasm of tongue NOS	Head and Neck	363375006	482546018
288433017	B015	B015.00	Malignant neoplasm of tongue, junctional zone	Head and Neck	187644001	288433017
288419017	B012	B012.00	Malignant neoplasm of tongue, tip and lateral border	Head and Neck	187637005	288419017
288454012	B0510	B051000	Malignant neoplasm of upper buccal sulcus	Head and Neck	187659007	288454012
288372017	B0021	B002100	Malignant neoplasm of upper lip, frenulum	Head and Neck	187608006	288372017
288363013	B002	B002.00	Malignant neoplasm of upper lip, inner aspect	Head and Neck	187606005	288363013
288377011	B002z	B002z00	Malignant neoplasm of upper lip, inner aspect NOS	Head and Neck	187606005	288356013
288350019	B0001	B000100	Malignant neoplasm of upper lip, lipstick area	Head and Neck	187601000	288350019
288364019	B0022	B002200	Malignant neoplasm of upper lip, mucosa	Head and Neck	187606005	288364019
288362015	B0023	B002300	Malignant neoplasm of upper lip, oral aspect	Head and Neck	187606005	288362015
1228504019	B000	B000.00	Malignant neoplasm of upper lip, vermilion border	Head and Neck	363372009	1228504019
155669014	B013	B013.00	Malignant neoplasm of ventral surface of tongue	Head and Neck	94134006	155669014
288453018	B051	B051.00	Malignant neoplasm of vestibule of mouth	Head and Neck	187658004	288453018
288775017	B2003	B200300	Malignant neoplasm of vestibule of nose	Head and Neck	187831008	288775017
1219533011	B042	B042.00	Malignant neoplasm, overlapping lesion of floor of mouth	Head and Neck	109830000	1219533011
1219468014	B214	B214.00	Malignant neoplasm, overlapping lesion of larynx	Head and Neck	109369002	1219468014
1219531013	B017	B017.00	Malignant overlapping lesion of tongue	Head and Neck	109823006	1219531013
288449015	B04z	B04z.00	Malignant tumour of floor of mouth	Head and Neck	363385007	482570019
289425014	B5500	B550000	Malignant tumour of head and neck	Head and Neck	255056009	380072014
288571011	B08z	B08z.00	Malignant tumour of hypopharynx	Head and Neck	363399006	482598016
724791000006119	B21	B21..00	Malignant tumour of larynx	Head and Neck	363429002	482656014
288550018	B072z	B072z00	Malignant tumour of lateral wall of nasopharynx	Head and Neck	363398003	482595018

453301018	B007	B007.00	Malignant tumour of lip	Head and Neck	363348004	482495016
288409011	B00z1	B00z100	Malignant tumour of lipstick area of lip	Head and Neck	275399006	411376018
288792014	B20z	B20z.00	Malignant tumour of nasal sinuses	Head and Neck	363506007	482798018
6247711000006111	^ESCTMA624771		Malignant tumour of oral cavity	Head and Neck	363505006	482797011
288469013	B055	B055.00	Malignant tumour of palate	Head and Neck	363390005	482579018
720491000006119	B0732	B073200	Malignant tumour of posterior margin of nasal septum and choanae	Head and Neck	254484001	378896011
721311000006114	B01	B01..00	Malignant tumour of tongue	Head and Neck	363375006	482546018
720801000006112	B0000	B000000	Malignant tumour of vermilion border of lip	Head and Neck	421249001	2618242015
288355012	B001z	B001z00	Malignant tumour of vermilion border of lower lip	Head and Neck	363373004	482542016
288351015	B000z	B000z00	Malignant tumour of vermilion border of upper lip	Head and Neck	363372009	482541011
5592891000006111	^ESCTSQ559289		Squamous cell carcinoma of tongue	Head and Neck	276952000	413333011
879691000006112	B01-99	B01..99	Tongue carcinoma	Head and Neck	363375006	879691000006112
725861000006118	B03	B03..00	Malignant neoplasm of gum	Head and Neck	363382005	482560016
288440016	B03z	B03z.00	Malignant neoplasm of gum NOS	Head and Neck	363382005	482560016
720761000006115	B030	B030.00	Malignant neoplasm of upper gum	Head and Neck	363383000	482562012
288564018	B07z	B07z.00	Malignant tumour of nasopharynx	Head and Neck	187692001	288526015
288541015	B071z	B071z00	Malignant tumour of posterior wall of nasopharynx	Head and Neck	187693006	288528019
1224870013	BB5D5	BB5D500	[M]Hepatocellular carcinoma NOS	HPB	25370001	1224870013
309871000006116	BB5D5-1	BB5D511	[M]Hepatoma NOS	HPB	25370001	1224870013
309891000006115	BB5D5-2	BB5D512	[M]Hepatoma, malignant	HPB	25370001	1224870013
7534911000006110	^ESCTAD753491		Adenocarcinoma of pancreas	HPB	700423003	2990575018
380139012	B1612-1	B161211	Carcinoma common bile duct	HPB	255086002	380139012
535221000006113	B160-1	B160.11	Carcinoma of gallbladder	HPB	372140005	1210716013
306641000006116	BB5D1	BB5D100	Cholangiocarcinoma	HPB	70179006	116557016
6012841000006119	^ESCTCH601284		Cholangiocarcinoma	HPB	312104005	2310231000000118
342834014	B1501	B150100	Hepatoblastoma of liver	HPB	109843000	342834014
342807011	B1503	B150300	Hepatocellular carcinoma	HPB	109841003	342807011
292101010	Byu11	Byu1100	Liver carcinoma	HPB	109841003	342812012
11919851000006115	^ESCT1191985		Liver cell carcinoma	HPB	109841003	342811017
727781000006114	B16	B16..00	Malignant neoplasm gallbladder and extrahepatic bile ducts	HPB	363415003	482629012
727791000006112	B16z	B16z.00	Malignant neoplasm gallbladder/extrahepatic bile ducts NOS	HPB	363415003	482629012
727911000006116	B162	B162.00	Malignant neoplasm of ampulla of Vater	HPB	363417006	482633017
288717016	B171	B171.00	Malignant neoplasm of body of pancreas	HPB	187791002	288717016
726361000006119	B1612	B161200	Malignant neoplasm of common bile duct	HPB	93763008	155198014
288734012	B17y0	B17y000	Malignant neoplasm of ectopic pancreatic tissue	HPB	187798008	288734012
288694017	B1510	B151000	Malignant neoplasm of interlobular bile ducts	HPB	187773007	288694017
155305011	B151	B151.00	Malignant neoplasm of intrahepatic bile ducts	HPB	187777008	288698019
288699010	B151z	B151z00	Malignant neoplasm of intrahepatic bile ducts NOS	HPB	187777008	288698019
2619456011	B1512	B151200	Malignant neoplasm of intrahepatic biliary passages	HPB	187777008	2619456011
288698019	B1514	B151400	Malignant neoplasm of intrahepatic gall duct	HPB	187777008	288698019
288700011	B152	B152.00	Malignant neoplasm of liver	HPB	93870000	155343014
288688013	B15	B15..00	Malignant neoplasm of liver and intrahepatic bile ducts	HPB	187767006	288688013
288702015	B15z	B15z.00	Malignant neoplasm of liver and intrahepatic bile ducts NOS	HPB	187767006	288688013
288731016	B17y	B17y.00	Malignant neoplasm of other specified sites of pancreas	HPB	363418001	482635012

288736014	B17z	B17z.00	Malignant neoplasm of pancreas NOS	HPB	363418001	482635012
288723014	B173	B173.00	Malignant neoplasm of pancreatic duct	HPB	187793004	288723014
288735013	B17yz	B17yz00	Malignant neoplasm of specified site of pancreas NOS	HPB	363418001	482635012
288720012	B172	B172.00	Malignant neoplasm of tail of pancreas	HPB	187792009	288720012
720451000006113	B16y	B16y.00	Malignant neoplasm other gallbladder/extrahepatic bile duct	HPB	363415003	482629012
1219538019	B175	B175.00	Malignant neoplasm, overlapping lesion of pancreas	HPB	109848009	1219538019
726201000006111	B161	B161.00	Malignant tumour of extrahepatic bile duct	HPB	363416002	482631015
725711000006113	B160	B160.00	Malignant tumour of gallbladder	HPB	363353009	482506012
725121000006118	B170	B170.00	Malignant tumour of head of pancreas	HPB	363419009	482638014
723401000006118	B17	B17..00	Malignant tumour of pancreas	HPB	363418001	482635012
288690014	B1500	B150000	Primary carcinoma of liver	HPB	187769009	288690014
157732017	B150	B150.00	Primary malignant neoplasm of liver	HPB	95214007	157732017
288692018	B150z	B150z00	Primary malignant neoplasm of liver NOS	HPB	95214007	157732017
155207017	B1610	B161000	Malignant neoplasm of cystic duct	HPB	93770008	155207017
288707014	B1611	B161100	Malignant neoplasm of hepatic duct	HPB	187784000	288707014
288727010	B174	B174.00	Malignant neoplasm of Islets of Langerhans	HPB	187794005	288727010
1216160012	BBr01	BBr0100	[M]Acute leukaemia NOS	Leukaemia	24072005	1216160012
1216729017	BBrA4	BBrA400	[M]Hairy cell leukaemia	Leukaemia	54087003	1216729017
291985019	BBrz	BBrz.00	[M]Leukaemia NOS	Leukaemia	128931003	207615015
1222221019	BBgG-2	BBgG.12	[M]Lymphoblastic lymphoma NOS	Leukaemia	115244002	202936014
292185016	ByuD9	ByuD900	[X]Other leukaemia of unspecified cell type	Leukaemia	93143009	154099013
292177013	ByuD5	ByuD500	[X]Other lymphoid leukaemia	Leukaemia	188725004	289876019
292181013	ByuD7	ByuD700	[X]Other monocytic leukaemia	Leukaemia	188744006	289917018
292178015	ByuD6	ByuD600	[X]Other myeloid leukaemia	Leukaemia	188732008	289892012
292182018	ByuD8	ByuD800	[X]Other specified leukaemias	Leukaemia	93143009	154099013
456641000006119	B670	B670.00	Acute erythraemia and erythroleukaemia	Leukaemia	93451002	154686014
289964018	B680	B680.00	Acute leukaemia	Leukaemia	91855006	201610019
292006015	BBr21	BBr2100	Acute lymphoblastic leukaemia - category	Leukaemia	413440007	2535405016
509741017	B640	B640.00	Acute lymphoid leukaemia	Leukaemia	91857003	509741017
2534415018	B660	B660.00	Acute monocytic leukaemia	Leukaemia	413441006	2534415018
344846014	B675	B675.00	Acute myelofibrosis	Leukaemia	109991003	344846014
201612010	B650	B650.00	Acute myeloid leukaemia	Leukaemia	91861009	201612010
304401000006111	BBr61	BBr6100	Acute myeloid leukaemia	Leukaemia	17788007	478421017
3992711000006112	^ESCTAC399271		Acute myeloid leukaemia, disease	Leukaemia	91861009	1207830016
133678011	B674	B674.00	Acute panmyelosis	Leukaemia	109991003	2532080014
289885019	B64y2	B64y200	Adult T-cell leukaemia	Leukaemia	188729005	289885019
459741000006115	B65y1	B65y100	APL - Acute promyelocytic leukaemia	Leukaemia	110004001	1215869018
414160018	B6400	B640000	B-cell acute lymphoblastic leukaemia	Leukaemia	277571004	414160018
414008012	B6410	B641000	B-cell chronic lymphocytic leukaemia	Leukaemia	277473004	414008012
289903010	B6530	B653000	Chloroma	Leukaemia	188737002	289903010
554641000006115	B651-1	B651.11	Chronic granulocytic leukaemia	Leukaemia	92818009	1217279014
289967013	B681	B681.00	Chronic leukaemia	Leukaemia	92812005	201651017
2168681000000113	B6410-1	B641011	Chronic lymphocytic leukaemia of B-cell type	Leukaemia	277473004	2168681000000113
201652012	B641	B641.00	Chronic lymphoid leukaemia	Leukaemia	92814006	201652012
306931000006115	BBr23	BBr2300	Chronic lymphoid leukaemia	Leukaemia	51092000	495910011
555131000006118	B641-1	B641.11	Chronic lymphoid leukaemia, disease	Leukaemia	92814006	1207831017
289923011	B661	B661.00	Chronic monocytic leukaemia	Leukaemia	188745007	289923011
201656010	B651	B651.00	Chronic myeloid leukaemia	Leukaemia	92818009	201656010
289900013	B651z	B651z00	Chronic myeloid leukaemia NOS	Leukaemia	92818009	201656010
474077018	B691	B691.00	Chronic myelomonocytic leukaemia	Leukaemia	127225006	474077018
306971000006117	BBr68	BBr6800	Chronic myelomonocytic leukaemia	Leukaemia	128831004	474620018
289898011	B6512	B651200	Chronic neutrophilic leukaemia	Leukaemia	188734009	289898011
2233381000000115	B6411	B641100	Clinical stage A chronic lymphocytic leukaemia	Leukaemia	863741000000108	2233381000000115

201658011	B670-1	B670.11	Di Guglielmo's disease	Leukaemia	93451002	154686014
251577010	1429	1429.00	H/O: leukaemia	Leukaemia	161436008	2532140019
1591211000006111	B624-2	B624.12	Hairy cell leukaemia	Leukaemia	118613001	2764402015
289972016	B68z	B68z.00	Leukaemia	Leukaemia	93143009	154099013
289963012	B68	B68..00	Leukaemia of unspecified cell type	Leukaemia	93143009	154099013
745001000006114	B624	B624.00	Leukaemic reticuloendotheliosis	Leukaemia	118613001	1215903015
745011000006112	B624-1	B624.11	Leukaemic reticuloendotheliosis	Leukaemia	118613001	1215903015
289773015	B624z	B624z00	Leukaemic reticuloendotheliosis NOS	Leukaemia	118613001	1215903015
746301000006117	B6243	B624300	Leukaemic reticuloendotheliosis of intra-abdominal lymph nodes	Leukaemia	93144003	510216012
289756017	B6240	B624000	LRE - Leukaemic reticuloendotheliosis	Leukaemia	118613001	1215903015
289875015	B64-1	B64..11	Lymphatic leukaemia	Leukaemia	188725004	289875015
289876019	B64	B64..00	Lymphoid leukaemia	Leukaemia	188725004	289876019
880821000006114	B640-99	B640.99	Lymphoid leukaemia - acute	Leukaemia	91857003	880821000006114
880831000006112	B641-99	B641.99	Lymphoid leukaemia - chronic	Leukaemia	92814006	880831000006112
289889013	B64z	B64z.00	Lymphoid leukaemia NOS	Leukaemia	188725004	289876019
731621000006113	B67y0	B67y000	Lymphosarcoma cell leukaemia	Leukaemia	188725004	289876019
458201000006115	B690	B690.00	M4 - Acute myelomonocytic leukaemia	Leukaemia	110005000	1215872013
716871000006112	B673	B673.00	Mast cell leukaemia	Leukaemia	110002002	2765332017
289949018	B672	B672.00	Megakaryocytic leukaemia	Leukaemia	188754005	289949018
289919015	B66-2	B66..12	Monoblastic leukaemia	Leukaemia	188744006	289919015
289917018	B66	B66..00	Monocytic leukaemia	Leukaemia	188744006	289917018
289933015	B66z	B66z.00	Monocytic leukaemia NOS	Leukaemia	188744006	289917018
289892012	B65	B65..00	Myeloid leukaemia	Leukaemia	188732008	289892012
880851000006117	B650-99	B650.99	Myeloid leukaemia - acute	Leukaemia	91861009	880851000006117
880861000006115	B651-99	B651.99	Myeloid leukaemia - chronic	Leukaemia	92818009	880861000006115
289912012	B65z	B65z.00	Myeloid leukaemia NOS	Leukaemia	188732008	289892012
157091019	B653	B653.00	Myeloid sarcoma	Leukaemia	94719007	157091019
289974015	B69	B69..00	Myelomonocytic leukaemia	Leukaemia	188768003	289974015
289955011	B67y	B67y.00	Other and unspecified leukaemia	Leukaemia	93143009	154099013
289959017	B67yz	B67yz00	Other and unspecified leukaemia NOS	Leukaemia	93143009	154099013
289971011	B68y	B68y.00	Other leukaemia of unspecified cell type	Leukaemia	93143009	154099013
289881011	B64y	B64y.00	Other lymphoid leukaemia	Leukaemia	188725004	289876019
289888017	B64yz	B64yz00	Other lymphoid leukaemia NOS	Leukaemia	188725004	289876019
289926015	B66y	B66y.00	Other monocytic leukaemia	Leukaemia	188744006	289917018
289930017	B66yz	B66yz00	Other monocytic leukaemia NOS	Leukaemia	188744006	289917018
289911017	B65yz	B65yz00	Other myeloid leukaemia NOS	Leukaemia	188732008	289892012
289934014	B67	B67..00	Other specified leukaemia	Leukaemia	93143009	154099013
289960010	B67z	B67z.00	Other specified leukaemia NOS	Leukaemia	93143009	154099013
202801000006110	B64y1	B64y100	Prolymphocytic leukaemia	Leukaemia	110006004	2765132011
289968015	B682	B682.00	Subacute leukaemia	Leukaemia	302855005	444829010
289880012	B642	B642.00	Subacute lymphoid leukaemia	Leukaemia	188726003	289880012
289924017	B662	B662.00	Subacute monocytic leukaemia	Leukaemia	188746008	289924017
289901012	B652	B652.00	Subacute myeloid leukaemia	Leukaemia	188736006	289901012
289980011	B692	B692.00	Subacute myelomonocytic leukaemia	Leukaemia	188770007	289980011
289948014	B672-1	B672.11	Thrombocytic leukaemia	Leukaemia	188754005	289948014
6841421000006114	^ESCTAD684142		Adenocarcinoma of caecum	LGI	413446001	2535206017
6765141000006116	^ESCTAD676514		Adenocarcinoma of large intestine	LGI	408645001	2160192016
5346461000006110	^ESCTAD534646		Adenocarcinoma of rectum	LGI	254582000	379090011
5898351000006111	^ESCTAD589835		Adenocarcinoma of sigmoid colon	LGI	301756000	443200011
380137014	B142-1	B142.11	Anal carcinoma	LGI	363352004	482503016
1228540017	B133-99	B133.99	Ca sigmoid colon	LGI	363410008	879861000006115
841331000006115	B1z0-1	B1z0.11	Cancer of bowel	LGI	363508008	1228620018
6245011000006115	^ESCTCA624501		Cancer of colon	LGI	363406005	1228536014
380129018	B134-1	B134.11	Carcinoma of caecum	LGI	255081007	380129018
5512021000006115	^ESCTCA551202		Carcinoma of colon	LGI	269533000	403809019

379088010	B141-1	B141.11	Carcinoma of rectum	LGI	254582000	379090011
5699751000006114	^ESCTCA569975		Carcinoma of sigmoid colon	LGI	285312008	424280011
573611000006118	B13z-1	B13z.11	Colonic cancer	LGI	363406005	482611019
15263681000006114	^ESCT1526368		Colorectal cancer	LGI	1286877004	5255860018
1488641018	4M10	4M10.00	Dukes stage A	LGI	394939004	1488641018
1488642013	4M11	4M11.00	Dukes stage B	LGI	394940002	1488642013
1488643015	4M12	4M12.00	Dukes stage C1	LGI	394941003	1488643015
729581000006111	B14y	B14y.00	Malig neop other site rectum, rectosigmoid junction and anus	LGI	187760008	288675010
727281000006117	B135	B135.00	Malignant neoplasm of appendix	LGI	363411007	482621010
1216464010	B134	B134.00	Malignant neoplasm of caecum	LGI	363350007	1216464010
726331000006111	B13	B13..00	Malignant neoplasm of colon	LGI	363406005	3446619012
288749016	B18y1	B18y100	Malignant neoplasm of mesocaecum	LGI	187810005	288749016
288751017	B18y2	B18y200	Malignant neoplasm of mesorectum	LGI	187811009	288751017
288671018	B13y	B13y.00	Malignant neoplasm of other specified sites of colon	LGI	363406005	482611019
288675010	B14	B14..00	Malignant neoplasm of rectum, rectosigmoid junction and anus	LGI	187760008	288675010
721821000006114	B137	B137.00	Malignant neoplasm of splenic flexure of colon	LGI	363413005	482625018
720501000006110	B14z	B14z.00	Malignant neoplasm rectum,rectosigmoid junction and anus NOS	LGI	187760008	288675010
288670017	B138	B138.00	Malignant neoplasm, overlapping lesion of colon	LGI	187757001	288670017
155076015	B142	B142.00	Malignant tumour of anal canal	LGI	363352004	482503016
288685011	B143	B143.00	Malignant tumour of anus	LGI	363490009	482768013
6245171000006113	^ESCTMA624517		Malignant tumour of appendix	LGI	363411007	482622015
727321000006111	B136	B136.00	Malignant tumour of ascending colon	LGI	363412000	482623013
6242891000006114	^ESCTMA624289		Malignant tumour of caecum	LGI	363350007	482499010
403682011	B13z	B13z.00	Malignant tumour of colon	LGI	363406005	482611019
725951000006112	B132	B132.00	Malignant tumour of descending colon	LGI	363409003	482618013
725191000006116	B130	B130.00	Malignant tumour of hepatic flexure	LGI	363407001	482614010
722441000006110	B140	B140.00	Malignant tumour of rectosigmoid junction	LGI	363414004	482627014
155488014	B141	B141.00	Malignant tumour of rectum	LGI	363351006	482502014
6242981000006116	^ESCTMA624298		Malignant tumour of rectum	LGI	363351006	6242981000006116
721991000006115	B133	B133.00	Malignant tumour of sigmoid colon	LGI	363410008	482619017
721421000006112	B131	B131.00	Malignant tumour of transverse colon	LGI	363408006	482615011
7970001000006114	^ESCTPR797000		Primary adenocarcinoma of colon	LGI	1701000119104	2915311014
6243001000006117	^ESCTRE624300		Rectal cancer	LGI	363351006	1228486017
379089019	B141-2	B141.12	Rectal carcinoma	LGI	254582000	379090011
306131000006118	BB5S2-2	BB5S212	[M]Bronchiolar carcinoma	Lung	112677002	1219789016
1219789016	BB5S2	BB5S200	[M]Bronchiolo-alveolar adenocarcinoma	Lung	112677002	1219789016
1773151000006112	EMISNQAD45		Adenocarcinoma of lung	Lung	254626006	379172013
9868091000006114	^ESCTAD986809		Adenocarcinoma of right lung	Lung	15956381000119100	3317337019
880051000006113	B222-99	B222.99	Ca upper lobe bronchus/lung	Lung	269464000	880051000006113
1772971000006112	EMISNQCA85		Carcinoid tumour of lung	Lung	254627002	379173015
7362951000006113	^ESCTCA736295		Carcinoma of lung	Lung	448993007	2901727017
733371000006119	B22z-1	B22z.11	Lung cancer	Lung	93880001	3288586014
292105018	Byu20	Byu2000	Malignant neoplasm of bronchus or lung	Lung	1090881000000100	2732641000000116
288813012	B2210	B221000	Malignant neoplasm of carina of bronchus	Lung	187857006	288813012
155287019	B2211	B221100	Malignant neoplasm of hilus of lung	Lung	93827000	155287019
288827019	B2240	B224000	Malignant neoplasm of lower lobe bronchus	Lung	187869003	288827019
288828012	B2241	B224100	Malignant neoplasm of lower lobe of lung	Lung	187870002	288828012
288826011	B224	B224.00	Malignant neoplasm of lower lobe, bronchus or lung	Lung	187868006	288826011
288829016	B224z	B224z00	Malignant neoplasm of lower lobe, bronchus or lung NOS	Lung	187868006	288826011

288808013	B22	B22..00	Malignant neoplasm of lower respiratory tract	Lung	430621000	2765453013
155361017	B221	B221.00	Malignant neoplasm of main bronchus	Lung	372065009	1210643012
288815017	B221z	B221z00	Malignant neoplasm of main bronchus NOS	Lung	372065009	1210643012
288823015	B2230	B223000	Malignant neoplasm of middle lobe bronchus	Lung	187865009	288823015
288824014	B2231	B223100	Malignant neoplasm of middle lobe of lung	Lung	187866005	288824014
288822013	B223	B223.00	Malignant neoplasm of middle lobe, bronchus or lung	Lung	187864008	288822013
288825010	B223z	B223z00	Malignant neoplasm of middle lobe, bronchus or lung NOS	Lung	187864008	288822013
288832018	B22y	B22y.00	Malignant neoplasm of other sites of bronchus or lung	Lung	363358000	482516016
723301000006110	B225	B225.00	Malignant neoplasm of overlapping lesion of bronchus and lung	Lung	109371002	1219469018
288819011	B2220	B222000	Malignant neoplasm of upper lobe bronchus	Lung	187861000	288819011
288820017	B2221	B222100	Malignant neoplasm of upper lobe of lung	Lung	187862007	288820017
403688010	B222	B222.00	Malignant neoplasm of upper lobe, bronchus or lung	Lung	269464000	403688010
288821018	B222z	B222z00	Malignant neoplasm of upper lobe, bronchus or lung NOS	Lung	269464000	403688010
403689019	B22z	B22z.00	Malignant tumour of lung	Lung	363358000	482516016
1773131000006117	EMISNQNO51		Non-small cell lung cancer	Lung	254637007	379195016
1773111000006111	EMISNQPR69		Primary malignant neoplasm of lung	Lung	93880001	510792012
1773121000006115	EMISNQSM10		Small cell lung cancer	Lung	254632001	379181019
1773141000006110	EMISNQSQ1		Squamous cell carcinoma of lung	Lung	254634000	379184010
1225763012	BBmD	BBmD.00	[M] Cutaneous lymphoma	Lymphoma	28054005	1225763012
1221109014	BBj	BBj..00	[M]Hodgkin's disease	Lymphoma	14537002	1221109014
1222219012	BBgC-1	BBgC.11	[M]Lymphocytic lymphoma NOS	Lymphoma	115244002	202936014
311431000006119	BBg1-1	BBg1.11	[M]Lymphoma NOS	Lymphoma	21964009	36846011
291887011	BBg	BBg..00	[M]Lymphomas, NOS or diffuse	Lymphoma	414628006	2535396014
291891018	BBg10	BBg1000	[M]Malignant lymphoma, diffuse NOS	Lymphoma	115244002	202936014
1230392016	BBgR	BBgR.00	[M]Malignant lymphoma, large cell, diffuse NOS	Lymphoma	46732000	1230392016
1222289015	BBg7	BBg7.00	[M]Malignant lymphoma, lymphoplasmacytoid type	Lymphoma	19340000	1222289015
311871000006119	BBg2	BBg2.00	[M]Malignant lymphoma, non-Hodgkin's type	Lymphoma	1929004	1222276014
314061000006117	BBg2-1	BBg2.11	[M]Non-Hodgkin's lymphoma	Lymphoma	1929004	1222274012
400011000006119	ByuDF-1	ByuDF11	[X]Non-Hodgkin's lymphoma NOS	Lymphoma	118601006	2663475013
292169014	ByuD0	ByuD000	[X]Other Hodgkin's disease	Lymphoma	118599009	2663473018
292174018	ByuD3	ByuD300	[X]Other specified types of non-Hodgkin's lymphoma	Lymphoma	118601006	177020011
292173012	ByuD2	ByuD200	[X]Other types of diffuse non-Hodgkin's lymphoma	Lymphoma	109962001	174656018
292170010	ByuD1	ByuD100	[X]Other types of follicular non-Hodgkin's lymphoma	Lymphoma	308121000	451429010
292193016	ByuDE	ByuDE00	[X]Unspecified B-cell non-Hodgkin's lymphoma	Lymphoma	1091921000000103	2734911000000119
11903061000006113	^ESCT1190306		B-cell lymphoma (clinical)	Lymphoma	109979007	174675011
82041000006113	B627W	B627W00	B-cell non-Hodgkin's lymphoma	Lymphoma	1091921000000103	2734911000000119
2663190016	B602	B602.00	Burkitt's lymphoma	Lymphoma	118617000	2663190016
289612010	B602z	B602z00	Burkitt's lymphoma NOS	Lymphoma	118617000	2663190016
289605018	B6023	B602300	Burkitt's lymphoma of intra-abdominal lymph nodes	Lymphoma	188512009	289605018
289604019	B6022	B602200	Burkitt's lymphoma of intrathoracic lymph nodes	Lymphoma	188511002	289604019
289603013	B6021	B602100	Burkitt's lymphoma of lymph nodes of head, face and neck	Lymphoma	188510001	289603013

527801000006112	B6025	B602500	Burkitt's lymphoma of lymph nodes of inguinal region and lower limb	Lymphoma	188514005	289607014
3635398013	^ESCT1169431		Classical Hodgkin lymphoma	Lymphoma	762690000	3635398013
1815331000006113	B62E3	B62E300	Cutaneous T-cell lymphoma	Lymphoma	400122007	1787104010
39671000006114	B62xX	B62xX00	Cutaneous/peripheral T-cell lymphoma	Lymphoma	277613000	414240018
198006010	B627E	B627E00	Diffuse large B-cell lymphoma	Lymphoma	847741000000106	2197851000000117
444799014	B627D	B627D00	Diffuse non-Hodgkin's centroblastic lymphoma	Lymphoma	302842009	444799014
1219545019	B6276	B627600	Diffuse non-Hodgkin's immunoblastic (diffuse) lymphoma	Lymphoma	109966003	1219545019
1668101000000112	B627A	B627A00	Diffuse non-Hodgkin's large cell lymphoma	Lymphoma	109969005	1668101000000112
292191019	ByuDC	ByuDC00	Diffuse non-Hodgkin's lymphoma	Lymphoma	109962001	174656018
289812013	B6278	B627800	Diffuse non-Hodgkin's lymphoma undifferentiated (diffuse)	Lymphoma	188679001	289812013
621071000006112	B6277	B627700	Diffuse non-Hodgkin's lymphoma, lymphoblastic (clinical)	Lymphoma	109965004	174660015
621091000006113	B627X	B627X00	Diffuse non-Hodgkin's lymphoma, unspecified	Lymphoma	109962001	174656018
621041000006116	B6275	B627500	Diffuse non-Hodgkin's mixed small and large cell (diffuse) lymphoma	Lymphoma	188676008	289807012
289799010	B6273	B627300	Diffuse non-Hodgkin's small cell (diffuse) lymphoma	Lymphoma	188674006	289799010
1815151000006116	B628	B628.00	Follicular lymphoma	Lymphoma	308121000	451429010
1815161000006119	B6280	B628000	Follicular lymphoma grade 1	Lymphoma	847481000000109	2197241000000116
1815171000006114	B6281	B628100	Follicular lymphoma grade 2	Lymphoma	847631000000107	2197621000000114
763421000006116	B627C-1	B627C11	Follicular lymphoma NOS	Lymphoma	308121000	451429010
414278016	B6272	B627200	Follicular non-Hodgkin's large cell lymphoma	Lymphoma	277641001	414278016
451429010	B627C	B627C00	Follicular non-Hodgkin's lymphoma	Lymphoma	308121000	451429010
763431000006118	B6271	B627100	Follicular non-Hodgkin's mixed small cleaved and large cell lymphoma	Lymphoma	188672005	289795016
414257019	B6270	B627000	Follicular non-Hodgkin's small cleaved cell lymphoma	Lymphoma	277625002	414257019
5602241000006114	^ESCTHI560224		High grade B-cell lymphoma	Lymphoma	277617004	414245011
1815031000006110	B61-1	B61..11	Hodgkin lymphoma	Lymphoma	118599009	2663473018
2730231000006116	^ESCTHO273023		Hodgkin lymphoma	Lymphoma	14537002	195620018
826681000006115	B610	B610.00	Hodgkin lymphoma, nodular lymphocyte predominance (clinical)	Lymphoma	118605002	202988019
215914013	BBj6	BBj6.00	Hodgkin lymphoma, nodular sclerosis	Lymphoma	52248008	198368015
2663473018	B61	B61..00	Hodgkin's disease	Lymphoma	118599009	2663473018
1815101000006115	B61z-1	B61z.11	Hodgkin's disease (clinical)	Lymphoma	118599009	177017015
826121000006114	B61z	B61z.00	Hodgkin's disease NOS	Lymphoma	118599009	2663473018
289702012	B61zz	B61zz00	Hodgkin's disease NOS	Lymphoma	118599009	2663473018
289703019	B61z0	B61z000	Hodgkin's disease NOS, unspecified site	Lymphoma	118599009	177017015
289705014	B61z2	B61z200	Hodgkin's disease of intrathoracic lymph nodes	Lymphoma	93522004	154818017
826181000006113	B61z4	B61z400	Hodgkin's disease of lymph nodes of axilla AND/OR upper limb	Lymphoma	93523009	154820019
289704013	B61z1	B61z100	Hodgkin's disease of lymph nodes of head, face AND/OR neck	Lymphoma	93524003	154823017
826171000006110	B61z5	B61z500	Hodgkin's disease of lymph nodes of inguinal region AND/OR lower limb	Lymphoma	93525002	154826013
289711012	B61z8	B61z800	Hodgkin's disease of lymph nodes of multiple sites	Lymphoma	93526001	154829018
289710013	B61z7	B61z700	Hodgkin's disease of spleen	Lymphoma	93527005	154831010
2663187010	B616	B616.00	Hodgkin's disease, lymphocytic depletion	Lymphoma	118610003	2663187010
289701017	B616z	B616z00	Hodgkin's disease, lymphocytic depletion NOS	Lymphoma	118610003	177029012
826471000006116	B6164	B616400	Hodgkin's disease, lymphocytic depletion of lymph nodes of axilla and upper limb	Lymphoma	188589009	289696012

289699017	B6167	B616700	Hodgkin's disease, lymphocytic depletion of spleen	Lymphoma	188592008	289699017
826261000006110	B613	B613.00	Hodgkin's disease, lymphocytic-histiocytic predominance	Lymphoma	118607005	177026017
826991000006114	B6130	B613000	Hodgkin's disease, lymphocytic-histiocytic predominance (clinical)	Lymphoma	118607005	177026017
826921000006112	B6133	B613300	Hodgkin's disease, lymphocytic-histiocytic predominance of intra-abdominal lymph nodes	Lymphoma	93493001	154775019
826931000006110	B6136	B613600	Hodgkin's disease, lymphocytic-histiocytic predominance of intrapelvic lymph nodes	Lymphoma	93494007	154776018
826941000006117	B6132	B613200	Hodgkin's disease, lymphocytic-histiocytic predominance of intrathoracic lymph nodes	Lymphoma	93495008	154777010
826951000006115	B6131	B613100	Hodgkin's disease, lymphocytic-histiocytic predominance of lymph nodes of head, face and neck	Lymphoma	188554007	289650016
826911000006116	B6135	B613500	Hodgkin's disease, lymphocytic-histiocytic predominance of lymph nodes of inguinal region and lower limb	Lymphoma	188559002	289655014
2663461018	B615	B615.00	Hodgkin's disease, mixed cellularity	Lymphoma	118609008	2663461018
289679011	B615z	B615z00	Hodgkin's disease, mixed cellularity NOS	Lymphoma	118609008	177028016
826561000006112	B6152	B615200	Hodgkin's disease, mixed cellularity of intrathoracic lymph nodes	Lymphoma	188576003	289672019
826571000006117	B6151	B615100	Hodgkin's disease, mixed cellularity of lymph nodes of head, face and neck	Lymphoma	188575004	289671014
289670010	B6150	B615000	Hodgkin's disease, mixed cellularity of unspecified site	Lymphoma	118609008	177028016
1220414014	B614	B614.00	Hodgkin's disease, nodular sclerosis	Lymphoma	118608000	1220414014
289669014	B614z	B614z00	Hodgkin's disease, nodular sclerosis NOS	Lymphoma	118608000	177027014
826631000006116	B6143	B614300	Hodgkin's disease, nodular sclerosis of intra-abdominal lymph nodes	Lymphoma	188567005	289663010
826651000006111	B6142	B614200	Hodgkin's disease, nodular sclerosis of intrathoracic lymph nodes	Lymphoma	188566001	289662017
826661000006113	B6144	B614400	Hodgkin's disease, nodular sclerosis of lymph nodes of axilla and upper limb	Lymphoma	188568000	289664016
826611000006110	B6141	B614100	Hodgkin's disease, nodular sclerosis of lymph nodes of head, face and neck	Lymphoma	188565002	289661012
826671000006118	B6148	B614800	Hodgkin's disease, nodular sclerosis of lymph nodes of multiple sites	Lymphoma	188572001	289668018
289660013	B6140	B614000	Hodgkin's disease, nodular sclerosis of unspecified site	Lymphoma	118608000	177027014
2663476014	B611	B611.00	Hodgkin's granuloma	Lymphoma	118602004	2663476014
289629013	B6111	B611100	Hodgkin's granuloma of lymph nodes of head, face and neck	Lymphoma	188534006	289629013
289680014	B6160	B616000	Hodgkin's lymphocytic depletion of unspecified site	Lymphoma	118610003	177029012
289621011	B6103	B610300	Hodgkin's paragranuloma of intra-abdominal lymph nodes	Lymphoma	93493001	3635497016
826751000006118	B6101	B610100	Hodgkin's paragranuloma of lymph nodes of head, face, and neck	Lymphoma	93542008	154855017
2663376010	B612	B612.00	Hodgkin's sarcoma	Lymphoma	118606001	2663376010
289643015	B6124	B612400	Hodgkin's sarcoma of lymph nodes of axilla and upper limb	Lymphoma	188547001	289643015
289659015	B613z	B613z00	Hodgkin's, lymphocytic-histiocytic predominance NOS	Lymphoma	118607005	177026017
1815461000006110	B62F0	B62F000	Low grade B-cell lymphoma	Lymphoma	277615007	414243016
2168691000000110	B62F2	B62F200	Lymphoblastic (diffuse) lymphoma	Lymphoma	109965004	2168691000000110

344670010	B62x1	B62x100	Lymphoepithelioid lymphoma	Lymphoma	109976000	344670010
289591011	B601	B601.00	Lymphosarcoma	Lymphoma	188498009	289591011
289580016	B60	B60..00	Lymphosarcoma and reticulosarcoma	Lymphoma	188487008	289580016
289601010	B601z	B601z00	Lymphosarcoma NOS	Lymphoma	188498009	289591011
289595019	B6013	B601300	Lymphosarcoma of intra-abdominal lymph nodes	Lymphoma	188502002	289595019
289594015	B6012	B601200	Lymphosarcoma of intrathoracic lymph nodes	Lymphoma	188501009	289594015
289593014	B6011	B601100	Lymphosarcoma of lymph nodes of head, face and neck	Lymphoma	188500005	289593014
731691000006110	B6015	B601500	Lymphosarcoma of lymph nodes of inguinal region and lower limb	Lymphoma	188504001	289597010
289599013	B6017	B601700	Lymphosarcoma of spleen	Lymphoma	188506004	289599013
289592016	B6010	B601000	Lymphosarcoma of unspecified site	Lymphoma	188498009	289591011
728721000006111	B62y	B62y.00	Malignant lymphoma	Lymphoma	118600007	2663474012
311671000006118	BBg1	BBg1.00	Malignant lymphoma	Lymphoma	21964009	36846011
289820010	B62x	B62x.00	Malignant lymphoma (clinical)	Lymphoma	118600007	177019017
289829011	B62yz	B62yz00	Malignant lymphoma NOS	Lymphoma	118600007	2663474012
289830018	B62y0	B62y000	Malignant lymphoma NOS of unspecified site	Lymphoma	118600007	2663474012
289842011	B62y3	B62y300	Malignant lymphoma of intra-abdominal lymph nodes	Lymphoma	93191005	154196016
289845013	B62y6	B62y600	Malignant lymphoma of intrapelvic lymph nodes	Lymphoma	93192003	154198015
289832014	B62y2	B62y200	Malignant lymphoma of intrathoracic lymph nodes	Lymphoma	93193008	154200014
728781000006110	B62y4	B62y400	Malignant lymphoma of lymph nodes of axilla AND/OR upper limb	Lymphoma	93194002	154202018
289831019	B62y1	B62y100	Malignant lymphoma of lymph nodes of head, face AND/OR neck	Lymphoma	93195001	154205016
728771000006112	B62y5	B62y500	Malignant lymphoma of lymph nodes of inguinal region AND/OR lower limb	Lymphoma	93196000	154208019
289847017	B62y8	B62y800	Malignant lymphoma of lymph nodes of multiple sites	Lymphoma	93197009	154211018
289846014	B62y7	B62y700	Malignant lymphoma of spleen	Lymphoma	93198004	154213015
311461000006111	BBk	BBk..00	Malignant lymphoma, follicular AND/OR nodular	Lymphoma	115245001	176001017
414253015	B6279-1	B627911	Maltoma	Lymphoma	277622004	414253015
123978016	B62F1	B62F100	Mantle cell lymphoma	Lymphoma	443487006	2839720017
7334771000006117	^ESCTMA733477		Marginal zone lymphoma	Lymphoma	447100004	2883837010
304151000006118	BBm9	BBm9.00	Monocytoid B-cell lymphoma	Lymphoma	128803008	207315010
414252013	B6279	B627900	Mucosa-associated lymphoma	Lymphoma	277622004	414252013
2663191017	B621	B621.00	Mycosis fungoides	Lymphoma	118618005	2663191017
289739013	B621z	B621z00	Mycosis fungoides NOS	Lymphoma	118618005	177037016
157075017	B6214	B621400	Mycosis fungoides of lymph nodes of axilla and upper limb	Lymphoma	94710006	157075017
683691000006113	B6215	B621500	Mycosis fungoides of lymph nodes of inguinal region and lower limb	Lymphoma	94712003	157079011
289738017	B6218	B621800	Mycosis fungoides of lymph nodes of multiple sites	Lymphoma	188627002	289738017
289727017	B6210	B621000	Mycosis fungoides of unspecified site	Lymphoma	118618005	177037016
673611000006113	B620	B620.00	Nodular lymphoma	Lymphoma	269476000	403709014
289726014	B620z	B620z00	Nodular lymphoma NOS	Lymphoma	269476000	403709014
157686016	B6203	B620300	Nodular lymphoma of intra-abdominal lymph nodes	Lymphoma	95186006	157686016
289718018	B6201	B620100	Nodular lymphoma of lymph nodes of head, face and neck	Lymphoma	188609000	289718018

673681000006118	B6205	B620500	Nodular lymphoma of lymph nodes of inguinal region and lower limb	Lymphoma	188613007	289722011
157692010	B6208	B620800	Nodular lymphoma of lymph nodes of multiple sites	Lymphoma	95192000	157692010
289717011	B6200	B620000	Nodular lymphoma of unspecified site	Lymphoma	269476000	403709014
853831000006113	EGTON299		Non hodgkin lymphoma	Lymphoma	853831000006109	853831000006113
2839871014	B627-1	B627.11	Non-Hodgkin lymphoma	Lymphoma	118601006	2663475013
291963013	BBmH	BBmH.00	Non-Hodgkin lymphoma (category)	Lymphoma	128929007	207612017
587201000006115	B627	B627.00	Non-Hodgkin's lymphoma	Lymphoma	118601006	2663475013
406154013	ByuDF	ByuDF00	Non-Hodgkin's lymphoma (clinical)	Lymphoma	118601006	177020011
1815091000006114	B61C	B61C.00	Other classical Hodgkin lymphoma	Lymphoma	118599009	2663473018
289613017	B60y	B60y.00	Other specified reticulosarcoma or lymphosarcoma	Lymphoma	188487008	289580016
289813015	B627B	B627B00	Other types of follicular non-Hodgkin's lymphoma	Lymphoma	308121000	451429010
2659756011	B62x2	B62x200	Peripheral T-cell lymphoma	Lymphoma	109977009	2659756011
289614011	B60z	B60z.00	Reticulosarcoma or lymphosarcoma NOS	Lymphoma	188487008	289580016
146651000006118	B622	B622.00	Sezary's disease	Lymphoma	118611004	2821061019
289749011	B622z	B622z00	Sezary's disease NOS	Lymphoma	118611004	177030019
344657018	B62x0	B62x000	T-zone lymphoma	Lymphoma	109975001	344657018
344806015	B62x6	B62x600	True histiocytic lymphoma	Lymphoma	109988003	344806015
1221446018	BBEG0	BBEG000	[M]Acral lentiginous melanoma, malignant	Melanoma	16974005	1221445019
310961000006112	BBEG-1	BBEG.11	[M]Lentigo maligna melanoma	Melanoma	302836005	444775014
396171000006118	Byu40	Byu4000	[X]Malignant melanoma of other+unspecified parts of face	Melanoma	93225001	154247017
292118014	Byu41	Byu4100	[X]Malignant melanoma of skin, unspecified	Melanoma	93655004	155060012
292116013	Byu4	Byu4.00	[X]Melanoma and other malignant neoplasms of skin	Melanoma	372130007	1210707014
305231000006118	BBEA	BBEA.00	Amelanotic melanoma	Melanoma	70594002	117272014
5911811000006115	^ESCTLE591181		Lentigo maligna melanoma	Melanoma	302837001	444778011
403754010	BBE1	BBE1.00	Malignant melanoma	Melanoma	2092003	4601016
6365751000006116	^ESCTMA636575		Malignant melanoma	Melanoma	372244006	1210820017
289052011	B3275	B327500	Malignant melanoma of ankle	Melanoma	188073001	289052011
289012014	B3220	B322000	Malignant melanoma of auricle (ear)	Melanoma	188033007	289012014
289028016	B3250	B325000	Malignant melanoma of axilla	Melanoma	188049009	289028016
454085011	B3257	B325700	Malignant melanoma of back	Melanoma	310498001	454085011
289029012	B3251	B325100	Malignant melanoma of breast	Melanoma	188050009	289029012
289030019	B3252	B325200	Malignant melanoma of buttock	Melanoma	188051008	289030019
457109011	B3258	B325800	Malignant melanoma of chest wall	Melanoma	313248004	457109011
289017015	B3231	B323100	Malignant melanoma of chin	Melanoma	188038003	289017015
1694781000006115	EMISNQMA25		Malignant melanoma of choroid	Melanoma	255021005	379994016
289011019	B322	B322.00	Malignant melanoma of ear and external auricular canal	Melanoma	188032002	289011019
289014010	B322z	B322z00	Malignant melanoma of ear and external auricular canal NOS	Melanoma	188032002	289011019
289013016	B3221	B322100	Malignant melanoma of external auditory meatus	Melanoma	188034001	289013016
1235785014	B3230	B323000	Malignant melanoma of external surface of cheek	Melanoma	93217003	1235785014
1235798017	B3234	B323400	Malignant melanoma of external surface of nose	Melanoma	93643005	1235798017
409844012	B509	B509.00	Malignant melanoma of eye	Melanoma	274087000	409844012
1235786010	B3232	B323200	Malignant melanoma of eyebrow	Melanoma	93223008	1235786010
728201000006110	B321	B321.00	Malignant melanoma of eyelid	Melanoma	231834005	347406017
289043019	B3264	B326400	Malignant melanoma of finger	Melanoma	188064009	289043019
289054012	B3277	B327700	Malignant melanoma of foot	Melanoma	188075008	289054012

289041017	B3262	B326200	Malignant melanoma of fore-arm	Melanoma	188062008	289041017
289019017	B3233	B323300	Malignant melanoma of forehead	Melanoma	188040008	289019017
289056014	B3279	B327900	Malignant melanoma of great toe	Melanoma	188077000	289056014
289031015	B3253	B325300	Malignant melanoma of groin	Melanoma	188052001	289031015
289042012	B3263	B326300	Malignant melanoma of hand	Melanoma	188063003	289042012
289053018	B3276	B327600	Malignant melanoma of heel	Melanoma	188074007	289053018
289047018	B3270	B327000	Malignant melanoma of hip	Melanoma	188068007	289047018
289049015	B3272	B327200	Malignant melanoma of knee	Melanoma	188070003	289049015
289009011	B320	B320.00	Malignant melanoma of lip	Melanoma	188030005	289009011
289051016	B3274	B327400	Malignant melanoma of lower leg	Melanoma	188072006	289051016
289046010	B327	B327.00	Malignant melanoma of lower limb and hip	Melanoma	188067002	289046010
289057017	B327z	B327z00	Malignant melanoma of lower limb or hip NOS	Melanoma	188067002	289046010
289025018	B3241	B324100	Malignant melanoma of neck	Melanoma	188046002	289025018
289060012	B32y	B32y.00	Malignant melanoma of other specified skin site	Melanoma	93655004	155060012
289033017	B3255	B325500	Malignant melanoma of perineum	Melanoma	188054000	289033017
289050015	B3273	B327300	Malignant melanoma of popliteal fossa area	Melanoma	188071004	289050015
289024019	B3240	B324000	Malignant melanoma of scalp	Melanoma	188045003	289024019
289023013	B324	B324.00	Malignant melanoma of scalp and neck	Melanoma	188044004	289023013
289026017	B324z	B324z00	Malignant melanoma of scalp and/or neck	Melanoma	188044004	1775484014
289039018	B3260	B326000	Malignant melanoma of shoulder	Melanoma	188060000	289039018
155060012	B32	B32..00	Malignant melanoma of skin	Melanoma	93655004	155060012
289063014	B32z	B32z.00	Malignant melanoma of skin NOS	Melanoma	93655004	155060012
289022015	B323z	B323z00	Malignant melanoma of skin of face	Melanoma	93225001	154247017
289015011	B323	B323.00	Malignant melanoma of skin of face	Melanoma	93225001	154247017
289027014	B325	B325.00	Malignant melanoma of skin of trunk	Melanoma	93651008	155054012
289021010	B3235	B323500	Malignant melanoma of temple	Melanoma	188042000	289021010
289048011	B3271	B327100	Malignant melanoma of thigh	Melanoma	188069004	289048011
289044013	B3265	B326500	Malignant melanoma of thumb	Melanoma	188065005	289044013
289055013	B3278	B327800	Malignant melanoma of toe	Melanoma	188076009	289055013
289037016	B325z	B325z00	Malignant melanoma of trunk	Melanoma	269579005	403893010
289034011	B3256	B325600	Malignant melanoma of umbilicus	Melanoma	188055004	289034011
289040016	B3261	B326100	Malignant melanoma of upper arm	Melanoma	188061001	289040016
727731000006113	B326	B326.00	Malignant melanoma of upper limb	Melanoma	269580008	403894016
289045014	B326z	B326z00	Malignant melanoma of upper limb or shoulder NOS	Melanoma	269580008	403894016
880161000006114	B324-99	B324.99	Melanoma - head/neck	Melanoma	188044004	880161000006114
880191000006118	B327-99	B327.99	Melanoma - lower limb	Melanoma	188067002	880191000006118
880171000006119	B325-99	B325.99	Melanoma - trunk	Melanoma	109284008	880171000006119
880151000006112	B32-99	B32..99	Melanoma of skin	Melanoma	93655004	880151000006112
7278381000006116	^ESCTME727838		Metastatic malignant melanoma	Melanoma	443493003	2838285019
314051000006119	BBE2	BBE2.00	Nodular melanoma	Melanoma	2142002	4703012
173816012	B32y0	B32y000	Overlapping malignant melanoma of skin	Melanoma	109267002	173816012
5349281000006110	^ESCTSU534928		Superficial spreading malignant melanoma of skin	Melanoma	254730000	379402015
317201000006115	BBEH	BBEH.00	Superficial spreading melanoma	Melanoma	55320002	91969014
403771011	B00-1	B00..11	Carcinoma of lip	Non-oropharyngeal	269515006	403771011
729571000006113	B20y	B20y.00	Malig neop other site nasal cavity, middle ear and sinuses	Non-oropharyngeal	187828007	288770010
724411000006118	B0033	B003300	Malig neoplasm of lower lip, oral aspect	Non-oropharyngeal	271568003	406398015
288430019	B014	B014.00	Malignant neoplasm of anterior 2/3 of tongue unspecified	Non-oropharyngeal	363360003	482519011
288423013	B0130	B013000	Malignant neoplasm of anterior 2/3 of tongue ventral surface	Non-oropharyngeal	187640005	288423013

288443019	B040	B040.00	Malignant neoplasm of anterior portion of floor of mouth	Non-oropharyngeal	187652003	288443019
288552014	B073	B073.00	Malignant neoplasm of anterior wall of nasopharynx	Non-oropharyngeal	187700006	288552014
5590751000006110	^ESCTAD559075		Adenocarcinoma of oesophagus	Oesophagus	276803003	413100012
6364671000006117	^ESCTCA636467		Carcinoma of oesophagus	Oesophagus	372138000	1207350011
288597017	B102	B102.00	Malignant neoplasm of abdominal oesophagus	Oesophagus	187724003	288597017
288589012	B100	B100.00	Malignant neoplasm of cervical oesophagus	Oesophagus	187722004	288589012
288608011	B105	B105.00	Malignant neoplasm of lower third of oesophagus	Oesophagus	187727005	288608011
288607018	B104	B104.00	Malignant neoplasm of middle third of oesophagus	Oesophagus	187726001	288607018
1216488012	B10	B10..00	Malignant neoplasm of oesophagus	Oesophagus	363402007	1216488012
403674011	B10z	B10z.00	Malignant neoplasm of oesophagus NOS	Oesophagus	363402007	1216488012
288614016	B10y	B10y.00	Malignant neoplasm of other specified part of oesophagus	Oesophagus	363402007	482603012
288594012	B101	B101.00	Malignant neoplasm of thoracic oesophagus	Oesophagus	187723009	288594012
288602012	B103	B103.00	Malignant neoplasm of upper third of oesophagus	Oesophagus	187725002	288602012
1215862010	B106	B106.00	Malignant neoplasm, overlapping lesion of oesophagus	Oesophagus	109835005	1215862010
4746821000006110	^ESCTMA474682		Malignant tumour of lower third of oesophagus	Oesophagus	187727005	288609015
6244811000006110	^ESCTMA624481		Malignant tumour of oesophagus	Oesophagus	363402007	482603012
1216486011	B10z-1	B10z.11	Oesophageal cancer	Oesophagus	363402007	1216486011
5590771000006117	^ESCTSQ559077		Squamous cell carcinoma of oesophagus	Oesophagus	276804009	413101011
288533015	B0710	B071000	Malignant neoplasm of adenoid	Oropharyngeal	187694000	288533015
288506019	B064	B064.00	Malignant neoplasm of anterior epiglottis	Oropharyngeal	187681002	288506019
288509014	B064z	B064z00	Malignant neoplasm of anterior epiglottis NOS	Oropharyngeal	187681002	288506019
288411019	B0100	B010000	Malignant neoplasm of base of tongue dorsal surface	Oropharyngeal	187631006	288411019
451428019	B215	B215.00	Malignant neoplasm of epiglottis NOS	Oropharyngeal	187842004	288796012
288507011	B0640	B064000	Malignant neoplasm of epiglottis, free border	Oropharyngeal	187682009	288507011
288485015	B0620	B062000	Malignant neoplasm of faucial pillar	Oropharyngeal	187675005	288485015
1228530015	B0600	B060000	Malignant neoplasm of faucial tonsil	Oropharyngeal	363393007	1228530015
288508018	B0641	B064100	Malignant neoplasm of glossoepiglottic fold	Oropharyngeal	187683004	288508018
155332018	B066	B066.00	Malignant neoplasm of lateral wall of oropharynx	Oropharyngeal	448868009	2901613017
724881000006110	B016	B016.00	Malignant neoplasm of lingual tonsil	Oropharyngeal	363377003	482550013
288518011	B06z	B06z.00	Malignant neoplasm of oropharynx NOS	Oropharyngeal	363392002	482583018
288516010	B06y	B06y.00	Malignant neoplasm of oropharynx, other specified sites	Oropharyngeal	363392002	482583018
288517018	B06yz	B06yz00	Malignant neoplasm of other specified site of oropharynx NOS	Oropharyngeal	363392002	482583018
1228529013	B0601	B060100	Malignant neoplasm of palatine tonsil	Oropharyngeal	363393007	1228529013
2475984012	B0622	B062200	Malignant neoplasm of palatoglossal arch	Oropharyngeal	254459004	2475984012
723391000006115	B0623	B062300	Malignant neoplasm of palatopharyngeal arch	Oropharyngeal	187675005	288479010
288546013	B0720	B072000	Malignant neoplasm of pharyngeal recess	Oropharyngeal	187697007	288546013
288568015	B083	B083.00	Malignant neoplasm of posterior pharynx	Oropharyngeal	187709007	288568015
1228509012	B010-1	B010.11	Malignant neoplasm of posterior third of tongue	Oropharyngeal	363376007	1228509012
288515014	B067	B067.00	Malignant neoplasm of posterior wall of oropharynx	Oropharyngeal	187688008	288515014
721621000006110	B053	B053.00	Malignant neoplasm of soft palate	Oropharyngeal	363388009	482575012
721361000006112	B061	B061.00	Malignant neoplasm of tonsillar fossa	Oropharyngeal	363394001	482587017
288486019	B062	B062.00	Malignant neoplasm of tonsillar pillar	Oropharyngeal	187675005	288486019
721041000006111	B054	B054.00	Malignant neoplasm of uvula	Oropharyngeal	363389001	482577016

288478019	B060z	B060z00	Malignant neoplasm tonsil NOS	Oropharyngeal	363393007	482585013
727381000006110	B010	B010.00	Malignant tumour of base of tongue	Oropharyngeal	363376007	482549013
723571000006111	B06	B06..00	Malignant tumour of oropharynx	Oropharyngeal	363392002	482583018
721351000006110	B060	B060.00	Malignant tumour of tonsil	Oropharyngeal	363393007	482586014
288505015	B062z	B062z00	Malignant tumour of tonsillar fossa	Oropharyngeal	363394001	482587017
723321000006117	B0602	B060200	Overlapping malignant neoplasm of tonsil	Oropharyngeal	110013004	174720013
7007951000006111	^ESCTSQ700795		Squamous cell carcinoma of oropharynx	Oropharyngeal	423464009	2643843013
1231544010	BB5R9	BB5R900	[M] Neuroendocrine carcinoma	Other/unknown	55937004	1231545011
304681000006116	BB5-1	BB5..11	[M]Adenocarcinomas	Other/unknown	35917007	59935017
291414018	BB5R1	BB5R100	[M]Carcinoid tumour, malignant	Other/unknown	189607006	291404010
1233222010	BB14	BB14.00	[M]Carcinomatosis	Other/unknown	7010000	1233222010
1222216017	BB5f1-1	BB5f111	[M]Follicular carcinoma	Other/unknown	5257006	9812015
1229372013	BB55	BB55.00	[M]Linitis plastica	Other/unknown	37995004	1229372013
403758013	BBc1	BBc1.00	[M]Neuroblastoma NOS	Other/unknown	87364003	144849016
314701000006115	BB9L	BB9L.00	[M]Paget's disease, extramammary, excluding Paget's disease of bone	Other/unknown	71447003	1233389019
1216595017	BBba	BBba.00	[M]Primitive neuroectodermal tumour	Other/unknown	39781001	1216596016
1219790013	BB83	BB83.00	[M]Pseudomyxoma peritonei	Other/unknown	112679004	1219790013
291854012	BBc9	BBc9.00	[M]Retinoblastomas	Other/unknown	19906005	33508018
316291000006113	BB13-1	BB13.11	[M]Secondary carcinoma	Other/unknown	79282002	131544016
316301000006114	BB03-1	BB03.11	[M]Secondary neoplasm	Other/unknown	14799000	25140016
1222303015	BBQ72	BBQ7200	[M]Teratoma, malignant, NOS	Other/unknown	19467007	1222303015
396091000006118	Byu58	Byu5800	[X]Mal neoplasm/connective+soft tissue of trunk,unspecified	Other/unknown	269469005	403699012
396191000006117	Byu23	Byu2300	[X]Malignant neopl/overlapping les/resp+intrathoracic organs	Other/unknown	93841009	510723012
396281000006111	ByuC	ByuC.00	[X]Malignant neoplasm of ill-defined, secondary and unspecified sites	Other/unknown	363346000	482491013
292102015	Byu12	Byu1200	[X]Malignant neoplasm of intestinal tract, part unspecified	Other/unknown	363508008	482802017
292146011	Byu82	Byu8200	[X]Malignant neoplasm of male genital organ, unspecified	Other/unknown	363515000	482817018
292158011	ByuC0	ByuC000	[X]Malignant neoplasm of other specified sites	Other/unknown	363346000	1208875016
292132011	Byu57	Byu5700	[X]Malignant neoplasm of peritoneum, unspecified	Other/unknown	363492001	482771017
292166019	ByuC8	ByuC800	[X]Malignant neoplasm without specification of site	Other/unknown	302817000	444727016
396471000006112	Byu33	Byu3300	[X]Malignant neoplasm/bone+articular cartilage, unspecified	Other/unknown	443679004	2839107012
396501000006117	Byu59	Byu5900	[X]Malignant neoplasm/connective + soft tissue,unspecified	Other/unknown	269469005	403699012
396511000006119	Byu24	Byu2400	[X]Malignant neoplasm/ill-defined sites within resp system	Other/unknown	449096009	2899758015
396541000006115	Byu80	Byu8000	[X]Malignant neoplasm/other specified male genital organs	Other/unknown	363515000	482817018
396561000006116	Byu21	Byu2100	[X]Malignant neoplasm/overlap lesion/heart,mediastinm+pleura	Other/unknown	109384006	1217361012
396631000006112	ByuE	ByuE.00	[X]Malignant neoplasms/independent (primary) multiple sites	Other/unknown	188478004	289566011
396641000006119	ByuE0	ByuE000	[X]Malignant neoplasms/independent(primary)multiple sites	Other/unknown	188478004	289566011
398291000006110	Byu50-1	Byu5011	[X]Mesothelioma of lung	Other/unknown	109378008	2660053016
292165015	ByuC7	ByuC700	[X]Secondary malignant neoplasm of other specified sites	Other/unknown	128462008	206784011
291372011	BB52	BB52.00	Adenocarcinoma	Other/unknown	35917007	59935017
304661000006114	BB57	BB57.00	Adenocarcinoma - intestinal type	Other/unknown	25190001	1224772013

291374012	BB53	BB53.00	Adenocarcinoma, metastatic	Other/unknown	4590003	7897016
304701000006118	BB5J	BB5J.00	Adenoid cystic carcinoma	Other/unknown	11671000	20161014
304921000006113	BBB0	BBB0.00	Adenosquamous carcinoma	Other/unknown	59367005	98607018
4031071000006114	^ESCTBO403107		Bony metastasis	Other/unknown	94222008	1235816014
880041000006111	B22-99	B22..99	Ca trachea/bronchus/lung	Other/unknown	430621000	880041000006111
880031000006118	B22-98	B22..98	Ca trachea/bronchus/lung NOS	Other/unknown	430621000	880031000006118
533321000006119	B-1	B....11	Cancer	Other/unknown	363346000	1228478017
1488798019	1O0	1O0..00	Cancer confirmed	Other/unknown	395099008	1488798019
292126018	Byu51	Byu5100	Cancer, mesothelioma	Other/unknown	109378008	3036244012
380094018	B3-1	B3...11	Carcinoma of bone, connective tissue, skin and breast	Other/unknown	255068000	380094018
537051000006118	B1-1	B1...11	Carcinoma of digestive organs and peritoneum	Other/unknown	269456006	403673017
380092019	B4-1	B4...11	Carcinoma of genitourinary organ	Other/unknown	255066001	380092019
380211016	B5-1	B5...11	Carcinoma of other and unspecified sites	Other/unknown	363346000	1228478017
291330010	BB19	BB19.00	Carcinoma, anaplastic	Other/unknown	58248003	96799014
291327015	BB13	BB13.00	Carcinoma, metastatic	Other/unknown	79282002	131542017
450901012	B590-1	B590.11	Carcinomatosis	Other/unknown	307593001	450901012
1576201000006111	EGTON2B		Cause of Death- Malignant Neoplasms	Other/unknown	1576201000006107	1576201000006111
543041000006111	B5832	B583200	Cerebral metastases	Other/unknown	94248000	1235820013
543111000006111	B51-1	B51..11	Cerebral tumour - malignant	Other/unknown	428061005	2692069010
291443014	BB5X1	BB5X100	Clear cell adenocarcinoma	Other/unknown	30546008	51122012
880761000006113	B5z-98	B5z..98	Disseminated Ca -unspecif site	Other/unknown	541861000000107	880761000006113
403704016	B590	B590.00	Disseminated malignancy	Other/unknown	405843009	2163172012
307631000006111	BB91-1	BB91.11	Duct carcinoma	Other/unknown	82711006	137210015
3845561000006111	^ESCTDU384556		Ductal carcinoma	Other/unknown	82711006	137208017
291718019	BBQ3	BBQ3.00	Embryonal carcinoma	Other/unknown	28047004	46954010
291452017	BB5f1	BB5f100	Follicular adenocarcinoma	Other/unknown	5257006	9812015
309041000006115	BBQ2	BBQ2.00	Germinoma	Other/unknown	28307001	47386017
310321000006119	BB9G	BB9G.00	Infiltrating ductular carcinoma	Other/unknown	58477004	97180015
291540014	BB9F	BB9F.00	Lobular carcinoma	Other/unknown	89740008	148778015
732441000006113	B56-1	B56..11	Lymph node metastases	Other/unknown	94392001	156323012
730031000006113	B310z	B310z00	Malig neop connective and soft tissue head, face, neck NOS	Other/unknown	302816009	444723017
730241000006113	B312z	B312z00	Malig neop connective and soft tissue hip and leg NOS	Other/unknown	187999008	288974012
729281000006113	B31y	B31y.00	Malig neop connective and soft tissue other specified site	Other/unknown	269469005	403699012
729361000006113	B310	B310.00	Malig neop of connective and soft tissue head, face and neck	Other/unknown	302816009	444723017
729371000006118	B314z	B314z00	Malig neop of connective and soft tissue of abdomen NOS	Other/unknown	188015001	288991011
729431000006111	B315z	B315z00	Malig neop of connective and soft tissue of pelvis NOS	Other/unknown	363366009	482531013
729441000006118	B313z	B313z00	Malig neop of connective and soft tissue of thorax NOS	Other/unknown	363365008	482529016
729501000006119	B52	B52..00	Malig neop of other and unspecified parts of nervous system	Other/unknown	372063002	1210641014
729521000006112	B24y	B24y.00	Malig neop of other site of heart, thymus and mediastinum	Other/unknown	187881004	288841011
729541000006117	B304z	B304z00	Malig neop of scapula and long bones of upper arm NOS	Other/unknown	187929000	288898016
729551000006115	B2z0	B2z0.00	Malig neop of upper respiratory tract, part unspecified	Other/unknown	187833006	288777013
729561000006118	B1z	B1z..00	Malig neop oth/ill-defined sites digestive tract/peritoneum	Other/unknown	255077007	380111011

729601000006118	B2z	B2z..00	Malig neop other/ill-defined sites resp/intrathoracic organs	Other/unknown	428100006	2691955010
728461000006112	B524	B524.00	Malig neopl peripheral nerves and autonomic nervous system	Other/unknown	188321006	289384012
728471000006117	B52W	B52W.00	Malig neopl, overlap lesion brain & other part of CNS	Other/unknown	109911004	174604014
353772013	B5762	B576200	Malignant ascites	Other/unknown	236005001	353772013
308261000006116	BBP5	BBP5.00	Malignant epithelioid mesothelioma	Other/unknown	65278006	1232663018
705211000006111	B232	B232.00	Malignant mesothelioma of pleura	Other/unknown	254645002	379214013
313701000006118	BB02	BB02.00	Malignant neoplasm	Other/unknown	363346000	1228480011
291326012	BB12	BB12.00	Malignant Neoplasm (Morphology)	Other/unknown	367651003	504898018
289987014	B6z	B6z..00	Malignant neoplasm lymphatic or haematopoietic tissue NOS	Other/unknown	269475001	403705015
727811000006111	B6y	B6y..00	Malignant neoplasm lymphatic or haematopoietic tissue OS	Other/unknown	269475001	403705015
289441010	B552	B552.00	Malignant neoplasm of abdomen	Other/unknown	188366002	289441010
155078019	B242	B242.00	Malignant neoplasm of anterior mediastinum	Other/unknown	449224009	2901920014
727761000006116	B082	B082.00	Malignant neoplasm of aryepiglottic fold, hypopharyngeal aspect	Other/unknown	187708004	288566016
288799017	B2130	B213000	Malignant neoplasm of arytenoid cartilage	Other/unknown	187843009	288799017
289246015	B482	B482.00	Malignant neoplasm of body of penis	Other/unknown	188230001	289246015
403691010	B30	B30..00	Malignant neoplasm of bone and articular cartilage	Other/unknown	443679004	2839107012
288959010	B30z	B30z.00	Malignant neoplasm of bone and articular cartilage	Other/unknown	1090821000000101	2732521000000110
729331000006116	B3	B3...00	Malignant neoplasm of bone, connective tissue, skin and breast	Other/unknown	271467005	406262013
288869015	B300	B300.00	Malignant neoplasm of bones of skull and face	Other/unknown	187900002	288869015
288883018	B300z	B300z00	Malignant neoplasm of bones of skull and face NOS	Other/unknown	187900002	288869015
288941012	B3082	B308200	Malignant neoplasm of calcaneum	Other/unknown	187967002	288941012
454201010	B3310	B331000	Malignant neoplasm of canthus	Other/unknown	310599006	454201010
155168019	B544	B544.00	Malignant neoplasm of carotid body	Other/unknown	447883002	2900052019
288910011	B3051	B305100	Malignant neoplasm of carpal bone - lunate	Other/unknown	187938003	288910011
288909018	B3050	B305000	Malignant neoplasm of carpal bone - scaphoid	Other/unknown	187937008	288909018
288962013	B3103	B310300	Malignant neoplasm of cartilage of ear	Other/unknown	187987001	288962013
288771014	B2000	B200000	Malignant neoplasm of cartilage of nose	Other/unknown	187829004	288771014
482741013	B525	B525.00	Malignant neoplasm of cauda equina	Other/unknown	363477002	482741013
289179012	B41y0	B41y000	Malignant neoplasm of cervical stump	Other/unknown	188183000	289179012
288885013	B3020	B302000	Malignant neoplasm of cervical vertebra	Other/unknown	187916000	288885013
289436019	B5511	B551100	Malignant neoplasm of chest wall	Other/unknown	712750007	3285316016
727181000006113	B506	B506.00	Malignant neoplasm of choroid	Other/unknown	363466008	482728014
289352017	B5150	B515000	Malignant neoplasm of choroid plexus	Other/unknown	188292007	289352017
289308013	B5000	B500000	Malignant neoplasm of ciliary body	Other/unknown	188263008	289308013
155191015	B3032	B303200	Malignant neoplasm of clavicle	Other/unknown	93757004	155191015
174518012	B1420	B142000	Malignant neoplasm of cloacogenic zone	Other/unknown	363491008	482769017
155193017	B5452	B545200	Malignant neoplasm of coccygeal body	Other/unknown	93759001	155193017
288929017	B3064	B306400	Malignant neoplasm of coccygeal vertebra	Other/unknown	187957007	288929017
726371000006114	B503	B503.00	Malignant neoplasm of conjunctiva	Other/unknown	363463000	482722010
288960017	B31	B31..00	Malignant neoplasm of connective and other soft tissue	Other/unknown	269469005	403699012
288991011	B314	B314.00	Malignant neoplasm of connective and soft tissue of abdomen	Other/unknown	188015001	288991011
729381000006115	B3140	B314000	Malignant neoplasm of connective and soft tissue of abdominal wall	Other/unknown	188016000	288992016

288986010	B3130	B313000	Malignant neoplasm of connective and soft tissue of axilla	Other/unknown	188010006	288986010
288999013	B3150	B315000	Malignant neoplasm of connective and soft tissue of buttock	Other/unknown	188020001	288999013
288971016	B3114	B311400	Malignant neoplasm of connective and soft tissue of finger	Other/unknown	187996001	288971016
288979019	B3124	B312400	Malignant neoplasm of connective and soft tissue of foot	Other/unknown	188004006	288979019
288969016	B3112	B311200	Malignant neoplasm of connective and soft tissue of fore-arm	Other/unknown	187994003	288969016
288970015	B3113	B311300	Malignant neoplasm of connective and soft tissue of hand	Other/unknown	187995002	288970015
288975013	B3120	B312000	Malignant neoplasm of connective and soft tissue of hip	Other/unknown	188000002	288975013
729401000006115	B312	B312.00	Malignant neoplasm of connective and soft tissue of hip and lower limb	Other/unknown	187999008	288974012
729411000006117	B3151	B315100	Malignant neoplasm of connective and soft tissue of inguinal region	Other/unknown	188021002	289000010
729421000006113	B3123	B312300	Malignant neoplasm of connective and soft tissue of lower leg	Other/unknown	188003000	288978010
288995019	B315	B315.00	Malignant neoplasm of connective and soft tissue of pelvis	Other/unknown	188019007	288995019
289001014	B3152	B315200	Malignant neoplasm of connective and soft tissue of perineum	Other/unknown	188022009	289001014
730251000006110	B3122	B312200	Malignant neoplasm of connective and soft tissue of popliteal space	Other/unknown	188002005	288977017
288967019	B3110	B311000	Malignant neoplasm of connective and soft tissue of shoulder	Other/unknown	187992004	288967019
729451000006116	B3121	B312100	Malignant neoplasm of connective and soft tissue of thigh and upper leg	Other/unknown	188001003	288976014
288985014	B313	B313.00	Malignant neoplasm of connective and soft tissue of thorax	Other/unknown	188009001	288985014
288972011	B3115	B311500	Malignant neoplasm of connective and soft tissue of thumb	Other/unknown	187997005	288972011
288980016	B3125	B312500	Malignant neoplasm of connective and soft tissue of toe	Other/unknown	188005007	288980016
729291000006111	B311	B311.00	Malignant neoplasm of connective and soft tissue of upper limb and shoulder	Other/unknown	187991006	288966011
289007013	B31z	B31z.00	Malignant neoplasm of connective and soft tissue, site NOS	Other/unknown	269469005	403699012
288968012	B3111	B311100	Malignant neoplasm of connective and soft tissue, upper arm	Other/unknown	187993009	288968012
728481000006119	B3141	B314100	Malignant neoplasm of connective and soft tissues of lumbar spine	Other/unknown	188017009	288993014
728491000006116	B3133	B313300	Malignant neoplasm of connective and soft tissues of thoracic spine	Other/unknown	188013008	288989015
289317013	B5010	B501000	Malignant neoplasm of connective tissue of orbit	Other/unknown	188268004	289317013
726561000006114	B504	B504.00	Malignant neoplasm of cornea	Other/unknown	363464006	482724011
289361017	B51y0	B51y000	Malignant neoplasm of corpus callosum	Other/unknown	188301005	289361017
288894019	B3033	B303300	Malignant neoplasm of costal cartilage	Other/unknown	187925006	288894019
726631000006118	B3034	B303400	Malignant neoplasm of costo-vertebral joint	Other/unknown	187926007	288895018
726641000006111	B520	B520.00	Malignant neoplasm of cranial nerves	Other/unknown	188307009	289367018
289374011	B520z	B520z00	Malignant neoplasm of cranial nerves NOS	Other/unknown	188307009	289367018
288800018	B2131	B213100	Malignant neoplasm of cricoid cartilage	Other/unknown	187844003	288800018
155209019	B3131	B313100	Malignant neoplasm of diaphragm	Other/unknown	93772000	155209019

403673017	B1	B1...00	Malignant neoplasm of digestive organs and peritoneum	Other/unknown	269456006	403673017
288766019	B1zz	B1zz.00	Malignant neoplasm of digestive tract and peritoneum NOS	Other/unknown	255077007	380111011
726071000006111	B2410	B241000	Malignant neoplasm of endocardium	Other/unknown	363436001	482670014
726141000006116	B484	B484.00	Malignant neoplasm of epididymis	Other/unknown	363452003	482700015
155228014	B3000	B300000	Malignant neoplasm of ethmoid bone	Other/unknown	93786001	155228014
288712010	B161z	B161z00	Malignant neoplasm of extrahepatic bile ducts NOS	Other/unknown	363416002	482631015
1208876015	B50	B50..00	Malignant neoplasm of eye	Other/unknown	363461003	1208876015
289330014	B50z	B50z.00	Malignant neoplasm of eye NOS	Other/unknown	363461003	1208876015
729311000006110	B500	B500.00	Malignant neoplasm of eyeball excluding conjunctiva, cornea, retina and choroid	Other/unknown	188261005	289305011
289316016	B500z	B500z00	Malignant neoplasm of eyeball NOS	Other/unknown	188261005	289305011
155246015	B3070	B307000	Malignant neoplasm of femur	Other/unknown	93798006	155246015
155247012	B3071	B307100	Malignant neoplasm of fibula	Other/unknown	93799003	155247012
288921019	B305C	B305C00	Malignant neoplasm of fifth metacarpal bone	Other/unknown	187949009	288921019
288947011	B3088	B308800	Malignant neoplasm of first metatarsal bone	Other/unknown	187973001	288947011
288950014	B308B	B308B00	Malignant neoplasm of fourth metatarsal bone	Other/unknown	187976009	288950014
155259011	B3001	B300100	Malignant neoplasm of frontal bone	Other/unknown	93806005	155259011
406263015	B4	B4...00	Malignant neoplasm of genitourinary organ	Other/unknown	271468000	406263015
725771000006116	B481	B481.00	Malignant neoplasm of glans penis	Other/unknown	363451005	482698012
289341011	B5103	B510300	Malignant neoplasm of globus pallidus	Other/unknown	188285002	289341011
155269017	B5450	B545000	Malignant neoplasm of glomus jugulare	Other/unknown	93814004	155269017
155272012	B3132	B313200	Malignant neoplasm of great vessels	Other/unknown	93817006	155272012
289209019	B4510	B451000	Malignant neoplasm of greater vestibular (Bartholin's) gland	Other/unknown	188211001	289209019
403697014	B305	B305.00	Malignant neoplasm of hand bones	Other/unknown	269467007	403697014
288923016	B305z	B305z00	Malignant neoplasm of hand bones NOS	Other/unknown	269467007	403697014
289424013	B550	B550.00	Malignant neoplasm of head, neck and face	Other/unknown	188353002	289424013
289431012	B550z	B550z00	Malignant neoplasm of head, neck and face NOS	Other/unknown	188353002	289424013
725151000006110	B241	B241.00	Malignant neoplasm of heart	Other/unknown	363435002	482668017
288852017	B24z	B24z.00	Malignant neoplasm of heart, thymus and mediastinum NOS	Other/unknown	187881004	288841011
725221000006111	B6-1	B6...11	Malignant neoplasm of histiocytic tissue	Other/unknown	269475001	403706019
288901015	B3042	B304200	Malignant neoplasm of humerus	Other/unknown	187932002	288901015
289437011	B5512	B551200	Malignant neoplasm of intrathoracic site NOS	Other/unknown	428100006	2691955010
289311014	B5001	B500100	Malignant neoplasm of iris	Other/unknown	188264002	289311014
155308013	B3061	B306100	Malignant neoplasm of ischium	Other/unknown	93842002	155308013
725451000006118	B121	B121.00	Malignant neoplasm of jejunum	Other/unknown	363404008	482607013
288510016	B065	B065.00	Malignant neoplasm of junctional region of epiglottis	Other/unknown	187685006	288510016
724721000006116	B507	B507.00	Malignant neoplasm of lacrimal duct	Other/unknown	188272000	289322013
289322013	B502	B502.00	Malignant neoplasm of lacrimal gland	Other/unknown	188272000	289322013
289325010	B5070	B507000	Malignant neoplasm of lacrimal sac	Other/unknown	188273005	289325010
724761000006110	B213	B213.00	Malignant neoplasm of laryngeal cartilage	Other/unknown	363431006	482660012
288935017	B307z	B307z00	Malignant neoplasm of long bone of lower limb	Other/unknown	449627008	2912930013
288931014	B307	B307.00	Malignant neoplasm of long bones of leg	Other/unknown	449627008	2912930013
288887017	B3022	B302200	Malignant neoplasm of lumbar vertebra	Other/unknown	187918004	288887017
403705015	B6	B6...00	Malignant neoplasm of lymphatic and haemopoietic tissue	Other/unknown	269475001	403705015
288872010	B3002	B300200	Malignant neoplasm of malar bone	Other/unknown	187903000	288872010
155368011	B301	B301.00	Malignant neoplasm of mandible	Other/unknown	448668007	2901472014
155370019	B300A	B300A00	Malignant neoplasm of maxilla	Other/unknown	93888008	155370019
288942017	B3083	B308300	Malignant neoplasm of medial cuneiform	Other/unknown	187968007	288942017

724671000006113	B24X	B24X.00	Malignant neoplasm of mediastinum, part unspecified	Other/unknown	363494000	482776010
482537012	B18y7	B18y700	Malignant neoplasm of mesentery	Other/unknown	363370001	482537012
380146015	B305-2	B305.12	Malignant neoplasm of metacarpal bones	Other/unknown	255091001	380146015
288810010	B2201	B220100	Malignant neoplasm of mucosa of trachea	Other/unknown	187854004	288810010
724051000006110	B2412	B241200	Malignant neoplasm of myocardium	Other/unknown	363437005	482672018
155402013	B3003	B300300	Malignant neoplasm of nasal bone	Other/unknown	93916003	155402013
289326011	B5071	B507100	Malignant neoplasm of nasolacrimal duct	Other/unknown	188274004	289326011
289397014	B52z	B52z.00	Malignant neoplasm of nervous system	Other/unknown	372063002	1210641014
155421017	B3004	B300400	Malignant neoplasm of occipital bone	Other/unknown	93927001	155421017
482641017	B18y3	B18y300	Malignant neoplasm of omentum	Other/unknown	363421004	482641017
723541000006115	B501	B501.00	Malignant neoplasm of orbit	Other/unknown	363462005	482720019
289319011	B501z	B501z00	Malignant neoplasm of orbit NOS	Other/unknown	363462005	482720019
288875012	B3005	B300500	Malignant neoplasm of orbital bone	Other/unknown	187906008	288875012
723601000006116	B55z	B55z.00	Malignant neoplasm of other and ill defined site NOS	Other/unknown	302817000	444727016
289423019	B55	B55..00	Malignant neoplasm of other and ill-defined sites	Other/unknown	302817000	444727016
289574012	B5z	B5z..00	Malignant neoplasm of other and unspecified site NOS	Other/unknown	363346000	1208875016
723641000006119	B5y	B5y..00	Malignant neoplasm of other and unspecified site OS	Other/unknown	363346000	482491013
289251014	B48y	B48y.00	Malignant neoplasm of other male genital organ	Other/unknown	363515000	482817018
289259011	B48yz	B48yz00	Malignant neoplasm of other male genital organ NOS	Other/unknown	363515000	482817018
288857011	B2zy	B2zy.00	Malignant neoplasm of other site of respiratory tract	Other/unknown	93986008	155490010
289396017	B52y	B52y.00	Malignant neoplasm of other specified part of nervous system	Other/unknown	372063002	1210641014
288839010	B23y	B23y.00	Malignant neoplasm of other specified pleura	Other/unknown	363433009	482664015
289329016	B50y	B50y.00	Malignant neoplasm of other specified site of eye	Other/unknown	363461003	1208876015
289448016	B55y	B55y.00	Malignant neoplasm of other specified sites	Other/unknown	363346000	482491013
289298018	B4Ay0	B4Ay000	Malignant neoplasm of overlapping lesion of urinary organs	Other/unknown	188256008	289298018
723451000006119	B4A4	B4A4.00	Malignant neoplasm of paraurethral glands	Other/unknown	363460002	1228591019
155444019	B3006	B300600	Malignant neoplasm of parietal bone	Other/unknown	93945001	155444019
155446017	B18y4	B18y400	Malignant neoplasm of parietal peritoneum	Other/unknown	93947009	155446017
155447014	B230	B230.00	Malignant neoplasm of parietal pleura	Other/unknown	449067008	2901158012
288924010	B306	B306.00	Malignant neoplasm of pelvic bones, sacrum and coccyx	Other/unknown	187952001	288924010
155452016	B18y5	B18y500	Malignant neoplasm of pelvic peritoneum	Other/unknown	449377002	2901085015
722761000006119	B553	B553.00	Malignant neoplasm of pelvis	Other/unknown	363484005	482755017
289445018	B553z	B553z00	Malignant neoplasm of pelvis NOS	Other/unknown	363484005	482755017
288930010	B306z	B306z00	Malignant neoplasm of pelvis, sacrum or coccyx NOS	Other/unknown	187952001	288924010
289260018	B48z	B48z.00	Malignant neoplasm of penis and other male genital organ NOS	Other/unknown	363515000	482817018
289242018	B48	B48..00	Malignant neoplasm of penis and other male genital organs	Other/unknown	363515000	482817018
155459013	B2413	B241300	Malignant neoplasm of pericardium	Other/unknown	93957005	155459013
288741018	B1801	B180100	Malignant neoplasm of perinephric tissue	Other/unknown	187803004	288741018
289389019	B5244	B524400	Malignant neoplasm of peripheral nerve of abdomen	Other/unknown	188326001	289389019

289390011	B5245	B524500	Malignant neoplasm of peripheral nerve of pelvis	Other/unknown	188327005	289390011
289388010	B5243	B524300	Malignant neoplasm of peripheral nerve of thorax	Other/unknown	188325002	289388010
722871000006117	B5242	B524200	Malignant neoplasm of peripheral nerves of lower limb, including hip	Other/unknown	188324003	289387017
722901000006117	B5241	B524100	Malignant neoplasm of peripheral nerves of upper limb, including shoulder	Other/unknown	188323009	289386014
288952018	B308D	B308D00	Malignant neoplasm of phalanges of foot	Other/unknown	187978005	288952018
288922014	B305D	B305D00	Malignant neoplasm of phalanges of hand	Other/unknown	187950009	288922014
723001000006113	B23	B23..00	Malignant neoplasm of pleura	Other/unknown	363433009	482664015
723031000006117	B080	B080.00	Malignant neoplasm of postcricoid region	Other/unknown	363400004	482599012
155473016	B243	B243.00	Malignant neoplasm of posterior mediastinum	Other/unknown	448670003	2901476012
155477015	B5531	B553100	Malignant neoplasm of presacral region	Other/unknown	93973004	155477015
155479017	B3062	B306200	Malignant neoplasm of pubis	Other/unknown	93975006	155479017
155483017	B3043	B304300	Malignant neoplasm of radius	Other/unknown	93979000	155483017
288858018	B2zz	B2zz.00	Malignant neoplasm of respiratory system	Other/unknown	449096009	2899758015
722521000006116	B505	B505.00	Malignant neoplasm of retina	Other/unknown	363465007	482726013
288742013	B1802	B180200	Malignant neoplasm of retrocaecal tissue	Other/unknown	187804005	288742013
722541000006111	B056	B056.00	Malignant neoplasm of retromolar area	Other/unknown	363391009	482581016
1228549016	B180	B180.00	Malignant neoplasm of retroperitoneum	Other/unknown	363420003	1228549016
288739019	B18	B18..00	Malignant neoplasm of retroperitoneum and peritoneum	Other/unknown	187801002	288739019
288744014	B180z	B180z00	Malignant neoplasm of retroperitoneum NOS	Other/unknown	363420003	482639018
155495017	B3030	B303000	Malignant neoplasm of rib	Other/unknown	93990005	155495017
288897014	B303z	B303z00	Malignant neoplasm of rib, sternum and clavicle NOS	Other/unknown	187920001	288889019
288889019	B303	B303.00	Malignant neoplasm of ribs, sternum and clavicle	Other/unknown	187920001	288889019
288928013	B3063	B306300	Malignant neoplasm of sacral vertebra	Other/unknown	187956003	288928013
155503017	B3040	B304000	Malignant neoplasm of scapula	Other/unknown	93997008	155503017
288898016	B304	B304.00	Malignant neoplasm of scapula and long bones of upper arm	Other/unknown	187929000	288898016
289252019	B48y0	B48y000	Malignant neoplasm of seminal vesicle	Other/unknown	188234005	289252019
721961000006111	B2002	B200200	Malignant neoplasm of septum of nose	Other/unknown	363423001	482644013
288953011	B308z	B308z00	Malignant neoplasm of short bones of leg NOS	Other/unknown	712525007	3082865010
292111015	Byu3	Byu3.00	Malignant neoplasm of skeletal system	Other/unknown	443679004	2839107012
288747019	B18y	B18y.00	Malignant neoplasm of specified parts of peritoneum	Other/unknown	187808008	288747019
288756010	B18yz	B18yz00	Malignant neoplasm of specified parts of peritoneum NOS	Other/unknown	187808008	288747019
289452016	B55yz	B55yz00	Malignant neoplasm of specified site NOS	Other/unknown	363346000	482491013
721701000006112	B485	B485.00	Malignant neoplasm of spermatic cord	Other/unknown	363453008	482702011
155582012	B3007	B300700	Malignant neoplasm of sphenoid bone	Other/unknown	94066004	155582012
288760013	B1z1	B1z1.00	Malignant neoplasm of spleen NEC	Other/unknown	363499005	482785010
155592016	B3031	B303100	Malignant neoplasm of sternum	Other/unknown	94073009	155592016
155604019	B3081	B308100	Malignant neoplasm of talus	Other/unknown	94083008	155604019
288963015	B3104	B310400	Malignant neoplasm of tarsus of eyelid	Other/unknown	187988006	288963015
155607014	B3008	B300800	Malignant neoplasm of temporal bone	Other/unknown	94085001	155607014
288754013	B18y6	B18y600	Malignant neoplasm of the pouch of Douglas	Other/unknown	187814001	288754013
729531000006110	B2	B2...00	Malignant neoplasm of thoracic cavity structure	Other/unknown	428100006	2691955010
288886014	B3021	B302100	Malignant neoplasm of thoracic vertebra	Other/unknown	187917009	288886014
289432017	B551	B551.00	Malignant neoplasm of thorax	Other/unknown	188361007	289432017
289438018	B551z	B551z00	Malignant neoplasm of thorax NOS	Other/unknown	188361007	289432017
721271000006114	B240	B240.00	Malignant neoplasm of thymus	Other/unknown	363434003	482666018

288841011	B24	B24..00	Malignant neoplasm of thymus, heart and mediastinum	Other/unknown	187881004	288841011
288802014	B2133	B213300	Malignant neoplasm of thyroid cartilage	Other/unknown	187846001	288802014
155624015	B3072	B307200	Malignant neoplasm of tibia	Other/unknown	94099002	155624015
721391000006116	B220	B220.00	Malignant neoplasm of trachea	Other/unknown	363432004	482662016
288811014	B220z	B220z00	Malignant neoplasm of trachea NOS	Other/unknown	363432004	482662016
289257013	B48y1	B48y100	Malignant neoplasm of tunica vaginalis	Other/unknown	188235006	289257013
2475564017	B300B	B300B00	Malignant neoplasm of turbinate	Other/unknown	187830009	2475564017
288787012	B2012	B201200	Malignant neoplasm of tympanic antrum	Other/unknown	187836003	288787012
288784017	B2011	B201100	Malignant neoplasm of tympanic cavity	Other/unknown	187835004	288784017
155641018	B3044	B304400	Malignant neoplasm of ulna	Other/unknown	94112009	155641018
289561018	B59	B59..00	Malignant neoplasm of unspecified site	Other/unknown	302817000	444727016
289571016	B59z	B59z.00	Malignant neoplasm of unspecified site NOS	Other/unknown	363346000	482491013
720951000006119	B497	B497.00	Malignant neoplasm of urachus	Other/unknown	363456000	482708010
721481000006111	B063	B063.00	Malignant neoplasm of vallecula	Other/unknown	363395000	482589019
720341000006115	B302	B302.00	Malignant neoplasm of vertebral column	Other/unknown	363438000	482674017
288888010	B302z	B302z00	Malignant neoplasm of vertebral column NOS	Other/unknown	363438000	482674017
155677013	B300C	B300C00	Malignant neoplasm of vomer	Other/unknown	94142007	155677013
288578017	B0z1	B0z1.00	Malignant neoplasm of Waldeyer's ring	Other/unknown	187716008	288578017
288896017	B3035	B303500	Malignant neoplasm of xiphoid process	Other/unknown	187927003	288896017
155681013	B3009	B300900	Malignant neoplasm of zygomatic bone	Other/unknown	94145009	155681013
720461000006110	B1zy	B1zy.00	Malignant neoplasm other spec digestive tract and peritoneum	Other/unknown	255077007	380111011
288790018	B206	B206.00	Malignant neoplasm, overlapping lesion of accessory sinuses	Other/unknown	187838002	288790018
1219537012	B163	B163.00	Malignant neoplasm, overlapping lesion of biliary tract	Other/unknown	109847004	1219537012
288764016	B1z2	B1z2.00	Malignant neoplasm, overlapping lesion of digestive system	Other/unknown	187824009	288764016
1217367011	B508	B508.00	Malignant neoplasm, overlapping lesion of eye and adnexa	Other/unknown	109948008	1217367011
730001000006117	B25	B25..00	Malignant neoplasm, overlapping lesion of heart, mediastinum and pleura	Other/unknown	109384006	1217361012
720591000006115	B48y2	B48y200	Malignant neoplasm, overlapping lesion of male genital organs	Other/unknown	109874003	1219539010
1219467016	B074	B074.00	Malignant neoplasm, overlapping lesion of nasopharynx	Other/unknown	109367000	1219467016
1219540012	B487	B487.00	Malignant neoplasm, overlapping lesion of penis	Other/unknown	109875002	1219540012
720001000006119	B5246	B524600	Malignant neoplasm, overlapping lesion of peripheral nerves and autonomic nervous system	Other/unknown	109919002	1219543014
1219535016	B124	B124.00	Malignant neoplasm, overlapping lesion of small intestine	Other/unknown	109837002	1219535016
720031000006110	B30W	B30W.00	Malignant neoplasm/overlap lesion/bone+articulr cartilage	Other/unknown	109347009	173899016
720041000006117	B45X	B45X.00	Malignant neoplasm/overlapping lesion/feml genital organs	Other/unknown	109878000	174564019
881291000006118	BB02-99	BB02.99	Malignant neoplasms	Other/unknown	86049000	881291000006118
289566011	B592	B592.00	Malignant neoplasms of independent (primary) multiple sites	Other/unknown	188478004	289566011
406265010	B5	B5...00	Malignant neoplastic disease	Other/unknown	363346000	1208875016
8041921000006114	^ESCTMA804192		Malignant neuroendocrine tumour	Other/unknown	133531000119104	3289508017
138104014	H51y7	H51y700	Malignant pleural effusion	Other/unknown	83270006	138104014
445587010	B62x4	B62x400	Malignant reticulosis	Other/unknown	118612006	445587010
2549374014	B3065	B306500	Malignant sacral teratoma	Other/unknown	416842003	2549374014

317611000006119	BBB61	BBB6100	Malignant thymoma	Other/unknown	15949004	1221248015
312071000006117	BB08	BB08.00	Malignant tumour - small cell type	Other/unknown	82267002	1216992014
725811000006116	B0621	B062100	Malignant tumour of anterior pillar of fauces	Other/unknown	254459004	378845014
288561014	B073z	B073z00	Malignant tumour of anterior wall of nasopharynx	Other/unknown	187700006	288553016
289435015	B5510	B551000	Malignant tumour of axilla	Other/unknown	363502009	482790013
396521000006110	ByuA0	ByuA000	Malignant tumour of cranial nerve	Other/unknown	188307009	289367018
288847010	B241z	B241z00	Malignant tumour of heart	Other/unknown	363435002	482668017
288803016	B213z	B213z00	Malignant tumour of laryngeal cartilage	Other/unknown	363431006	482660012
289447014	B555	B555.00	Malignant tumour of lower limb	Other/unknown	363504005	482794016
396101000006112	ByuDB	ByuDB00	Malignant tumour of lymphoid haemopoietic and related tissue	Other/unknown	269475001	403706019
292143015	Byu8	Byu8.00	Malignant tumour of male genital organ	Other/unknown	363515000	482818011
292110019	Byu25	Byu2500	Malignant tumour of mediastinum	Other/unknown	363494000	482776010
292152012	ByuA2	ByuA200	Malignant tumour of meninges	Other/unknown	363497007	482781018
289429015	B5504	B550400	Malignant tumour of neck	Other/unknown	363489000	482765011
289249010	B483	B483.00	Malignant tumour of penis	Other/unknown	363516004	482819015
6247341000006114	^ESCTMA624734		Malignant tumour of peritoneum	Other/unknown	363492001	482772012
288757018	B18z	B18z.00	Malignant tumour of peritoneum and retroperitoneum	Other/unknown	187801002	288737017
288840012	B23z	B23z.00	Malignant tumour of pleura	Other/unknown	363433009	482664015
288763010	B1z1z	B1z1z00	Malignant tumour of spleen	Other/unknown	363499005	482785010
4747901000006113	^ESCTMA474790		Malignant tumour of supraglottis	Other/unknown	187842004	288796012
380059013	B595	B595.00	Malignant tumour of unknown origin	Other/unknown	255052006	380059013
291538016	BB9B	BB9B.00	Medullary carcinoma	Other/unknown	32913002	54929015
403762019	Byu50	Byu5000	Mesothelioma (malignant, clinical disorder)	Other/unknown	109378008	2660053016
173941016	B2414	B241400	Mesothelioma of pericardium	Other/unknown	109383000	173941016
174532014	B181	B181.00	Mesothelioma of peritoneum	Other/unknown	109853004	174532014
312601000006119	BBP1	BBP1.00	Mesothelioma, malignant	Other/unknown	62064005	103178011
704711000006119	B57-1	B57..11	Metastases of respiratory and/or digestive systems	Other/unknown	269473008	403703010
6052771000006112	^ESCTME605277		Metastasis from malignant tumour of lung	Other/unknown	315006004	459462017
4031161000006115	^ESCTME403116		Metastasis to brain	Other/unknown	94225005	1235818010
157761000006113	B560	B560.00	Metastasis to head and neck lymph node	Other/unknown	303194003	445248014
739801000006115	B577-1	B577.11	Metastasis to liver	Other/unknown	94381002	511275014
7969741000006112	^ESCTME796974		Metastasis to lung from adenocarcinoma	Other/unknown	1661000119106	2967714019
5703481000006115	^ESCTME570348		Metastasis to lung of unknown primary	Other/unknown	285604008	424639014
156825010	B5831	B583100	Metastasis to spinal cord	Other/unknown	94600009	1235865016
4409561000006113	^ESCTME440956		Metastatic cancer	Other/unknown	128462008	2162156010
881301000006117	BB13-99	BB13.99	Metastatic Carcinoma	Other/unknown	463191000000105	881301000006117
14841931000006114	^ESCT1484193		Metastatic malignant neoplasm	Other/unknown	128462008	5084452013
4031011000006117	^ESCTME403101		Metastatic malignant neoplasm to bone	Other/unknown	94222008	155872017
4031141000006119	^ESCTME403114		Metastatic malignant neoplasm to brain	Other/unknown	94225005	155882016
4034891000006116	^ESCTME403489		Metastatic malignant neoplasm to liver	Other/unknown	94381002	156294011
4035171000006112	^ESCTME403517		Metastatic malignant neoplasm to lung	Other/unknown	94391008	156322019
6689401000006119	^ESCTME668940		Metastatic squamous cell carcinoma	Other/unknown	403906006	1782910017
4031021000006113	^ESCTME403102		Metastatic tumour of bone	Other/unknown	94222008	1217283014
313041000006119	BB821	BB82100	Mucinous adenocarcinoma	Other/unknown	72495009	120422012
313131000006113	BB71	BB71.00	Mucoepidermoid carcinoma	Other/unknown	4079000	8016013
313711000006115	BB03	BB03.00	Neoplasm, metastatic	Other/unknown	14799000	25140016
144849016	B546	B546.00	Neuroblastoma	Other/unknown	87364003	144849016
3408821000006112	^ESCTNE340882		Neuroendocrine carcinoma	Other/unknown	55937004	93038015
5324321000006115	^ESCTNE532432		Neuroendocrine carcinoma	Other/unknown	253000007	376795013
1887221000006112	BBz0	BBz0.00	Neuroendocrine neoplasm	Other/unknown	128928004	207611012
1870381000006113	BB1P	BB1P.00	Non-small cell carcinoma	Other/unknown	128632008	207023019
314111000006116	BB1K	BB1K.00	Oat cell carcinoma	Other/unknown	76817009	127539014

289565010	B591	B591.00	Other malignant neoplasm NOS	Other/unknown	363346000	1228480011
247091000006118	B182	B182.00	Overlapping malignant lesion of retroperitoneum and peritoneum	Other/unknown	187807003	288746011
396551000006118	Byu32	Byu3200	Overlapping malignant neoplasm of bone and articular cartilage	Other/unknown	109347009	173899016
720631000006115	B51y2	B51y200	Overlapping malignant neoplasm of brain	Other/unknown	109912006	174605010
396161000006113	ByuA3	ByuA300	Overlapping malignant neoplasm of brain and other parts of the central nervous system	Other/unknown	109911004	174604014
396571000006111	ByuC1	ByuC100	Overlapping malignant neoplasm of ill-defined site	Other/unknown	109358000	173910017
459732012	1D18	1D18.00	Pain from metastases	Other/unknown	315241008	459732012
314791000006110	BB5f6	BB5f600	Papillary and follicular adenocarcinoma	Other/unknown	189643000	291453010
291343010	BB22	BB22.00	Papillary carcinoma	Other/unknown	25910003	43414013
216227011	B5850	B585000	Pathological fracture due to metastatic bone disease	Other/unknown	134421000	216227011
13950731000006110	^ESCT1395073		Pleural effusion due to malignant neoplastic disease	Other/unknown	860792009	3944188015
342851019	B1502	B150200	Primary angiosarcoma of liver	Other/unknown	109844006	342851019
720581000006118	B26	B26..00	Primary malignant neoplasm of intrathoracic organs	Other/unknown	93841009	510723012
454091013	B593	B593.00	Primary malignant neoplasm of unknown site	Other/unknown	310504009	454091013
4035201000006111	^ESCTPU403520		Pulmonary metastasis	Other/unknown	94391008	1235838011
289582012	B6001	B600100	Reticulosarcoma of lymph nodes of head, face and neck	Other/unknown	188489006	289582012
291855013	BBc9z	BBc9z00	Retinoblastoma	Other/unknown	19906005	33508018
157511000006115	B563z	B563z00	Secondary and unspec malig neop axilla and upper limb LN NOS	Other/unknown	94398002	156345013
157531000006114	B5618	B561800	Secondary and unspec malig neop bronchopulmonary lymph nodes	Other/unknown	94227002	155885019
157611000006119	B5624	B562400	Secondary and unspec malig neop external iliac lymph nodes	Other/unknown	94336001	156171017
157661000006116	B564	B564.00	Secondary and unspec malig neop inguinal and lower limb LN	Other/unknown	94395004	156335011
157701000006112	B562z	B562z00	Secondary and unspec malig neop intra-abdominal LN NOS	Other/unknown	94347008	156199014
157711000006110	B562	B562.00	Secondary and unspec malig neop intra-abdominal lymph nodes	Other/unknown	94347008	156199014
157721000006119	B565z	B565z00	Secondary and unspec malig neop intrapelvic LN NOS	Other/unknown	94350006	156209013
157731000006116	B565	B565.00	Secondary and unspec malig neop intrapelvic lymph nodes	Other/unknown	94350006	156209013
157741000006114	B561z	B561z00	Secondary and unspec malig neop intrathoracic LN NOS	Other/unknown	94351005	156213018
157751000006111	B561	B561.00	Secondary and unspec malig neop intrathoracic lymph nodes	Other/unknown	94351005	156213018
157771000006118	B56y	B56y.00	Secondary and unspec malig neop lymph nodes multiple sites	Other/unknown	303201005	445249018
157781000006115	B56z	B56z.00	Secondary and unspec malig neop lymph nodes NOS	Other/unknown	94392001	156323012
157811000006118	B564z	B564z00	Secondary and unspec malig neop of inguinal and leg LN NOS	Other/unknown	94395004	156335011
157861000006115	B5614	B561400	Secondary and unspec malig neop post mediastinal lymph nodes	Other/unknown	94408005	156373010
880681000006114	B577-99	B577.99	Secondary Ca liver	Other/unknown	94381002	880681000006114
289554017	B58y2-1	B58y211	Secondary cancer of the cervix	Other/unknown	188469005	289554017
511385013	B58y4-1	B58y411	Secondary cancer of the vulva	Other/unknown	94681006	511385013

380206018	B58-1	B58..11	Secondary carcinoma of other specified sites	Other/unknown	128462008	206783017
380193010	B57-2	B57..12	Secondary carcinoma of respiratory and/or digestive systems	Other/unknown	269473008	403703010
151101000006116	B575z	B575z00	Secondary malig neop of large intestine or rectum NOS	Other/unknown	94365007	156249017
151121000006114	B57z	B57z.00	Secondary malig neop of respiratory or digestive system NOS	Other/unknown	269473008	403703010
151141000006119	B576z	B576z00	Secondary malig neop of retroperitoneum or peritoneum NOS	Other/unknown	188445006	289527015
151151000006117	B574z	B574z00	Secondary malig neop of small intestine or duodenum NOS	Other/unknown	94580002	156781013
155711012	B587	B587.00	Secondary malignant neoplasm of adrenal gland	Other/unknown	94161006	155711012
157521000006111	B5630	B563000	Secondary malignant neoplasm of axillary lymph nodes	Other/unknown	94181007	155759012
155773015	B5811	B581100	Secondary malignant neoplasm of bladder	Other/unknown	94186002	155773015
1235814012	B585	B585.00	Secondary malignant neoplasm of bone and bone marrow	Other/unknown	94222008	1235814012
155879014	B5830	B583000	Secondary malignant neoplasm of brain	Other/unknown	94225005	155879014
289546019	B583	B583.00	Secondary malignant neoplasm of brain and spinal cord	Other/unknown	188462001	289546019
289549014	B583z	B583z00	Secondary malignant neoplasm of brain or spinal cord NOS	Other/unknown	188462001	289546019
511250010	B58y0	B58y000	Secondary malignant neoplasm of breast	Other/unknown	94297009	511250010
157871000006110	B5619	B561900	Secondary malignant neoplasm of bronchopulmonary lymph nodes	Other/unknown	94227002	155885019
289553011	B58y2	B58y200	Secondary malignant neoplasm of cervix uteri	Other/unknown	188469005	289553011
155967013	B5750	B575000	Secondary malignant neoplasm of colon	Other/unknown	94260004	155967013
156007019	B5740	B574000	Secondary malignant neoplasm of duodenum	Other/unknown	94275007	156007019
289557012	B58y8	B58y800	Secondary malignant neoplasm of epididymis and vas deferens	Other/unknown	188471005	2668736017
289530010	B57y	B57y.00	Secondary malignant neoplasm of gastrointestinal tract	Other/unknown	94313005	156111011
425781000006111	ByuC4	ByuC400	Secondary malignant neoplasm of gastrointestinal tract	Other/unknown	94313005	156111011
156169017	B5742	B574200	Secondary malignant neoplasm of ileum	Other/unknown	94335002	156169017
157561000006117	B5623	B562300	Secondary malignant neoplasm of iliac lymph nodes	Other/unknown	94336001	156171017
157651000006118	B5632	B563200	Secondary malignant neoplasm of infraclavicular lymph nodes	Other/unknown	94338000	156175014
156237018	B580	B580.00	Secondary malignant neoplasm of kidney	Other/unknown	94360002	156237018
151321000006111	B575	B575.00	Secondary malignant neoplasm of large intestine	Other/unknown	94365007	156249017
156291015	B577	B577.00	Secondary malignant neoplasm of liver	Other/unknown	94381002	156291015
151331000006114	B153	B153.00	Secondary malignant neoplasm of liver	Other/unknown	94381002	156291015
156319016	B570	B570.00	Secondary malignant neoplasm of lung	Other/unknown	94391008	156319016
406333010	B56	B56..00	Secondary malignant neoplasm of lymph node	Other/unknown	94392001	156323012
157801000006116	B5606	B560600	Secondary malignant neoplasm of lymph nodes of face	Other/unknown	94393006	156327013
359291000006111	ByuC2	ByuC200	Secondary malignant neoplasm of lymph nodes of multiple sites	Other/unknown	94396003	156339017
157501000006118	B563	B563.00	Secondary malignant neoplasm of lymph nodes of upper limb	Other/unknown	94398002	156345013
157481000006111	B5613	B561300	Secondary malignant neoplasm of mediastinal lymph nodes	Other/unknown	94408005	156373010

156375015	B571	B571.00	Secondary malignant neoplasm of mediastinum	Other/unknown	94409002	156375015
157631000006113	B5622	B562200	Secondary malignant neoplasm of mesenteric lymph nodes	Other/unknown	94410007	156379014
157941000006112	B5621	B562100	Secondary malignant neoplasm of mesenteric lymph nodes	Other/unknown	94410007	156379014
359281000006113	ByuC6	ByuC600	Secondary malignant neoplasm of nervous system	Other/unknown	94442001	156461011
157971000006116	B5603	B560300	Secondary malignant neoplasm of occipital lymph nodes	Other/unknown	94449005	156479010
289550014	B584	B584.00	Secondary malignant neoplasm of other part of nervous system	Other/unknown	94442001	156461011
289559010	B58z	B58z.00	Secondary malignant neoplasm of other specified site NOS	Other/unknown	128462008	206784011
151401000006110	B58yz	B58yz00	Secondary malignant neoplasm of other specified site NOS	Other/unknown	128462008	206784011
406266011	B58y	B58y.00	Secondary malignant neoplasm of other specified sites	Other/unknown	128462008	206784011
289536016	B581z	B581z00	Secondary malignant neoplasm of other urinary organ NOS	Other/unknown	94663008	156981017
289535017	B581	B581.00	Secondary malignant neoplasm of other urinary organs	Other/unknown	94663008	156981017
156493015	B586	B586.00	Secondary malignant neoplasm of ovary	Other/unknown	94455000	156493015
157591000006113	B5604	B560400	Secondary malignant neoplasm of parotid lymph nodes	Other/unknown	94475009	156541015
157821000006114	B5600	B560000	Secondary malignant neoplasm of parotid lymph nodes	Other/unknown	94475009	156541015
157841000006119	B5633	B563300	Secondary malignant neoplasm of pectoral axillary lymph nodes	Other/unknown	94477001	156545012
156557015	B58y7	B58y700	Secondary malignant neoplasm of penis	Other/unknown	94481001	156557015
1235875018	B5761	B576100	Secondary malignant neoplasm of peritoneum	Other/unknown	94627008	1235875018
156585018	B572	B572.00	Secondary malignant neoplasm of pleura	Other/unknown	94493005	156585018
156607018	B58y5	B58y500	Secondary malignant neoplasm of prostate	Other/unknown	94503003	156607018
156627017	B5751	B575100	Secondary malignant neoplasm of rectum	Other/unknown	94513006	156627017
151111000006118	B57	B57..00	Secondary malignant neoplasm of respiratory and digestive systems	Other/unknown	269473008	403703010
289515018	B573	B573.00	Secondary malignant neoplasm of respiratory tract	Other/unknown	94515004	156631011
425771000006113	ByuC3	ByuC300	Secondary malignant neoplasm of respiratory tract	Other/unknown	94515004	156631011
1215809015	B5760	B576000	Secondary malignant neoplasm of retroperitoneum	Other/unknown	94628003	1215809015
151131000006112	B576	B576.00	Secondary malignant neoplasm of retroperitoneum and peritoneum	Other/unknown	188445006	289527015
156777013	B582	B582.00	Secondary malignant neoplasm of skin	Other/unknown	94579000	156777013
289545015	B582z	B582z00	Secondary malignant neoplasm of skin NOS	Other/unknown	94579000	156777013
156699010	B5826	B582600	Secondary malignant neoplasm of skin of breast	Other/unknown	94544002	156699010
156719018	B5821	B582100	Secondary malignant neoplasm of skin of face	Other/unknown	94554003	156719018
289538015	B5820	B582000	Secondary malignant neoplasm of skin of head	Other/unknown	188454009	289538015
289543010	B5825	B582500	Secondary malignant neoplasm of skin of hip and leg	Other/unknown	188459004	289543010
156747019	B5822	B582200	Secondary malignant neoplasm of skin of neck	Other/unknown	94566009	156747019
289542017	B5824	B582400	Secondary malignant neoplasm of skin of shoulder and arm	Other/unknown	188458007	289542017
156765015	B5823	B582300	Secondary malignant neoplasm of skin of trunk	Other/unknown	94575006	156765015

151621000006119	B574	B574.00	Secondary malignant neoplasm of small intestine	Other/unknown	94580002	156781013
157891000006111	B5605	B560500	Secondary malignant neoplasm of submandibular lymph nodes	Other/unknown	94609005	156851012
157901000006110	B5607	B560700	Secondary malignant neoplasm of submental lymph nodes	Other/unknown	94611001	156855015
157931000006119	B5640	B564000	Secondary malignant neoplasm of superficial inguinal lymph nodes	Other/unknown	94612008	156857011
156883018	B58y6	B58y600	Secondary malignant neoplasm of testis	Other/unknown	94623007	156883018
156919012	B58y9	B58y900	Secondary malignant neoplasm of tongue	Other/unknown	94638008	156919012
157641000006115	B5617	B561700	Secondary malignant neoplasm of tracheobronchial lymph nodes	Other/unknown	94642006	156931014
157911000006113	B5616	B561600	Secondary malignant neoplasm of tracheobronchial lymph nodes	Other/unknown	94642006	156931014
409845013	B594	B594.00	Secondary malignant neoplasm of unknown site	Other/unknown	274088005	409845013
156973018	B5810	B581000	Secondary malignant neoplasm of ureter	Other/unknown	94659001	156973018
156977017	B5812	B581200	Secondary malignant neoplasm of urethra	Other/unknown	94661005	156977017
359271000006110	ByuC5	ByuC500	Secondary malignant neoplasm of urinary system	Other/unknown	94663008	156981017
156989015	B58y1	B58y100	Secondary malignant neoplasm of uterus	Other/unknown	94665001	156989015
156997010	B58y3	B58y300	Secondary malignant neoplasm of vagina	Other/unknown	94668004	156997010
157025019	B58y4	B58y400	Secondary malignant neoplasm of vulva	Other/unknown	94681006	157025019
151421000006117	B58	B58..00	Secondary malignant neoplastic disease	Other/unknown	128462008	206784011
152611000006116	B560z	B560z00	Secondary unspec malig neop lymph nodes head/face/neck NOS	Other/unknown	303194003	445248014
291332019	BB1J	BB1J.00	Small cell carcinoma	Other/unknown	74364000	123497012
316761000006111	BB1D	BB1D.00	Spindle cell carcinoma	Other/unknown	65692009	109146010
291348018	BB2B	BB2B.00	Squamous cell carcinoma, metastatic	Other/unknown	64204000	106728012
1772981000006110	EMISNQTH25		Thymoma	Other/unknown	444231005	2841385011
81401000006110	B62z3	B62z300	Unspec malig neop lymphoid/histiocytic intra-abdominal nodes	Other/unknown	118600007	177018013
81421000006117	B62z1	B62z100	Unspec malig neop lymphoid/histiocytic lymph node head/neck	Other/unknown	118600007	177018013
81431000006119	B62z5	B62z500	Unspec malig neop lymphoid/histiocytic nodes inguinal/leg	Other/unknown	118600007	177018013
81461000006111	B62z8	B62z800	Unspec malig neop lymphoid/histiocytic of multiple sites	Other/unknown	118600007	177018013
552661000006114	B420	B420.00	Choriocarcinoma	Other/unknown	188188009	289184018
1216940017	BBr27	BBr2700	[M]Adult T-cell leukaemia/lymphoma	Other haematological	77430005	1216940017
313271000006116	BBn0-1	BBn0.11	[M]Multiple myeloma	Other haematological	109989006	344820010
313381000006111	BBn0-2	BBn0.12	[M]Myeloma NOS	Other haematological	55921005	1231541019
1231541019	BBn0	BBn0.00	[M]Plasma cell myeloma	Other haematological	55921005	1231541019
306981000006119	BBs2	BBs2.00	Chronic myeloproliferative disease	Other haematological	128842008	207443019
648841000006119	B9374	B937400	Essential thrombocythaemia	Other haematological	109994006	1476449014
2535665011	D3y0	D3y0.00	Essential thrombocytosis	Other haematological	109994006	2535665011
289904016	B6531	B653100	Granulocytic sarcoma	Other haematological	188738007	289904016

824231000006111	B625-1	B625.11	Histiocytosis X (acute, progressive)	Other haematological	118614007	177033017
2535666012	B9375	B937500	Idiopathic thrombocythaemia	Other haematological	109994006	2535666012
786661000006115	B937-2	B937.12	Idiopathic thrombocythaemia	Other haematological	109994006	2535666012
1215865012	BBs4	BBs4.00	Idiopathic thrombocythaemia	Other haematological	128844009	474634018
5701111000006119	^ESCTIG570111		IgA myeloma	Other haematological	285420006	424415012
5701131000006113	^ESCTIG570113		IgG myeloma	Other haematological	285421005	424416013
344818012	B630-1	B630.11	Kahler's disease	Other haematological	109989006	344818012
6860401000006118	^ESCTKA686040		Kappa light chain myeloma	Other haematological	414553000	2534493014
457317011	B6303	B630300	Lambda light chain myeloma	Other haematological	313427003	457317011
967761000006115	C37yB	C37yB00	Langerhans cell histiocytosis	Other haematological	65399007	199591010
745971000006116	B625	B625.00	Letterer-Siwe disease (clinical)	Other haematological	118614007	177033017
289780018	B625z	B625z00	Letterer-Siwe disease NOS	Other haematological	118614007	177033017
154082012	B6252	B625200	Letterer-Siwe disease of intrathoracic lymph nodes	Other haematological	93135004	154082012
154089015	B6258	B625800	Letterer-Siwe disease of lymph nodes of multiple sites	Other haematological	93139005	154089015
289776011	B6250	B625000	Letterer-Siwe disease of unspecified sites	Other haematological	118614007	177033017
403710016	B62zz	B62zz00	Lymphoid and histiocytic malignancy NOS	Other haematological	269475001	403706019
445586018	B623	B623.00	Malignant histiocytosis	Other haematological	118612006	445586018
289754019	B623z	B623z00	Malignant histiocytosis NOS	Other haematological	118612006	177031015
154183017	B6233	B623300	Malignant histiocytosis of intra-abdominal lymph nodes	Other haematological	93182006	154183017
728571000006118	B6231	B623100	Malignant histiocytosis of lymph nodes of head, face and neck	Other haematological	188640007	289751010
289750011	B6230	B623000	Malignant histiocytosis of unspecified site	Other haematological	118612006	177031015
292175017	ByuD4	ByuD400	Malignant immunoproliferative disease (clinical)	Other haematological	109980005	174676012
289826016	B62x5	B62x500	Malignant immunoproliferative small intestinal disease	Other haematological	188691005	289826016
289793011	B626z	B626z00	Malignant mast cell tumour NOS	Other haematological	118615008	177034011
289781019	B626	B626.00	Malignant mast cell tumours	Other haematological	188660004	289781019
289849019	B62z	B62z.00	Malignant neoplasms of lymphoid and histiocytic tissue NOS	Other haematological	118600007	177018013
289867014	B6300	B630000	Malignant plasma cell neoplasm, extramedullary plasmacytoma	Other haematological	188718006	289867014
289783016	B6260	B626000	Mast cell malignancy	Other haematological	397009000	1776745013

716911000006110	B6265	B626500	Mast cell malignancy of lymph nodes of inguinal region and lower limb	Other haematological	188666005	289788013
289791013	B6268	B626800	Mast cell malignancy of lymph nodes of multiple sites	Other haematological	188669003	289791013
291959018	BBm5	BBm5.00	Mature (peripheral) T-cell neoplasm	Other haematological	414655002	2535406015
344820010	B630	B630.00	Multiple myeloma	Other haematological	109989006	344820010
695561000006118	B63	B63..00	Multiple myeloma and immunoproliferative neoplasms	Other haematological	109989006	344820010
683051000006114	B937W-1	B937W11	Myelodysplasia	Other haematological	109995007	174702016
313331000006110	BBv	BBv..00	Myelodysplastic syndrome	Other haematological	128623006	1208660014
683071000006116	B937W	B937W00	Myelodysplastic syndrome	Other haematological	109995007	2659757019
683041000006112	B937-4	B937.14	Myelodysplastic syndrome (clinical)	Other haematological	109995007	174702016
1815691000006116	B677	B677.00	Myelodysplastic/myeloproliferative disease	Other haematological	445738007	2882898013
88121017	D41y1	D41y100	Myelofibrosis	Other haematological	52967002	88121017
683191000006119	B936-1	B936.11	Myeloma - solitary	Other haematological	415112005	2534490012
344819016	B630-2	B630.12	Myelomatosis	Other haematological	109989006	344819016
683351000006111	B6y0-1	B6y0.11	Myeloproliferative disease	Other haematological	425333006	2645960018
2645960018	B6y0	B6y0.00	Myeloproliferative disorder	Other haematological	425333006	2645960018
201743012	B631	B631.00	Plasma cell leukaemia	Other haematological	95210003	201743012
225051000006119	B6302	B630200	Plasmacytoma	Other haematological	415112005	2534490012
18484012	B6304	B630400	Plasmacytoma - disorder	Other haematological	415112005	2537380010
344859018	B934-1	B934.11	Polycythaemia rubra vera	Other haematological	109992005	344859018
344860011	B934	B934.00	Polycythaemia vera	Other haematological	109992005	344860011
215919015	BBs0	BBs0.00	Polycythaemia vera	Other haematological	128841001	474628013
554151000006112	B671	B671.00	Polycythaemia vera (clinical)	Other haematological	109992005	174698015
2535663016	B934-2	B934.12	Primary polycythaemia	Other haematological	109992005	2535663016
206411000006114	B9374-1	B937411	Primary thrombocythaemia	Other haematological	109994006	1476449014
1211679013	B600	B600.00	Reticulosarcoma	Other haematological	373168002	1211679013
289590012	B600z	B600z00	Reticulosarcoma NOS	Other haematological	373168002	1211679013
157745018	B6003	B600300	Reticulosarcoma of intra-abdominal lymph nodes	Other haematological	95224004	157745018
157752016	B6007	B600700	Reticulosarcoma of spleen	Other haematological	95231000	157752016

289581017	B6000	B600000	Reticulosarcoma of unspecified site	Other haematological	373168002	1211679013
6843711000006111	^ESCTSM684371		Smouldering myeloma	Other haematological	413587002	2769066012
139571000006117	B6301	B630100	Solitary myeloma	Other haematological	415112005	2534490012
293380012	C3330	C333000	Waldenstrom's macroglobulinaemia	Other haematological	190818004	293380012
318171000006118	BBmK	BBmK.00	Waldenstrom's macroglobulinaemia	Other haematological	35562000	197158017
312671000006113	BBs	BBs..00	[M]Miscellaneous myeloproliferative and lymphoproliferative disorders	Other haematological	415181008	2534443010
396621000006114	ByuD	ByuD.00	[X]Malignant neoplasms of lymphoid, haematopoietic and related tissue	Other haematological	269475001	403705015
1228570010	B440-1	B440.11	Cancer of ovary	Ovary	363443007	1228570010
723281000006111	B440	B440.00	Malignant tumour of ovary	Ovary	363443007	482684016
6623661000006114	^ESCTAD662366		Adenocarcinoma of prostate	Prostate	399490008	1778899017
6616761000006119	^ESCTCA661676		Cancer of prostate	Prostate	399068003	1786665019
5352541000006110	^ESCTCA535254		Carcinoma of prostate	Prostate	254900004	379766015
1490806016	4M00	4M00.00	Gleason prostate grade 2-4 (low)	Prostate	369775001	1490806016
1488634012	4M01	4M01.00	Gleason prostate grade 5-7 (medium)	Prostate	394932008	1488634012
1490807013	4M02	4M02.00	Gleason prostate grade 8-10 (high)	Prostate	369777009	1490807013
1717461000000112	14270	1427000	H/O: prostate cancer	Prostate	428262008	1717461000000112
722361000006115	B46	B46..00	Malignant tumour of prostate	Prostate	399068003	1773293010
6052511000006110	^ESCTME605251		Metastasis from malignant tumour of prostate	Prostate	314994000	459437017
6052541000006114	^ESCTME605254		Metastatic prostate cancer	Prostate	314994000	3036413018
4027551000006117	^ESCTPR402755		Primary malignant neoplasm of prostate	Prostate	93974005	510942015
5352571000006119	^ESCTPR535257		Prostate cancer	Prostate	254900004	3036967016
7696221000006119	^ESCTPR769622		Prostate cancer metastatic to bone	Prostate	712849003	3286034016
318211000006116	BBL71-2	BBL7112	Wilms' tumour	Renal	302849000	444813019
880481000006111	B4A0-99	B4A0.99	Ca kidney	Renal	188250002	880481000006111
5352831000006119	^ESCTCL535283		Clear cell carcinoma of kidney	Renal	254915003	379798014
289288012	B4A00	B4A0000	Hypernephroma	Renal	702391001	3648998018
289285010	B4A0	B4A0.00	Malignant neoplasm of kidney parenchyma	Renal	188250002	289285010
177051000006111	B4A-1	B4A..11	Malignant tumour of kidney	Renal	363518003	482823011
4750361000006114	^ESCTMA475036		Malignant tumour of kidney parenchyma	Renal	188250002	289286011
7559611000006116	^ESCTME755961		Metastatic renal cell carcinoma	Renal	702392008	2995513016
7559601000006119	^ESCTRE755960		Renal cell carcinoma	Renal	702391001	2995609017
315861000006111	BB5a0	BB5a000	Renal cell carcinoma - morphology	Renal	41607009	1229779019
1228510019	B02	B02..00	Malignant neoplasm of major salivary glands	Salivary	363378008	1228510019
288437016	B02y	B02y.00	Malignant neoplasm of other major salivary glands	Salivary	363378008	482552017
721881000006113	B022	B022.00	Malignant neoplasm of sublingual gland	Salivary	363381003	482558018
1228513017	B021	B021.00	Malignant neoplasm of submandibular gland	Salivary	363380002	1228513017
288438014	B02z	B02z.00	Malignant tumour of major salivary gland	Salivary	363378008	482553010
723501000006117	B020	B020.00	Malignant tumour of parotid gland	Salivary	363379000	482554016
291782010	BBW4	BBW4.00	[M]Chondrosarcoma NOS	Sarcoma	14990007	25445016
1232905013	BBJH	BBJH.00	[M]Dedifferentiated liposarcoma	Sarcoma	67280001	1232906014
1231237017	BBG1	BBG1.00	[M]Fibrosarcoma NOS	Sarcoma	53654007	1231237017
1230800010	BBTA	BBTA.00	[M]Kaposi's sarcoma	Sarcoma	49937004	1230800010
291638011	BBJ1	BBJ1.00	[M]Liposarcoma NOS	Sarcoma	49430005	82332016
1222660012	BBV1	BBV1.00	[M]Osteosarcoma NOS	Sarcoma	21708004	1222660012
291624016	BBFz	BBFz.00	[M]Soft tissue tumour or sarcoma NOS	Sarcoma	115224008	175980010
291618010	BBF	BBF..00	[M]Soft tissue tumours and sarcomas NOS	Sarcoma	115224008	175980010
1232439019	BBN1	BBN1.00	[M]Synovial sarcoma NOS	Sarcoma	63211008	1232439019
292128017	Byu53	Byu5300	[X]Kaposi's sarcoma, unspecified	Sarcoma	109385007	202818010

305551000006110	BBT1-1	BBT1.11	Angiosarcoma	Sarcoma	39000009	65416019
291676017	BBL9	BBL9.00	Carcinosarcoma	Sarcoma	63264007	105165013
306771000006119	BBa5	BBa5.00	Chordoma	Sarcoma	50007008	83295011
215889013	BBGM	BBGM.00	Dermatofibrosarcoma	Sarcoma	76594008	127194014
413089014	B339	B339.00	Dermatofibrosarcoma protuberans	Sarcoma	276799004	413089014
307821000006112	BBK36	BBK3600	Embryonal rhabdomyosarcoma	Sarcoma	14269005	24268019
307891000006114	BBL0	BBL0.00	Endometrial stromal sarcoma	Sarcoma	70555003	117193019
308351000006112	BBY0	BBY0.00	Ewing's sarcoma	Sarcoma	76909002	127685019
288762017	B1z11	B1z1100	Fibrosarcoma of spleen	Sarcoma	187822008	288762017
2576875014	B9054	B905400	Gastrointestinal stromal tumour	Sarcoma	420120006	2576875014
292136014	Byu5B	Byu5B00	Kaposi's sarcoma (clinical)	Sarcoma	109385007	202818010
173950019	B6z0	B6z0.00	Kaposi's sarcoma of lymph nodes	Sarcoma	109391009	173950019
755631000006110	B592X	B592X00	Kaposi's sarcoma of multiple organs	Sarcoma	109392002	173951015
173946014	B05z0	B05z000	Kaposi's sarcoma of palate	Sarcoma	109388009	173946014
173944012	B33z0	B33z000	Kaposi's sarcoma of skin	Sarcoma	109386008	173944012
289008015	B31z0	B31z000	Kaposi's sarcoma of soft tissue	Sarcoma	188029000	289008015
755671000006113	B59zX	B59zX00	Kaposi's sarcoma, unspecified	Sarcoma	109385007	202818010
7281991000006118	^ESCTLE728199		Leiomyosarcoma	Sarcoma	443719001	2840084012
291653015	BBK02	BBK0200	Leiomyosarcoma - category	Sarcoma	400011005	1779284015
722911000006119	B5240	B524000	Malignant neoplasm of peripheral nerves of head, face and neck	Sarcoma	188322004	289385013
313521000006119	BBV9	BBV9.00	Myxoid chondrosarcoma	Sarcoma	75622000	125602017
313541000006114	BBJ5	BBJ5.00	Myxoid liposarcoma	Sarcoma	27849002	46609012
2164653019	B30z0	B30z000	Osteosarcoma	Sarcoma	307576001	2164653019
315511000006111	BBF4-1	BBF4.11	Pleomorphic cell sarcoma	Sarcoma	87992000	145862011
215896010	BBK31	BBK3100	Rhabdomyosarcoma	Sarcoma	30924005	51781010
291622017	BBF1	BBF1.00	Sarcoma - category	Sarcoma	372151005	2672980015
380093012	B3-2	B3...12	Sarcoma of bone and connective tissue	Sarcoma	255067005	380093012
316821000006112	BBF3	BBF3.00	Spindle cell sarcoma	Sarcoma	9801004	17126017
6765161000006117	^ESCTAD676516		Adenocarcinoma of stomach	Stomach	408647009	2160194015
288647013	B114	B114.00	Malignant neoplasm of body of stomach	Stomach	187742008	288647013
288625013	B110	B110.00	Malignant neoplasm of cardia of stomach	Stomach	187732006	288625013
288633014	B110z	B110z00	Malignant neoplasm of cardia of stomach NOS	Stomach	187732006	288625013
288628010	B1100	B110000	Malignant neoplasm of cardiac orifice of stomach	Stomach	187733001	288628010
288632016	B1101	B110100	Malignant neoplasm of cardio-oesophageal junction of stomach	Stomach	187734007	288632016
288644018	B113	B113.00	Malignant neoplasm of fundus of stomach	Stomach	187741001	288644018
288631011	B1101-1	B110111	Malignant neoplasm of gastro-oesophageal junction	Stomach	187734007	288631011
288650011	B116	B116.00	Malignant neoplasm of greater curve of stomach unspecified	Stomach	269460009	403680015
288652015	B11y	B11y.00	Malignant neoplasm of other specified site of stomach	Stomach	363349007	482497012
288655018	B11yz	B11yz00	Malignant neoplasm of other specified site of stomach NOS	Stomach	363349007	482497012
288641014	B112	B112.00	Malignant neoplasm of pyloric antrum of stomach	Stomach	187740000	288641014
288638017	B1111	B111100	Malignant neoplasm of pyloric canal of stomach	Stomach	187738005	288638017
288635019	B111	B111.00	Malignant neoplasm of pylorus of stomach	Stomach	187736009	288635019
288639013	B111z	B111z00	Malignant neoplasm of pylorus of stomach NOS	Stomach	187736009	288634015
1219534017	B117	B117.00	Malignant neoplasm, overlapping lesion of stomach	Stomach	109836006	1219534017
288649011	B115	B115.00	Malignant tumour of lesser curve of stomach	Stomach	269459004	403678014

721851000006117	B11	B11..00	Malignant tumour of stomach	Stomach	363349007	482498019
288656017	B11z	B11z.00	Malignant tumour of stomach	Stomach	363349007	482498019
291715016	BBQ1z	BBQ1z00	[M]Seminoma NOS	Testicular	443677002	2840468018
174562015	B471	B471.00	Malignant neoplasm of descended testis	Testicular	109876001	174562015
403702017	B47z	B47z.00	Malignant neoplasm of testis NOS	Testicular	363449006	482694014
289223017	B470	B470.00	Malignant neoplasm of undescended testis	Testicular	188219004	289223017
289234019	B470z	B470z00	Malignant neoplasm of undescended testis NOS	Testicular	188219004	289220019
109371000006115	B4711	B471100	Malignant teratoma of descended testis	Testicular	417554000	2548606013
109381000006117	B47z-2	B47z.12	Malignant teratoma of testis	Testicular	416769008	2549298016
721171000006119	B47	B47..00	Malignant tumour of testis	Testicular	363449006	482694014
1216406014	BBQB	BBQB.00	Mixed germ cell tumour	Testicular	32844007	196959015
291714017	BBQ1	BBQ1.00	Seminoma - category	Testicular	443677002	2840468018
457319014	B4710	B471000	Seminoma of descended testis	Testicular	313429000	457319014
380180017	B47z-1	B47z.11	Seminoma of testis	Testicular	255107005	380180017
457318018	B4702	B470200	Seminoma of undescended testis	Testicular	313428008	457318018
291364014	BB4z	BB4z.00	[M]Transitional cell papilloma or carcinoma NOS	Urinary tract	118287003	175970011
292147019	Byu9	Byu9.00	[X]Malignant neoplasm of urinary tract	Urinary tract	419052002	2577717014
6620981000006119	^ESCTBL662098		Bladder cancer	Urinary tract	399326009	1786810016
880461000006118	B49-99	B49..99	Carcinoma bladder	Urinary tract	399326009	880461000006118
5356111000006119	^ESCTCA535611		Carcinoma of bladder	Urinary tract	255108000	380181018
459399016	B498	B498.00	Local recurrence of malignant tumour of urinary bladder	Urinary tract	314968009	459399016
289271017	B493	B493.00	Malignant neoplasm of anterior wall of urinary bladder	Urinary tract	188242006	289271017
289275014	B495	B495.00	Malignant neoplasm of bladder neck	Urinary tract	188244007	289275014
289267015	B491	B491.00	Malignant neoplasm of dome of urinary bladder	Urinary tract	188240003	289267015
289270016	B492	B492.00	Malignant neoplasm of lateral wall of urinary bladder	Urinary tract	188241004	289270016
289279015	B49y	B49y.00	Malignant neoplasm of other site of urinary bladder	Urinary tract	399326009	1773307015
289297011	B4Ay	B4Ay.00	Malignant neoplasm of other urinary organs	Urinary tract	419052002	2577717014
289272012	B494	B494.00	Malignant neoplasm of posterior wall of urinary bladder	Urinary tract	188243001	289272012
722471000006119	B4A10	B4A1000	Malignant neoplasm of renal calyces	Urinary tract	188252005	289289016
289296019	B4A1z	B4A1z00	Malignant neoplasm of renal pelvis NOS	Urinary tract	363457009	482710012
289262014	B490	B490.00	Malignant neoplasm of trigone of urinary bladder	Urinary tract	188239000	289262014
289276010	B496	B496.00	Malignant neoplasm of ureteric orifice	Urinary tract	188245008	289276010
289294016	B4A11	B4A1100	Malignant neoplasm of ureteropelvic junction	Urinary tract	188253000	289294016
720991000006113	B4A3	B4A3.00	Malignant neoplasm of urethra	Urinary tract	363459007	482714015
1786811017	B49	B49..00	Malignant neoplasm of urinary bladder	Urinary tract	399326009	1786811017
289281018	B49z	B49z.00	Malignant neoplasm of urinary bladder NOS	Urinary tract	399326009	1786811017
292148012	Byu90	Byu9000	Malignant neoplasm of urinary organ	Urinary tract	448233000	2899410013
289280017	B49y0	B49y000	Malignant neoplasm, overlapping lesion of bladder	Urinary tract	188247000	289280017
722481000006116	B4A1	B4A1.00	Malignant tumour of renal pelvis	Urinary tract	363457009	482710012
720961000006117	B4A2	B4A2.00	Malignant tumour of ureter	Urinary tract	363458004	482713014
6620961000006112	^ESCTMA662096		Malignant tumour of urinary bladder	Urinary tract	399326009	1773307015
729481000006112	B4A	B4A..00	Malignant tumour of urinary system	Urinary tract	419052002	2577717014
289299014	B4Az	B4Az.00	Malignant tumour of urinary system	Urinary tract	419052002	2577717014
315031000006113	BB4A	BB4A.00	Papillary transitional cell carcinoma	Urinary tract	12400006	21340019
291363015	BB43	BB43.00	Transitional cell carcinoma	Urinary tract	27090000	45317019

1755811000006115	BB4C	BB4C.00	[M]Grade 2 (Stage pTa) papillary urothelial/transitional cell carcinoma	Urinary tract	754361000000102	1667521000000113
5356121000006110	^ESCTTR535612		Transitional cell carcinoma of bladder	Urinary tract	255109008	380182013
317731000006116	BB4	BB4..00	Transitional cell papilloma AND/OR carcinoma	Urinary tract	118287003	175970011
318001000006113	BB43-1	BB43.11	Urothelial carcinoma	Urinary tract	27090000	45320010
4356681000006112	^ESCTAD435668		Adenocarcinoma of endometrium	Uterus	123845008	192334017
880381000006119	B43-99	B43..99	Carcinoma body of uterus	Uterus	371972005	880381000006119
5352211000006114	^ESCTEN535221		Endometrial carcinoma	Uterus	254878006	379735014
307971000006119	BB5j2	BB5j200	Endometrioid carcinoma	Uterus	30289006	50697019
1210561014	B43	B43..00	Malignant neoplasm of body of uterus	Uterus	371972005	1210561014
289198014	B43z	B43z.00	Malignant neoplasm of body of uterus NOS	Uterus	371972005	1210561014
289188015	B4300	B430000	Malignant neoplasm of cornu of corpus uteri	Uterus	188190005	289188015
289192010	B430z	B430z00	Malignant neoplasm of corpus uteri NOS	Uterus	188189001	289187013
289187013	B430	B430.00	Malignant neoplasm of corpus uteri, excluding isthmus	Uterus	188189001	289187013
980021000006113	B4302-1	B430211	Malignant neoplasm of endometrium	Uterus	188192002	289190019
289190019	B4302	B430200	Malignant neoplasm of endometrium of corpus uteri	Uterus	188192002	289190019
289189011	B4301	B430100	Malignant neoplasm of fundus of corpus uteri	Uterus	188191009	289189011
289193017	B431	B431.00	Malignant neoplasm of isthmus of uterine body	Uterus	188195000	289193017
289195012	B431z	B431z00	Malignant neoplasm of isthmus of uterine body NOS	Uterus	188195000	289193017
289194011	B4310	B431000	Malignant neoplasm of lower uterine segment	Uterus	188195000	3491683016
289191015	B4303	B430300	Malignant neoplasm of myometrium of corpus uteri	Uterus	188193007	289191015
289197016	B43y	B43y.00	Malignant neoplasm of other site of uterine body	Uterus	371972005	1210561014
289196013	B432	B432.00	Malignant neoplasm of overlapping lesion of corpus uteri	Uterus	188198003	289196013
289166015	B40	B40..00	Malignant neoplasm of uterus	Uterus	371973000	1210562019
4025691000006117	^ESCTPR402569		Primary malignant neoplasm of endometrium	Uterus	93781006	510605012

Abbreviations: LGI (lower gastrointestinal), HPB (hepatopancreatobiliary), OG (oesophago-gastric), CNS (central nervous system)

Supplementary Code List 3. Codes for the ascertainment of cancer cases the National Cancer Registration and Analysis Service, Office of National Statistics and Hospital Episode Statistics datasets.

Code system	Code	Classification
ICD10	C00	Head and Neck
ICD10	C01	Head and Neck (Oropharyngeal)
ICD10	C02	Head and Neck
ICD10	C03	Head and Neck
ICD10	C04	Head and Neck
ICD10	C05	Head and Neck
ICD10	C06	Head and Neck
ICD10	C07	Head and Neck
ICD10	C08	Head and Neck
ICD10	C09	Head and Neck (Oropharyngeal)
ICD10	C10	Head and Neck (Oropharyngeal)
ICD10	C11	Head and Neck
ICD10	C12	Head and Neck
ICD10	C13	Head and Neck
ICD10	C14	Head and Neck
ICD10	C15	Oesophagus
ICD10	C16	Gastric
ICD10	C17	Other digestive tract
ICD10	C26	Other digestive tract
ICD10	C18	Lower gastrointestinal
ICD10	C19	Lower gastrointestinal
ICD10	C20	Lower gastrointestinal
ICD10	C21	Lower gastrointestinal
ICD10	C22	Hepatobiliary pancreatic
ICD10	C23	Hepatobiliary pancreatic
ICD10	C24	Hepatobiliary pancreatic
ICD10	C25	Hepatobiliary pancreatic
ICD10	C30	Head and Neck
ICD10	C31	Head and Neck
ICD10	C32	Head and Neck
ICD10	C33	Lung
ICD10	C34	Lung
ICD10	C37	Other
ICD10	C38	Other
ICD10	C39	Other
ICD10	C40	Sarcoma
ICD10	C41	Sarcoma
ICD10	C46	Sarcoma
ICD10	C47	Sarcoma
ICD10	C49	Sarcoma
ICD10	C42	Other haematological
ICD10	C43	Melanoma
ICD10	C45	Other
ICD10	C48	Other
ICD10	C50	Breast
ICD10	C51	Gynae
ICD10	C52	Gynae
ICD10	C53	Gynae (Cervix)
ICD10	C54	Gynae (Uterus)
ICD10	C55	Gynae (Uterus)
ICD10	C56	Gynae (Ovary)
ICD10	C57	Gynae
ICD10	C58	Gynae
ICD10	C60	Other
ICD10	C61	Prostate
ICD10	C62	Testicular
ICD10	C63	Other
ICD10	C64	Renal
ICD10	C65	Urinary tract
ICD10	C66	Urinary tract
ICD10	C67	Urinary tract
ICD10	C68	Urinary tract
ICD10	C69	Other
ICD10	C70	Central nervous system
ICD10	C71	Central nervous system
ICD10	C72	Central nervous system
ICD10	C73	Endocrine
ICD10	C74	Endocrine
ICD10	C75	Endocrine
ICD10	C76	Other
ICD10	C77	Other
ICD10	C78	Other
ICD10	C79	Other
ICD10	C80	Other (Cancer of unknown primary C80.0)
ICD10	C81	Lymphoma
ICD10	C82	Lymphoma
ICD10	C83	Lymphoma

ICD10	C84	Lymphoma
ICD10	C85	Lymphoma
ICD10	C86	Lymphoma
ICD10	C88	Lymphoma
ICD10	C90	Other haematological
ICD10	C91	Leukaemia
ICD10	C92	Leukaemia
ICD10	C93	Leukaemia
ICD10	C94	Leukaemia
ICD10	C95	Leukaemia
ICD10	C96	Other haematological
ICD10	C97	Other
ICD9	2090	Other
ICD9	2091	Other
ICD9	2092	Other
ICD9	2093	Other
ICD9	140	Head and Neck (Oropharyngeal 141.0, 141. 6)
ICD9	141	Head and Neck
ICD9	142	Head and Neck
ICD9	143	Head and Neck
ICD9	144	Head and Neck
ICD9	145	Head and Neck
ICD9	146	Head and Neck (Oropharyngeal)
ICD9	147	Head and Neck
ICD9	148	Head and Neck
ICD9	149	Head and Neck
ICD9	150	Oesophagus
ICD9	151	Gastric
ICD9	152	Other digestive tract
ICD9	153	Lower gastrointestinal
ICD9	154	Lower gastrointestinal
ICD9	155	Hepatobiliary pancreatic
ICD9	156	Hepatobiliary pancreatic
ICD9	157	Hepatobiliary pancreatic
ICD9	158	Other
ICD9	159	Other digestive tract
ICD9	160	Head and Neck
ICD9	161	Head and Neck
ICD9	162	Lung
ICD9	163	Other
ICD9	164	Other
ICD9	170	Sarcoma
ICD9	171	Sarcoma
ICD9	176	Sarcoma
ICD9	172	Melanoma
ICD9	174	Breast
ICD9	175	Breast
ICD9	179	Gynae (Uterus)
ICD9	180	Gynae (Cervix)
ICD9	181	Gynae
ICD9	182	Gynae (Uterus)
ICD9	183	Gynae (183.0 Ovary)
ICD9	184	Gynae
ICD9	185	Prostate
ICD9	186	Testicular
ICD9	187	Other
ICD9	188	Urinary tract
ICD9	189	Renal
ICD9	190	Other
ICD9	191	Central nervous system
ICD9	192	Central nervous system
ICD9	193	Endocrine
ICD9	194	Endocrine
ICD9	195	Other
ICD9	196	Other
ICD9	197	Other
ICD9	198	Other
ICD9	199	Other
ICD9	200	Lymphoma
ICD9	201	Lymphoma
ICD9	202	Lymphoma
ICD9	203	Other haematological
ICD9	204	Leukaemia
ICD9	205	Leukaemia
ICD9	206	Leukaemia
ICD9	207	Leukaemia
ICD9	208	Leukaemia
